# VCP‐Regulated LMF2 Stabilizes the Cholesterol Biosynthesis Enzyme HMGCS1 to Drive Cancer Progression and Confer Capecitabine Resistance in Triple‐Negative Breast Cancer

**DOI:** 10.1002/advs.78301

**Published:** 2026-10-20

**Authors:** Qian Zhao, Lisa Andriani, Yin‐Ling Zhang, Shao‐Ying Yang, Yun‐Xiao Ling, Min‐Ying Huang, Jia‐Yang Cai, Fang‐Lin Zhang, Xin Hu, A‐Yong Cao, Zhi‐Min Shao, Guang‐Yu Liu, Da‐Qiang Li

**Affiliations:** ^1^ Cancer Institute Fudan University Shanghai Cancer Center Department of Oncology Shanghai Medical College Fudan University Shanghai China; ^2^ Key Laboratory of Breast Cancer in Shanghai Department of Breast Surgery Fudan University Shanghai Cancer Center Shanghai China; ^3^ Department of Breast and Thyroid Surgery, Shanghai General Hospital, School of Medicine Shanghai Jiao Tong University Shanghai China; ^4^ Institutes of Biomedical Sciences Fudan University Shanghai China; ^5^ Precision Cancer Medicine Center Fudan University Shanghai Cancer Center Shanghai China

**Keywords:** autophagic degradation, cancer progression, chemoresistance, metabolic reprogramming, triple‐negative breast cancer

## Abstract

Triple‐negative breast cancer (TNBC) is the most therapeutically challenging breast cancer subtype owing to its aggressive nature and lack of effective targeted therapies. Here, we identify lipase maturation factor 2 (LMF2), a previously uncharacterized gene in human cancer, as a novel oncogenic driver that promotes tumor growth, metastasis, and resistance to capecitabine in TNBC both in vitro and in vivo. Mechanistically, LMF2 localizes predominantly to the endoplasmic reticulum membrane and is targeted for autophagic degradation mediated by the autophagy receptor NBR1. Valosin‐containing protein (VCP) counteracts this process to stabilize LMF2 by impeding the LMF2‐NBR1 interaction, and pharmacological inhibition of VCP effectively suppressed LMF2‐driven TNBC progression and capecitabine resistance. LMF2, in turn, disrupts the interaction between the autophagy receptor NDP52 and HMGCS1, the rate‐limiting enzyme in cholesterol biosynthesis, thereby shielding HMGCS1 from autophagic degradation and enhancing cholesterol production. Importantly, re‐expression of HMGCS1 or exogenous cholesterol supplementation partially rescued the malignant and chemoresistant phenotypes impaired by LMF2 depletion. Clinically, the VCP‐LMF2‐HMGCS1 axis is significantly co‐upregulated in TNBC patient specimens. Collectively, these findings unveil a previously unrecognized VCP‐LMF2‐HMGCS1 regulatory axis that drives TNBC progression and chemoresistance, and identify LMF2 as a promising therapeutic target for this recalcitrant disease.

## Graphical abstract

LMF2 drives progression and capecitabine resistance in triple‐negative breast cancer (TNBC). Mechanistically, VCP prevents NBR1‐mediated autophagic degradation of LMF2, thereby stabilizing it. Stabilized LMF2 subsequently suppresses NDP52‐mediated autophagic degradation of HMGCS1, a rate‐limiting cholesterol biosynthesis enzyme. Consequently, elevated cholesterol production reinforces tumor progression and therapeutic resistance, identifying the VCP–LMF2–HMGCS1 axis as an actionable target in TNBC.

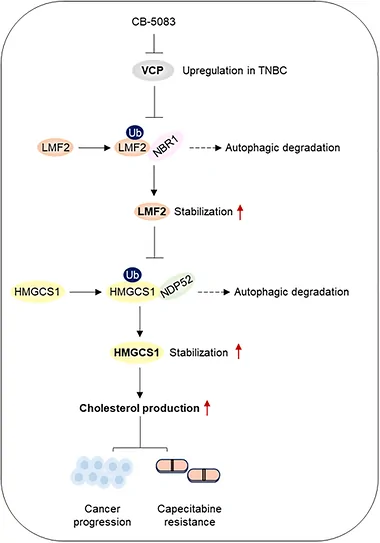

**Abbreviations**
ER: endoplasmic reticulum
HMGCS1: 3‐hydroxy‐3‐methylglutaryl‐coenzyme A synthase 1
IF: immunofluorescence
IP: immunoprecipitation
LMF2: lipase maturation factor 2
NBR1: next to BRCA1 gene 1 protein
NDP52: nuclear dot protein 52
RT‐qPCR: real‐time quantitative PCR
sgRNA: single‐guide RNA
shRNA: short hairpin RNA
siRNA: small interfering RNA
TNBC: triple‐negative breast cancer
VCP: valosin‐containing protein

## 1 Introduction

Triple‐negative breast cancer (TNBC) is a specific subtype of breast cancer, which is defined by the lack of expression of estrogen/progesterone receptors and human epidermal growth factor receptor 2 (HER2) [1]. It accounts for approximately 15%–20% of all breast cancer cases. TNBC is distinguished by its highly aggressive clinical features, marked by pronounced molecular heterogeneity, enhanced invasive and metastatic potential, and a high propensity for early recurrence and distant metastasis, thus collectively contributing to the worst prognosis among breast cancer subtypes [2, 3]. Moreover, the treatment landscape for TNBC remains particularly challenging, as these tumors are unresponsive to endocrine and HER2‐targeted therapies, making chemotherapy the cornerstone of systemic treatment [4]. Within this context, capecitabine, an oral fluoropyrimidine prodrug, has demonstrated significant efficacy as an adjuvant chemotherapy for TNBC in multiple clinical trials [5, 6, 7, 8, 9]. However, its clinical efficacy is frequently undermined by the development of intrinsic or acquired resistance [3, 10]. These clinical challenges underscore the urgent need to unravel the molecular mechanisms driving TNBC progression and chemoresistance as well as to discover novel therapeutic targets that could improve outcomes for patients with this recalcitrant disease.

To address these issues, we conducted an integrative analysis of large‐scale transcriptomic [11] and proteomic [12] datasets of TNBC from our center, along with external TNBC datasets from the Clinical Proteomic Tumor Analysis Consortium (CPTAC) and The Cancer Genome Atlas (TCGA). This approach revealed a significant upregulation of human lipase maturation factor 2 (LMF2) in TNBC tissues, underscoring its potential clinical relevance. However, the structural and functional properties of LMF2 remain poorly characterized to date. According to the annotations in the UniProt protein database (ID: Q9BU23), human LMF2 is a 707‐amino acid protein predicted to localize to the endoplasmic reticulum (ER) membrane and to possess 10 α‐helical transmembrane domains. This multi‑pass transmembrane architecture is characteristic of ER‑resident scaffold proteins that organize membrane‑associated signaling complexes or metabolic enzymes, and is frequently found in proteins that regulate lipid synthesis, transport, or storage at the ER membrane. Although it has been documented that lipase maturation factor 1 (LMF1), a paralog of LMF2, plays a critical role in the folding and transport of lipoprotein lipase and that mutations in LMF1 cause combined lipase deficiency and severe hypertriglyceridemia [13, 14]. A recent genome‑wide association study has implicated the LMF2 locus in non‑alcoholic fatty liver disease and cholesterol metabolism [15], pointing to a potentially evolutionarily conserved function of the LMF family in lipid homeostasis. In addition, a functional study demonstrated that knockdown of an LMF2‐like gene in the aphids reduces the survivability and reproductive capacity of the grain aphid, highlighting its potential as a target for pest control [16]. Collectively, the biological functions and regulatory mechanisms of LMF2 in human cancer, particularly in TNBC progression and chemoresistance, remain entirely unexplored and merit further investigation.

Metabolic reprogramming is a well‐established hallmark of human cancers. Accumulating evidence shows that TNBC exhibits hyperactivation of the cholesterol biosynthesis pathway, which has been strongly linked to its disease progression and therapeutic resistance [17, 18, 19, 20, 21]. This metabolic pathway begins with acetyl‐coenzyme A (acetyl‐CoA) and involves the coordinated action of over 20 enzymes, the majority of which are localized to the ER membrane [22]. Notably, 3‐hydroxy‐3‐methylglutaryl‐CoA synthase 1 (HMGCS1) is the first rate‐limiting enzyme in this pathway, which catalyzes the condensation of acetyl‐CoA and acetoacetyl‐CoA to form 3‐hydroxy‐3‐methylglutaryl‐CoA (HMG‐CoA) [23, 24]. Subsequently, HMG‐CoA is converted by HMG‐CoA reductase (HMGCR) into mevalonate, a key precursor for cholesterol synthesis [22]. Cholesterol serves as an essential biomolecule that not only maintains membrane integrity and fluidity but also acts as a key signaling molecule regulating multiple cellular processes [25]. Therefore, targeting the cholesterol biosynthesis pathway not only effectively suppresses TNBC progression but also significantly enhances the sensitivity of TNBC cells to anticancer therapies [17, 20, 26, 27].

As the first committed enzyme in cholesterol biosynthesis, HMGCS1 is tightly regulated at multiple levels, including transcription, translation, and post‐translational modifications. Notably, it has been documented that acetylation of HMGCS1 at lysine 273 by lysine acetyltransferase 5 enhances its protein stability [28], and that HMGCS1 undergoes ubiquitin‐dependent proteasomal degradation mediated by the E3 ubiquitin‐protein ligases SPOP [29] and CTLH [24]. Nevertheless, the precise molecular mechanisms governing HMGCS1 protein stability as well as its functional roles in TNBC progression and chemoresistance remain largely elusive. Given that HMGCS1 requires ER membrane association for its catalytic activity, we reasoned that its post‑translational stability would be intimately linked to the ER‑resident quality control machinery. LMF2, with its ER localization and multi‑pass transmembrane topology, is ideally situated to interact with or stabilize neighboring metabolic enzymes, a feature that directly prompted us to examine its role in HMGCS1 stability and cholesterol biosynthesis.

Autophagy is a highly conserved lysosomal degradation pathway in mammalian cells and can function through both non‐selective and selective mechanisms. Non‐selective autophagy is typically induced by nutrient deprivation and mediates the bulk degradation of cytoplasmic contents to replenish vital metabolites [30, 31]. In contrast, selective autophagy employs specific cargo receptors, such as sequestosome 1 (SQSTM1/p62), neighbor of BRCA1 gene 1 protein (NBR1), nuclear dot protein 52 (NDP52/CALCOCO2), optineurin (OPTN), Tax1 binding protein 1 (TAX1BP1), and toll‐interacting protein (TOLLIP), to identify and transport particular cellular substrates to lysosomes for degradation [32, 33]. Through these diverse mechanisms, autophagy enables cancer cells to adapt to proteotoxic and metabolic stresses, thereby supporting tumor survival, progression, and resistance to anticancer therapies [30, 31].

In this study, we present the first evidence that LMF2 acts as a novel oncogenic driver in TNBC to promote tumor growth, metastasis, and resistance to capecitabine. Mechanistically, we demonstrated that valosin‐containing protein (VCP/p97), a fundamental regulator of protein homeostasis and an emerging therapeutic target in human cancers [34, 35, 36], functions as an upstream stabilizer of LMF2 in TNBC cells by inhibiting its autophagic degradation via the cargo receptor NBR1. LMF2, in turn, stabilizes HMGCS1 through blocking its autophagic degradation mediated by the receptor NDP52 and enhances cholesterol production. Importantly, suppression of VCP abolished LMF2‐driven oncogenic phenotypes, while either re‐expression of HMGCS1 or exogenous cholesterol supplementation rescued the effects of LMF2 depletion. The consistent co‐upregulation of the VCP‐LMF2‐HMGCS1 axis in human TNBC specimens further underscores the clinical relevance of our findings. Collectively, these findings identify the VCP‐LMF2‐HMGCS1 axis as a critical regulator of TNBC progression and chemoresistance, and highlight LMF2 as a promising therapeutic target in TNBC.

## 2 Methods and Materials

### 2.1 Cell Culture and Reagents

Human TNBC cell lines, including BT20 (RRID:CVCL_0178), BT549 (RRID:CVCL_1092), HCC1806 (RRID:CVCL_1258), Hs578T (RRID:CVCL_0332), LM2‐4175 (RRID:CVCL_5998), MDA‐MB‐231 (hereafter abbreviated as MDA‐231, RRID:CVCL_0062), and SUM‐159 (RRID:CVCL_5423), as well as the human embryonic kidney 293T (HEK293T, RRID:CVCL_0063), were obtained from the Shanghai Key Laboratory of Breast Cancer, Fudan University Shanghai Cancer Center (FUSCC). Cell lines were authenticated using short tandem repeat profiling and confirmed to be free of cross‐contamination against reference databases. All cell lines were routinely tested negative for mycoplasma contamination via PCR assays and were maintained for fewer than 15 passages after resuscitation for all experimental evaluations. All cell lines were cultured in appropriate media under standard conditions as described previously [37]. A comprehensive list of the chemical reagents used in this study is provided in Table S1.

### 2.2 Clinical Samples and Multi‐Omics Datasets

All procedures involving human samples were reviewed and approved by the Medical Ethics Committee of Fudan University Shanghai Cancer Center (SCCIRB; approval no. NSFC‐25‐0303‐68). TNBC tissues and matched adjacent normal tissues were collected from patients who underwent surgical resection at FUSCC. Written informed consent was obtained from all patients prior to study enrollment. The RNA‐sequencing (RNA‐seq) [11] and quantitative proteomic [12] datasets of TNBC from the FUSCC‐TNBC cohort, as well as transcriptomic and proteomic datasets of all breast cancer types from the Chinese Breast Cancer Genome Atlas (CBCGA) Project cohort [38], have been described previously. Additionally, external transcriptomic and proteomic datasets for TNBC were sourced from The Cancer Genome Atlas (TCGA; https://tcga‐data.nci.nih.gov/tcga/) [39] and the Clinical Proteomic Tumor Analysis Consortium (CPTAC; https://proteomics.cancer.gov/data‐portal) [40], respectively. Transcriptomic datasets for all subtypes of breast cancer was obtained from the Molecular Taxonomy of the Breast Cancer International Consortium (METABRIC) (http://cbioportal.org) [41]. To investigate the association between LMF2 expression levels and drug response, we interrogated publicly available data from the Genomics of Drug Sensitivity in Cancer (GDSC) database (https://www.cancerrxgene.org/) [42]. Detailed information for all datasets analyzed in this study is provided in Table S2.

### 2.3 Generation and Transfection of Plasmid DNA Constructs

Full‐length human LMF2 and HMGCS1 cDNAs were amplified and subcloned into the Ubi‐MCS‐3FLAG‐CBh‐gcGFP‐IRES‐puromycin (#GV492, GeneChem, Shanghai) and pLVX‐IRES‐Neo lentiviral vector (#632181, Biofeng) to generate Flag‐LMF2 and HA‐HMGCS1, respectively. Truncated Flag‐LMF2 (NT and CT) and HA‐HMGCS1 (NT and CT) constructs were synthesized by Suzhou Hongxun Biotech (Suzhou, China) using their respective full‐length plasmids as templates. HA‐NBR1 and its truncation mutants (HA‐NBR1△PB1 and HA‐NBR1△UBA) were constructed using the same strategy and cloned into the pLVX‐IRES‐Neo lentiviral vector. Full‐length human NDP52 and its truncation mutants (△SKICH, △CC, and △LIM‐L) were amplified and subcloned into the pCDH‐CMV‐EF1‐Puro lentiviral vector (#CD510B‐1, System Biosciences) to generate the corresponding Flag‐tagged constructs. The HA‐TRIM21, HA‐VCP‐E578Q, and HA‐HMGCS1‐K273R expression plasmids were purchased from Suzhou Hongxun Biotech (Suzhou, China). Short hairpin RNAs (shRNAs) targeting LMF2 were designed and synthesized by Sigma–Aldrich Advanced Genomics and cloned into the pLKO.1‐TRC lentiviral vector (#10878, Addgene). The shVCP (#3 and #4) and HA‐VCP plasmids have been described previously [37]. Single‐guide RNAs (sgRNAs) targeting ATG5, NBR1, SQSTM1/p62, NDP52, and TOLLIP were designed and synthesized by Suzhou Hongxun Biotech (Suzhou, China). Small interfering RNAs (siRNAs) used in this study were synthesized by GenePharma (Shanghai, China). The primer sequences used for molecular cloning in this study are provided in Table S3. The targeting sequences for shRNAs, sgRNAs, and siRNAs are listed in Tables S4–S6, respectively.

Plasmid transient transfection was carried out using Tenfect DNA transfection reagent (#FT19301, TEYE Biotech) according to the manufacturer's instructions. To establish stable cell lines, lentiviral expression constructs were cotransfected with packaging plasmids into HEK293T cells for virus production. After 48 h, viral supernatants were collected and used to infect the indicated target cells in the presence of polybrene (#H9268, MilliporeSigma). Infected cells were selected with puromycin or G418 for approximately 2 weeks to generate stable populations. The efficiency of gene overexpression or knockdown was verified by immunoblotting.

### 2.4 Antibodies, Immunoblotting and Immunoprecipitation Assays

A detailed list of antibodies used in this study is provided in Table S7. Total proteins were extracted using RIPA lysis buffer supplemented with protease and phosphatase inhibitor cocktails (#B14002 and #B15003, Bimake), and protein concentrations were determined with a BCA assay kit (#20201ES90, Yeasen) according to the manufacturer's instructions. Equal amounts of protein were resolved by SDS‐PAGE and transferred onto PVDF membranes (#IPVH00010, Millipore). Membranes were blocked with 5% bovine serum albumin (BSA) (#69100, Proliant Biol) and incubated with primary antibodies at 4°C overnight, followed by appropriate secondary antibodies. Protein signals were visualized using an enhanced chemiluminescence detection kit (#180‐5001E, Tanon).

For exogenous immunoprecipitation (IP) assays, proteins were extracted using NP‐40 lysis buffer. Equal amounts of lysates were incubated with anti‐Flag magnetic beads (#GNI4510‐FG, Shanghai Genomics Technology), anti‐HA magnetic beads (#GNI4510‐HA‐P, Shanghai Genomics Technology), or anti‐HA nanobody agarose beads (#KTSM1305, AlpalifeBio, Shenzhen, China) at 4°C for at least 3 h with gentle rotation. After extensive washing, the immunoprecipitates were eluted and subjected to immunoblotting assays. For endogenous IP assays, cell lysates were incubated with specific primary antibodies overnight at 4°C, followed by protein A/G magnetic beads (#B23202, Bimake) for at least 3 h. After thorough washing, complexes were analyzed by immunoblotting.

### 2.5 RNA Extraction and Real‐Time Quantitative PCR (RT–qPCR) Assays

Total RNA was extracted using RNAiso Plus reagent (#9109, Takara) according to the manufacturer's protocol. Complementary DNA (cDNA) was synthesized from purified RNA using the HiScript III RT SuperMix kit (#R323‐01, Vazyme). RT‐qPCR assays were then performed using ChamQ Universal SYBR qPCR Master Mix (#Q711‐03, Vazyme) on a QuantStudio system (Applied Biosystems). All primers used in this study were designed and synthesized by HuaGene Biotech (Shanghai, China) (Table S8).

### 2.6 Immunofluorescent (IF) Staining

For IF staining, cells were washed with PBS, fixed with 4% paraformaldehyde (#E672002‐0500, Sangon Biotech), and permeabilized with 0.1% Triton X‐100 (#93443, Sigma–Aldrich). After blocking with 5% BSA, cells were incubated with primary antibodies overnight at 4°C, followed by fluorescent secondary antibodies for 1 h at room temperature. Nuclei were counterstained with DAPI (#ab104139, Abcam), and samples were mounted using Fluoroshield. Images were acquired using a Leica SP5 confocal microscope.

### 2.7 Immunohistochemical (IHC) Staining

IHC staining was conducted as described previously [43, 44], using primary antibodies against VCP, LMF2, and HMGCS1. 3,3’‐Diaminobenzidine (DAB)‐stained IHC images were quantified by calculating the percentage of DAB‐positive area using a color deconvolution method. Raw images were converted to optical density and separated into hematoxylin and DAB channels. The DAB channel was binarized using a manually selected threshold, and the DAB‐positive area fraction was computed [45]. All images were processed with a custom Python script, and the resulting fractions were used for further statistical analysis.

### 2.8 Proteomics Analysis

To identify proteins interacting with LMF2, HEK293T cells stably expressing Flag‐ LMF2 were lysed, followed by IP assays using anti‐Flag antibodies. Immunoprecipitated protein complexes were separated by SDS‐PAGE and visualized using a silver staining kit (#24612, Thermo Fisher Scientific, USA) following the manufacturer's protocol. Distinct bands were excised, destained, and subjected to in‐gel enzymatic digestion. The digested peptides were then analyzed by liquid chromatography‐tandem mass spectrometry (LC‐MS/MS) to identify potential interacting proteins.

To profile LMF2‐regulated downstream proteins, LM2‐4175 cells stably expressing two independent shRNAs targeting LMF2 (shLMF2 #4 and #5) or a negative control shRNA (shNC) were lysed in RIPA buffer. Equal amounts of protein were processed for isobaric tags for relative and absolute quantification (iTRAQ)‐based proteomic analysis.

### 2.9 Cycloheximide (CHX) Chase and Ubiquitination Assays

Determination of protein turnover was conducted by CHX chase assays as described previously [46]. For ubiquitination assays, HEK293T cells were transfected with the indicated plasmids for 48 h, followed by treatment with 10 µM MG‐132 for 6 h. Ubiquitination assays were then performed as previously described [47].

### 2.10 Measurement of Cholesterol Levels

After 24 h of culture at comparable cell densities, conditioned media from cultured cells were collected and centrifuged at 1000 × g for 20 min at 4°C to remove cellular debris. The resulting clarified supernatants were used to quantify cholesterol levels using a commercial cholesterol assay kit (#YJ594932B, mlbio) according to the manufacturer's protocol.

### 2.11 Measurement of Lipoprotein Lipase (LPL) Activity

After cell culture, cell lysates were collected and centrifuged at 12 000 × g for 3–5 min at 4°C to remove cellular debris. The resulting clarified supernatants were used to measure LPL activity using a commercial Lipoprotein Lipase Activity Assay Kit (#P2775S, Beyotime) according to the manufacturer's protocol.

### 2.12 Exogenous Cholesterol Supplementation Experiments

Cells were treated with 2.5 µg/mL soluble cholesterol (#S4154, Selleck) for 24 h prior to functional assays following protocols adapted from prior studies [18].

### 2.13 Cell Proliferation and Colony Formation Assays

Cells were trypsinized, counted, and seeded into 96‐well plates (1000 cells/well) for proliferation assays. Drug sensitivity was assessed by plating 2000–2500 cells per well in 96‐well plates, followed by treatment with the indicated compounds after overnight incubation. Cell viability was assessed using the CCK‐8 kit (#40203ES92, Yeasen), and absorbance at 450 nm (A450) was measured using a microplate reader. For colony formation assays, 1000 cells were plated in 12‐well plates (three replicates per group) and cultured for 12–14 days. Colonies were fixed in methanol and stained with 0.2% crystal violet for visualization and quantification.

### 2.14 Cell Migration and Invasion Assays

Migration assays were performed by seeding 2 × 10^4^ cells in 200 µL of serum‐free medium into the upper chamber of Transwell inserts without Matrigel coating (#353097, BD Falcon). Invasion assays were carried out by plating 3 × 10^4^ cells suspended in 200 µL of serum‐free medium into the upper chamber pre‐coated with Matrigel (#354480, BD Biocoat). In both assays, the lower chamber contained 800 µL of medium supplemented with 10% FBS as a chemoattractant. After 18–24 h of incubation, cells that traversed the membranes were fixed with methanol and stained with 0.2% crystal violet for visualization.

### 2.15 Xenograft Tumor Models and Treatment

All animal experimental procedures were approved by the Institutional Animal Care and Use Committee (IACUC) of Fudan University (approval no. FUSCC‐IACUC‐S2025‐0519) and performed in accordance with institutional guidelines for the care and use of laboratory animals. To evaluate tumorigenic potential, LM2‐4175 cells (1 × 10^6^) were orthotopically injected into the mammary fat pads of 6‐week‐old female BALB/c nude mice (*n* = 10). Tumor formation and growth were monitored regularly, and tumor volumes were measured using calipers and calculated as (length × width^2^)/2.

For in vivo drug sensitivity assays, LM2‐4175 (1 × 10^6^; *n* = 6) and MDA‐231 (3 × 10^6^; *n* = 6) xenograft models were established. Once tumors reached approximately 50–100 mm^3^, LM2‐4175–bearing mice were treated with capecitabine (250 mg/kg) or vehicle by oral gavage daily for 12 consecutive days, based on a previously reported dosing regimen in breast cancer xenograft models [48]. Separately, MDA‐231 bearing mice were treated with CB‐5083 (50 mg/kg) or vehicle by oral gavage once daily, starting approximately 1 week after tumor cell injection, following a 5‐days‐on/2‐days‐off schedule for 4 weeks [37]. For combination treatment studies, MDA‐231 orthotopic xenograft models were established as described above. Mice were randomly assigned to receive vehicle, capecitabine (250 mg/kg), or the combination of capecitabine (250 mg/kg) and CB‐5083 (50 mg/kg). Previous studies have demonstrated that oral capecitabine is well tolerated across a dose range from 0.52 mmol/kg (187 mg/kg) to 2.1 mmol/kg (755 mg/kg) given daily in nude mouse xenograft models [49, 50]. In addition, CB‐5083 administered continuously at 70–75 mg/kg daily is safe and well tolerated in nude mice and yields stable longitudinal body weight curves throughout the treatment course without any treatment‐related weight loss [51, 52]. Drug treatment began approximately 1 week after tumor cell injection and continued for four consecutive weeks, administered once daily following a 5‐days‐on/2‐days‐off schedule. Tumor volumes were measured every 3 days using the formula: volume = (length × width^2^)/2.

For lung metastasis experiments, LM2‐4175 (5 × 10^5^) or MDA‐231 (1 × 10^6^) cells were injected into the tail vein of 7‐week‐old female BALB/c nude mice (*n* = 6). One week after cell injection, mice received vehicle or CB‐5083 (50 mg/kg) via oral gavage, administered once daily for five consecutive days followed by a two‐day break (qd5/2 off) for a total of 4 weeks. Mice were sacrificed when body weight loss exceeded 10% or signs of cachexia were observed, and lung tissues were collected for further analysis.

### 2.16 Statistical Analysis

All data are presented as the mean ± standard deviation (SD) from at least three independent biological replicates, with sample sizes (n) indicated in the figure legends. Normal distribution and homogeneity of variance were assessed using the Shapiro–Wilk and Brown–Forsythe tests, respectively; no outliers were excluded. Comparisons between two groups were evaluated using two‐tailed unpaired/paired Student's *t*‐test (or Welch's *t*‐test) for parametric data, and Mann–Whitney U test or Wilcoxon matched‐pairs signed‐rank test for non‐parametric data. Multiple group comparisons were conducted using one‐way or two‐way ANOVA followed by Dunnett's or Tukey's post‐hoc test, or Kruskal–Wallis test with Dunn's post‐hoc test for non‐parametric data. Spearman's rank correlation was used to evaluate associations between variables. A two‐sided *p*‐value < 0.05 was considered statistically significant (*, *p* < 0.05; **, *p* < 0.01; ***, *p* < 0.001; ns, no significance). Specifically, experimental data analysis and plots were generated using GraphPad Prism (version 9.4) and custom Python scripts (version 3.9); image quantifications were performed using ImageJ (version 1.8.0), and figure panels alongside schematic illustrations were processed and assembled using Microsoft PowerPoint (version 2021).

## 3 Results

### 3.1 LMF2 Localizes Primarily to the ER Membrane and is Markedly Upregulated in TNBC Tissues

The ER is one of the largest membrane‐bound organelles in eukaryotic cells and serves as a central hub for the synthesis, modification, and secretion of cholesterol and lipoproteins [53]. Moreover, growing evidence has shown that dysregulated lipid metabolism plays a pivotal role in tumor initiation, progression, metastasis, and resistance to therapies [54, 55]. To elucidate the molecular mechanisms underlying TNBC progression and therapeutic resistance and to identify potential therapeutic targets, we conducted an integrated analysis of five independent datasets. These include an ER‐localized protein dataset (GO: 0005783), transcriptomic datasets of TNBC from our center (FUSCC) [17, 18, 19, 20, 21] and TCGA [39], as well as proteomic TNBC datasets from FUSCC [22] and CPTAC [40]. This approach identified 126 ER‐localized proteins that were consistently overexpressed in TNBC tissues (Figure 1A). The top 15 proteins with the highest tumor‐to‐normal tissue expression ratios based on the FUSCC proteomic dataset [12] are shown in Figure 1B. Among these, LMF2 was selected for further functional investigation owing to its marked upregulation in TNBC tissues and the absence of functional or mechanistic studies on its role in human cancers, underscoring its potential as a novel and clinically relevant target.

**FIGURE 1:**
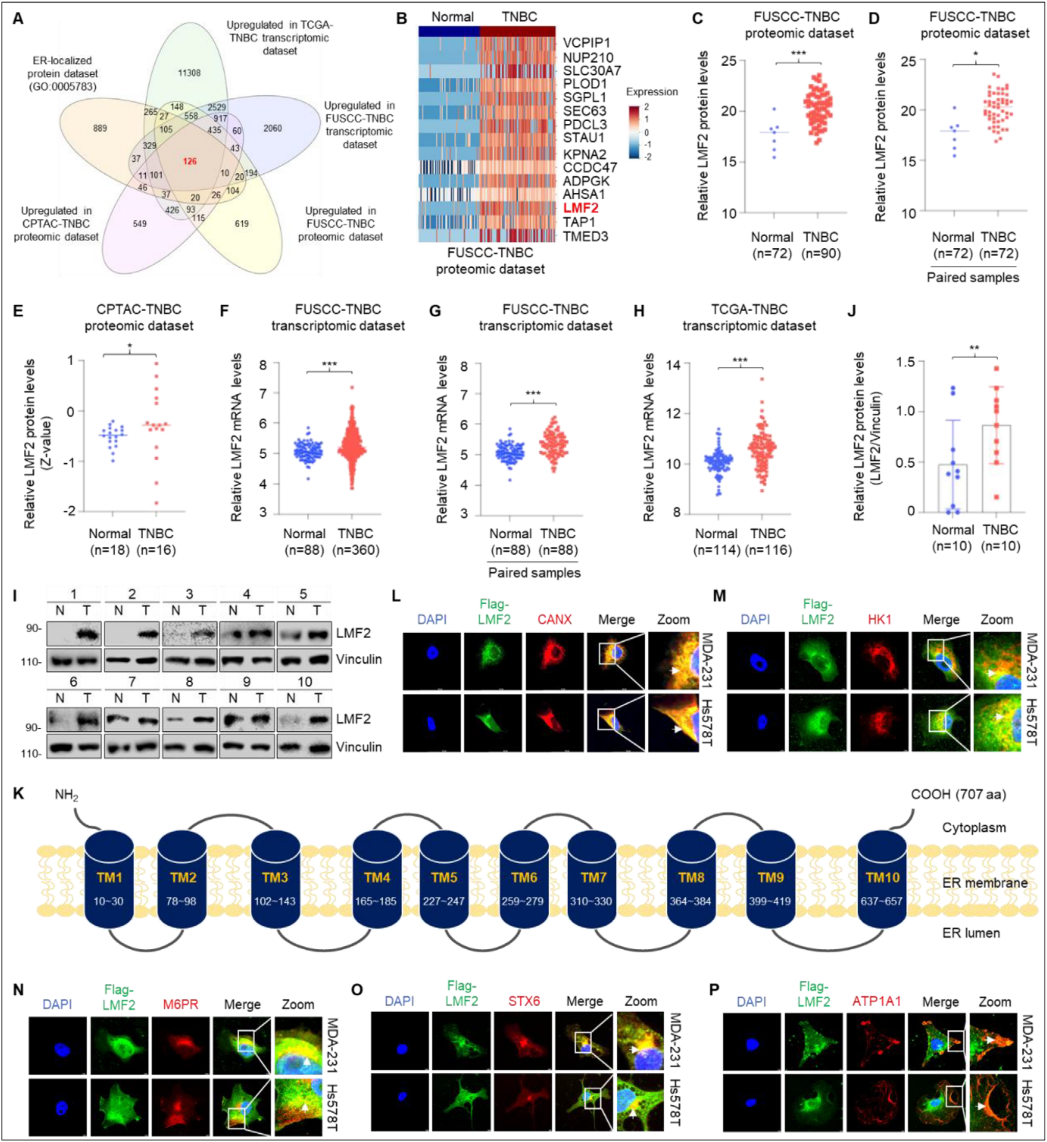
LMF2 localizes primarily to the ER membrane and is markedly upregulated in TNBC tissues. (A) Identification of 126 upregulated ER‐localized proteins in TNBC by integrated analyses of five independent datasets. (B) Heatmap showing the top 15 proteins with the highest tumor‐to‐normal tissue expression ratios based on the FUSCC proteomic dataset. (C, D) LMF2 protein levels in 90 TNBC samples and 72 adjacent normal tissues from the FUSCC‐TNBC proteomic dataset. Of note, LMF2 protein was detectable in only 7 out of the 72 normal samples and 62 out of the 90 TNBC samples, which is likely attributable to its extremely low expression levels in normal breast tissues and the inherent detection limits of high‑throughput mass spectrometry. Data are presented as medians with individual data points. (C) Comparison of LMF2 protein levels between detectable normal tissues (*n* = 7) and TNBC tissues (*n* = 62) analyzed by a two‐tailed Mann–Whitney U test. (D) Comparison of LMF2 protein levels in paired adjacent normal and TNBC tissues analyzed by two‐tailed Wilcoxon matched‐pairs signed‐rank test. (E) Relative LMF2 protein levels (Z‐score) in normal tissues (*n* = 18) and TNBC tissues (*n* = 16) from the CPTAC proteomic dataset. Data are presented as medians with individual data points and analyzed by a two‐tailed Mann–Whitney U test. (F, G) LMF2 mRNA levels in 360 TNBC samples and 88 adjacent normal tissues from the FUSCC‐TNBC transcriptomic dataset. Data are presented as medians with individual data points. (F) Comparison of LMF2 mRNA levels between adjacent normal tissues (*n* = 88) and TNBC tissues (*n* = 359, one sample excluded due to missing value) analyzed by two‐tailed Mann–Whitney U test. (G) Comparison of LMF2 mRNA levels in paired adjacent normal and TNBC tissues (*n* = 87 pairs, one pair excluded due to incomplete paired data/missing value) analyzed by two‐tailed Wilcoxon matched‐pairs signed‐rank test. (H) Relative LMF2 mRNA levels in adjacent normal tissues (*n* = 114) and TNBC tissues (*n* = 116) from the TCGA‐TNBC transcriptomic dataset. Data are presented as medians with individual data points and analyzed by a two‐tailed Mann–Whitney U test. (I, J) Immunoblotting analysis of LMF2 protein levels in 10 paired TNBC and adjacent normal tissues. Relative LMF2 expression levels (LMF2/Vinculin) were quantified with ImageJ software and are presented in J (data are presented as mean ± SD with individual data points, analyzed by paired two‐tailed Student's *t*‐test). (K) Schematic diagram of the predicted LMF2 topology, showing 10 transmembrane (TM) domains and ER localization. (L–P) MDA‐231 and Hs578T cells stably expressing Flag‐LMF2 were subjected to immunofluorescent staining with specific antibodies against Flag (green) and cell membrane markers (red), including CANX (ER membrane marker), HK1 (mitochondrial outer membrane marker), M6PR (lysosomal membrane marker), STX6 (Golgi membrane marker), and ATP1A1 (plasma membrane marker). DNA was counterstained with DAPI (blue). Colocalization of Flag‐LMF2 with cell membrane markers is indicated by the yellow signal. Scale bars: 50 µm (CANX); 5 µm (others). Notes: *, **, and *** indicate statistical significance at *p* < 0.05, *p* < 0.01, and *p* < 0.001 levels, respectively.

Next, we conducted a detailed analysis of LMF2 expression levels in TNBC tissues. An analysis of the TNBC proteomic dataset from the FUSCC cohort [12] showed that LMF2 expression was significantly elevated in tumor tissues relative to adjacent normal tissues. This upregulation was consistently observed in both unpaired and paired sample analyses (Figure 1C,D). This finding was further validated in an independent TNBC dataset from the CPTAC cohort [40], which also demonstrated an upregulation of LMF2 protein levels in TNBC tissues (Figure 1E). Correspondingly, transcriptomic analyses of TNBC datasets from both FUSCC [11] and TCGA [39] cohorts showed elevated LMF2 mRNA levels in TNBC tissues (Figure 1F,H). To further validate these findings, we detected LMF2 protein expression levels in 10 paired clinical TNBC samples and their matched adjacent normal tissues using immunoblotting assays. The results confirmed that LMF2 protein expression levels were significantly higher in TNBC tissues in the majority of cases (Figure 1I,J).

To date, the structural and functional characteristics of LMF2 remain largely unexplored. According to the annotations from the UniProt protein database (https://www.uniprot.org/) and the predictions from the Protter protein visualization tool (http://wlab.ethz.ch/protter), LMF2 is characterized as a multi‐pass transmembrane (TM) protein that is primarily localized to the ER membrane with ten predicted TM domains (designated TM1 to TM10) (Figure 1K). In line with this prediction, immunofluorescent staining demonstrated strong colocalization of LMF2 with the ER membrane marker calnexin (CANX) (Figure 1L, the yellow area indicated by the white arrows in the zoomed‐in images). In contrast, we observed significantly weaker colocalization of LMF2 with the mitochondrial outer membrane marker hexokinase 1 (HK1) (Figure 1M), the lysosomal membrane marker cation‐independent mannose‐6‐phosphate receptor (M6PR) (Figure 1N), and the Golgi membrane marker syntaxin 6 (STX6) (Figure 1O). Furthermore, LMF2 exhibited no notable colocalization with the plasma membrane marker sodium/potassium‐transporting ATPase subunit alpha 1 (ATP1A1) (Figure 1P). Together, these results suggest that LMF2 is primarily localized to the ER membrane and is upregulated in TNBC tissues.

To determine whether LMF2 is specifically upregulated in TNBC, we examined its mRNA and protein levels across three major breast cancer subtypes, including hormone receptor‑positive (HR+), HER2‑positive (HER2+), and TNBC, using publicly available transcriptomic and proteomic data from multiple large‑scale cohorts, including CBCGA (our center) [38], TCGA [39], METABRIC [41], and CPTAC [40]. The results revealed no apparent subtype‑specific differences in LMF2 expression at either the mRNA or protein level (Figure S1A,B), suggesting that LMF2 may exert broader functional roles in breast cancer beyond the TNBC subtype. Of note, the present study focused on addressing the functional and mechanistic roles of LMF2 in TNBC due to its high aggressiveness and the lack of established therapeutic targets. Nevertheless, its potential roles in other breast cancer subtypes remain to be further elucidated in the future.

### 3.2 LMF2 Drives the Malignant Progression of TNBC Cells Both In Vitro and In Vivo

To investigate the impacts of LMF2 on the biological behaviors of TNBC cells, we first assessed the protein expression levels of LMF2 in 7 representative TNBC cell lines by immunoblotting assays (Figure S2A). Based on this result, we stably overexpressed either an empty vector (EV) or Flag‐tagged LMF2 (Flag‐LMF2) in MDA‐231 and Hs578T cells, which exhibit low endogenous LMF2 expression levels. Conversely, we knocked down LMF2 in LM2‐4175 and BT549 cells, which display high endogenous levels of LMF2. The efficiency of LMF2 overexpression and knockdown in these generated cell lines was validated by immunoblotting assays (Figure 2A,B).

**FIGURE 2:**
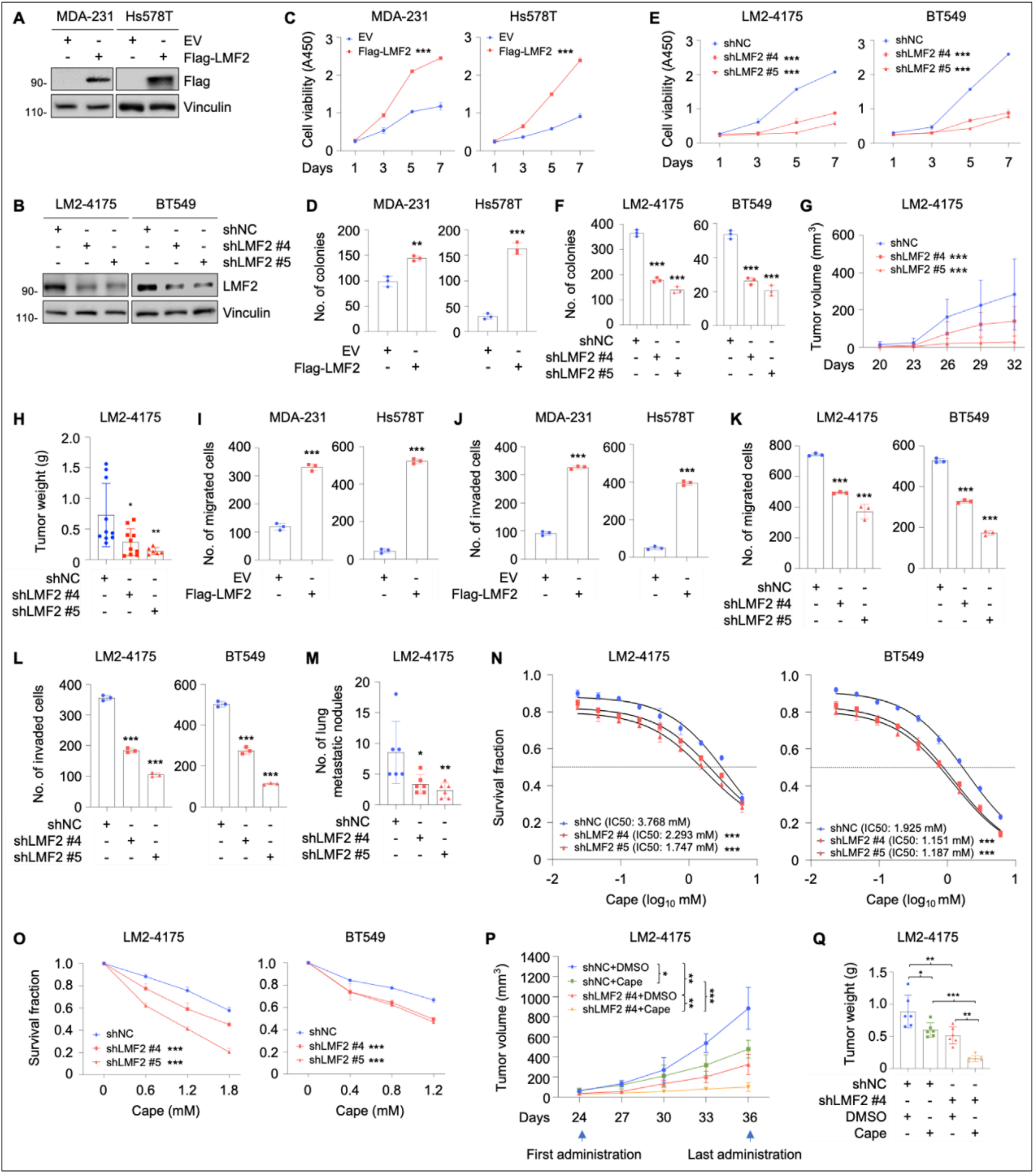
LMF2 promotes cancer progression and confers resistance to capecitabine in TNBC both in vitro and in vivo. (A, ‐B) Validation of the expression status of LMF2 in LMF2‐overexpressing MDA‐231 and Hs578T cells (A) and LMF2‐depleted LM2‐4175 and BT549 cells (B) by immunoblotting assays. EV, empty vector. (C, D) MDA‐231 and Hs578T stably expressing LMF2 were subjected to CCK‐8 (C) and colony formation (D) assays. The representative images of survival colonies are shown in Figure S1B, and the corresponding quantitative results are shown in D. Data are presented as mean ± SD (*n* = 3 independent experiments). Statistical significance for cell viability in (C) was determined by two‐way ANOVA followed by Šídák's multiple comparisons test (significant differences at Day 7 are shown) and two‐tailed unpaired Student's *t*‐test in (D). (E, F) LMF2‐depleted LM2‐4175 and BT549 cells were subjected to CCK‐8 (E) and colony formation (F) assays. The representative images of survival colonies are shown in Figure S1C, and the corresponding quantitative results are shown in (F). Data are presented as mean ± SD (*n* = 3 independent experiments). Statistical significance for cell viability in (E) was determined by two‐way ANOVA followed by Šídák's multiple comparisons test (significant differences at Day 7 are shown) and one‐way ANOVA followed by Tukey's multiple comparisons test in (F). (G, H) LM2‐4175 cells stably expressing shNC or shLMF2 (#4 and #5) were orthotopically injected into the mammary fat pad of 6‐week‐old BALB/c female nude mice (*n* = 10). Tumor volume and weight are shown in G and H, respectively. The representative images of removed xenograft tumors are shown in Figure S1D. Data are presented as mean ± SD (or median with interquartile range for (H). Statistical significance for tumor volume in (G) was determined by two‐way ANOVA followed by Dunnett's multiple comparisons test (significant differences at Day 32 are shown) , and non‐parametric Kruskal‐Wallis test followed by Dunn's multiple comparisons test for tumor weight in (H). (I, J) MDA‐231 and Hs578T stably expressing LMF2 were subjected to Transwell migration (I) and invasion (J) assays. The corresponding quantitative results are shown. The representative images of migrated and invaded cells are provided in Figure S1E. Data are presented as mean ± SD (*n* = 3 independent experiments). Statistical significance was determined by a two‐tailed unpaired Student's *t*‐test. (K, L) LMF2‐depleted LM2‐4175 and BT549 cells were subjected to Transwell migration (K) and invasion (L) assays. The corresponding quantitative results are shown. The representative images of migrated and invaded cells are provided in Figure S1F. Data are presented as mean ± SD (*n* = 3 independent experiments). Statistical significance was determined by ordinary one‐way ANOVA followed by Šídák's multiple comparisons test. (M) LM2‐4175 cells stably expressing shNC or shLMF2 (#4 and #5) were injected into the tail vein of 7‐week‐old female BALB/c nude mice (*n* = 6). Mice were sacrificed 6 weeks post‐injection, and lungs were collected. Quantitative results of metastatic lung nodules are shown. The representative images of lung metastases are presented in Figure S1G. Data are presented as mean ± SD (*n* = 6). Statistical differences between the control and individual knockdown groups were evaluated by a two‐tailed Mann‐Whitney test. (N) LM2‐4175 and BT549 cells stably expressing shNC or shLMF2 (#4 and #5) were treated with increasing concentrations of capecitabine (Cape) for 72 h. Cell viability was measured by CCK‐8 assays, and IC_50_ values were calculated by nonlinear regression. Data are presented as mean ± SD (*n* = 3). Statistical significance of differences in IC_50_ curves compared with the shNC group was determined by an extra sum‐of‐squares *F*‐test. (O) LM2‐4175 and BT549 cells stably expressing shNC or shLMF2 (#4 and #5) were subjected to clonogenic survival assays with increasing concentrations of capecitabine (Cape) for 7–14 days. The representative images of survival colonies are shown in Figure S2B. Data are presented as mean ± SD (*n* = 3). Statistical significance of differences across curves compared with the shNC group was analyzed by two‐way ANOVA followed by Šídák's multiple comparisons test. (P, Q) LM2‐4175 cells stably expressing shNC or shLMF2 #4 were orthotopically implanted into the mammary fat pads of 6‐week‐old BALB/c female nude mice (*n* = 6). Drug treatment began 24 days after implantation, with daily oral gavage of capecitabine (Cape; 250 mg/kg) or vehicle for 12 consecutive doses. Tumor volume (P) and weight (Q) are shown. The representative images of removed xenograft tumors are shown in Figure S2C. Data are presented as mean ± SD (*n* = 6). Statistical significance for tumor volume in (P) was determined by two‐way ANOVA followed by Tukey's multiple comparisons test (significant differences at Day 36 are shown) , and ordinary one‐way ANOVA followed by Tukey's multiple comparisons test for tumor weight in (Q). Notes: *, **, and *** indicate statistical significance at *p* < 0.05, *p* < 0.01, and *p* < 0.001 levels, respectively.

CCK‐8 and colony formation assays revealed that ectopic expression of LMF2 significantly enhanced the proliferative and clonogenic abilities of MDA‐231 and Hs578T cells (Figures 2C,D and S2B), whereas knockdown of LMF2 reduced the proliferative and colony formation capacity in LM2‐4175 and BT549 cells (Figures 2E,F and S2C). Furthermore, xenograft tumor assays in mice showed that knockdown of LMF2 suppressed tumor growth, leading to a significant reduction in both tumor volume and weight (Figures 2G,H and S2D). These results collectively suggest that LMF2 promotes TNBC cell proliferation in vitro and accelerates xenograft tumor growth in vivo.

Given the highly aggressive and metastatic nature of TNBC cells [1, 4], we next examined whether LMF2 affects the invasive and metastatic capabilities of TNBC cells. Transwell migration and invasion assays showed that overexpression of LMF2 significantly enhanced the migratory and invasive capabilities of MDA‐231 and Hs578T cells (Figures 2I,J and S2E), whereas knockdown of LMF2 reduced the migratory and invasive abilities of LM2‐4175 and BT549 cells (Figures 2K,L and S2F). Experimental lung metastasis assays via tail vein injection of cancer cells into mice showed that knockdown of LMF2 reduced the ability of LM2‐4175 cells to form lung metastases (Figures 2M and S2G,H). Collectively, these results suggest that LMF2 acts as a critical regulator of the migratory, invasive, and metastatic progression of TNBC cells.

Given that the TNBC cell lines used above are of the mesenchymal‐like subtype, we next evaluated the phenotypic effects of LMF2 depletion in HCC1806 cells, a well‐established representative model of the basal‐like 2 (BL2) subtype of TNBC [56]. As expected, a series of functional assays revealed that LMF2 knockdown in HCC1806 cells (Figure S3A) significantly reduced colony‐forming ability (Figure S3B,C) and impaired migratory and invasive capacities (Figure S3D–F). Taken together, these results suggest that LMF2 drives the malignant progression of TNBC cells both in vitro and in vivo.

### 3.3 LMF2 Confers Resistance of TNBC Cells to Capecitabine both In Vitro and in Mouse Models

As chemotherapy remains the cornerstone of systemic treatment for TNBC [4], we next investigated whether LMF2 influences chemosensitivity in this malignancy. Analysis of the GDSC dataset, the largest public resource for therapeutic biomarker discovery in cancer cells [42], revealed that high LMF2 expression was associated with reduced sensitivity to multiple chemotherapeutic agents. The strongest negative correlation was observed for 5‐fluorouracil (5‐FU) (Figure S4A).

Capecitabine is a prodrug of 5‐FU that is enzymatically converted into 5‐FU in vivo to exert its antitumor effects [9]. However, compared to 5‐FU, capecitabine offers several advantages in the clinical treatment of TNBC, including convenient oral administration, enhanced tumor targeting, reduced side effects, and therapeutic efficacy even in patients resistant to other chemotherapeutic agents [5, 6, 7, 8]. These attributes have established it as a preferred alternative to 5‐FU. In light of these facts, we next investigated the effects of LMF2 on the sensitivity of TNBC cells to capecitabine. CCK‐8 and colony formation assays demonstrated that knockdown of LMF2 enhanced the sensitivity of LM2‐4175 and BT549 cells to capecitabine (Figures 2N–O and S4B). Similar results were obtained in HCC1806 cells (Figure S4C).

These in vitro findings were further validated in xenograft tumor mouse models. The results showed that capecitabine administration led to more substantial suppression of tumor growth in mice implanted with LMF2‐depleted tumors relative to controls (Figures 2P,Q and S4D). Collectively, these results suggest that LMF2 promotes capecitabine resistance in TNBC, thus positioning it as a promising therapeutic target.

We next examined whether the altered drug sensitivity upon LMF2 depletion is mediated through modulating capecitabine‐induced DNA damage or apoptosis [57]. Immunoblotting assays revealed that in capecitabine‐treated control cells (shNC), the DNA damage marker γ‐H2AX (phospho‐histone H2AX at Ser139) [58] peaked at early time points and gradually declined by 12–24 h, indicating efficient DNA repair. By contrast, LMF2‐depleted cells exhibited persistently elevated γ‐H2AX signals throughout the 24‐h period following capecitabine treatment, suggesting that LMF2 deficiency impairs the resolution of capecitabine‐induced DNA lesions and converts transient genotoxic stress into sustained, unrepairable damage (Figure S5A). Consistently, in control cells treated with capecitabine, cleaved caspase‐3 (Asp175), a well‐established apoptosis marker [59], transiently increased at 12 h and subsequently declined by 24 h, indicating successful cellular adaptation and survival. In contrast, shLMF2‐expressing cells exhibited not only an earlier onset of cleaved caspase‐3 elevation but also a sustained, progressive increase that peaked at 24 h, suggesting the presence of an unabated apoptotic cascade (Figure S5A). Flow cytometry with Annexin V‐FITC/PI double staining further confirmed that LMF2 knockdown significantly enhanced capecitabine‐induced apoptosis in both cell lines (Figure S5B,C). Collectively, these findings indicate that LMF2 confers capecitabine chemoresistance through the modulation of DNA damage repair and apoptotic pathways.

### 3.4 VCP Stabilizes LMF2 by Inhibiting its Autophagic Degradation in TNBC Cells

To identify upstream regulators of LMF2, we screened for its interacting proteins by IP coupled with LC‐MS/MS assays. This screening was performed in HEK293T cells stably expressing either an empty vector or Flag‐LMF2 (Figure S6A). This approach identified 98 candidate proteins that specifically interact with LMF2 (Figure S6B). These candidates contain at least two spectral counts from uniquely matching peptides (unique spectral counts). The top ten candidate LMF2‐interacting proteins, ranked by the number of unique spectral counts identified by LC‐MS/MS, are shown in Figure 3A. Gene Ontology cellular component (GO‐CC) enrichment analysis showed that these proteins are primarily localized to the ER membrane and lumen (Figure S6C). Furthermore, Kyoto Encyclopedia of Genes and Genomes (KEGG) pathway analysis revealed significant enrichment of these candidate proteins in pathways related to protein processing in the ER (Figure S6D).

**FIGURE 3:**
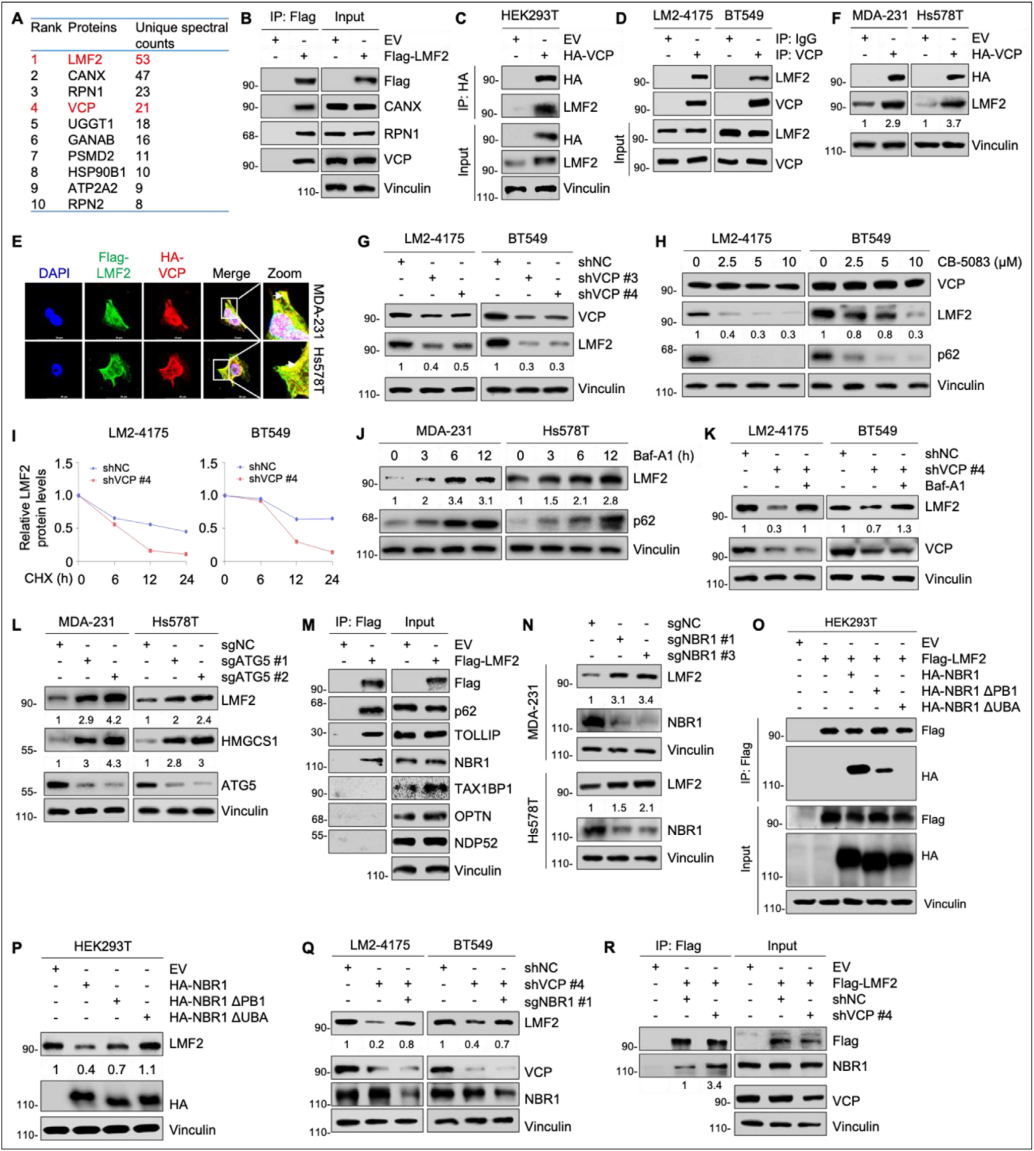
VCP stabilizes LMF2 by preventing its autophagic degradation mediated by NBR1. (A) The top 10 candidate LMF2‐interacting proteins, ranked by the number of unique spectral counts identified by LC‐MS/MS. (B, C) HEK293T cells were transfected with empty vector (EV), Flag‐LMF2 (B), or HA‐VCP (C) for 48 h and then subjected to sequential IP and immunoblotting assays with the indicated antibodies. (D) Cellular lysates from LM2‐4175 and BT549 cells were subjected to IP assays with control IgG or anti‐VCP antibody to detect the endogenous interaction between LMF2 and VCP.(E) MDA‐231 and Hs578T cells stably expressing Flag‐LMF2 and HA‐VCP were subjected to immunofluorescent staining with anti‐Flag (green) and anti‐HA (red) antibodies. DNA was counterstained with DAPI (blue). Colocalization between Flag‐LMF2 and HA‐VCP is indicated by the yellow signals (scale bar: 50 µm). (F, G) MDA‐231 and Hs578T cells stably expressing EV or HA‐VCP (F) and LM2‐4175 and BT549 cells stably expressing shVCP (#3 and #4) (G) were subjected to immunoblotting assays with the indicated antibodies. (H) LM2‐4175 and BT549 cells were treated with increasing concentrations of VCP inhibitor CB‐5083 for 8 h, followed by immunoblotting assays with the indicated antibodies. (I) LM2‐4175 and BT549 cells stably expressing shNC or shVCP #4 were treated with 100 µg/mL CHX for the indicated times, followed by immunoblotting assays with the indicated antibodies. The representative images of immunoblots are shown in Figure S4E, and the corresponding quantitative results are shown in I. (J) MDA‐231 and Hs578T cells were treated with 200 nM Baf‐A1 for the indicated times and subjected to immunoblotting assays with the indicated antibodies. (K) LM2‐4175 and BT549 cells stably expressing shVCP #4 were treated with 200 nM Baf‐A1 for 6 h and subjected to immunoblotting assays with the indicated antibodies. (L) MDA‐231 and Hs578T cells stably expressing sgATG5 (#1 and #2) were subjected to immunoblotting assays with the indicated antibodies. (M) HEK293T cells were transfected with EV or Flag‐LMF2 for 48 h and subjected to sequential IP and immunoblotting assays to examine the interaction between Flag‐LMF2 and the autophagy receptors. (N) Immunoblotting analysis of LMF2 protein levels in MDA‐231 and Hs578T cells stably expressing sgNBR1 (#1 and #3). (O) HEK293T cells were transfected with the indicated plasmids for 48 h and subjected to sequential IP and immunoblotting assays to examine the interaction between Flag‐LMF2 and HA‐NBR1 or its deletion mutants (ΔPB1 and ΔUBA). (P) HEK293T cells were transfected with HA‐NBR1 or its deletion mutants (ΔPB1 or ΔUBA) for 48 h and then subjected to immunoblotting assays using the indicated antibodies. (Q) LM2‐4175 and BT549 cells expressing shNC, shVCP #4 alone or in combination with sgNBR1 #1 were subjected to immunoblotting assays with the indicated antibodies. (R) HEK293T cells were transfected with EV, Flag‐LMF2, shNC, or shVCP #4 for 48 h and then subjected to sequential IP and immunoblotting assays with the indicated antibodies.

As shown in Figure 3B, IP combined with immunoblotting assays further demonstrated the interactions between LMF2 and its top three candidate binding partners, including calnexin (CANX), ribophorin I (RPN1), and valosin‐containing protein (VCP). Notably, all three are ER‐associated proteins. Moreover, the interaction between LMF2 and CANX has been previously documented in a large‐scale protein interactome screening study [60], which further underscores the reliability of our screening results. Further analysis of the FUSCC‐TNBC proteomic dataset [12] showed that all three proteins were highly expressed in TNBC tissues (Figure S6E–G) and exhibited positive correlations with LMF2 expression levels in these samples (Figure S6H–J).

Among the three LMF2‐interacting proteins, we selected VCP for further investigation in this study given its well‐established roles in maintaining protein homeostasis via regulating both proteasomal and autophagic degradation pathways, as well as its potential as an emerging therapeutic target in human cancers [34, 35, 36]. Further IP assays showed that ectopically expressed VCP as well as endogenous VCP interacted with endogenous LMF2 (Figure 3C,D). Unfortunately, we cannot perform reverse co‐IP assays to examine the interaction of endogenous LMF2 with endogenous VCP, as no commercial LMF2 antibody is currently available for IP assays. Immunofluorescent staining further revealed partial co‐localization of LMF2 with VCP in both MDA‐231 and Hs578T cells (Figure 3E, the yellow area indicated by the white arrows in the zoomed‐in images). These results suggest that LMF2 is a novel binding partner of VCP.

Given the pivotal role of VCP in maintaining protein homeostasis [34, 35, 36], we next explored the mutual regulatory relationship between VCP and LMF2. As shown in Figure 3F, ectopic expression of VCP in MDA‐231 and Hs578T cells enhanced LMF2 protein levels. Conversely, knockdown of VCP by two independent shRNAs or pharmacological inhibition of VCP by its selective inhibitor CB‐5083 [35, 51] led to a decrease in LMF2 protein levels in LM2‐4175 and BT549 cells (Figure 3G,H). As p62 is a validated substrate of VCP [35, 37], it was used as a positive control to monitor the efficacy of the VCP inhibitor CB‐5083 (Figure 3H). Consistently, CB‑5083 reduced both endogenous and ectopically expressed LMF2 protein levels in MDA‐231 cells (Figure S7A). In contrast, silencing LMF2 did not significantly alter VCP protein levels (Figure S7B). Collectively, these findings position VCP as an upstream regulator of LMF2, without evidence of feedback from LMF2 on VCP.

Notably, LMF2 mRNA levels were not significantly affected by either ectopic expression or genetic/pharmacological inhibition of VCP (Figure S7C–E), indicating that VCP regulates LMF2 at the post‐transcriptional level. Thus, we next performed CHX chase assays [46] to evaluate the effects of VCP on LMF2 protein turnover. The results showed that knockdown of VCP markedly accelerated LMF2 degradation and shortened its protein half‐life, indicating that VCP enhances the protein stability of LMF2 (Figures 3I and S7F).

In mammalian cells, protein stability is primarily governed by two major pathways, including the ubiquitin‐proteasome system and the autophagy‐lysosome pathway [61]. We first evaluated the impact of proteasome inhibition on LMF2 stability by treating MDA‐231 and BT‐549 cells with the proteasome inhibitor MG‐132, and found no significant change in LMF2 protein levels following MG‐132 treatment (Figure S7G). In contrast, inhibition of lysosomal function using either bafilomycin A1 (Baf‐A1) or ammonium chloride (NH_4_Cl) led to a pronounced accumulation of LMF2 protein levels (Figures 3J and S7H). Conversely, treatment with the autophagy inducer rapamycin (Rapa) reduced LMF2 protein expression levels (Figure S7I). Furthermore, the reduced LMF2 protein levels caused by knockdown of VCP in LM2‐4175 and BT‐549 cells was partially reversed following Baf‐A1 treatment (Figure 3K). Together, these results suggest that VCP stabilizes LMF2 primarily by suppressing its autophagy‐lysosomal degradation.

### 3.5 VCP Enhances LMF2 Stability by Blocking its NBR1‐Mediated Autophagic Degradation

In mammalian cells, autophagy is mechanistically categorized into three main types, including macroautophagy, microautophagy, and chaperone‐mediated autophagy (CMA) [30]. To examine whether LMF2 is degraded through CMA, we knocked down heat shock protein family A member 8 (HSPA8) and lysosomal associated membrane protein 2A (LAMP2A), two core components of the CMA machinery [62], and found that LMF2 protein levels remained largely unchanged following depletion of each individual gene (Figure S8A,B). This result suggests that CMA is not involved in LMF2 degradation. In contrast, treatment of cells with 3‐methyladenine (3‐MA), a selective inhibitor of macroautophagy [63], resulted in a remarkable accumulation of LMF2 (Figure S8C). Furthermore, knockdown of autophagy‐related 5 (ATG5), an essential gene for mammalian macroautophagy [64], also increased LMF2 protein levels in MDA‐231 and Hs578T cells (Figure 3L). These results collectively suggest that LMF2 is predominantly degraded through the macroautophagy pathway.

Although macroautophagy was initially described as a non‐selective process for nutrient recycling, growing evidence shows that it can also mediate the selective degradation of specific substrates through autophagy receptor proteins [32, 33]. To date, six major autophagy receptors have been identified, including p62, NBR1, NDP52, OPTN, TOLLIP, and TAX1BP1 [32, 33]. These receptors recognize specific cargo proteins and simultaneously bind to autophagosomal membranes via their LC3‐interacting region (LIR) motifs, thereby facilitating the delivery and subsequent degradation of cargos within the autophagosome [32, 33]. To determine which autophagy receptor protein mediates LMF2 degradation in TNBC cells, we first screened autophagy receptors that bind to LMF2 by IP assays. The results revealed that LMF2 selectively interacted with NBR1, p62, and TOLLIP, but not OPTN, NDP52, and TAX1BP1 (Figure 3M). Then, we individually knocked down NBR1, p62, and TOLLIP in MDA‐231 and Hs578T cells, and found that depletion of NBR1 resulted in a substantial increase in LMF2 protein levels (Figure 3N). In contrast, knockdown of either p62 or TOLLIP did not remarkably affect LMF2 protein levels (Figure S8D,E). These results indicate that NBR1 is the primary autophagy receptor responsible for LMF2 degradation.

NBR1 is an archetypal selective autophagy receptor, which contains an N‐terminal Phox and Bem1p (PB1) domain (residues 4‐85) and a C‐terminal ubiquitin‐associated (UBA) domain (residues 913‐957) (Figure S8F) [65, 66]. The PB1 domain is involved in interactions with other PB1 domain‐containing proteins (notably p62), while the UBA domain binds to ubiquitin and ubiquitinated cargos [65, 66]. Indeed, we found that LMF2 is a poly‐ubiquitinated protein (Figure S8G, compare lane 3 with 2). Unexpectedly, we found that the E3 ubiquitin‐protein ligase TRIM21, a potential binding partner of LMF2 identified by LC‐MS/MS assays above, was not responsible for mediating LMF2 ubiquitination (Figure S8G, compare lane 4 with 3). Moreover, NBR1 lacking its UBA domain failed to interact with LMF2 (Figure 3O) and was unable to downregulate LMF2 protein levels (Figure 3P). Furthermore, knockdown of VCP in LM2‐4175 and BT549 cells reduced LMF2 protein expression levels, and this effect was partially reversed by simultaneous knockdown of NBR1 in these cells (Figure 3Q). These results suggest that NBR1 is involved in VCP‐mediated stabilization of LMF2.

To gain deeper mechanistic insight into the above findings, we next investigated whether VCP regulates the association between LMF2 and NBR1. Notably, knockdown of VCP substantially potentiated their interaction (Figure 3R). We next examined whether VCP‐mediated regulation of the LMF2‐NBR1 interaction depends on its ATPase activity. As shown in Figure S9A, both wild‑type HA‑VCP and the ATPase‑deficient mutant E578Q [67, 68, 69] retained comparable binding to Flag‑LMF2. However, expression of the E578Q mutant led to a marked increase in the interaction between Flag‑LMF2 and endogenous NBR1. This result suggests that the ATPase activity of VCP is critical for disassembling or restraining the LMF2–NBR1 complex.

To delineate the LMF2 region responsible for VCP/NBR1 binding, we rationally divided LMF2 into two major fragments based on the structural annotation from UniProt (https://www.uniprot.org/uniprotkb/Q9BU23/entry) and the transmembrane topology illustrated in Figure 1K. The N‐terminal truncated fragment (NT, aa 1–350) encompasses the first seven transmembrane domains (TM1–TM7) along with their intervening cytoplasmic loops, which constitute the primary cytoplasmic interface. In contrast, the C‐terminal truncated fragment (CT, aa 351–707) contains the remaining three transmembrane domains (TM8–TM10) (Figure S9B). As shown in Figure S9C–F, the LMF2 NT, but not its CT, is required and sufficient for binding to VCP and NBR1. These results suggest that VCP and NBR1 may competitively bind to a shared region within the LMF2 NT (Figure S9G). In addition, we observed that the LMF2 NT (residues 1–350) migrated at a higher apparent molecular weight than the CT (residues 351–707) on SDS‐PAGE, despite its smaller theoretical mass. ProtParam analysis revealed that NT is highly hydrophobic and leucine‐rich (18.4%), whereas CT is hydrophilic and proline‐rich (7.5%). Hydrophobic NT likely binds more SDS or retains an extended conformation, slowing its gel mobility, while proline‐induced bends in CT favor a compact form that migrates faster. This gel‐shifting behavior is well documented for hydrophobic domains.

Collectively, these results demonstrate that VCP suppresses the autophagic degradation of LMF2 by disrupting its interaction with the autophagy receptor NBR1.

### 3.6 Pharmacological Inhibition of VCP Effectively Mitigates LMF2‐Driven Cancer Progression and Cape Resistance in TNBC

Given that VCP functions as an upstream regulator of LMF2 (Figure 3), we next assessed whether the potent and selective VCP inhibitor CB‑5083 [51] modulates LMF2‑driven TNBC progression and capecitabine resistance. Of note, CB‐5083 has been well documented to exert broad antitumor activity across both hematologic and solid tumor models [35, 70]. Furthermore, we have recently demonstrated that CB‐5083 effectively suppresses tumor growth and lung metastasis in mice bearing MDA‐MB‐231 cells stably expressing the empty vector pLVX [71].

Xenograft tumor assays in mice demonstrated that the enhanced xenograft tumor growth and lung metastatic potential induced by ectopic expression of LMF2 were partially impaired upon treatment with the VCP inhibitor CB‐5083 (Figure 4A–F). Furthermore, administration of CB‐5083 enhanced the sensitivity of MDA‐231 cells stably expressing LMF2 to capecitabine (Figure 4G–I). Collectively, these results suggest that pharmacological inhibition of VCP effectively counteracts LMF2‐driven cancer progression and capecitabine resistance in TNBC.

**FIGURE 4:**
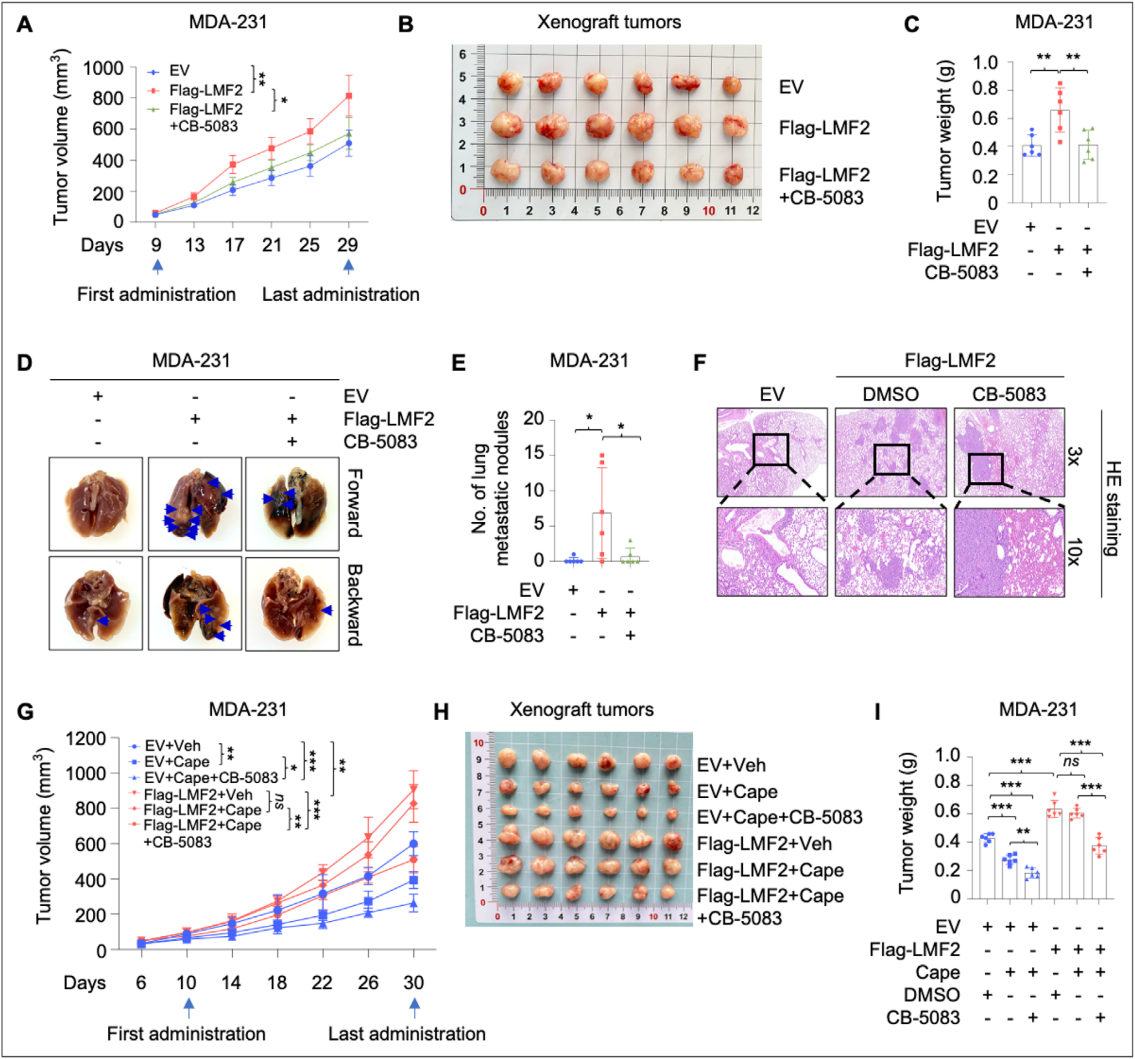
Pharmacological inhibition of VCP effectively mitigates LMF2‐driven cancer progression and capecitabine resistance in TNBC. (A–C) MDA‐231 cells stably expressing EV or Flag‐LMF2 were inoculated into mammary fat pads of 6‐week‐old BALB/c female nude mice. After 9 days of injection, mice inoculated with Flag‐LMF2‐expressing cells were randomly divided into two groups (*n* = 6 per group) and were administered with or without CB‐5083 (50 mg/kg) by oral gavage once daily for 4 weeks. Tumor volume (A), representative images of removed xenograft tumors (B), and tumor weights (C) are shown. Data are presented as mean ± SD (*n* = 6). Statistical significance was determined by two‐way ANOVA followed by Šídák's multiple comparisons test (A), and two‐tailed unpaired Student's *t*‐test for pairwise comparisons between indicated groups (C). (D–F) MDA‐231 cells stably expressing EV or Flag‐LMF2 were injected into the tail vein of 7‐week‐old female BALB/c nude mice. One week after injection, mice inoculated with Flag‐LMF2‐expressing cells were randomly divided into two groups (*n* = 6 per group) and were administered with or without CB‐5083 (50 mg/kg) by oral gavage once daily for 4 weeks. After 4 weeks of treatment, mice were euthanized, and lungs were collected. The representative images (D) and quantitative results (E) of metastatic lung nodules (D), and HE‐stained lung sections (F; scale bars, 500 µm for 3× images and 100 µm for 10× magnified images) are shown. Data are presented as mean ± SD (*n* = 6). Statistical significance was determined by a two‐tailed Mann‐Whitney test for pairwise comparisons between indicated groups (E). (G–I) MDA‐231 cells stably expressing EV or Flag‐LMF2 were inoculated into the mammary fat pad of 6‐week‐old BALB/c female nude mice (*n* = 18 per group). After 10 days of injection, mice in each group were randomly divided into three subgroups (*n* = 6 per subgroup) and administered with vehicle, capecitabine (Cape; 250 mg/kg) alone, or in combination with CB‐5083 (50 mg/kg) by oral gavage once daily for 4 weeks. Tumor volume (G), representative images of removed xenograft tumors (H), and tumor weights (I) are shown. Data are presented as mean ± SD (*n* = 6). Statistical significance for tumor volume in (G) was determined by two‐way ANOVA followed by Tukey's multiple comparisons test (significant differences at Day 30 are shown), and ordinary one‐way ANOVA followed by Šídák's multiple comparisons test for tumor weights in (I). Notes: *, **, and *** indicate statistical significance at *p* < 0.05, *p* < 0.01, and *p* < 0.001 levels, respectively.

### 3.7 LMF2 Enhances HMGCS1 Protein Stability and Boosts Cholesterol Biosynthesis

To identify the downstream effectors of LMF2 in TNBC cells, we performed label‐free quantitative proteomic assays using cellular lysates of LM2‐4175 cells stably expressing two independent shRNAs targeting LMF2 (Figure S10A). Based on a fold‐change threshold of >1.5 for differential protein expression, we identified 178 downregulated and 172 upregulated proteins following LMF2 knockdown (Figure S10B). The top ten most significantly downregulated proteins are presented in Figure S10C. To validate the above findings, we first analyzed the correlation of LMF2 expression levels with the three most significantly downregulated proteins, including malic enzyme 1 (ME1), HMGCS1, and integrin subunit beta 6 (ITGB6), in the FUSCC‐TNBC proteomic dataset [12]. Among these candidates, only HMGCS1 showed a significant positive correlation with LMF2 expression levels (Figure S10D–F). Thus, we selected HMGCS1 for further investigation.

Consistent with these findings, ectopic expression of LMF2 in MDA‐231 and Hs578T cells increased HMGCS1 protein levels, whereas knockdown of LMF2 in LM2‐4175 and BT549 cells decreased HMGCS1 expression levels (Figure 5A,B). By contrast, RT‐qPCR assays showed no significant changes in HMGCS1 mRNA levels upon either overexpression or knockdown of LMF2 (Figure S10G,H). These findings suggest that LMF2 regulates HMGCS1 expression via post‐transcriptional mechanisms.

**FIGURE 5:**
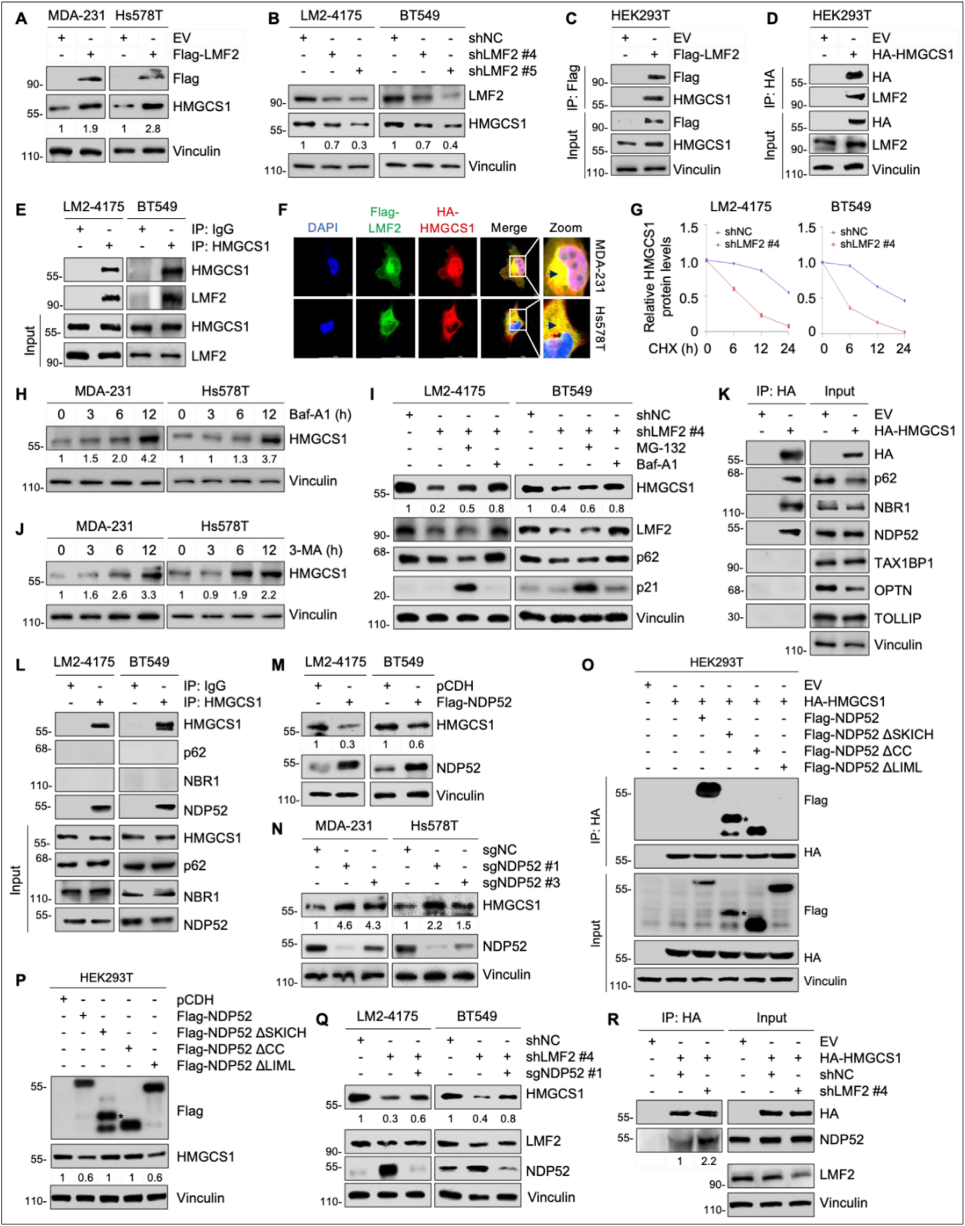
LMF2 stabilizes HMGCS1 by counteracting its NDP52‐mediated autophagic degradation. (A, B) MDA‐231 and Hs578T cells stably expressing EV or Flag‐LMF2 (A) and LM2‐4175 and BT549 cells stably expressing shLMF2 (#4 and #5) (B) were subjected to immunoblotting assays to detect HMGCS1 protein levels. (C, D) HEK293T cells were transfected with the indicated plasmids for 48 h and subjected to sequential IP and immunoblotting assays to examine the interaction between Flag‐LMF2 and HMGCS1 (C), and between HA‐HMGCS1 and LMF2 (D).(E) LM2‐4175 and BT549 cells were subjected to IP assasys with IgG or anti‐HMGCS1 antibody to detect the endogenous interaction between LMF2 and HMGCS1.(F) MDA‐231 and Hs578T cells stably expressing Flag‐LMF2 and HA‐HMGCS1 were subjected to immunofluorescent staining with anti‐Flag (green) and anti‐HA (red) antibodies. DNA was counterstained with DAPI (blue). Colocalization between Flag‐LMF2 and HA‐HMGCS1 is indicated by the yellow signals (scale bars: 5 µm for MDA‐231 and 50 µm for Hs578T cells). (G) LM2‐4175 and BT549 cells expressing shNC or shLMF2 #4 were treated with 100 µg/mL CHX for the indicated times, followed by immunoblotting assays. The quantitative results of relative HMGCS1 protein levels are shown. The corresponding immunoblots are shown in Figure S6I. (H) MDA‐231 and Hs578T cells were treated with 200 nM Baf‐A1 for the indicated times and subjected to immunoblotting assays. (I) LM2‐4175 and BT549 cells stably expressing shLMF2 (#4) were treated with 10 µM MG‐132 or 200 nm Baf‐A1 for 6 h and subjected to immunoblotting assays. (J) MDA‐231 and Hs578T cells were treated with 1 mM 3‐MA for the indicated times and subjected to immunoblotting assays to examine HMGCS1 expression levels. (K) HEK293T cells were transfected with EV or HA‐HMGCS1 for 48 h and then subjected to sequential IP and immunoblotting assays to assess the interaction between HA‐HMGCS1 and the indicated autophagy receptors. (L) LM2‐4175 and BT549 cells were subjected to IP assays with IgG or anti‐HMGCS1 antibody, followed by immunoblotting assays with the indicated antibodies. (M, N) LM2‐4175 and BT549 cells stably expressing Flag‐NDP52 (M) and MDA‐231 and Hs578T cells stably expressing sgNDP52 (#1 and #3) (N) were subjected to immunoblotting assays to examine HMGCS1 expression levels. (O) HEK293T cells were transfected with the indicated plasmids for 48 h and subjected to sequential IP and immunoblotting assays to examine the interaction between HA‐HMGCS1 and Flag‐NDP52 or its deletion mutants (ΔSKICH, ΔCC, or ΔLIML). The band corresponding to the ΔSKICH mutant is indicated by an asterisk (*). (P) HEK293T cells were transfected with Flag‐NDP52 or its deletion mutants (ΔSKICH, ΔCC, or ΔLIML) for 48 h and then subjected to immunoblotting assays to examine HMGCS1 protein levels. The band corresponding to the ΔSKICH mutant is indicated by an asterisk (*). (Q) LM2‐4175 and BT549 cells expressing shNC, shLMF2 #4 alone or in combination with sgNDP52 #1 were subjected to immunoblotting assays. (R) HEK293T cells were transfected with the indicated expression vectors for 48 h and then subjected to sequential IP and immunoblotting assays with the indicated antibodies.

Following these observations, we next examined whether LMF2 interacts with HMGCS1. Reciprocal IP assays demonstrated that ectopically expressed Flag‐LMF2 bound to endogenous HMGCS1 (Figure 5C), and conversely, HA‐HMGCS1 interacted with endogenous LMF2 (Figure 5D). This observed interaction between those two proteins was further confirmed by endogenous IP assays using an anti‐HMGCS1 antibody in both LM2‐4175 and BT549 cell lines (Figure 5E). As mentioned earlier, reverse IP assays could not be performed to investigate the interaction between endogenous LMF2 and HMGCS1 due to the lack of a commercially available LMF2 antibody suitable for IP assays. Additionally, immunofluorescent staining showed partial co‐localization of Flag‐LMF2 and HA‐HMGCS1 (Figure 5F, the yellow area indicated by the dark blue arrows in the zoomed‐in images).

We next investigated whether LMF2 regulates HMGCS1 protein stability. CHX chase assays revealed that silencing LMF2 accelerated HMGCS1 degradation and shortened its half‐life (Figures 5G and S10I). As a functional readout of the well‐established role of HMGCS1 in cholesterol biosynthesis [23, 24], we further demonstrated that overexpression of LMF2 enhanced, whereas depletion of LMF2 reduced, relative cholesterol levels in the culture supernatant (Figure S10J,K). Consistent with these results, LMF2 knockdown significantly reduced relative cholesterol levels, and this decrease was effectively rescued by co‑overexpression of HA‑HMGCS1 in both cell lines (Figure S10L). Together, these results suggest that LMF2 enhances the stability of the HMGCS1 protein and promotes cholesterol biosynthesis.

Given that LMF1, a paralog of LMF2, has been shown to promote lipoprotein lipase (LPL) activity [13, 14], we also evaluated LPL activity in LM2‐4175 and BT549 cells following LMF2 knockdown. The results showed that LMF2 depletion significantly reduced LPL activity in both cell lines compared to controls (Figure S10M). These findings indicate that LMF2 is required for maintaining LPL activity in these cells, and that the noted oncogenic functions of LMF2 in TNBC may be at least partially attributed to its regulation of LPL‐mediated lipid metabolism.

### 3.8 LMF2 Stabilizes HMGCS1 by Counteracting its NDP52‐Mediated Autophagic Degradation

To address the underlying mechanism by which LMF2 stabilizes HMGCS1, we first examined the degradation pathway of HMGCS1 in TNBC cells. To achieve this, we treated LM2‐4175 and BT549 cells with the proteasome inhibitor MG‐132, the autophagy inhibitors Baf‐A1 or NH_4_Cl, or the autophagy inducer rapamycin to assess their effects on HMGCS1 protein levels. While HMGCS1 has been shown to undergo ubiquitin‐dependent proteasomal degradation in other model systems [24, 29], we observed, however, only a modest upregulation of HMGCS1 protein following MG‐132 treatment in TNBC cells (Figure S11A). In contrast, lysosomal inhibition with either Baf‐A1 or NH_4_Cl substantially increased HMGCS1 protein levels (Figures 5H and S11B), whereas autophagy induction with rapamycin (Rapa) decreased them (Figure S11C). Moreover, Baf‐A1 was more effective than MG‐132 in rescuing the decreased HMGCS1 protein levels caused by LMF2 knockdown in LM2‐4175 and BT549 cells (Figure 5I). These results collectively suggest that HMGCS1 is primarily degraded via the autophagy‐lysosome pathway in TNBC cells.

To identify the specific autophagy pathways responsible for HMGCS1 degradation, we first knocked down two key components of the CMA machinery, HSPA8 and LAMP2A [62]. However, we found that depletion of neither gene significantly affected HMGCS1 protein levels (Figure S11D,E), indicating that CMA is not involved in HMGCS1 degradation in TNBC cells. In contrast, treatment of MDA‐231 and Hs578T cells with the selective macroautophagy inhibitor 3‐MA [63] increased HMGCS1 protein levels (Figure 5J). Consistent with this result, knockdown of ATG5, a key gene essential for macroautophagy in mammalian cells [64], also elevated HMGCS1 levels in both cell lines (Figure 3L). These findings indicate that macroautophagy serves as the major pathway responsible for HMGCS1 degradation in TNBC cells.

Based on these findings, we next performed IP assays to assess the interaction between HMGCS1 and six major autophagy receptors [32, 33]. The results showed that ectopically expressed HA‐HMGCS1 interacted with the autophagy receptors p62, NBR1, and NDP52 (Figure 5K). To further validate these interactions under physiological conditions, we conducted endogenous IP assays using an anti‐HMGCS1 antibody in LM2‐4175 and BT549 cells. Among those three receptors, only NDP52 was found to form a complex with endogenous HMGCS1, whereas no detectable interaction was observed with NBR1 or p62 (Figure 5L). These findings suggest that HMGCS1 specifically associates with NDP52 at endogenous expression levels.

Given that NDP52 mainly recognizes ubiquitinated substrates for autophagic degradation [72] and that HMGCS1 is a ubiquitinated protein [24, 29], we hypothesized that NDP52 may mediate the autophagic degradation of HMGCS1. To this test, we ectopically expressed NDP52 in LM2‐4175 and BT549 cells and observed a marked decrease in HMGCS1 protein levels (Figure 5M). Conversely, knockdown of NDP52 in MDA‐231 and Hs578T cells resulted in elevated HMGCS1 expression (Figure 5N). These findings confirm that NDP52 serves as the key autophagy receptor responsible for HMGCS1 degradation in TNBC cells.

Previous studies have shown that acetylation of HMGCS1 at lysine 273 (K273) by lysine acetyltransferase 5 enhances its protein stability [28], and that K273 is one of the predicted ubiquitination sites of HMGCS1 protein [29]. To determine whether K273 is required for NDP52‐mediated degradation of HMGCS1, we generated a HA‐tagged HMGCS1 mutant with a lysine‑to‑arginine substitution at residue 273 (K273R) and assessed its sensitivity to NDP52‑mediated degradation in HEK293T cells. As shown in Figure S11F, wild‑type HMGCS1 and the K273R mutant were comparably reduced upon Flag‑NDP52 co‑expression, indicating that the K273R mutation does not prevent NDP52‑induced HMGCS1 degradation. Thus, NDP52‑driven degradation of HMGCS1 proceeds independently of this residue.

Human NDP52 comprises an N‐terminal SKICH (skeletal muscle and kidney‐enriched inositol phosphatase carboxyl homology) domain (residues 1‐127), an intermediate coiled‐coil (CC) domain (residues 134‐350), and a C‐terminal LIM‐K (Lin11, Isl‐1, and Mec‐3‐like) domain (residues 395‐446) (Figure S11G) [72, 73, 74]. To determine which domain of NDP52 is responsible for recognizing and degrading HMGCS1, we constructed three individual domain deletion mutants, termed ΔSKICH, ΔCC, and ΔLIM‐L. IP assays showed that full‐length NDP52, as well as the ΔSKICH and ΔCC mutants, co‐immunoprecipitated with HA‐HMGCS1. In contrast, the ΔLIM‐L mutant failed to interact with HMGCS1 (Figure 5O). Notably, deletion of the SKICH or CC domain abolished the ability of NDP52 to downregulate HMGCS1 protein levels (Figure 5P). Together, these findings indicate that the LIM‐L domain mediates the recognition of HMGCS1 by NDP52, while the SKICH and CC domains are indispensable for its degradation. Consistent with our findings, NDP52 has been shown to use distinct domains to bind its substrates and facilitate their degradation [72, 73, 74, 75]. For instance, the SKICH domain of NDP52 is required for its interaction with the TLR adaptor molecule TIR‐domain‐containing adapter‐inducing interferon‐𝛽 (TRIF), while its LIM‐L domain mediates TRIF degradation [73]. Notably, although NDP52 lacking the SKICH domain (ΔSKICH) fails to bind TRIF, it still promotes TRIF degradation through a mechanism that remains to be elucidated [73]. In agreement with our data, the LIM‑L domain has been shown to mediate protein‑protein interactions [74, 76], as exemplified by its role in mediating the NDP52–MAVS interaction [74]. Given that NDP52 is a recently identified autophagy receptor [77], the exact mechanistic basis for the domain‑specific degradation of its substrates awaits further investigation.

Then, we investigated whether NDP52 is involved in the LMF2‐mediated regulation of HMGCS1. As shown in Figure 5Q, knockdown of LMF2 in LM2‐4175 and BT549 cells led to a decrease in HMGCS1 protein levels, which was partially reversed upon concurrent knockdown of NDP52. These results indicate that NDP52 is involved in LMF2‐dependent stabilization of HMGCS1 in TNBC cells. Moreover, we found that LMF2 knockdown enhanced the association between HMGCS1 and NDP52 (Figure 5R). In addition, we observed that LMF2 knockdown significantly elevated NDP52 protein levels in LM2‑4175 and BT549 cells (Figure 5Q) but did not produce a comparable effect in HEK293T cells (Figure 5R). We speculate that this discrepancy likely arises from the distinct genetic backgrounds and proteostatic landscapes between TNBC cells and HEK293T cells.

To investigate how LMF2 disrupts HMGCS1–NDP52 interaction, we next mapped the HMGCS1 region required for binding to both proteins. As shown in Figure S12A–F, both LMF2 and NDP52 associate with the C‐terminal domain of HMGCS1, raising the possibility of competitive binding at this region. Taken together, these results suggest that LMF2 stabilizes HMGCS1 by preventing its NDP52‐mediated autophagic degradation. LMF2 achieves this by disrupting the interaction between NDP52 and HMGCS1.

### 3.9 HMGCS1 Re‐Expression or Exogenous Cholesterol Supplementation Partially Rescues the Impaired Malignant and Chemoresistant Phenotypes Induced by LMF2 Depletion

To determine whether HMGCS1 mediates the noted oncogenic effects of LMF2 in TNBC, we performed rescue experiments by reintroducing HMGCS1 into LMF2‐depleted LM2‐4175 and BT549 cells (Figure 6A). CCK‐8 and colony formation assays demonstrated that re‐expression of HMGCS1 partially rescued the attenuated proliferation and clonogenicity of LMF2‐depleted LM2‐4175 and BT549 cells (Figure S13A–C). Moreover, Transwell assays showed that HMGCS1 re‐expression also partially restored the impaired migratory and invasive capabilities caused by LMF2 knockdown (Figure S13D–F).

**FIGURE 6:**
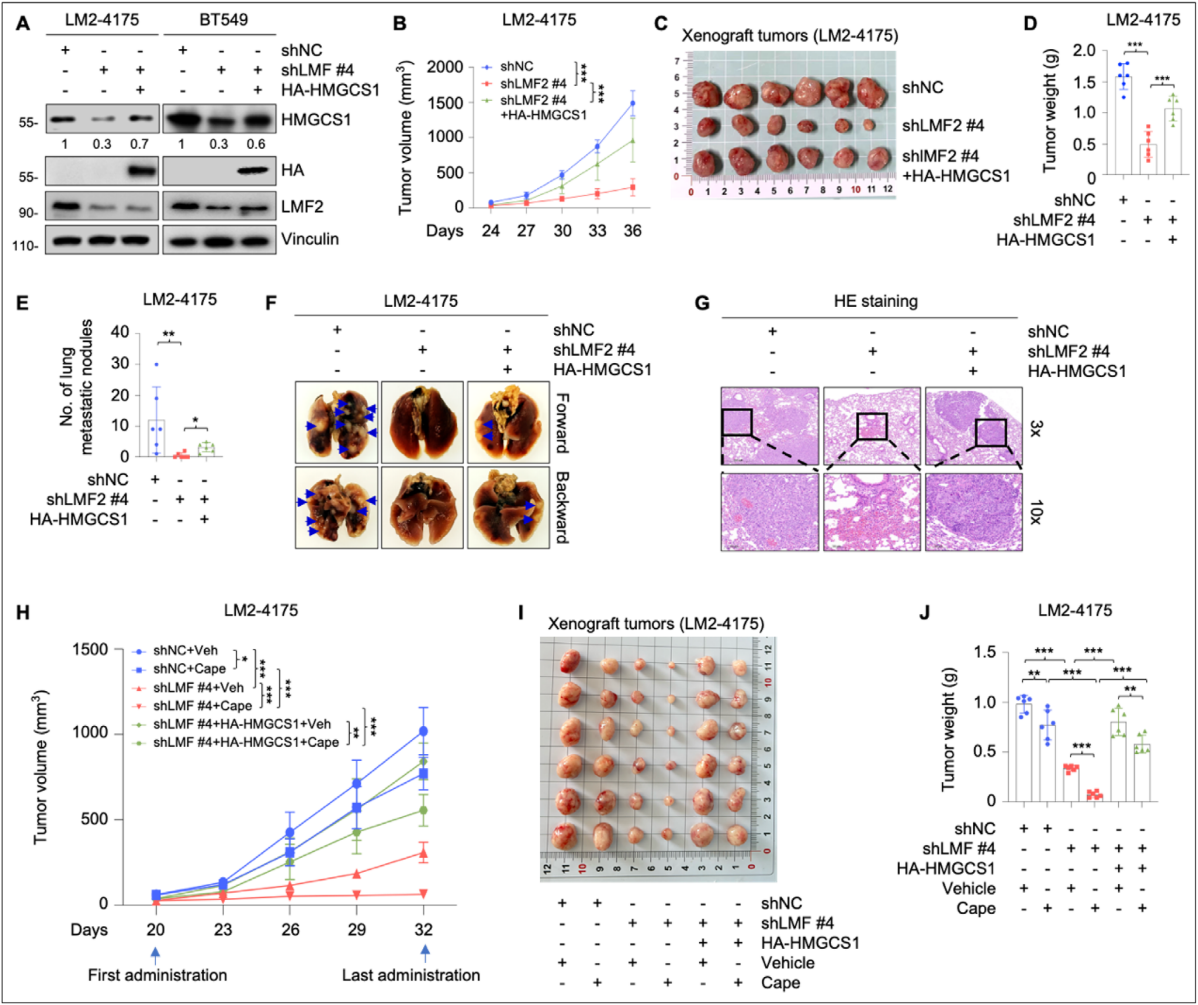
Re‐expression of HMGCS1 partially rescues the impaired malignant and chemoresistant phenotypes induced by LMF2 depletion in vivo. (A) LM2‐4175 and BT549 cells stably expressing shNC or shLMF2#4 were transfected with or without HA‐HMGCS1. After 48 h of transfection, cells were subjected to immunoblotting assays with the indicated antibodies. (B–D) LM2‐4175 cells expressing shNC, shLMF2 #4 alone or in combination with HA‐HMGCS1 were subcutaneously injected into 6‐week‐old BALB/c female nude mice (*n* = 6). Tumor volume (B), representative images of removed xenograft tumors (C), and tumor weights (D) are shown. Data are presented as mean ± SD (*n* = 6). Statistical significance for tumor volume in (B) was determined by two‐way ANOVA followed by Šídák's multiple comparisons test (significant differences at Day 36 are shown), and ordinary one‐way ANOVA followed by Šídák's multiple comparisons test for tumor weights in (D). (E–G) LM2‐4175 cells stably expressing shNC, shLMF2 #4 alone or in combination with HA‐HMGCS1 were injected into the tail vein of 7‐week‐old female BALB/c nude mice (*n* = 6). After 6 weeks of injection, mice were euthanized, lungs were harvested, and metastatic lung nodules were quantified. Quantitative results of metastatic lung nodules (E) and representative images of removed lung tissues (F) and HE‐stained sections of lung tissues (G; scale bars, 500 µm for 3× images and 100 µm for 10× magnified images) are shown. Data are presented as mean ± SD (*n* = 6). Statistical comparisons between indicated groups were analyzed by a two‐tailed Mann–Whitney U test. (H–J) LM2‐4175 cells stably expressing shNC, shLMF2 #4 alone or in combination with HA‐HMGCS1 were subcutaneously injected into 6‐week‐old female BALB/c nude mice. Twenty days after injection, each group was randomly divided into two subgroups (*n* = 6 per subgroup) and administered with vehicle or capecitabine (Cape, 250 mg/kg) by oral gavage once daily for 12 days. Mice were then euthanized, and tumors were excised and weighed. Tumor volume (H), representative images of xenograft tumors (I), and tumor weights (J) are shown. Data are presented as mean ± SD (*n* = 6). Statistical significance for tumor volume in (H) was determined by two‐way ANOVA with Tukey's multiple comparisons test (significant differences at Day 32 are shown), and ordinary one‐way ANOVA followed by Šídák's multiple comparisons test for tumor weights in (J). Notes: *, **, and *** indicate statistical significance at *p* < 0.05, *p* < 0.01, and *p* < 0.001 levels, respectively.

Given the well‐established role of HMGCS1 in cholesterol biosynthesis [23, 24] and our findings that LMF2 stabilizes HMGCS1 and enhances cholesterol production (Figures 5 and S10J–L), we next investigated whether supplementing exogenous cholesterol could rescue the impaired malignant phenotypes induced by LMF2 silencing. A series of in vitro functional assays demonstrated that the addition of exogenous soluble cholesterol to the culture medium partially restored the attenuated proliferative, migratory, and invasive capacities of LMF2‐depleted LM2‐4175 and BT549 cells (Figure S14A–F). These results further support the functional contribution of the HMGCS1‐cholesterol pathway to LMF2‐driven malignant phenotypes in TNBC cells.

To further validate these findings, we extended our investigation to in vivo mouse models. In orthotopic xenograft models, re‐expression of HMGCS1 in LMF2‐depleted cells significantly promoted tumor growth, as evidenced by increased tumor volume and weight (Figure 6B–D). Similarly, in experimental lung metastasis models established via tail vein injection, HMGCS1 restoration rescued the decrease in metastatic lung nodules resulting from LMF2 knockdown (Figure 6E,F). Consistent with these results, hematoxylin and eosin (HE) staining of mouse lung tissue sections revealed a higher metastatic burden upon HMGCS1 re‐expression, further supporting its role in promoting lung colonization (Figure 6G). Together, these findings suggest that HMGCS1 contributes to LMF2‐driven tumor growth and lung metastasis in TNBC.

Finally, to assess the contribution of HMGCS1 to LMF2‐mediated capecitabine resistance in TNBC in vivo, we performed mouse xenograft tumor assays. The results showed that knockdown of LMF2 increased the sensitivity of TNBC cells to capecitabine. This enhanced sensitivity was partially reversed upon re‐expression of HMGCS1 in LMF2‐depleted cells (Figure 6H–J). Collectively, these findings underscore the role of HMGCS1 in promoting LMF2‐driven chemoresistance in TNBC. In addition, LMF2 knockdown also sensitized TNBC cells to simvastatin (Figure S14G), a competitive inhibitor of HMG‐CoA reductase (HMGCR) [78], further supporting a functional link between this axis and the cholesterol biosynthesis pathway.

### 3.10 Clinical Relevance of the VCP‐LMF2‐HMGCS1 Axis in TNBC Specimens

To evaluate the clinical relevance of the proposed VCP‐LMF2‐HMGCS1 axis in TNBC, we next analyzed the expression levels of VCP, LMF2, and HMGCS1 in 14 paired TNBC samples and adjacent normal tissues using immunoblotting. The results demonstrated that all three proteins were significantly upregulated in tumor tissues compared to normal counterparts (Figure 7A–D). Correlation analysis further revealed a positive association of the expression levels between VCP and LMF2 (Figure 7E) as well as between LMF2 and HMGCS1 (Figure 7F). In addition, a significant correlation was also observed between VCP and HMGCS1 levels (Figure 7G).

**FIGURE 7:**
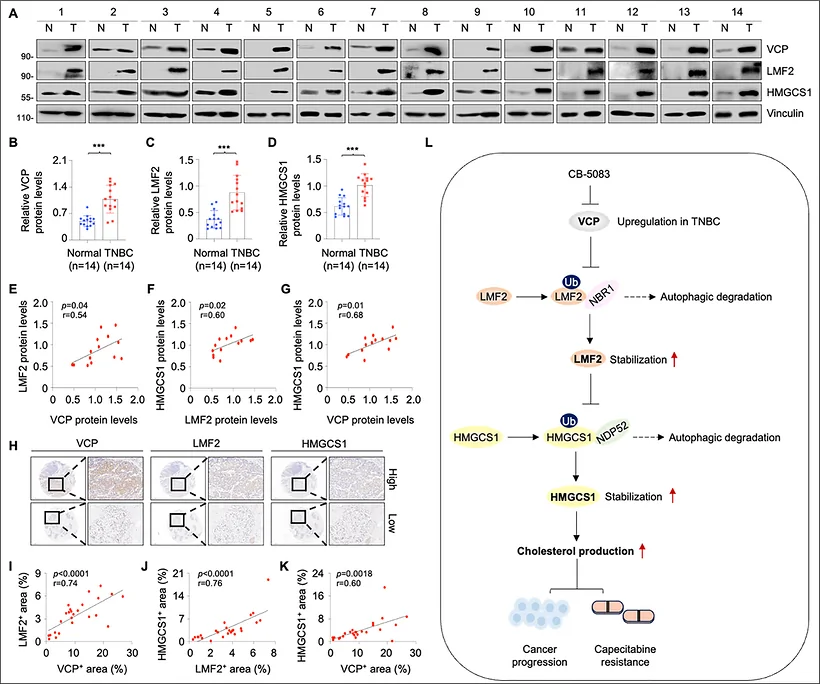
Please replace Figure 7 with the newly uploaded file "figure7.png" in the Attachments. In panel B, the y‐axis upper limit was adjusted so that two data points above 1.5 are fully displayed. All data, statistical analyses, and the figure legend remain completely unchanged.Clinical relevance of the VCP‐LMF2‐HMCGS1 axis in TNBC patient specimens. (A) Immunoblotting analyses of VCP, LMF2, and HMGCS1 protein levels in 14 paired TNBC and adjacent normal breast tissues. (B–D) Quantitative results of relative protein levels of VCP (B), LMF2 (C), and HMGCS1 (D) in TNBC and adjacent normal tissues (*n* = 14). Data are presented as mean ± SD (*n* = 14 pairs of clinical tissues). Statistical significance was determined by a paired two‐tailed Student's *t*‐test. (E–G) Correlation analysis of protein levels between VCP and LMF2 (E), LMF2 and HMGCS1 (F), and VCP and HMGCS1 (G) in TNBC tissues (*n* = 14). Correlations were analyzed by two‐tailed Pearson correlation analysis. (H) IHC staining of VCP, LMF2, and HMGCS1 expression in 25 TNBC samples. The representative images are shown. Top row, high protein expression; bottom row, low protein expression. Scale bars: 500 µm (left, low magnification) and 50 µm (right, high magnification). (I–K) Correlation analysis of IHC‐positive areas for VCP and LMF2 (I), LMF2 and HMGCS1 (J), and VCP and HMGCS1 (K) in 25 TNBC samples. Correlations were analyzed by two‐tailed Pearson correlation analysis. (L) The proposed working model. LMF2 is ubiquitinated, and this modification enables its recognition by NBR1, which subsequently targets it for lysosomal degradation. In TNBC, elevated VCP expression stabilizes LMF2 by counteracting this process. Similarly, HMGCS1 undergoes ubiquitination and is recognized by NDP52, leading to its autophagic degradation. Importantly, LMF2 exerts a protective effect on HMGCS1 by disrupting the HMGCS1–NDP52 interaction, thereby promoting HMGCS1 stability. The resulting enhancement of cholesterol biosynthesis fuels TNBC progression and confers resistance to capecitabine. This entire regulatory axis is effectively dismantled by the VCP inhibitor CB‑5083.

To further validate these findings, we performed immunohistochemical (IHC) staining of VCP, LMF2, and HMGCS1 in 25 TNBC patient samples (Figure 7H). Evaluation of IHC scores demonstrated consistent positive correlations among the expression levels of all three proteins, reinforcing their coordinated expression and clinical relevance in TNBC (Figure 7I–K). Together, these results support the proposed signaling cascade in which VCP stabilizes LMF2, which in turn enhances the protein stability of HMGCS1 in TNBC cells (Figure 7L).

To determine whether LMF2, VCP, and HMGCS1 are enriched in specific tumor niches, we analyzed a single‑cell RNA‑seq dataset from 34 TNBC patients (GEO: GSE246613) [79]. Clustering and cell‑type annotation revealed that this axis was preferentially expressed in cancer cells, with markedly lower levels in normal luminal cells and stromal compartments (fibroblasts and vascular endothelial cells) (Figure S15A). Feature plots reveal prominent co‑localization of all three genes with the stem cell marker CD44 and the proliferation marker MKI67 (Figure S15B, as indicated by the red box), indicating that this axis is highly active in proliferative, stem‑like tumor cells. We further examined epithelial–mesenchymal transition (EMT) markers, as cells at leading invasive margins typically downregulate epithelial traits and acquire mesenchymal characteristics program [80, 81]. Co‑expression profiles show that the axis is negatively correlated with the epithelial marker CDH1 (E‑cadherin), while positively correlated with the mesenchymal marker VIM (vimentin), and the EMT transcription factor ZEB1 (Figure S15B, as indicated by red box). Collectively, these single‑cell transcriptomic analysis provides evidence that the VCP–LMF2–HMGCS1 axis is preferentially active in aggressive, stem‑like and invasive front subpopulations.

## 4 Discussion

TNBC remains a major challenge and bottleneck in the clinical management of breast cancer due to its aggressive nature, scarcity of effective therapeutic targets, and frequent development of chemoresistance. In this study, we uncovered several interesting findings concerning the previously unexplored functional and mechanistic roles of LMF2 in driving cancer progression and conferring resistance to capecitabine in this recalcitrant disease (Figure 7L).

First, we provide the first evidence that LMF2 acts as an oncogenic driver to promote tumor growth, metastasis, and capecitabine resistance in TNBC. As the largest organelle in eukaryotic cells, the ER plays an essential role in cellular homeostasis and is a hotspot for a variety of key cellular functions, such as lipid metabolism, calcium homeostasis, and the synthesis, folding, modification, and trafficking of transmembrane and secreted proteins [82, 83, 84]. Consequently, disruption of ER homeostasis by various factors in the tumor and its microenvironment, such as hypoxia and nutrient deprivation, induces ER stress and triggers the ER stress response [83]. The response is primarily mediated by three ER transmembrane proteins, including inositol‐requiring enzyme 1α, PKR‐like endoplasmic reticulum kinase, and activating transcription factor 6 [85]. Notably, dysregulation of these proteins has been intimately linked to tumor growth and metastasis as well as therapeutic responses in TNBC [86, 87, 88, 89, 90]. By analyzing our recently published large‐scale transcriptomic [11] and proteomic [12] datasets of TNBC, we initially discovered that LMF2, an ER‐localized transmembrane protein, is significantly upregulated in TNBC tissues (Figure 1). However, its biological functions, particularly in cancer progression and therapeutic responsiveness, remain completely unknown. In this study, we demonstrated that LMF2 drives TNBC progression and confers resistance to capecitabine (Figures 1 and 2), identifying it as a potential biomarker and therapeutic target for this disease. However, our study did not address whether LMF2 exerts similar oncogenic functions in other breast cancer subtypes, which remains an important question for future investigation.

Second, we revealed that VCP stabilizes LMF2 by blocking its autophagic degradation mediated by the autophagy receptor NBR1. To date, the upstream regulatory mechanism of LMF2 in both normal and cancer cells remains unknown. Previous studies have shown that ER‐localized proteins are targeted for degradation via two key pathways, namely ER‐associated degradation (ERAD) and ER‐to‐lysosome degradation, which direct their substrates to the proteasome and lysosome for degradation, respectively [91, 92]. In this study, we demonstrate that LMF2 undergoes selective autophagic degradation mediated by the autophagy cargo receptor NBR1, and that VCP counteracts this process to stabilize LMF2 by disrupting the LMF2‐NBR1 interaction (Figure 3). Accordingly, pharmacological inhibition of VCP with its selective inhibitor CB‐5083 [35, 51] effectively attenuated LMF2‐driven tumor progression and reversed capecitabine resistance in TNBC (Figure 4). This mechanism aligns with our recent finding that VCP also blocks the autophagy receptor TOLLIP‐mediated autophagic degradation of transmembrane protein 63A in TNBC cells [37], underscoring a broader role for VCP in regulating cargo receptor‐mediated autophagy.

NBR1 is an autophagy cargo receptor for selective autophagosomal degradation of ubiquitinated targets, such as major histocompatibility complex class I and IκB kinase α [66, 93, 94]. Having confirmed that LMF2 is a ubiquitinated protein, we actually sought to identify its relevant E3 ubiquitin ligase. We found that the E3 ligase TRIM21, which has been documented to mediate ubiquitin‐dependent autophagic degradation of substrates such as IRF3 and G3BP1 [95, 96], is not responsible for LMF2 ubiquitination. Thus, future studies are warranted to identify the specific E3 ubiquitin ligases and deubiquitinating enzymes that regulate this process. Additionally, LMF2 contains seven predicted lysine ubiquitination sites distributed throughout the protein, as annotated in the PhosphoSitePlus database ( https://www.phosphosite.org ). However, fine‐mapping of these individual ubiquitination sites in the context of NBR1‐mediated LMF2 degradation remains an important direction for future research. Moreover, previous studies have shown that NBR1 is frequently downregulated in human breast cancer cells [97], but paradoxically drives pro‐metastatic differentiation and outgrowth of breast cancer under conditions of autophagy deficiency [98, 99], underscoring its context‐dependent role in breast cancer progression.

In addition to its central role in ERAD [34], VCP is also critically involved in multiple stages of autophagy, including initiation, maturation, and autophagic degradation [36, 100, 101]. For instance, VCP stabilizes Beclin‐1, a key regulator of macroautophagy, by enhancing the deubiquitinase activity of ataxin‐3 toward Beclin‐1, thereby promoting autophagy initiation [36]. Moreover, VCP is highly expressed in breast cancer stem‐like cells and plays an essential role in tumorigenesis and cancer progression [102]. Notably, pharmacological inhibition of VCP selectively triggers paraptosis in breast cancer cells without affecting normal cells [103], underscoring its promising therapeutic potential for treating LMF2‐driven TNBC.

Third, LMF2 suppresses NDP52‐mediated autophagic degradation of HMGCS1 and enhances cholesterol biosynthesis. Accumulating evidence shows that HMGCS1 plays an oncogenic role in breast cancer and other cancers and is associated with reduced drug sensitivity [104, 105]. Although previous studies have documented that HMGCS1 undergoes ubiquitin‐dependent proteasomal degradation mediated by the E3 ubiquitin‐protein ligases SPOP [29] and CTLH [24] in other model systems, the underlying mechanisms governing HMGCS1 protein stability in TNBC remain largely elusive. In this study, we demonstrated that HMGCS1 is predominantly degraded via the autophagy‐lysosome pathway in TNBC cells, and that LMF2 counteracts this process to stabilize HMGCS1. Mechanistically, LMF2 disrupts the interaction between HMGCS1 and the autophagic receptor NDP52, thereby preventing NDP52‐mediated autophagic degradation (Figure 5). Our finding is supported by an independent proteome‐scale human interactome study, which had documented an interaction between LMF2 and NDP52 [106].

NDP52 was originally identified as an innate immune autophagy receptor for ubiquitylated bacteria, thus coordinating their destruction by the autophagy pathway [76]. Subsequent studies have shown that it mediates the lysosomal degradation of distinct substrates, such as Toll‐like receptor (TLR) signaling molecules TRIF and TNF receptor‐associated factor 6 [73], transcription factor p65 [107], Rab22a‐NeoF fusion protein [108], and mitochondrial antiviral signaling protein (MAVS) [74]. The human NDP52 protein contains three key domains, including an N‐terminal SKICH domain, a central coiled‐coil (CC) domain, and a C‐terminal LIM‐K domain [72, 73, 74, 75]. Critically, these domains mediate distinct functions in substrate binding and facilitating degradation [72, 73, 74, 75]. In our study, we found that the LIM‐K domain of NDP52 mediates binding to HMGCS1, whereas its SKICH and CC domains are required for facilitating HMGCS1 degradation. Despite this functional insight, the expression status and role of NDP52 in breast cancer remain uncharacterized to date.

Consistent with the critical role of HMGCS1 in cholesterol biosynthesis [23, 24], we further demonstrated that LMF2 enhances cholesterol levels both in the culture supernatant and in the serum of xenograft tumor‐bearing mice. Multiple clinical and experimental studies have shown that cholesterol not only plays a crucial role in tumorigenesis by activating multiple oncogenic signaling pathways [54, 109], but also promotes tumor metastasis and therapeutic resistance by facilitating epithelial‐mesenchymal transition, impairing immune cell function, conferring resistance to cell death in tumor cells [54, 109, 110], and modulating the expression and activity of ATP‐binding cassette (ABC) multidrug efflux transporters [111]. Statins (HMGCR inhibitors) not only suppress tumor growth and metastatic potential by blocking intracellular cholesterol synthesis, but also enhance the susceptibility of cancer cells to chemotherapy and immunotherapy [54, 112, 113]. In line with these findings, activation of the cholesterol biosynthesis pathway has been shown to drive TNBC growth and metastasis [17, 18], while its inhibition via statins or other agents effectively restrains TNBC progression [20, 26].

In support of our proposed model that LMF2 drives TNBC progression and chemoresistance by stabilizing HMGCS1 to enhance cholesterol production, we further demonstrated by functional rescue experiments that either re‐expression of HMGCS1 or supplementation with exogenous cholesterol partially rescues the impaired malignancy and chemoresistance caused by LMF2 loss (Figure 6). Furthermore, the significant co‐upregulation of VCP, LMF2, and HMGCS1 in human TNBC specimens underscores the clinical relevance of this axis (Figure 7).

Finally, we must acknowledge that this study has some limitations. First, although we have demonstrated that LMF2 is a ubiquitinated protein, the specific E3 ubiquitin ligases or deubiquitinating enzymes regulating its ubiquitination remain to be identified. Second, in addition to protein stability, this study did not investigate whether transcriptional or epigenetic regulatory mechanisms also contribute to LMF2 upregulation in TNBC tissues. Third, given the noted function of LMF1 in regulating the maturation and transport of active LPL in lipoprotein metabolism [42], we did not investigate whether LMF2 exerts its oncogenic role in TNBC by regulating LPL in addition to HMGCS1‐mediated cholesterol metabolism.

In summary, findings presented in this study uncover a previously unknown function for ER‐localized LMF2 in driving tumor growth, metastasis, and chemoresistance of TNBC, and reveal its upstream (VCP) and downstream (HMGCS1) regulatory mechanisms in TNBC. Targeting this pathway may represent a promising therapeutic strategy for blocking cancer progression and overcoming chemoresistance in TNBC.

## Author Contributions

**Q.Z**.: data curation, formal analysis, investigation, and writing the manuscript draft. **L.A. and Y.L.Z**.: Data analysis and investigation. **S.Y.Y., Y.X.L., M.Y.H., J.Y.C., and F.L.Z**.: Methodology and validation. **Z.M.S., G.Y.L., A.Y.C., and X.H**.: Clinical specimens and research resources. **D.Q.L**.: Funding acquisition, project administration, and manuscript editing. All authors have read and approved the final submitted manuscript.

## Funding

This work is supported, in whole or in part, by the National Natural Science Foundation of China (82372633 and 82573776 to DQL, and 82203729 to YLZ).

## Ethics Statement

The study protocol involving human participants was reviewed and approved by the Medical Ethics Committee of Fudan University Shanghai Cancer Center (SCCIRB; approval no. NSFC‐25‐0303‐68). All clinical tissue specimens and associated clinical records were collected with written informed consent obtained from all patients prior to study enrollment. All animal experimental procedures were approved by the Institutional Animal Care and Use Committee (IACUC) of Fudan University (approval no. FUSCC‐IACUC‐S2025‐0519) and performed in accordance with institutional guidelines for the care and use of laboratory animals.

## Conflicts of Interest

The authors declare no conflicts of interest.

## Supporting information




**Supporting File 1**: advs78301‐sup‐0001‐SuppMat.docx.

**Supporting File 2**: advs78301‐sup‐0002‐Images.pdf.

## Data Availability

The data that support the findings of this study are available within the article and its supplementary materials. All public and published multi‐omics and drug response datasets analyzed in this study were retrieved from publicly accessible repositories: the FUSCC‐TNBC transcriptomic dataset was obtained from the National Omics Data Encyclopedia (NODE, https://www.biosino.org/node) under accession number OEP000155; the FUSCC‐TNBC proteomic dataset was obtained from the Integrated Proteome Resources (iProX, https://www.iprox.org) under accession number IPX0003222000; the CBCGA transcriptomic dataset was obtained from the Genome Sequence Archive for Human (GSA‐Human, https://ngdc.cncb.ac.cn/gsa‐human) under accession number PRJCA017539; the CBCGA proteomic dataset was obtained from iProX under accession number IPX0006535000; the TCGA breast cancer transcriptomic dataset (TCGA‐BRCA) was accessed via the Genomic Data Commons Data Portal (GDC, https://portal.gdc.cancer.gov); the METABRIC breast cancer dataset was accessed through cBioPortal (https://www.cbioportal.org); the CPTAC breast cancer proteomic dataset (CPTAC‐BRCA) was retrieved from the Clinical Proteomic Tumor Analysis Consortium Data Portal (https://proteomics.cancer.gov/data‐portal); the endoplasmic reticulum–localized gene set was obtained from the Gene Ontology resource under accession number GO:0005783; and the pharmacogenomic profiling data were acquired from the Genomics of Drug Sensitivity in Cancer database (GDSC, https://www.cancerrxgene.org). A comprehensive summary of all datasets and identifiers is detailed in Table S2. All other raw data and relevant materials supporting the findings of this study are available within the article and its Supporting Information. The data that support the findings of this study are available from the corresponding author upon reasonable request.
