# Supplementary material for: VCP‐Regulated LMF2 Stabilizes the Cholesterol Biosynthesis Enzyme HMGCS1 to Drive Cancer Progression and Confer Capecitabine Resistance in Triple‐Negative Breast Cancer
Source: Adv Sci (Weinh). 2026 Oct 20:e78301. Online ahead of print. doi: 10.1002/advs.78301 (PMC13683977; doi:10.1002/advs.78301)

Figure 1I

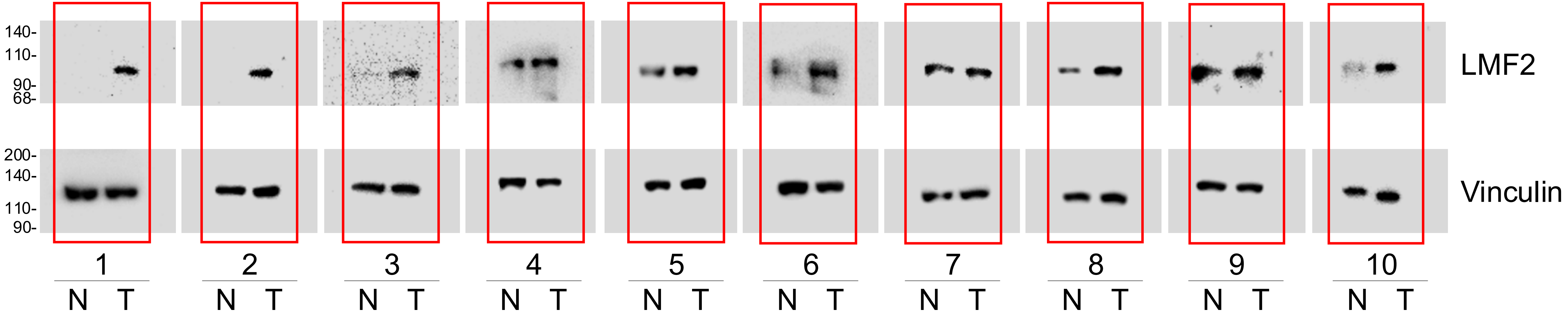
140-
110-
90-
68-
200-
140-
110-
90-
LMF2
Vinculin
1
2
3
4
5
6
7
8
9
10
N T
N T
N T
N T
N T
N T
N T
N T
N T
N T

Figure 2A

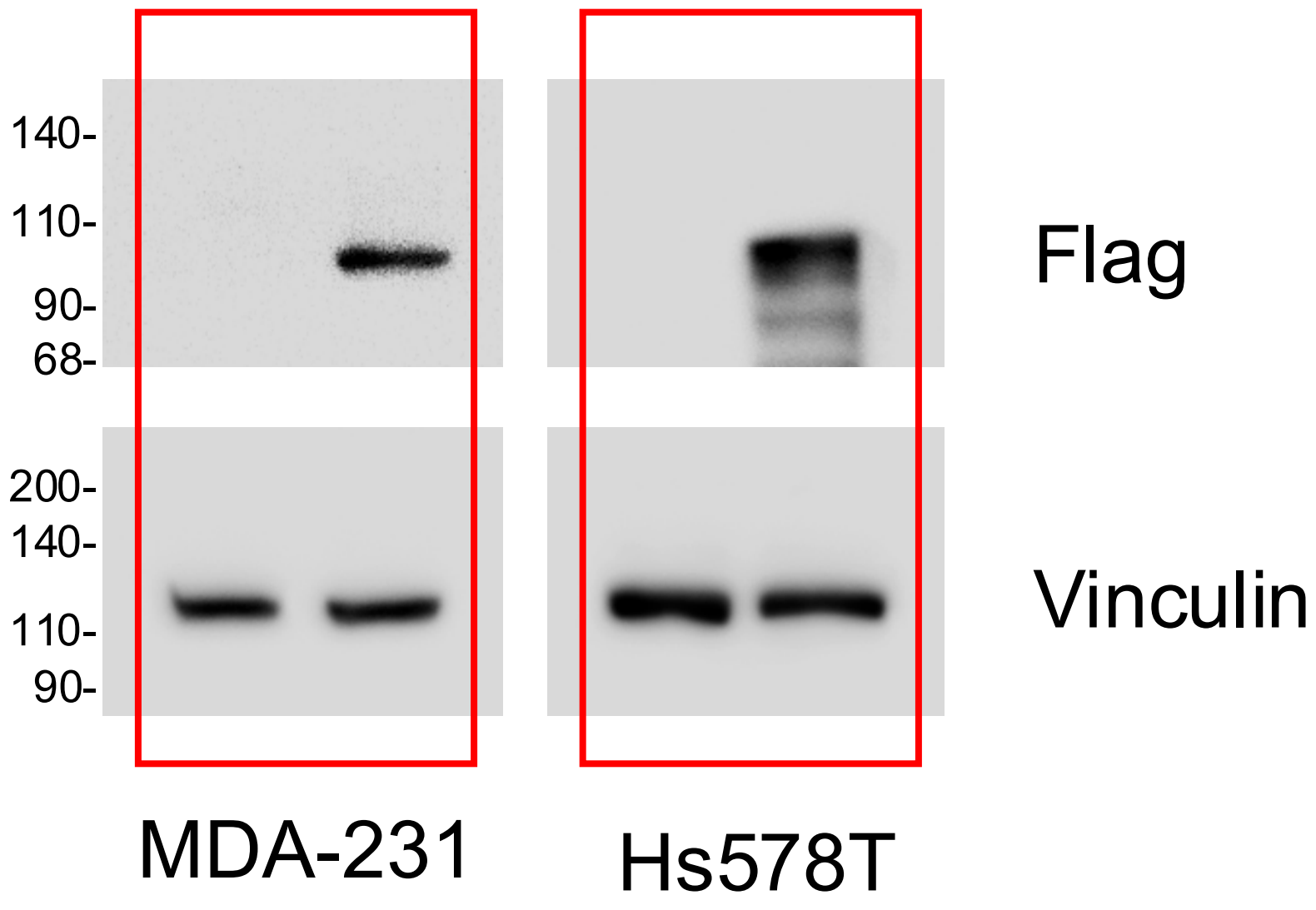

Figure 2B

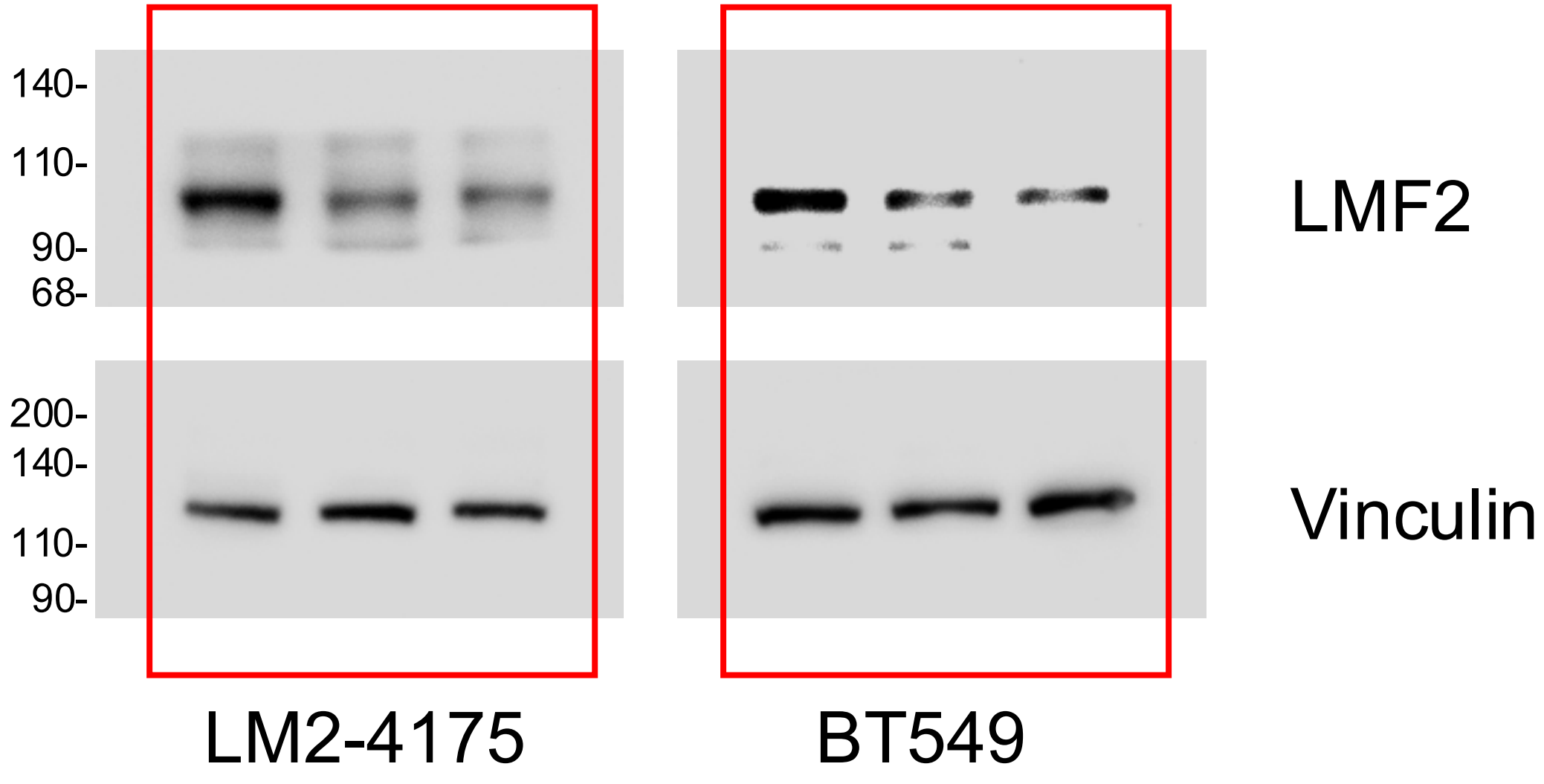

Figure 3B

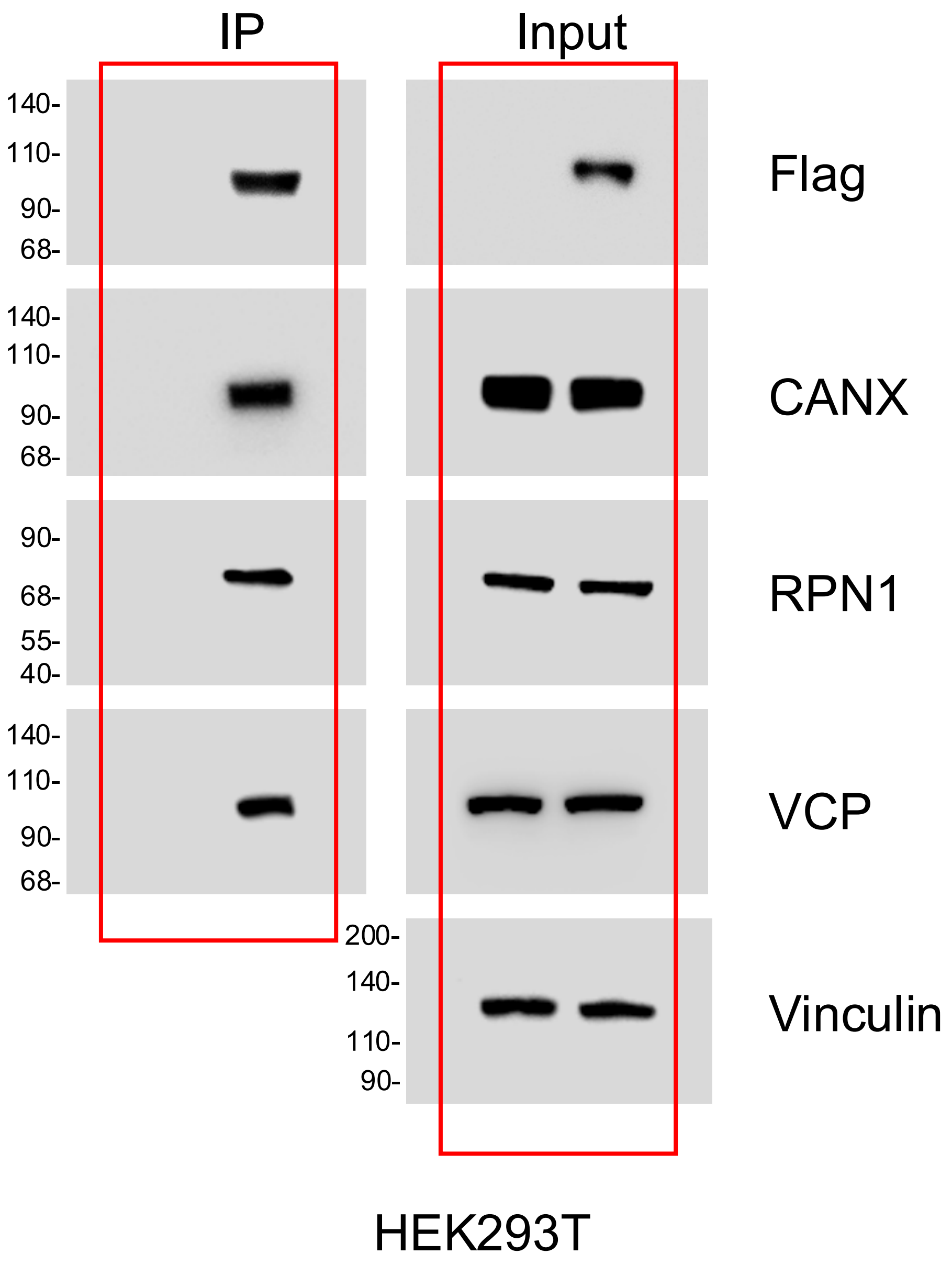

Figure 3C

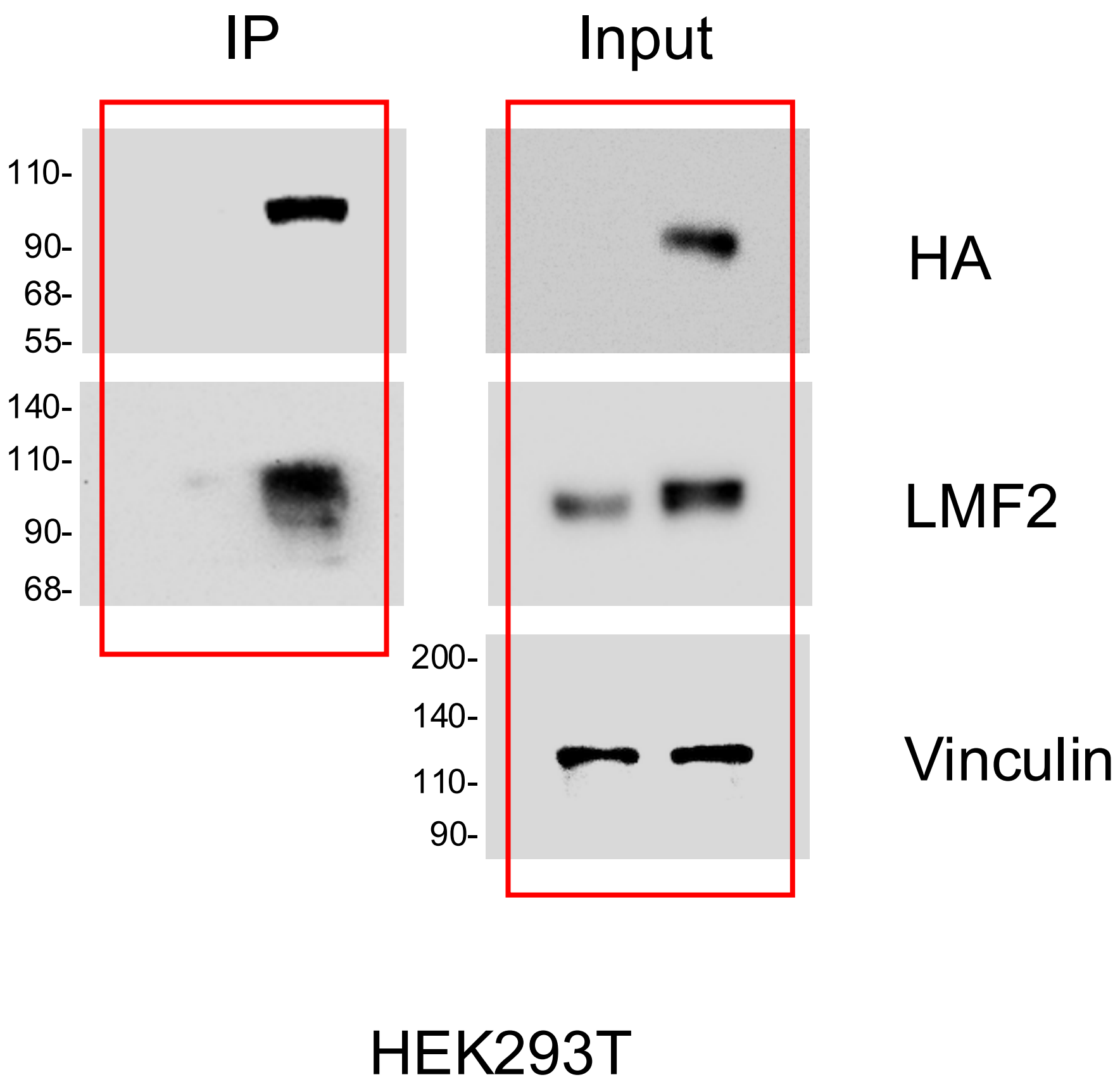

Figure 3D

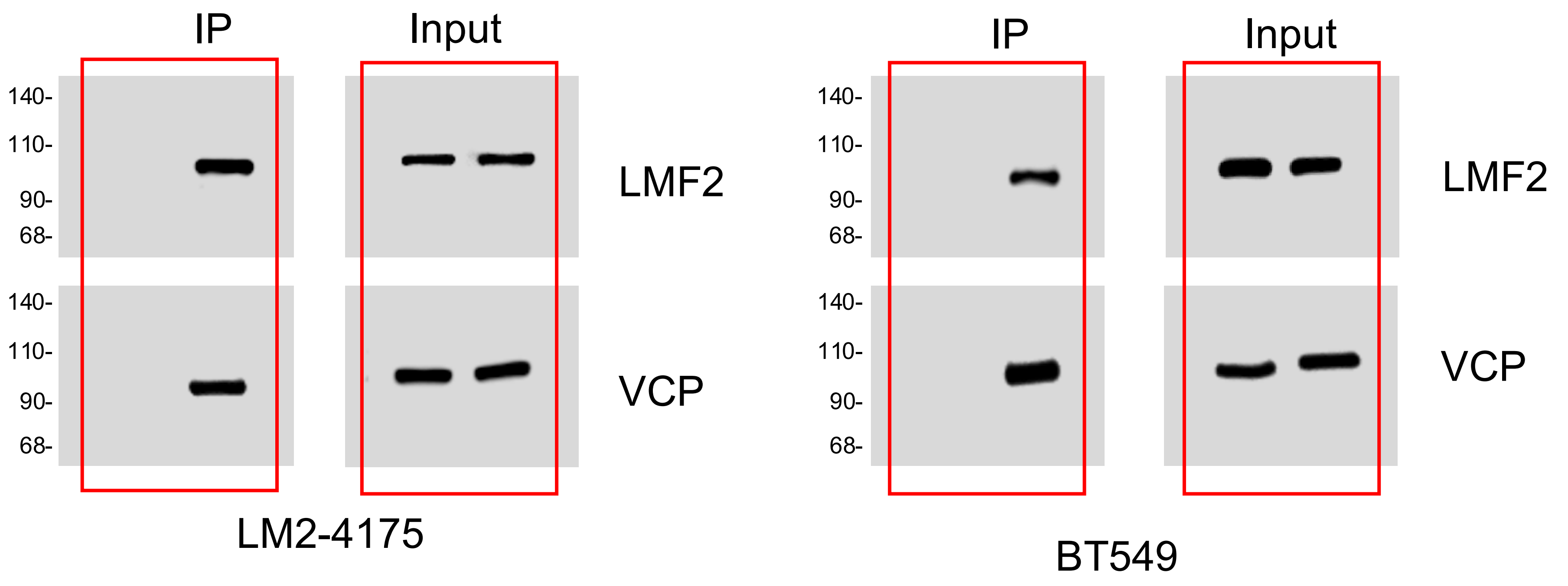

Figure 3F

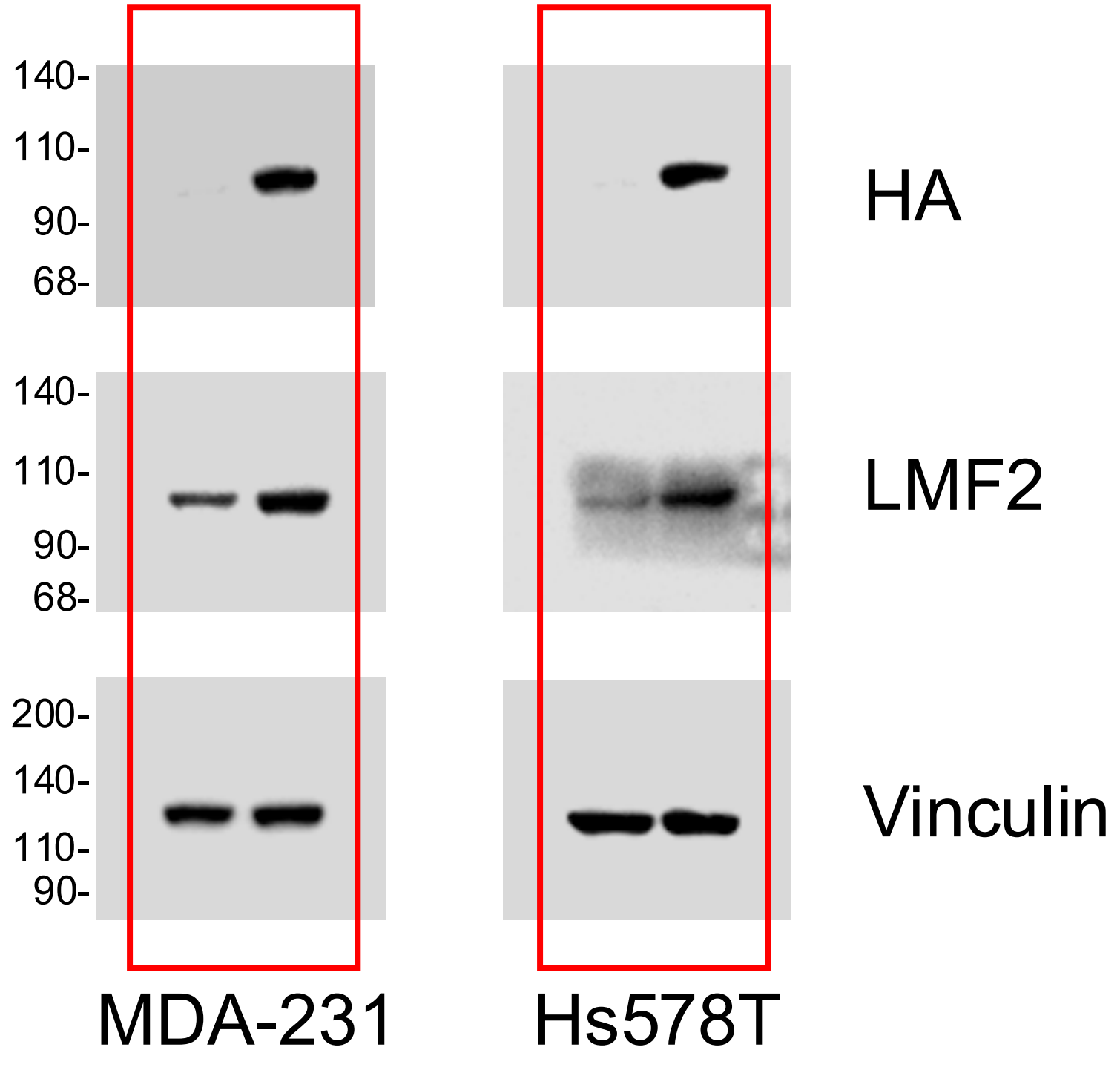

Figure 3G

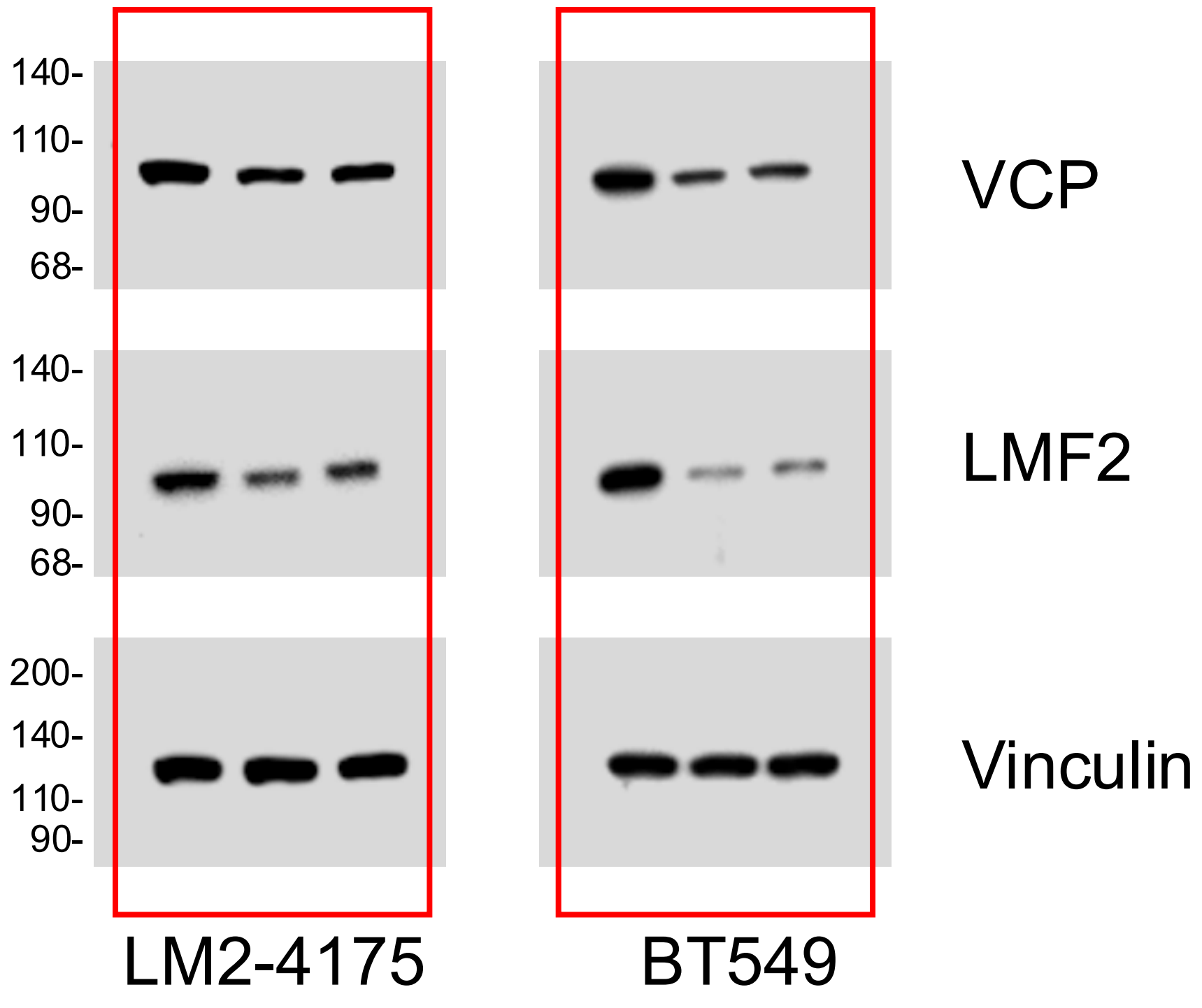

Figure 3H

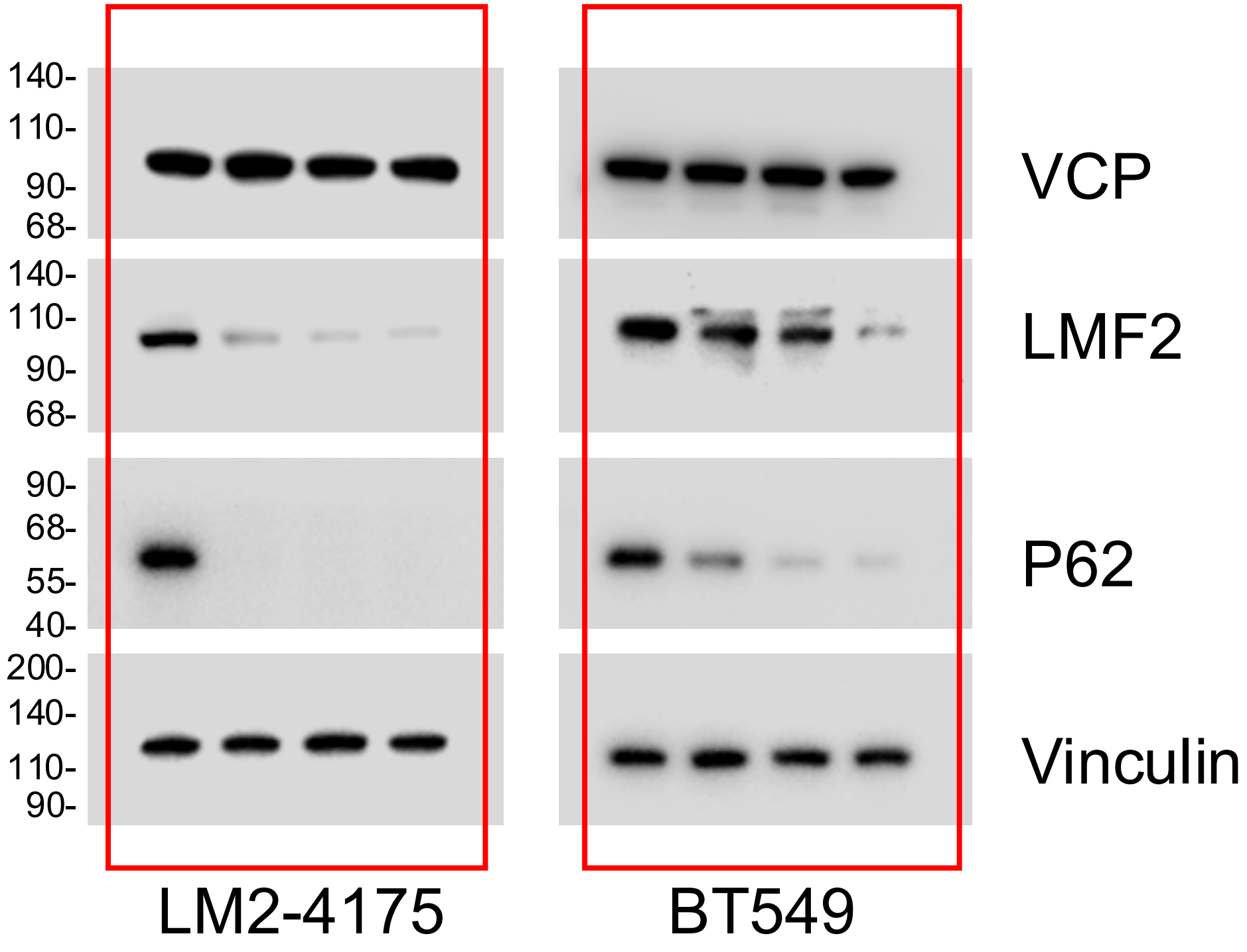

Figure 3J

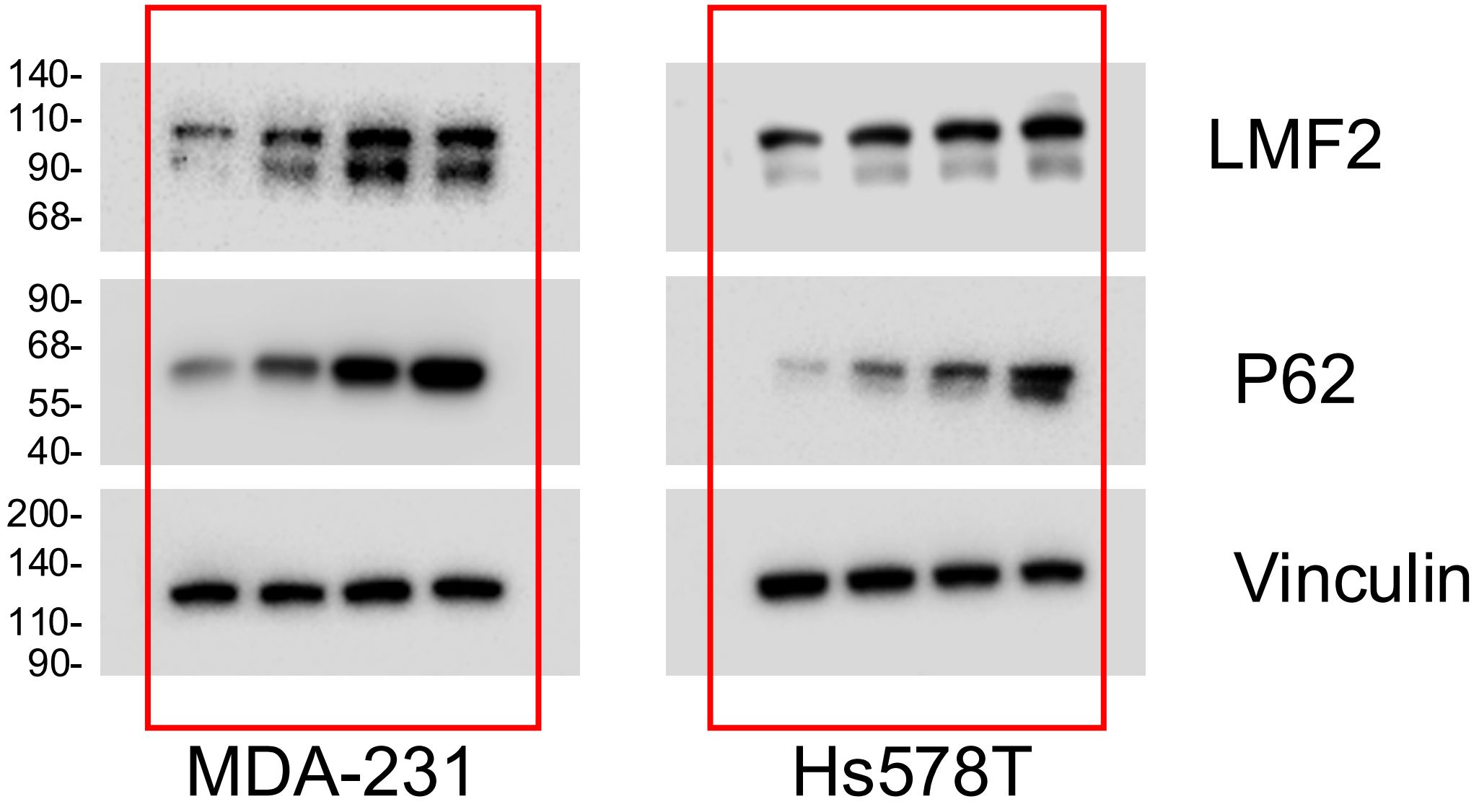

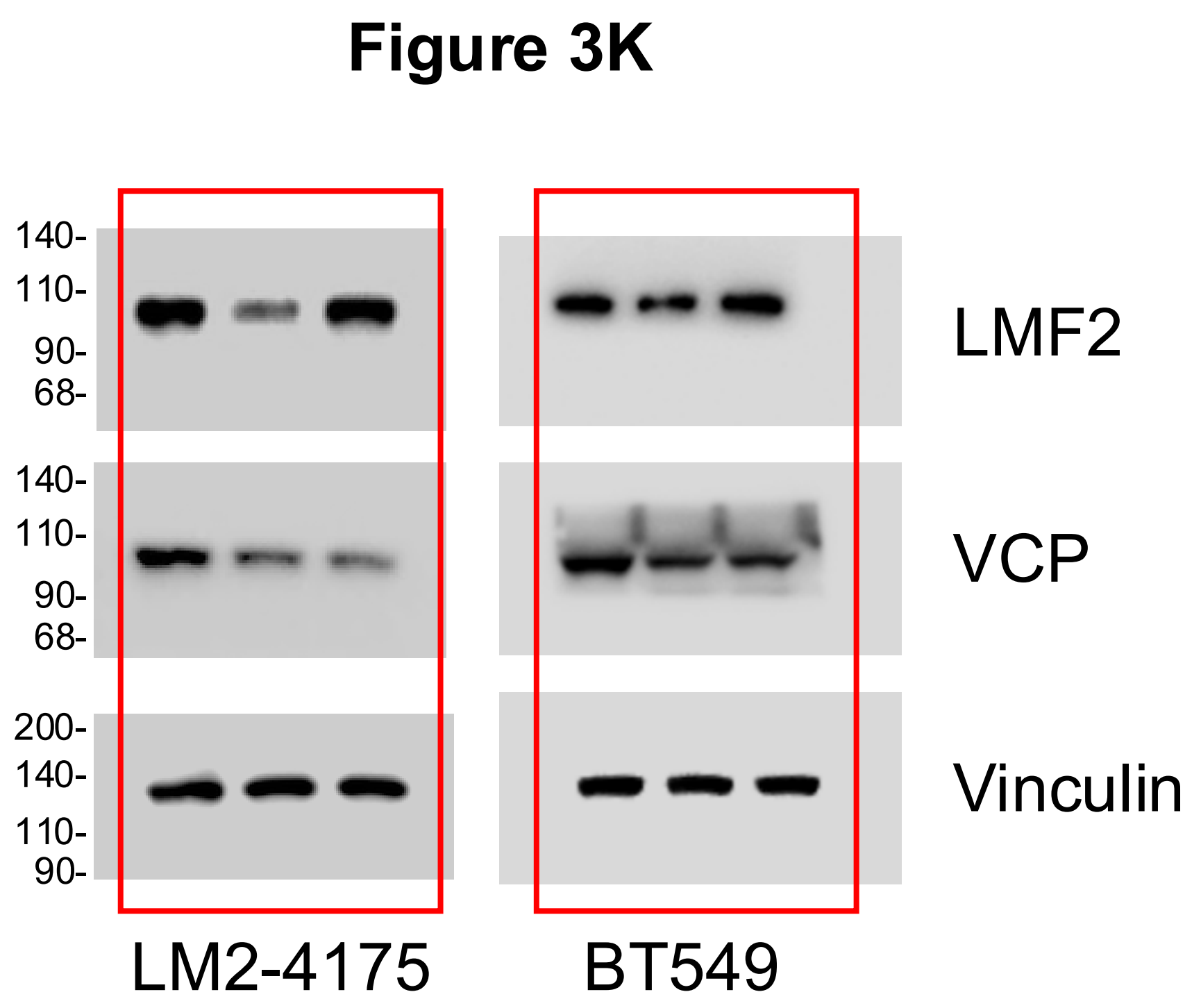

Figure 3K
140-
110-
90-
68-
LMF2
140-
110-
90-
68-
VCP
200-
140-
110-
90-
Vinculin
LM2-4175
BT549

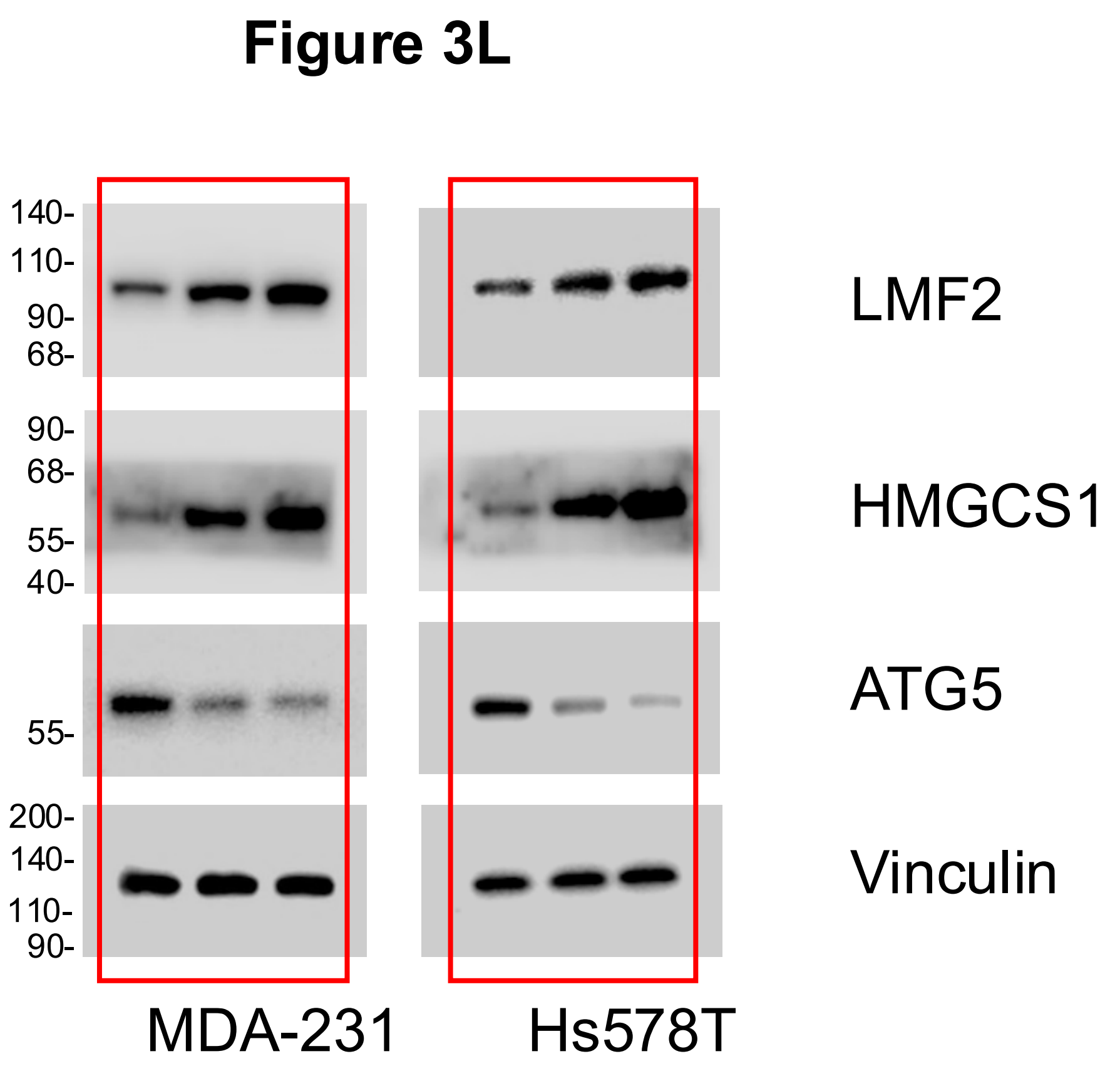

Figure 3L
140-
110-
90-
68-
LMF2
90-
68-
55-
40-
HMGCS1
ATG5
55-
200-
140-
110-
90-
Vinculin
MDA-231
Hs578T

Figure 3M

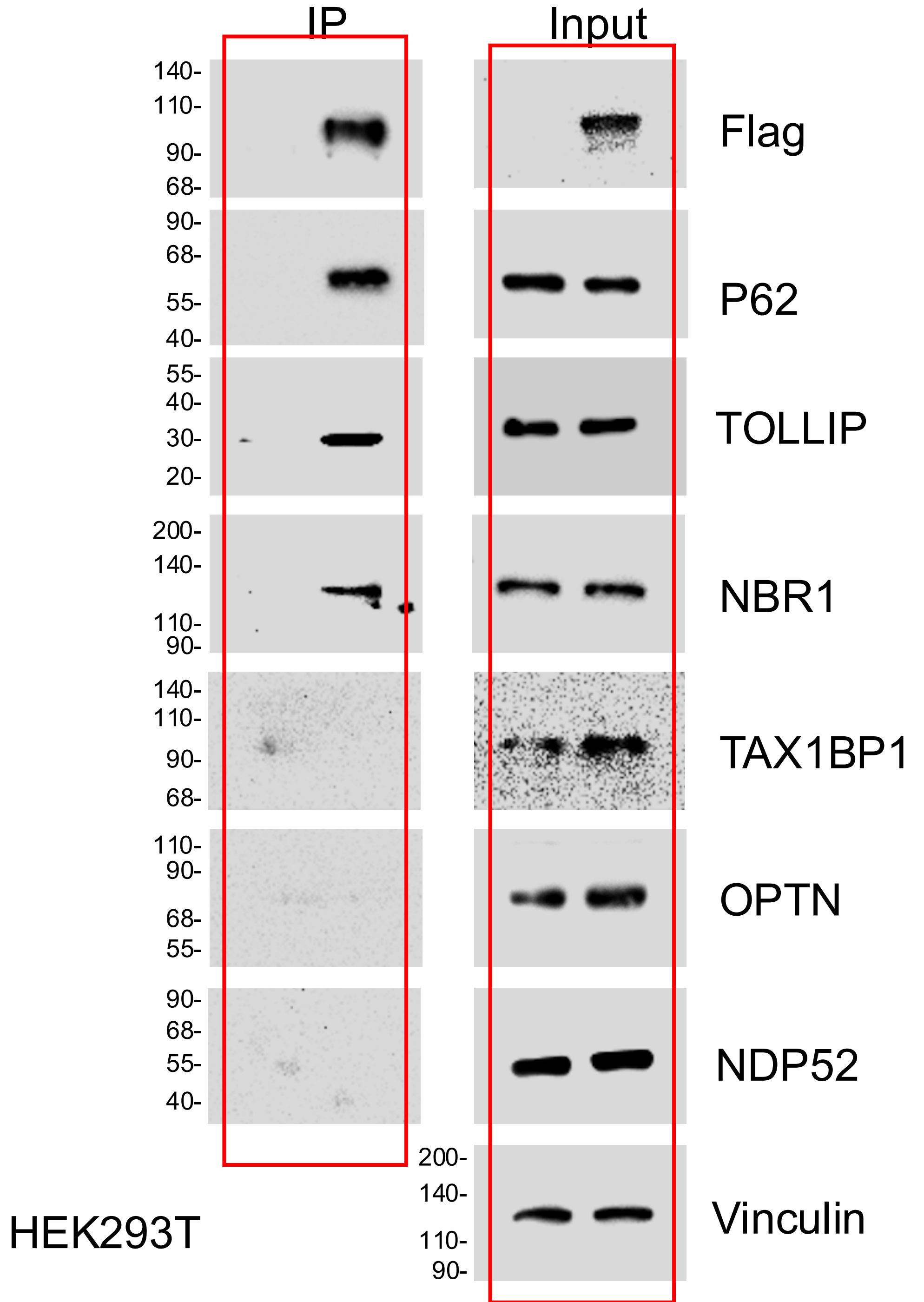
IP
Input
140-
110-
90-
68-
Flag
90-
68-
55-
40-
P62
55-
40-
30-
20-
TOLLIP
200-
140-
110-
90-
NBR1
140-
110-
90-
68-
TAX1BP1
110-
90-
68-
55-
OPTN
90-
68-
55-
40-
NDP52
200-
140-
110-
90-
Vinculin
HEK293T

Figure 3N

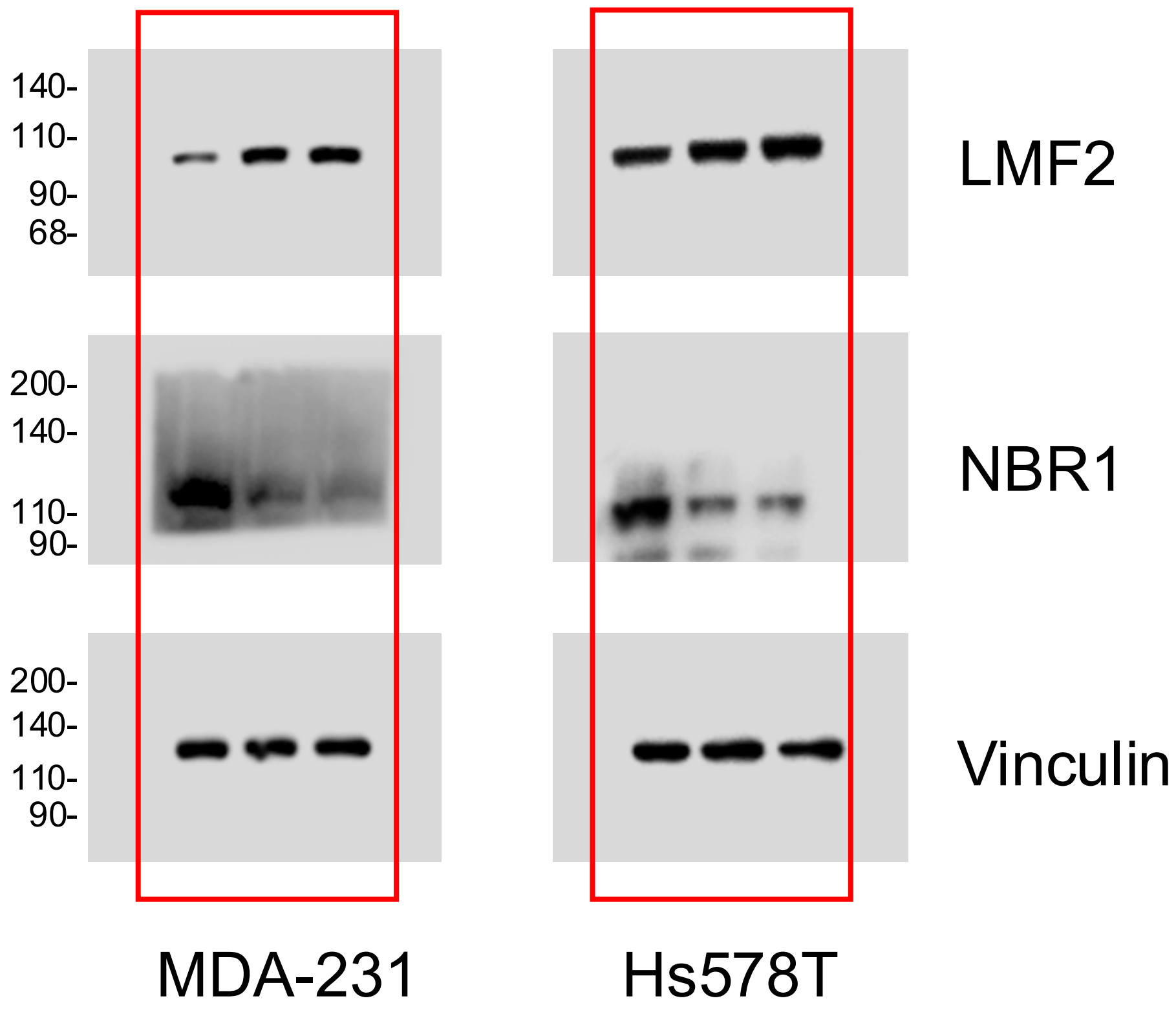

Figure 3O

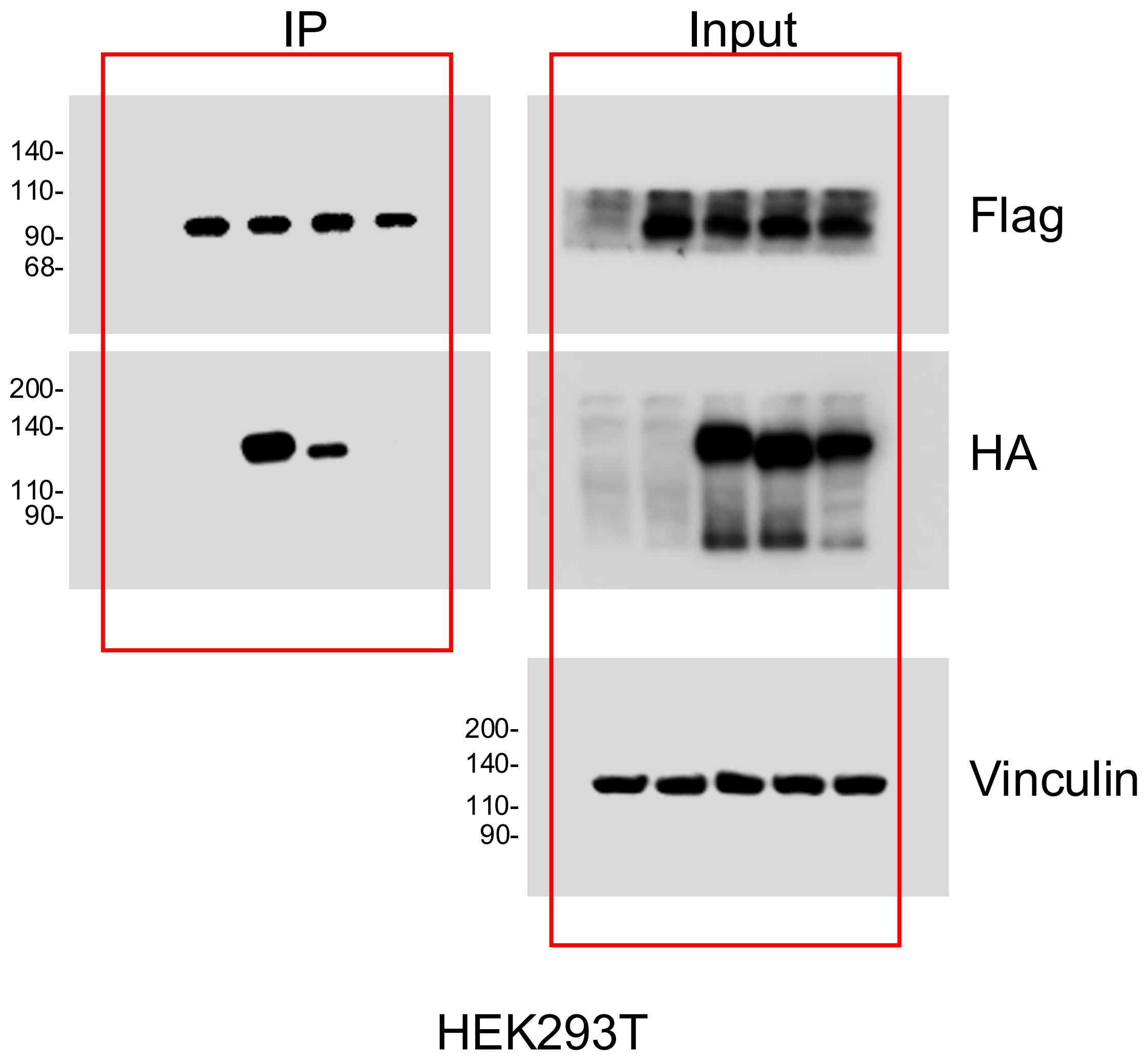

Figure 3P

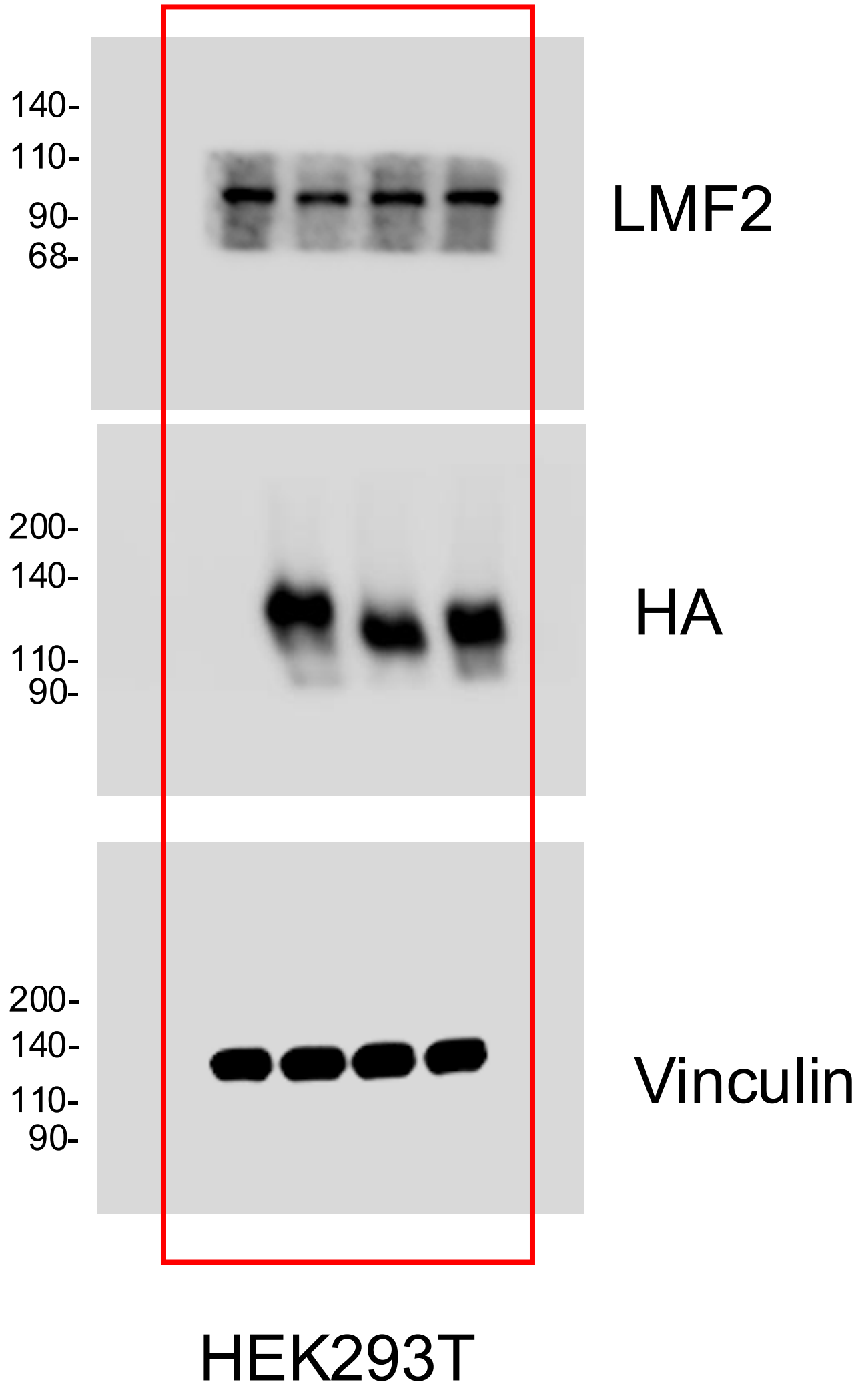

Figure 3Q

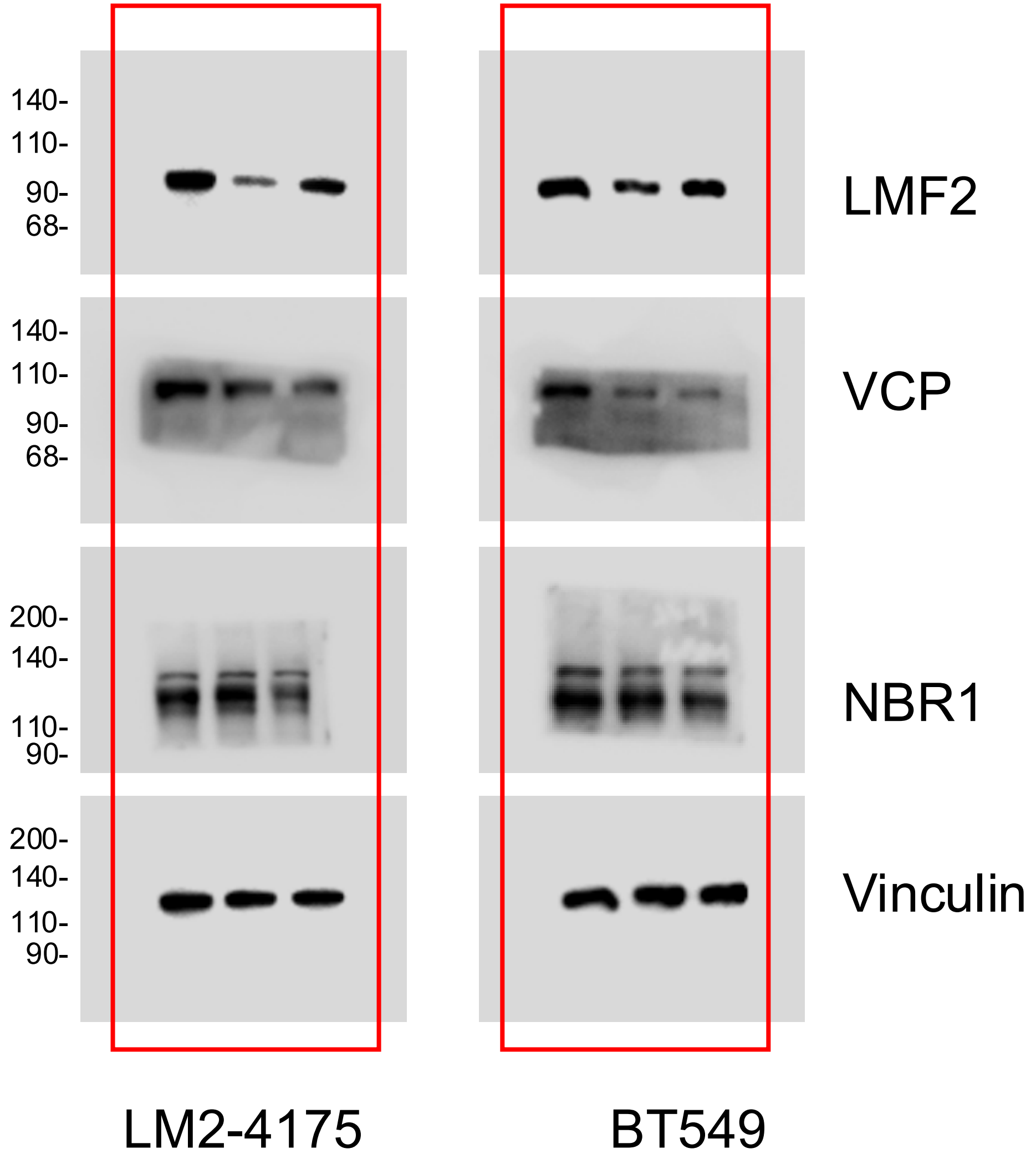

Figure 3R

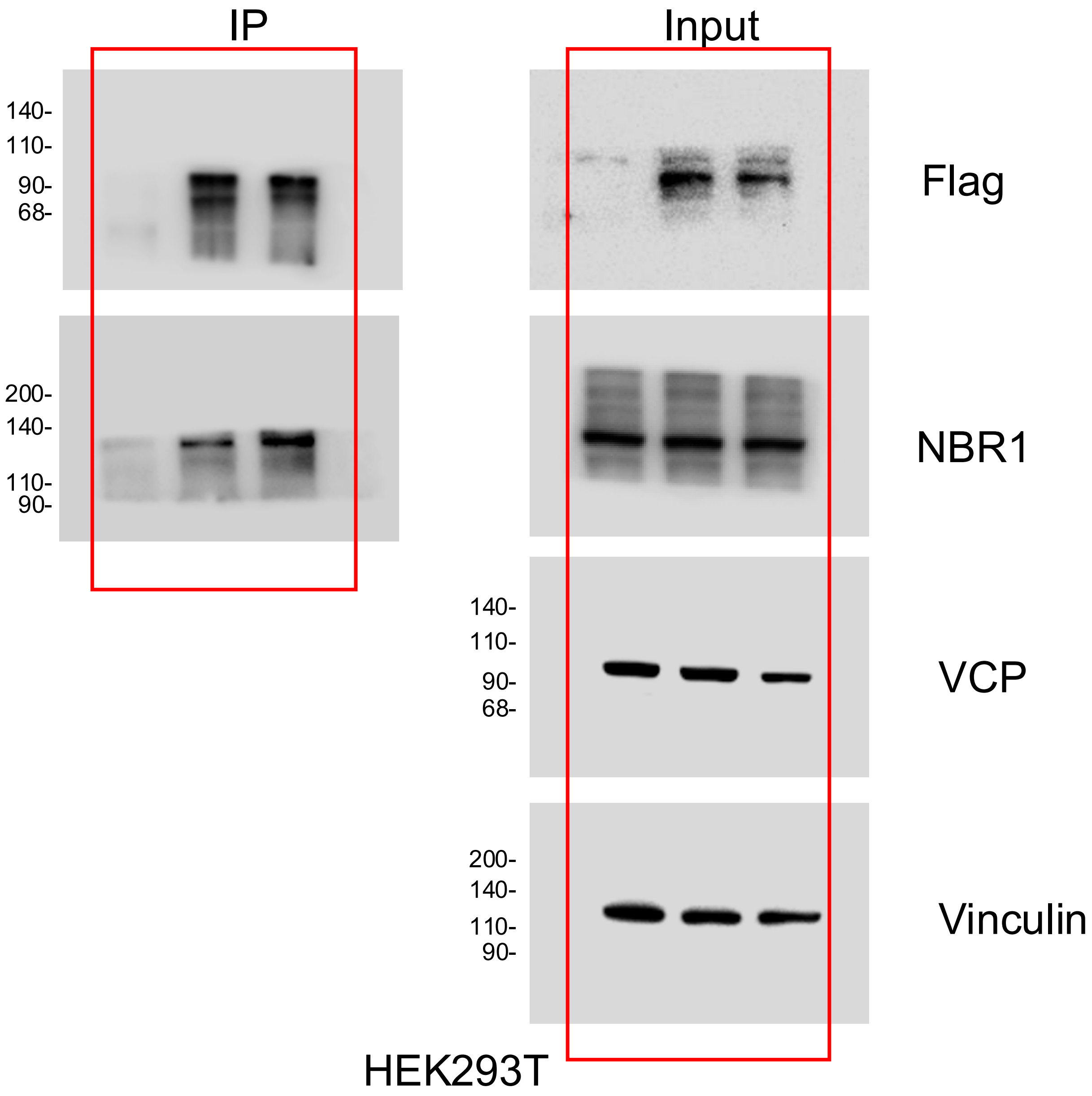
IP
Input
140-
110-
90-
68-
200-
140-
110-
90-
Flag
NBR1
140-
110-
90-
68-
VCP
200-
140-
110-
90-
Vinculin
HEK293T

Figure 5A

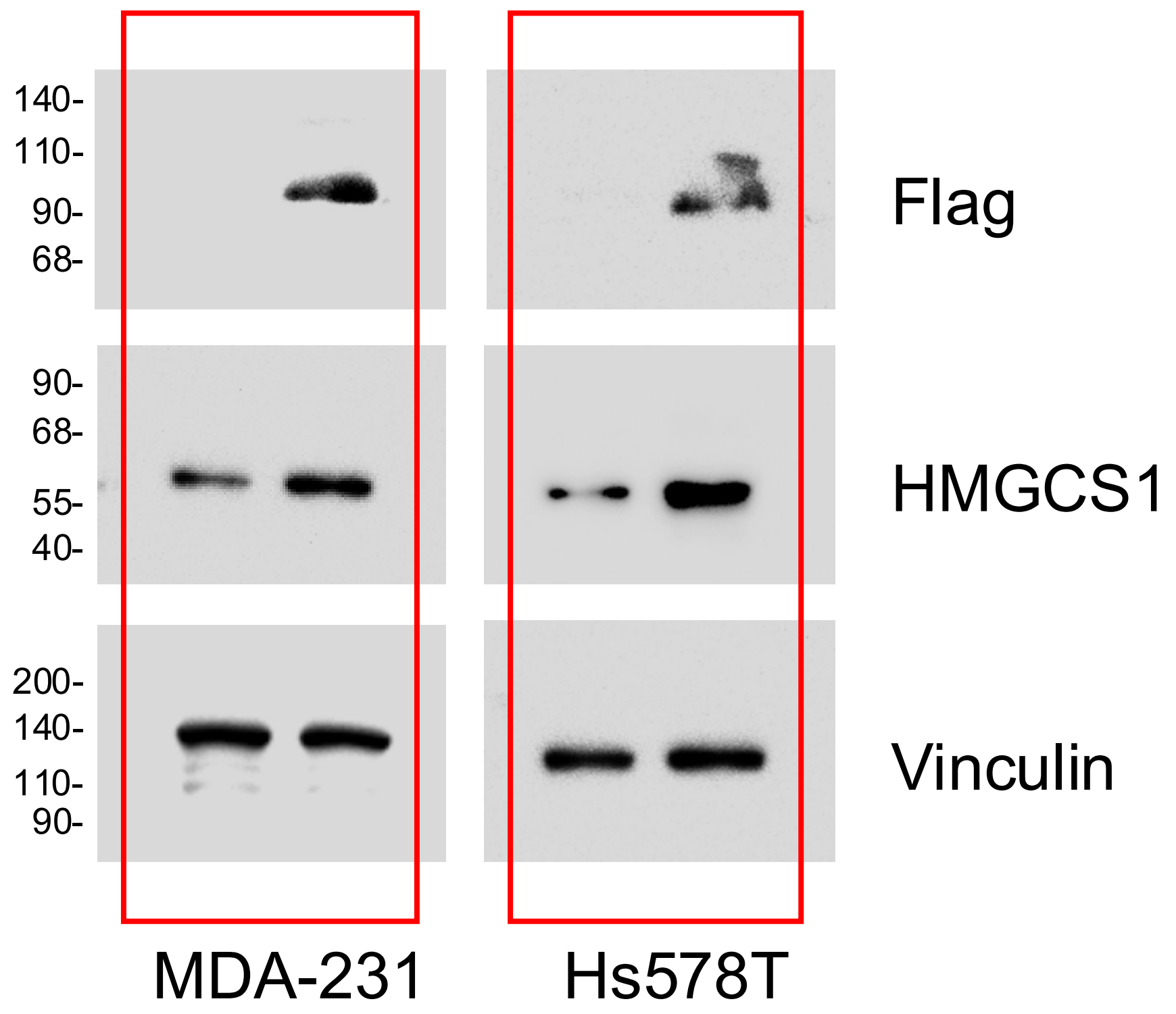

Figure 5B

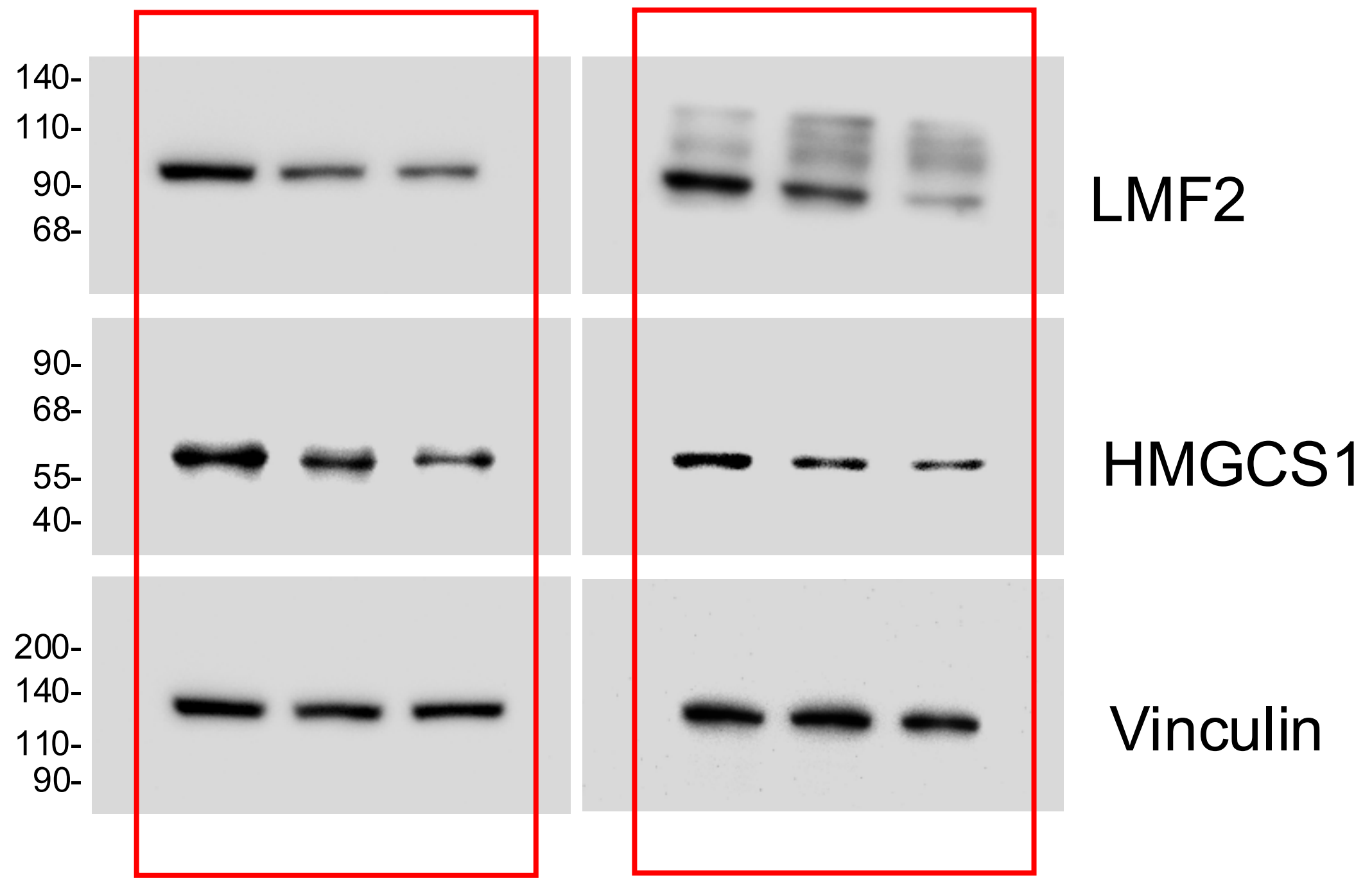

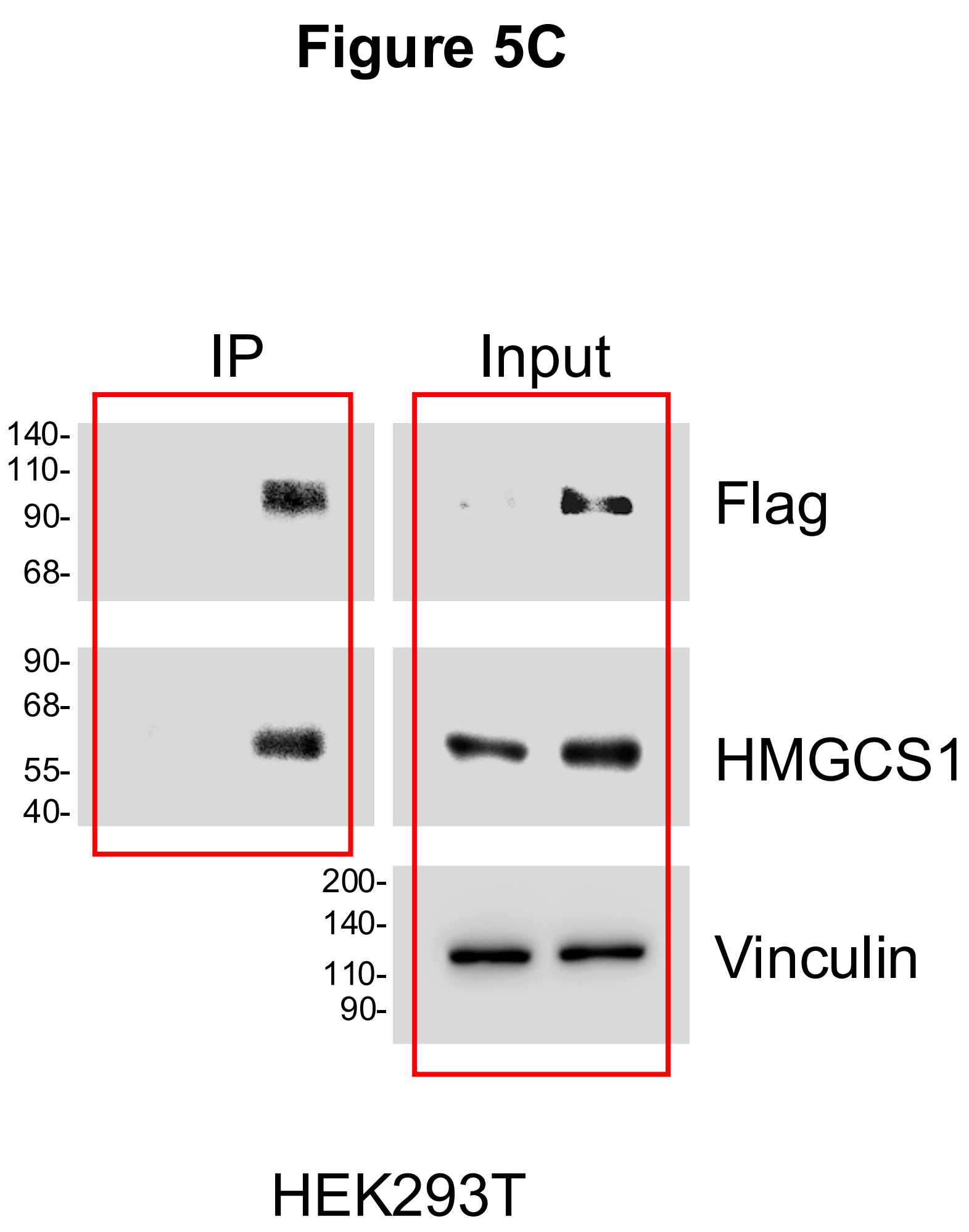
Figure 5C
IP
Input
140-
110-
90-
68-
Flag
90-
68-
55-
40-
HMGCS1
200-
140-
110-
90-
Vinculin
HEK293T

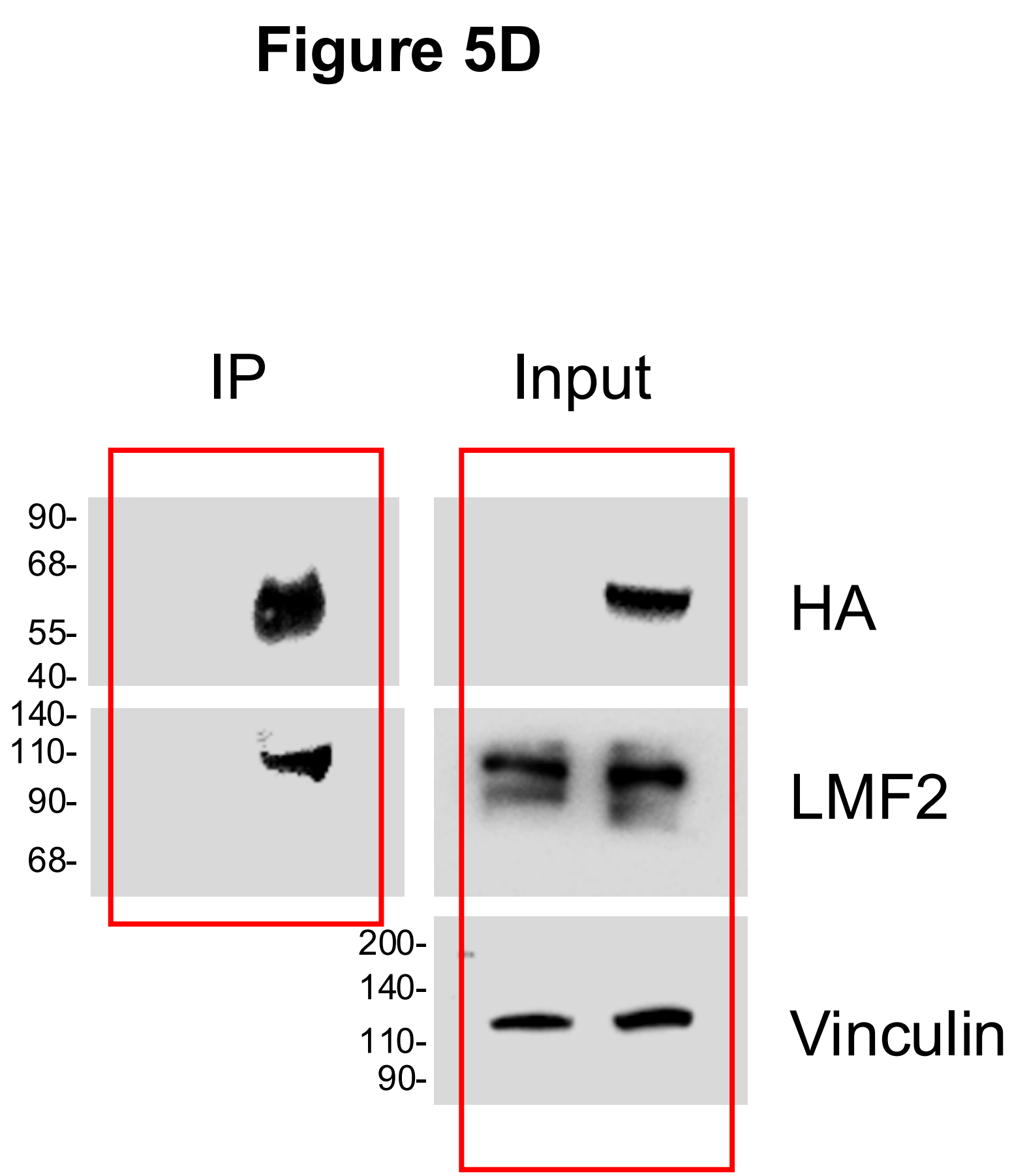
Figure 5D
IP
Input
90-
68-
55-
40-
HA
140-
110-
90-
68-
LMF2
200-
140-
110-
90-
Vinculin

Figure 5E

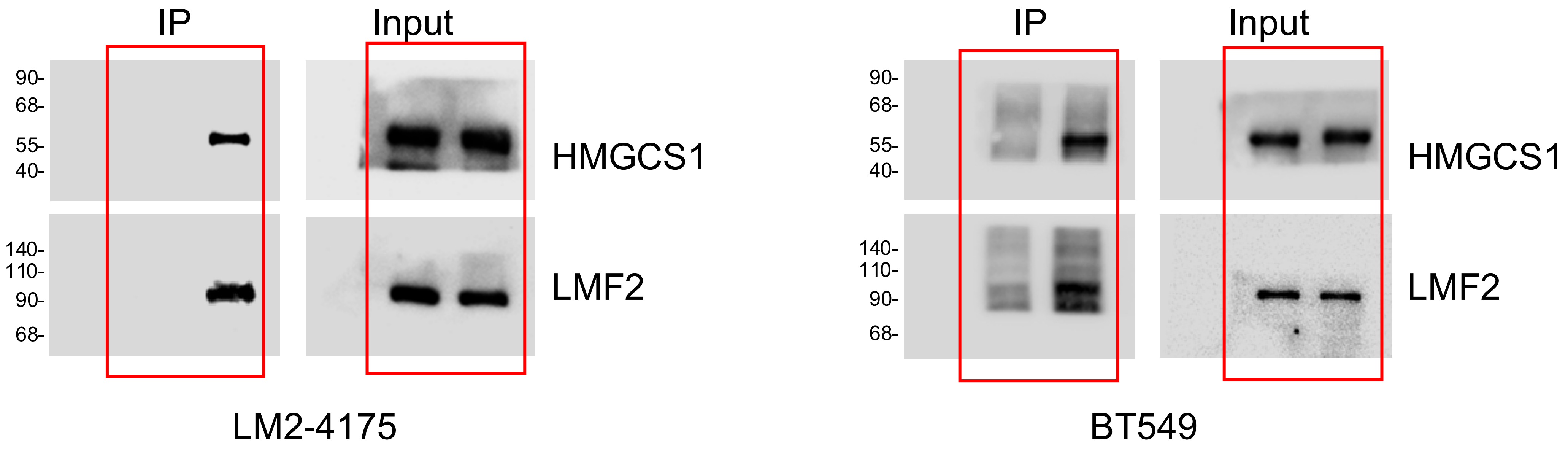
IP
Input
90-
68-
55-
40-
HMGCS1
140-
110-
90-
68-
LMF2
LM2-4175
IP
Input
90-
68-
55-
40-
HMGCS1
140-
110-
90-
68-
LMF2
BT549

Figure 5H

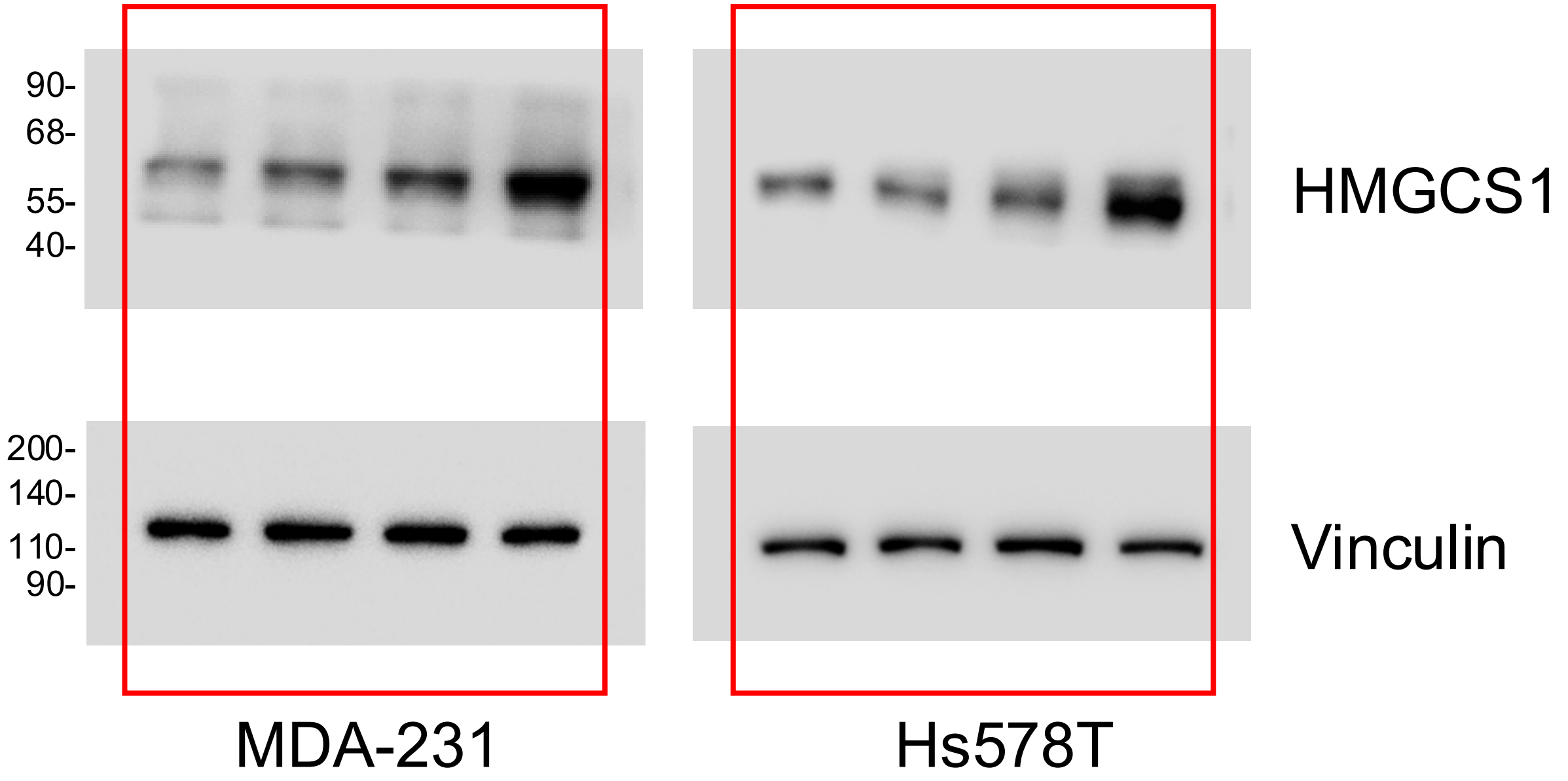

Figure 5I

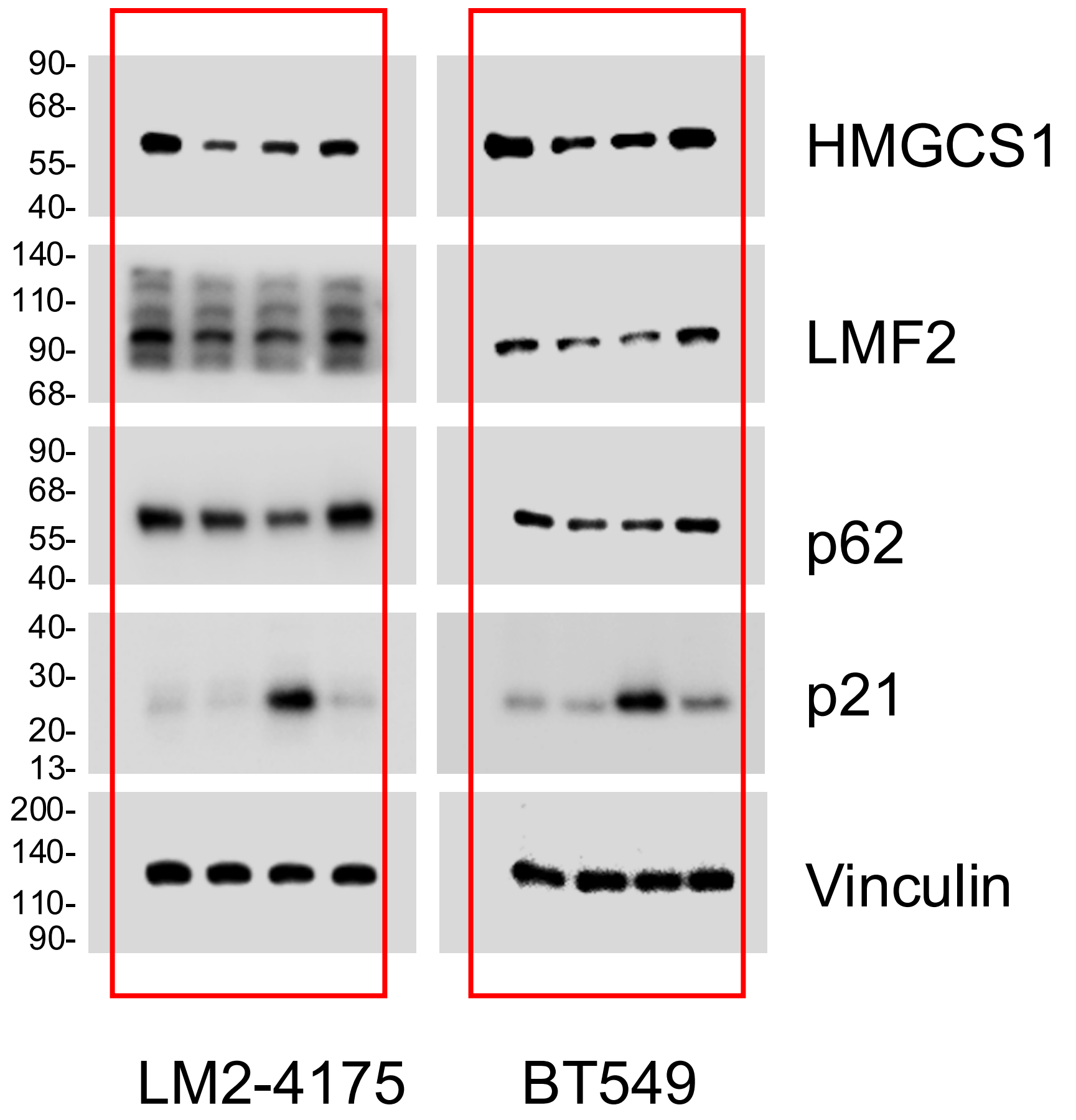

Figure 5J

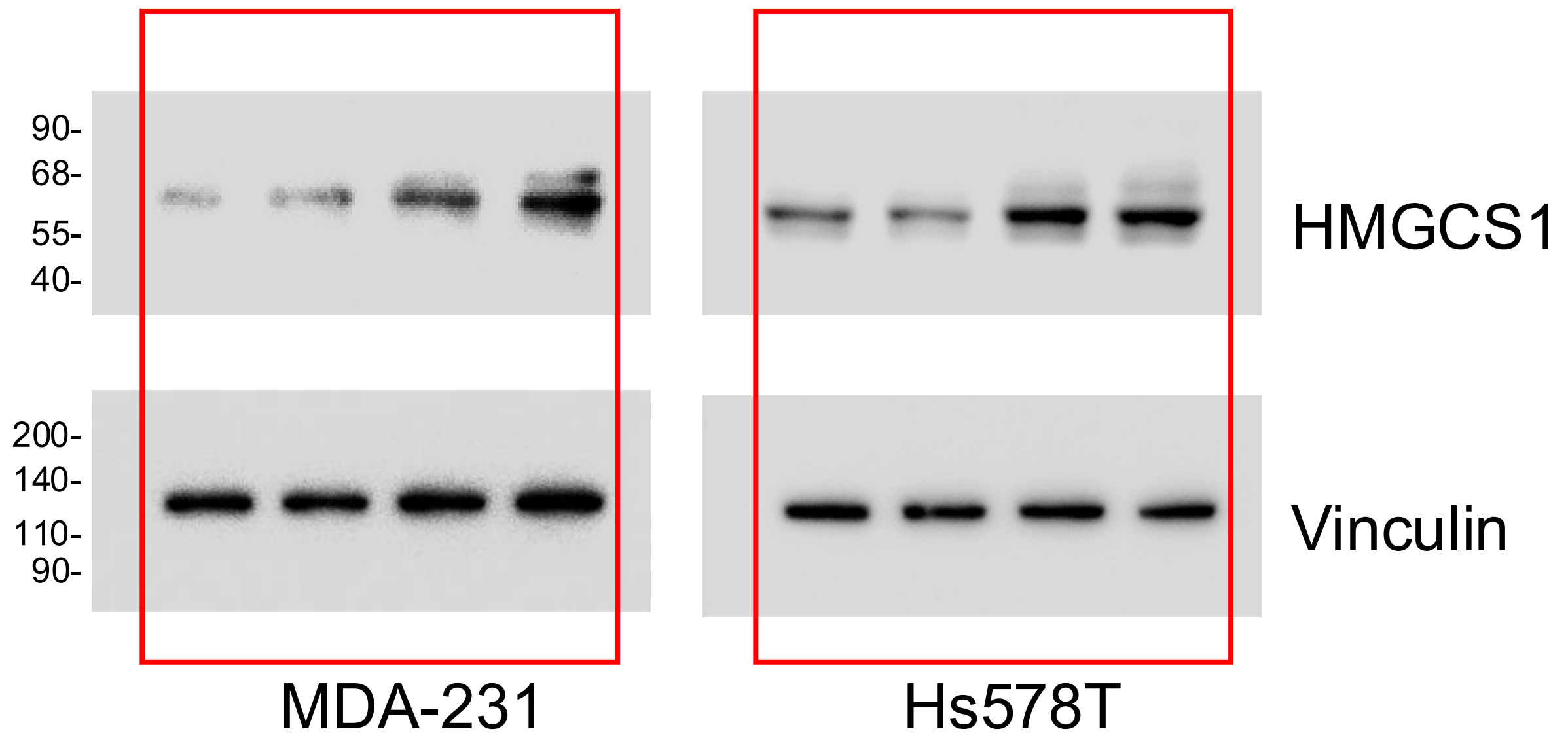

Figure 5K

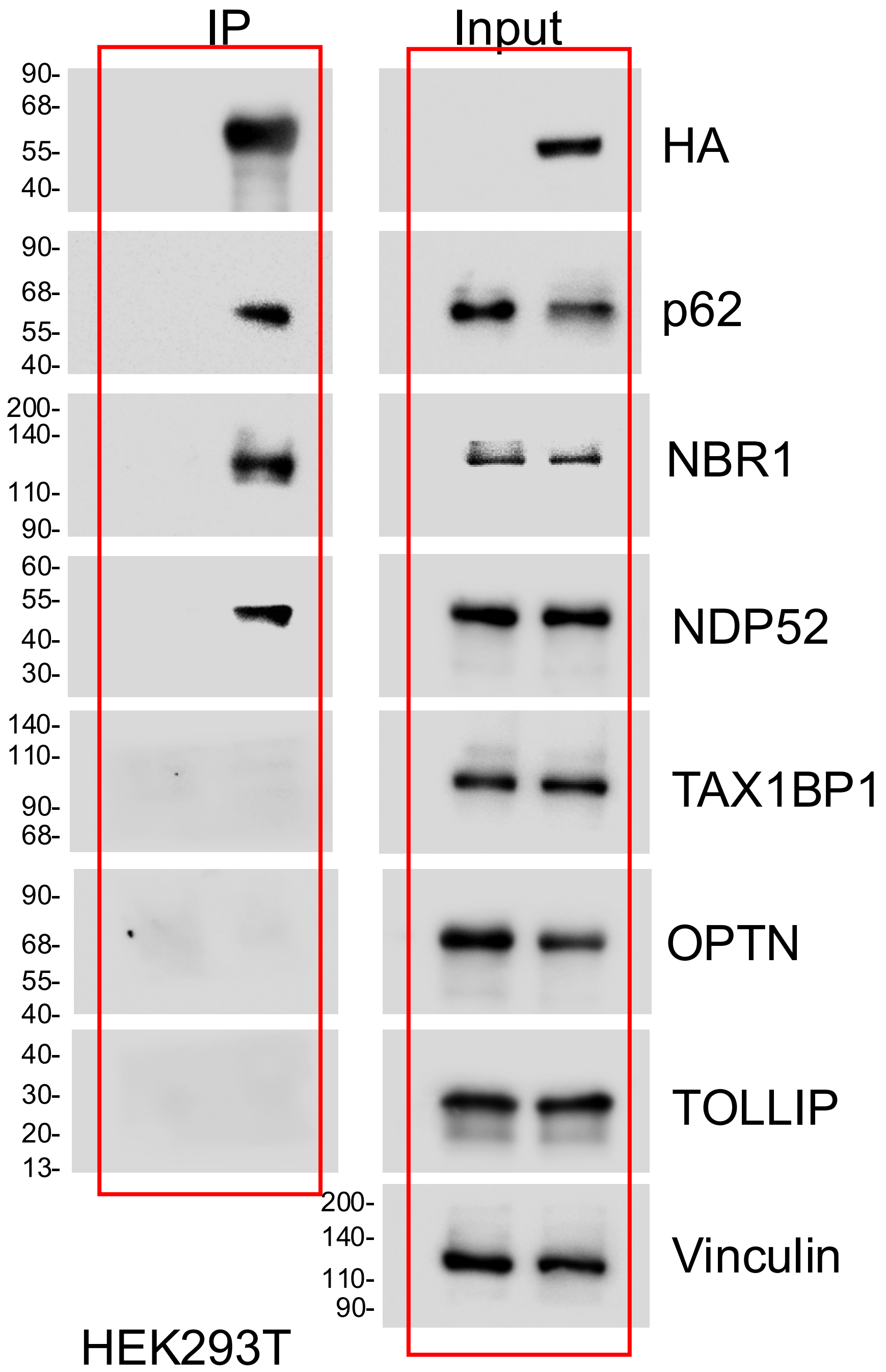

Figure 5L

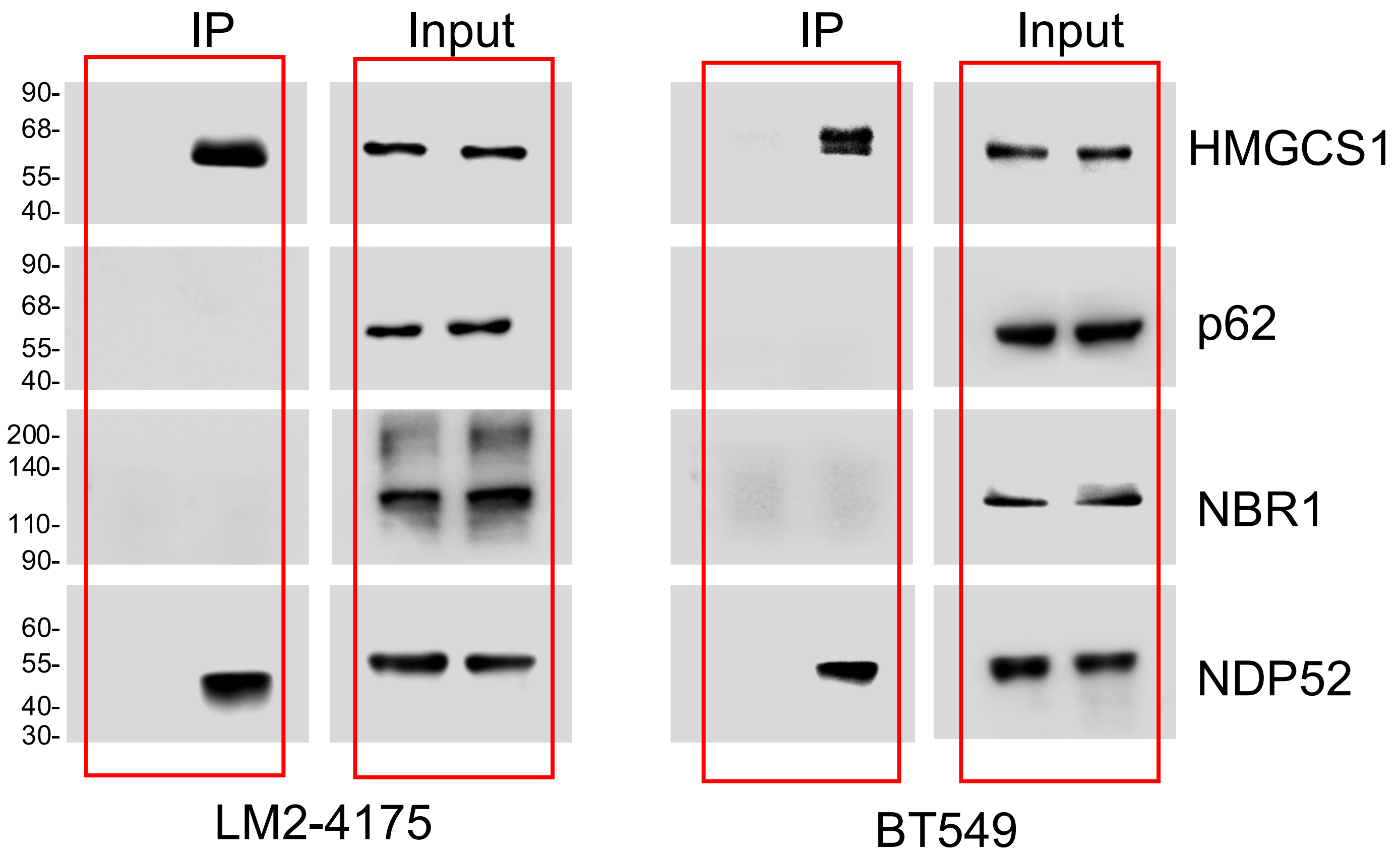

Figure 5M

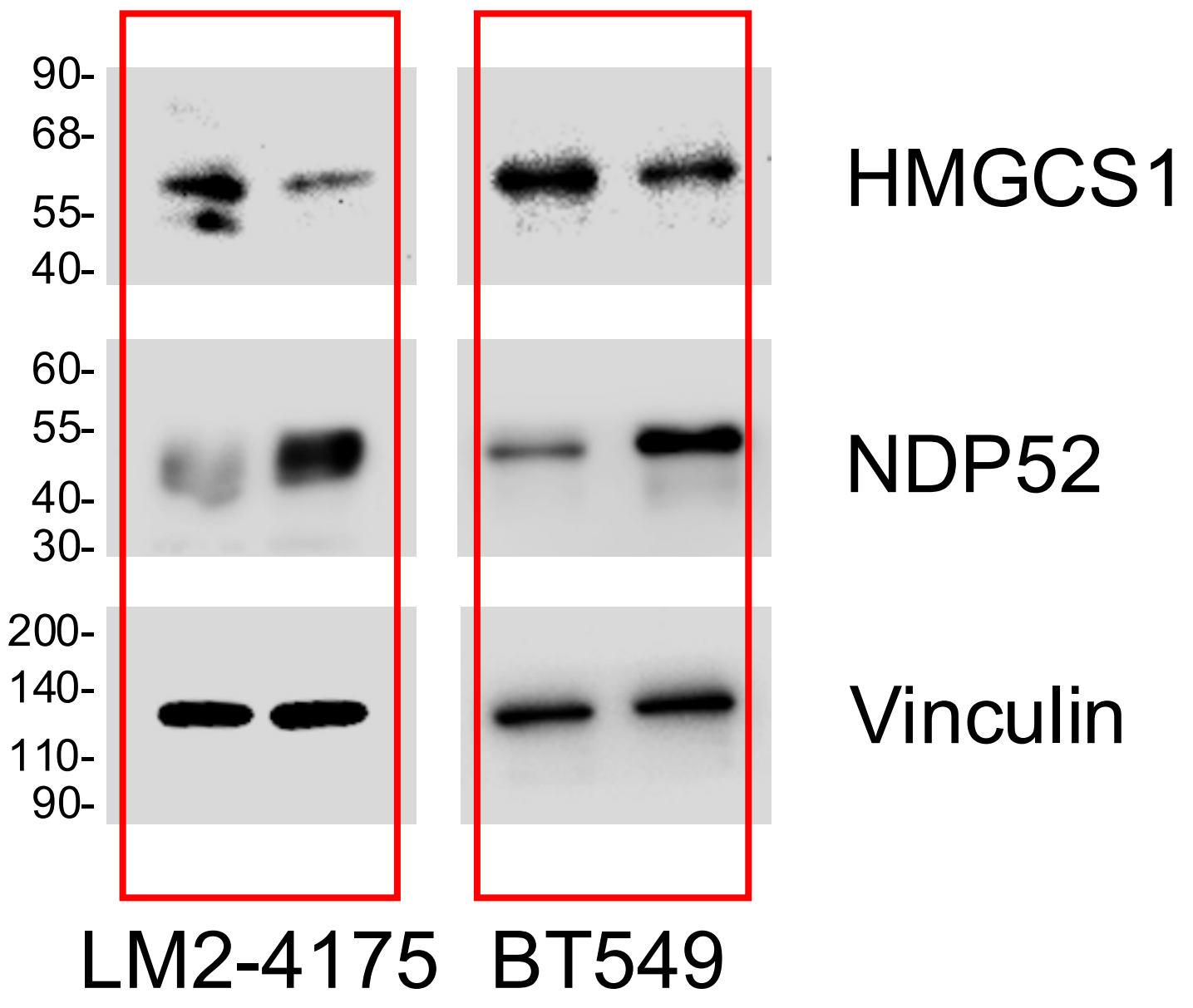

Figure 5N

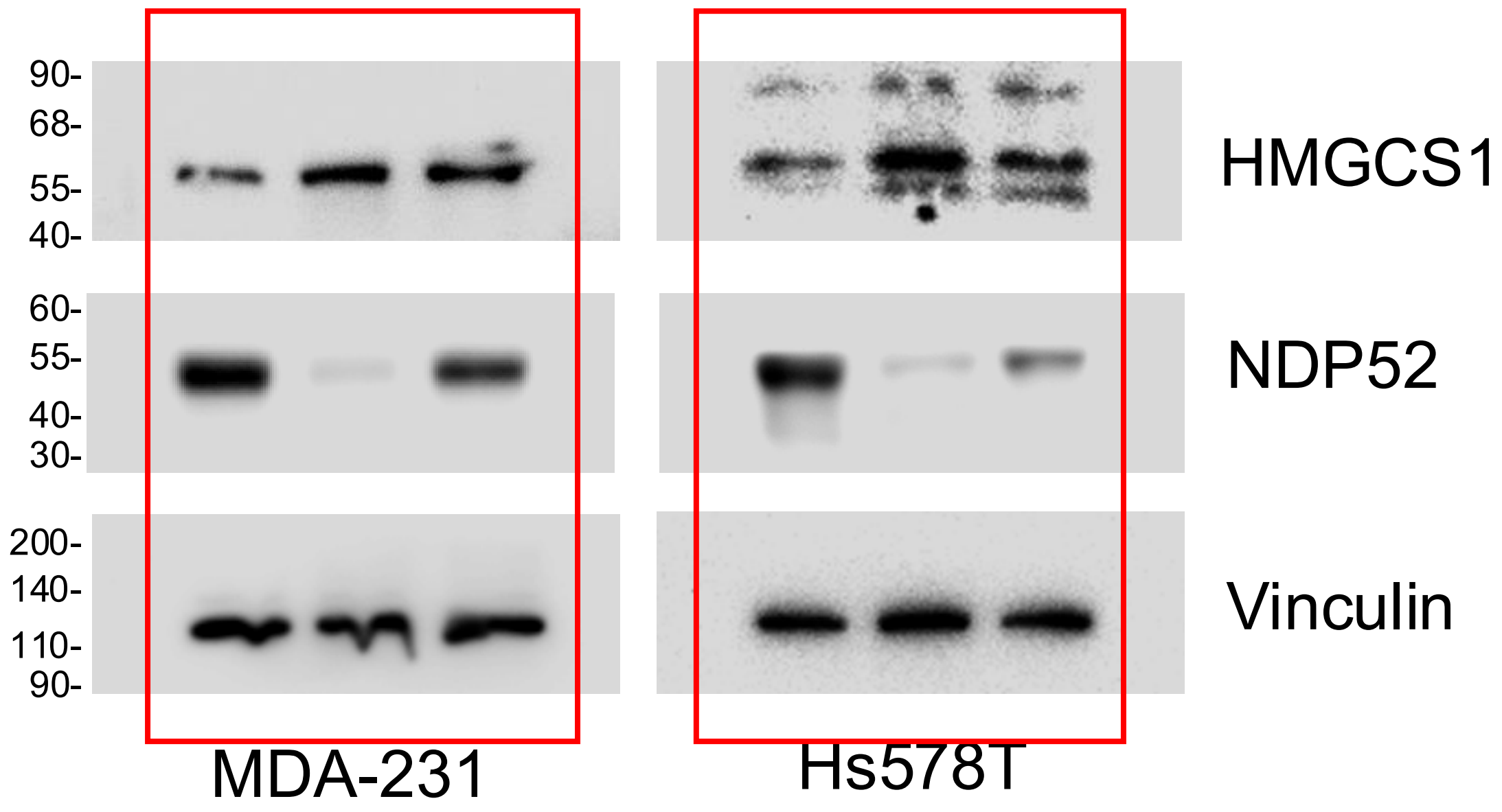

Figure 5O

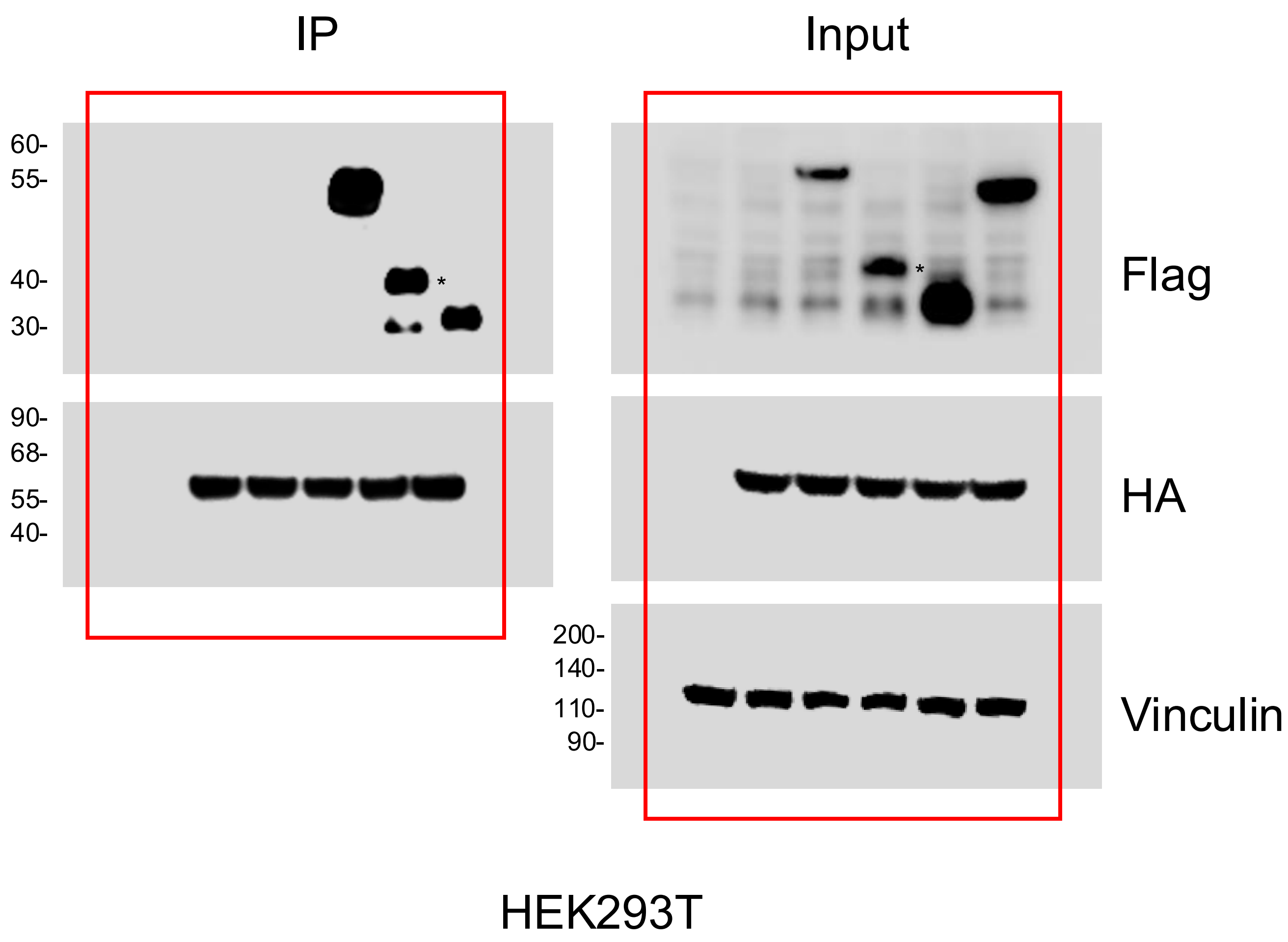

Figure 5P

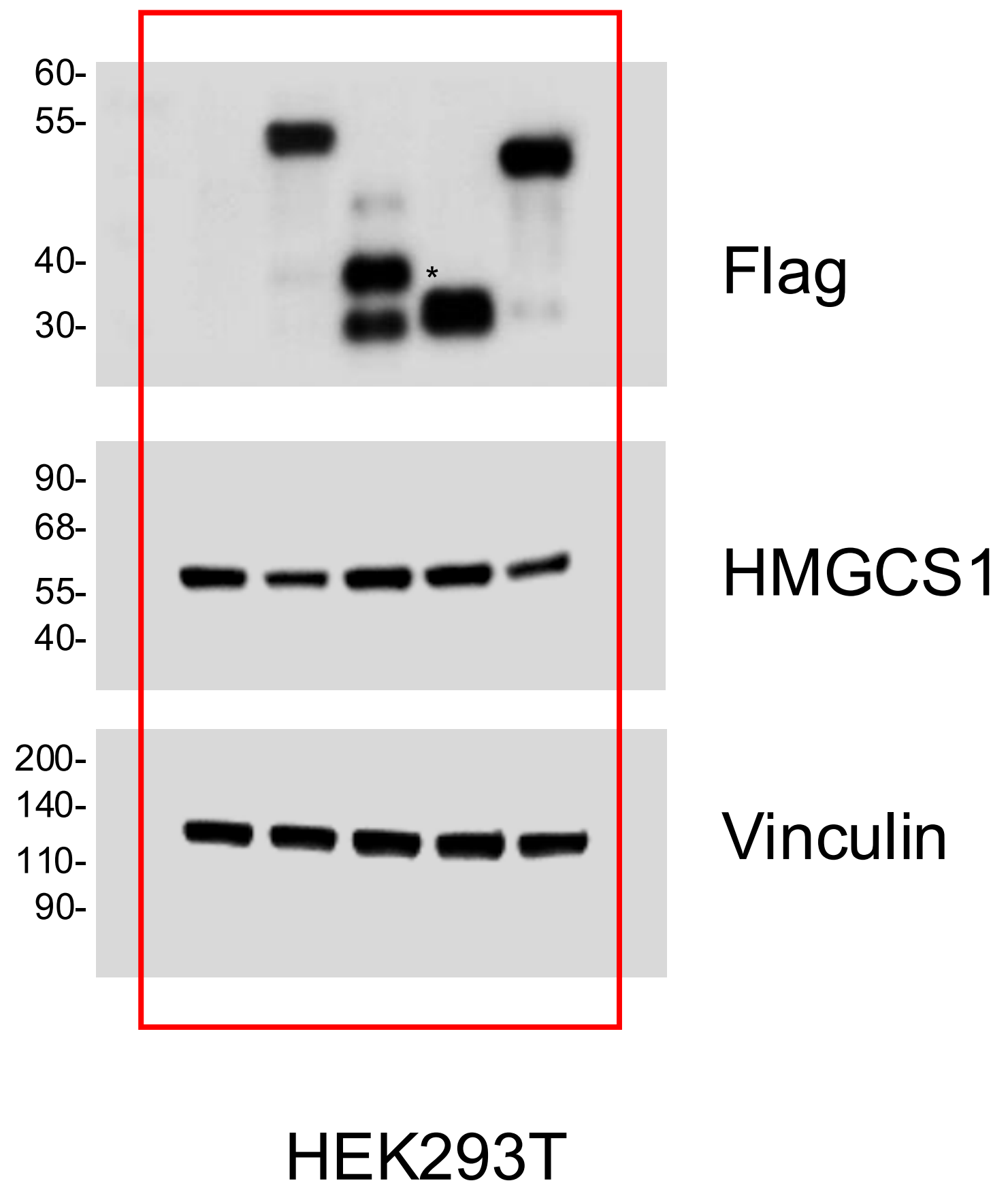

Figure 5Q

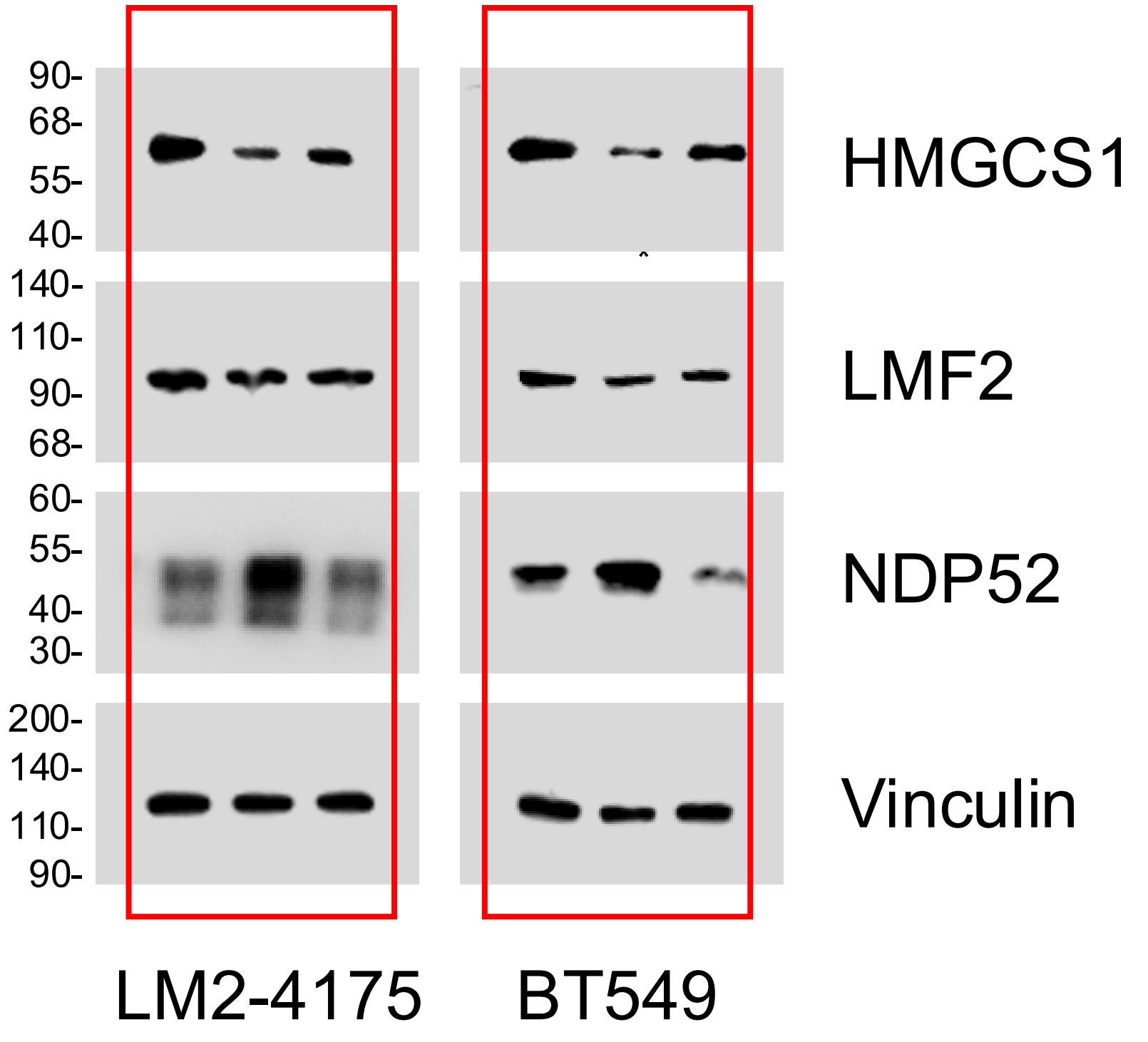

Figure 5R

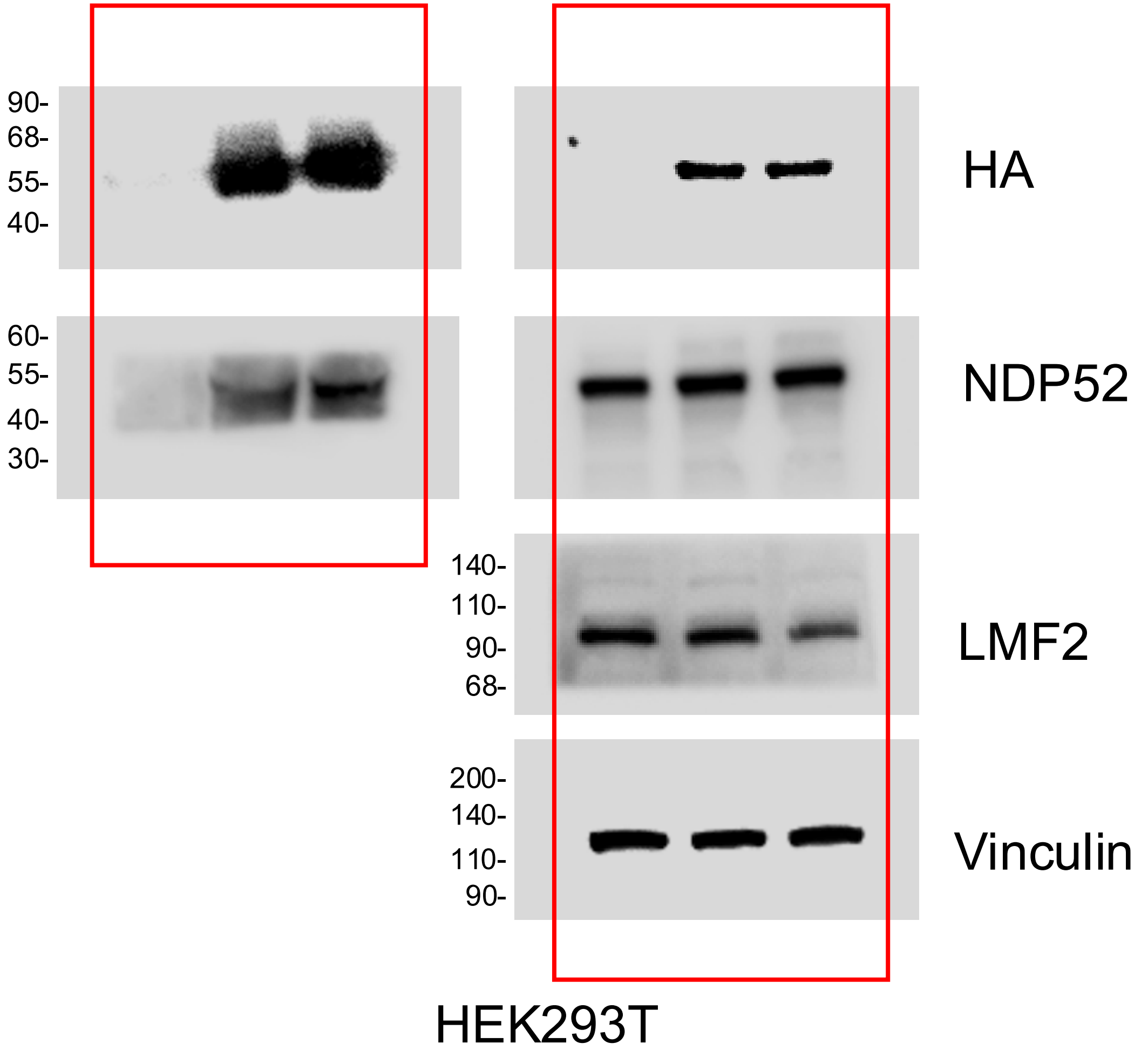

Figure 6A

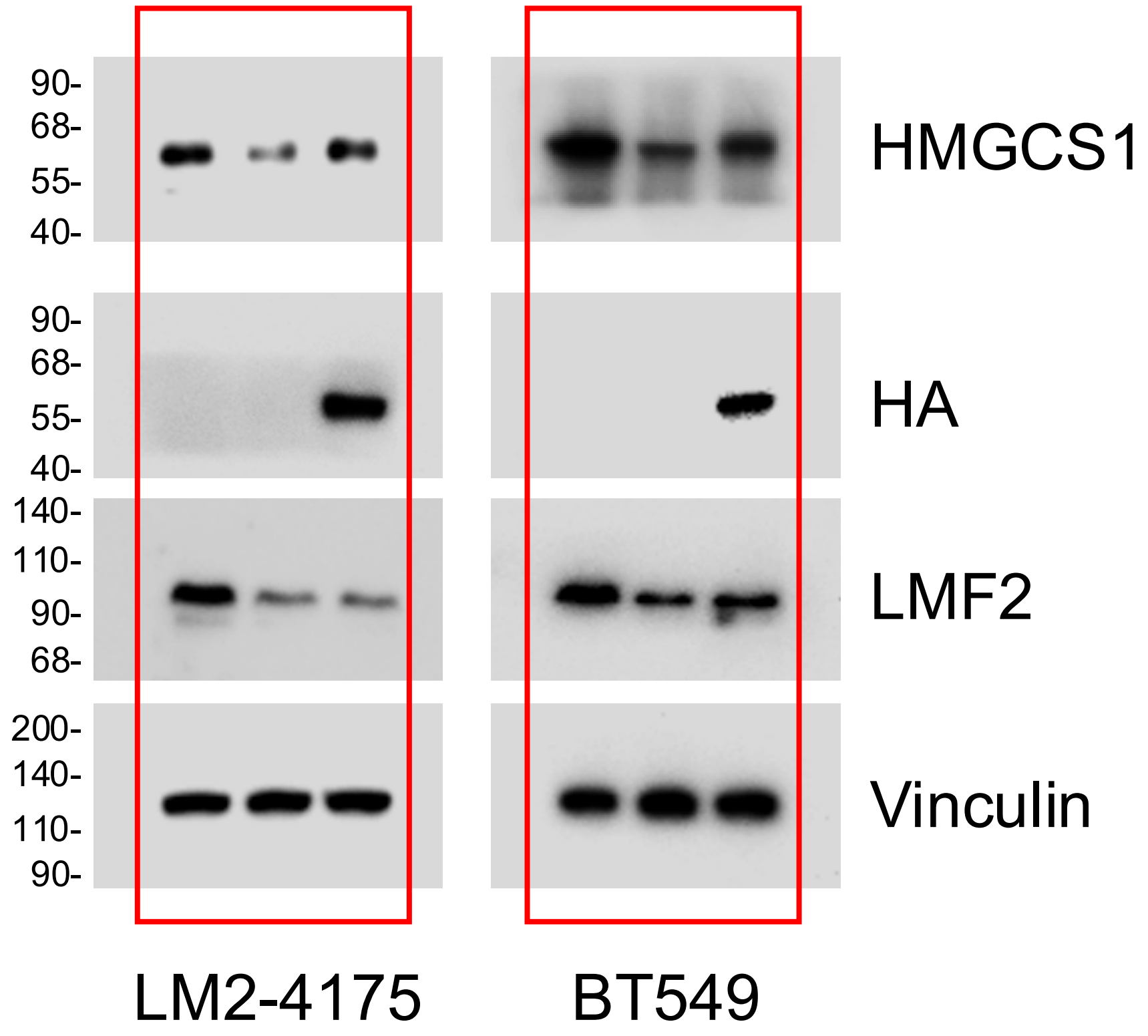

90-
68-
55-
40-
90-
68-
55-
40-
140-
110-
90-
68-
200-
140-
110-
90-
HMGCS1
HA
LMF2
Vinculin
LM2-4175
BT549

Figure 7A

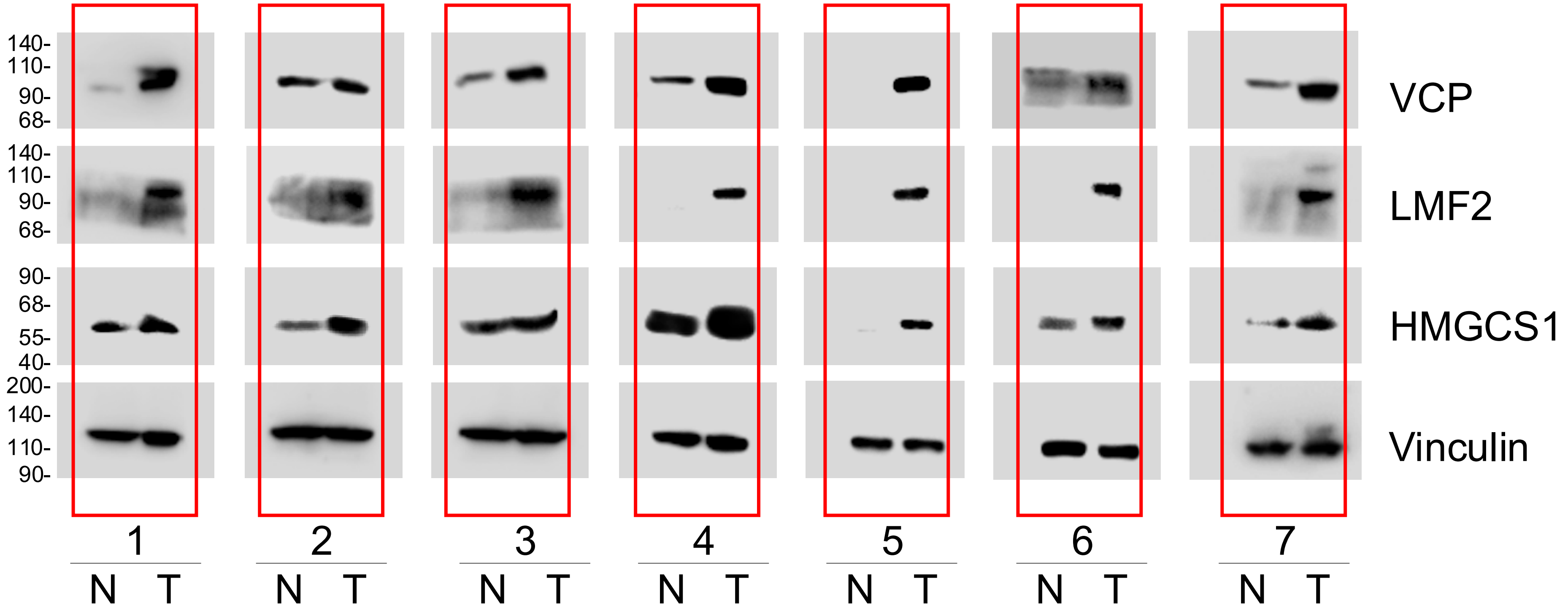
140-
110-
90-
68-
140-
110-
90-
68-
90-
68-
55-
40-
200-
140-
110-
90-
VCP
LMF2
HMGCS1
Vinculin
1
2
3
4
5
6
7
N T
N T
N T
N T
N T
N T
N T

Figure 7A

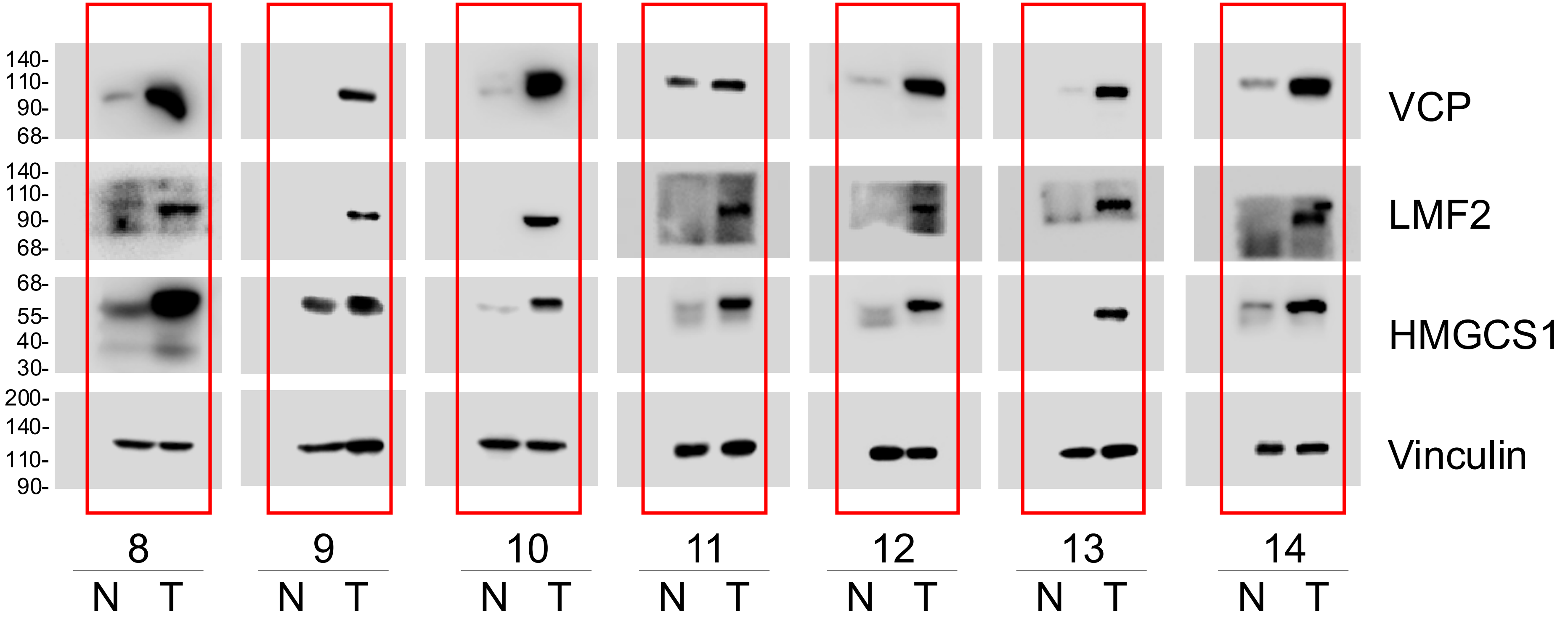

140-
110-
90-
68-
140-
110-
90-
68-
68-
55-
40-
30-
200-
140-
110-
90-
VCP
LMF2
HMGCS1
Vinculin
8
9
10
11
12
13
14
N T
N T
N T
N T
N T
N T
N T

Figure S2A

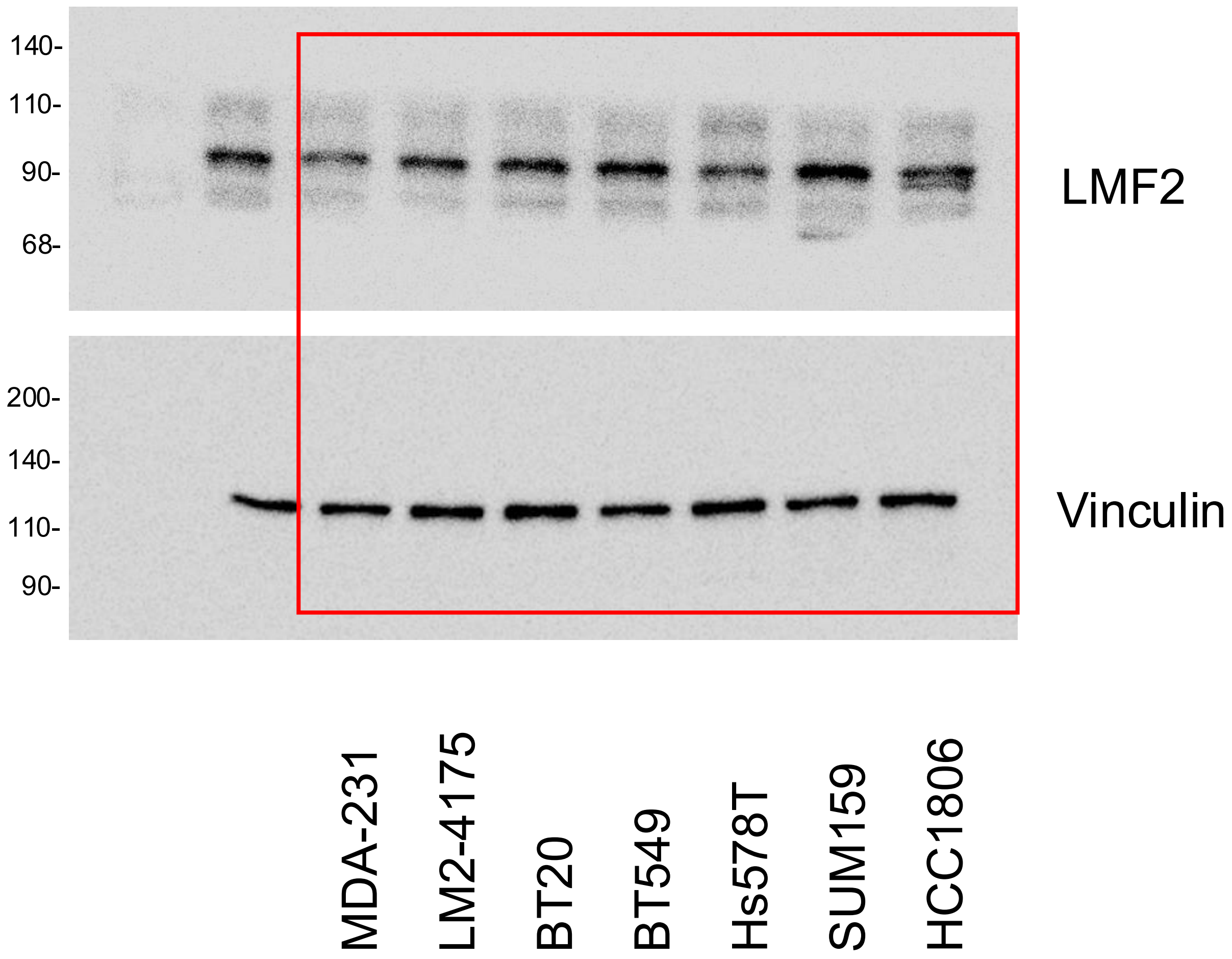
140-
110-
90-
68-
LMF2
200-
140-
110-
90-
Vinculin
MDA-231
LM2-4175
BT20
BT549
Hs578T
SUM159
HCC1806

Figure S3A

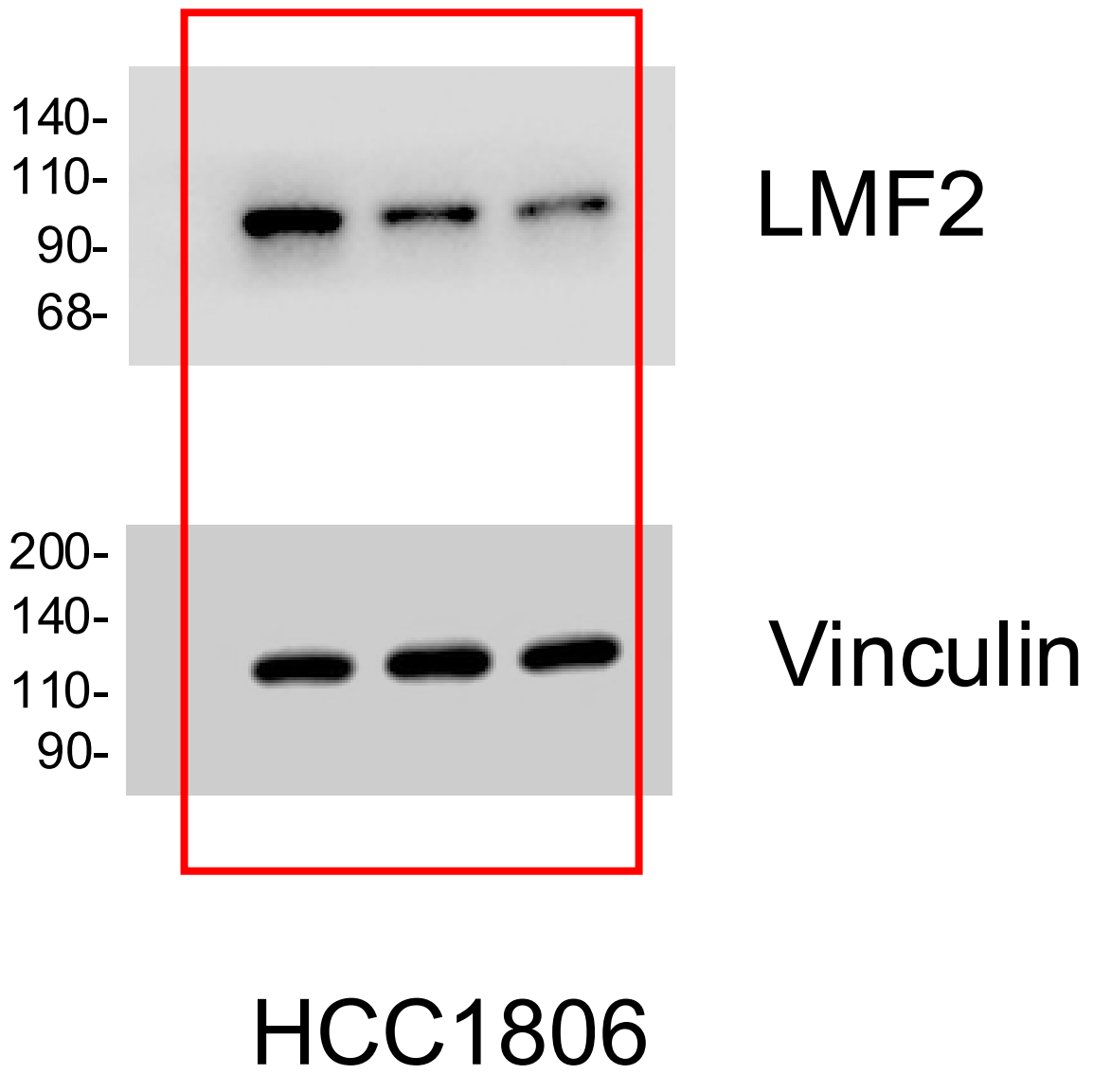

Figure S5A

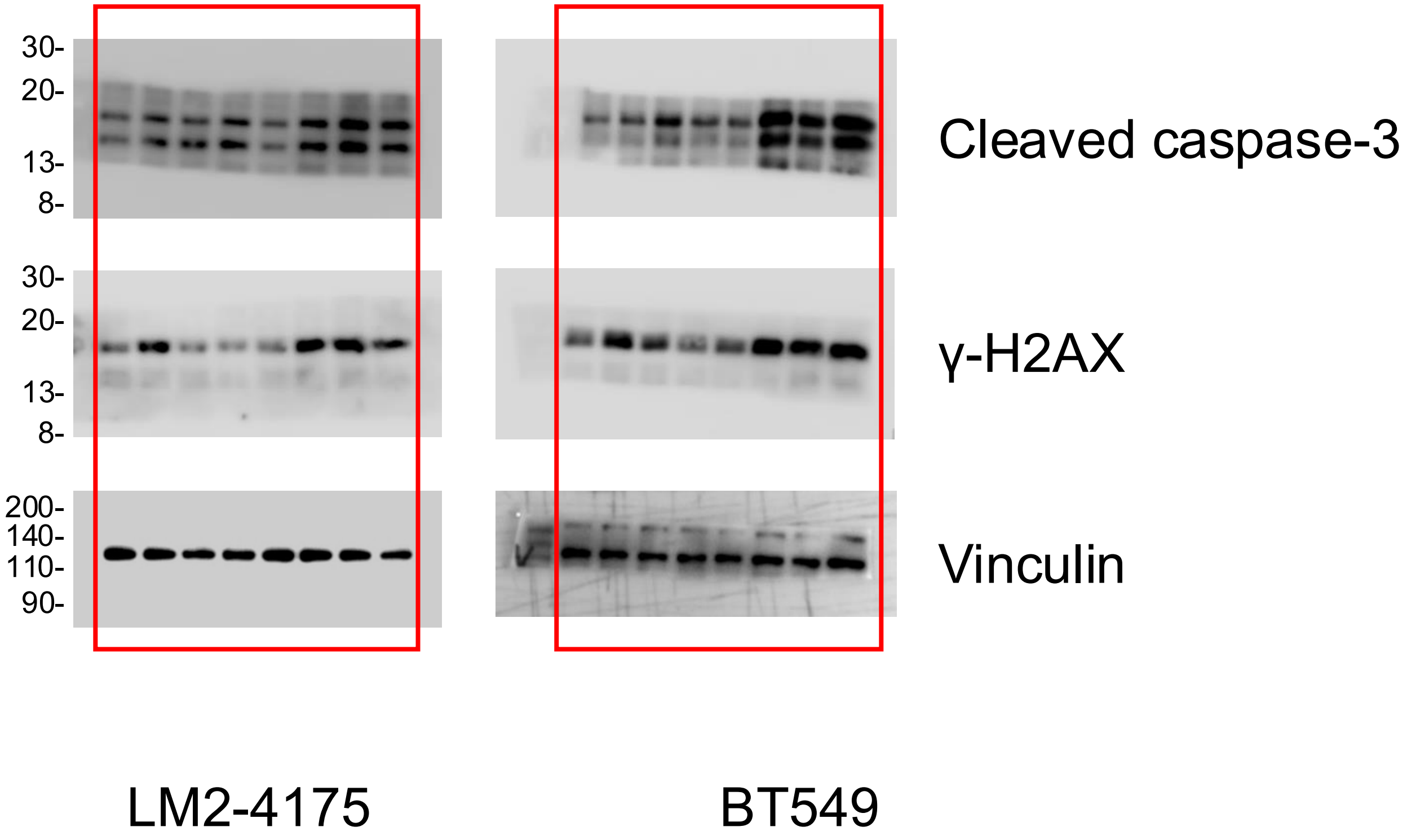

Figure S7A

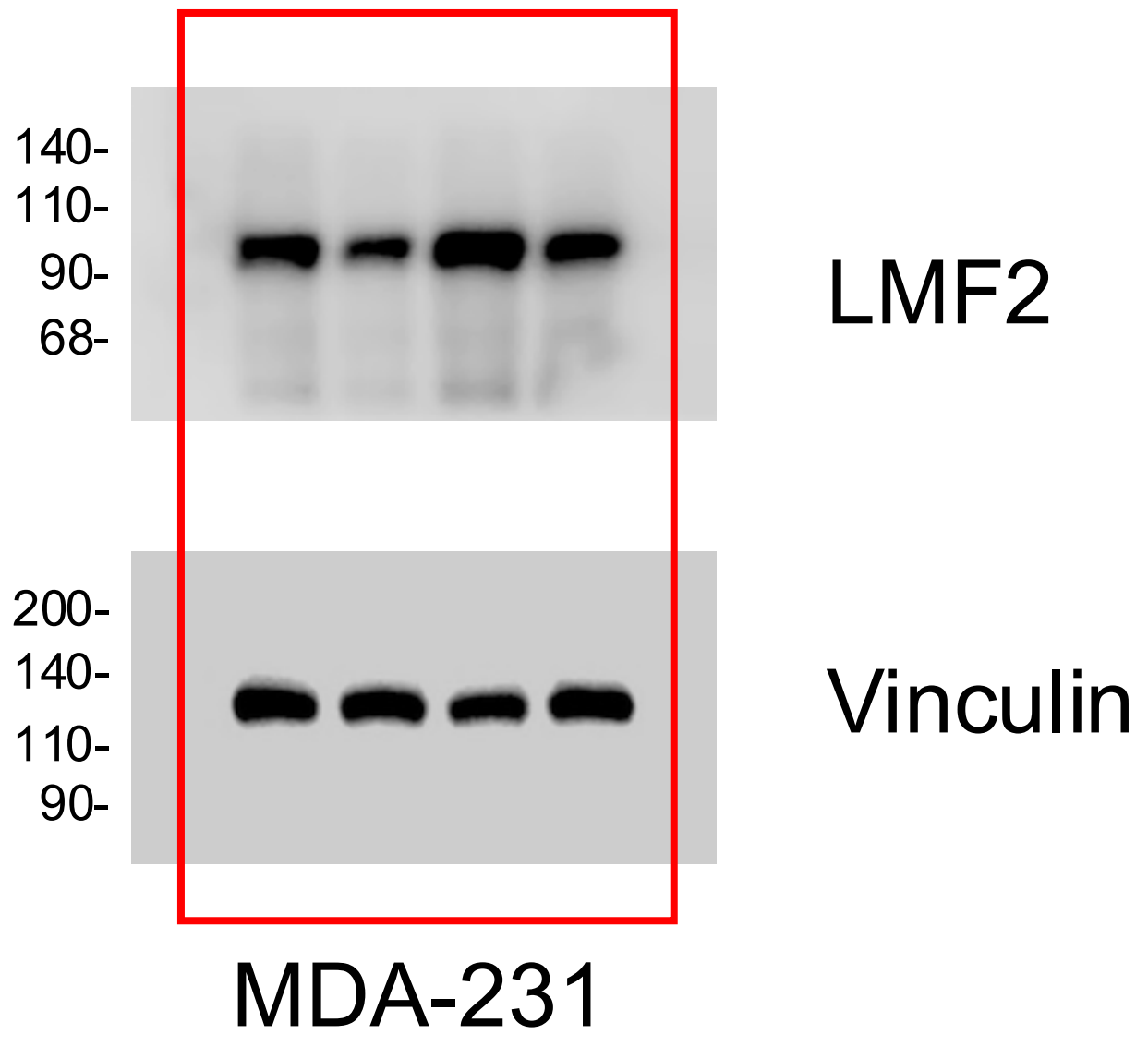

Figure S7B

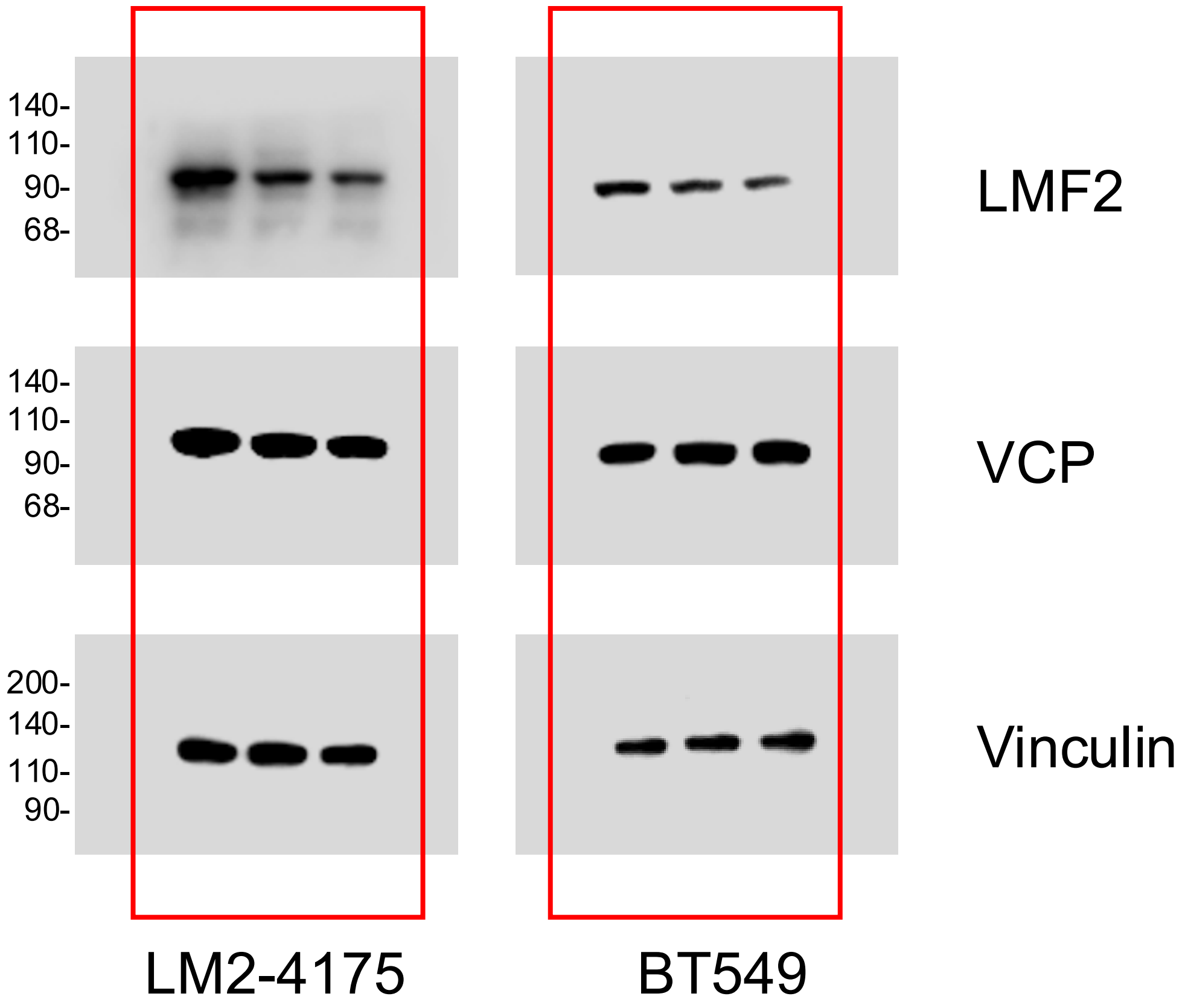

Figure S7F

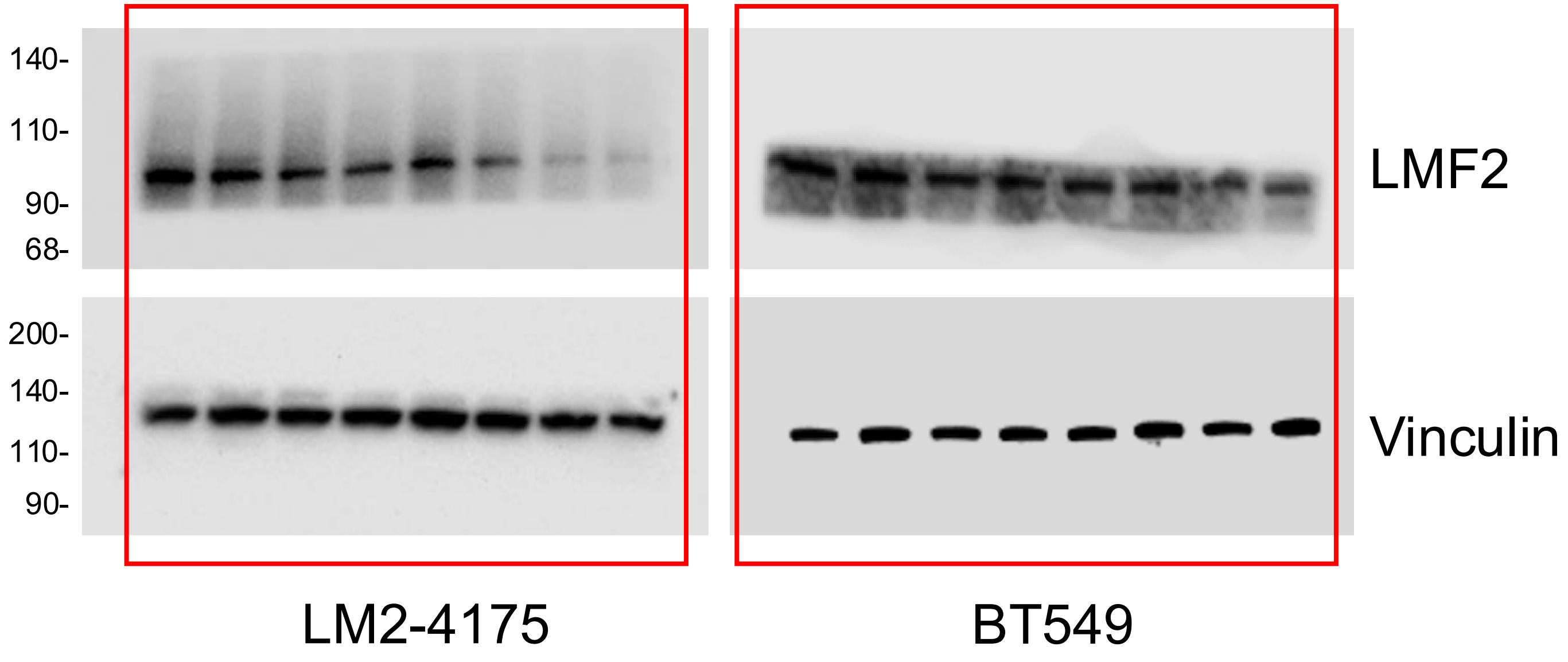

Figure S7G

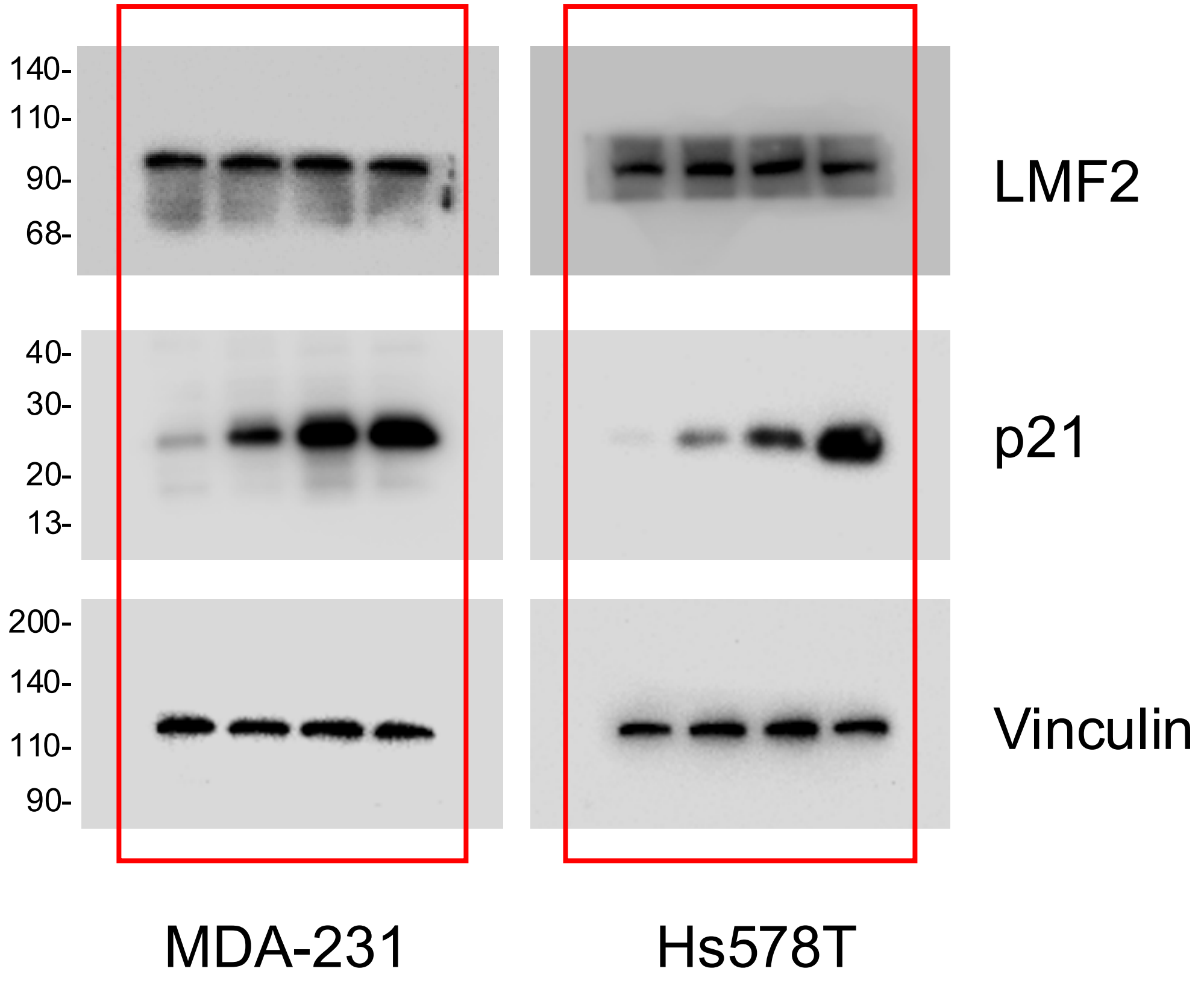

Figure S7H

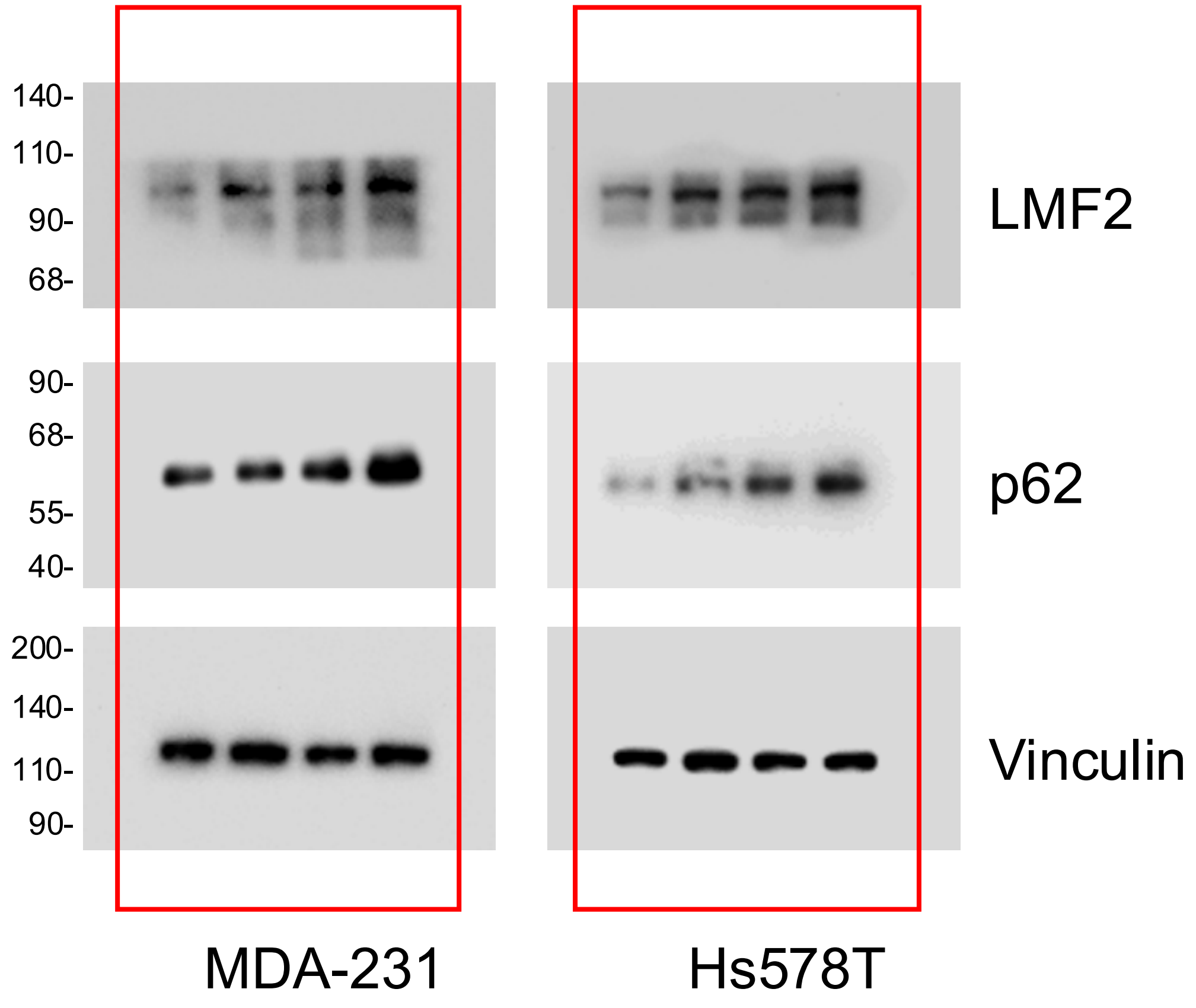

Figure S7I

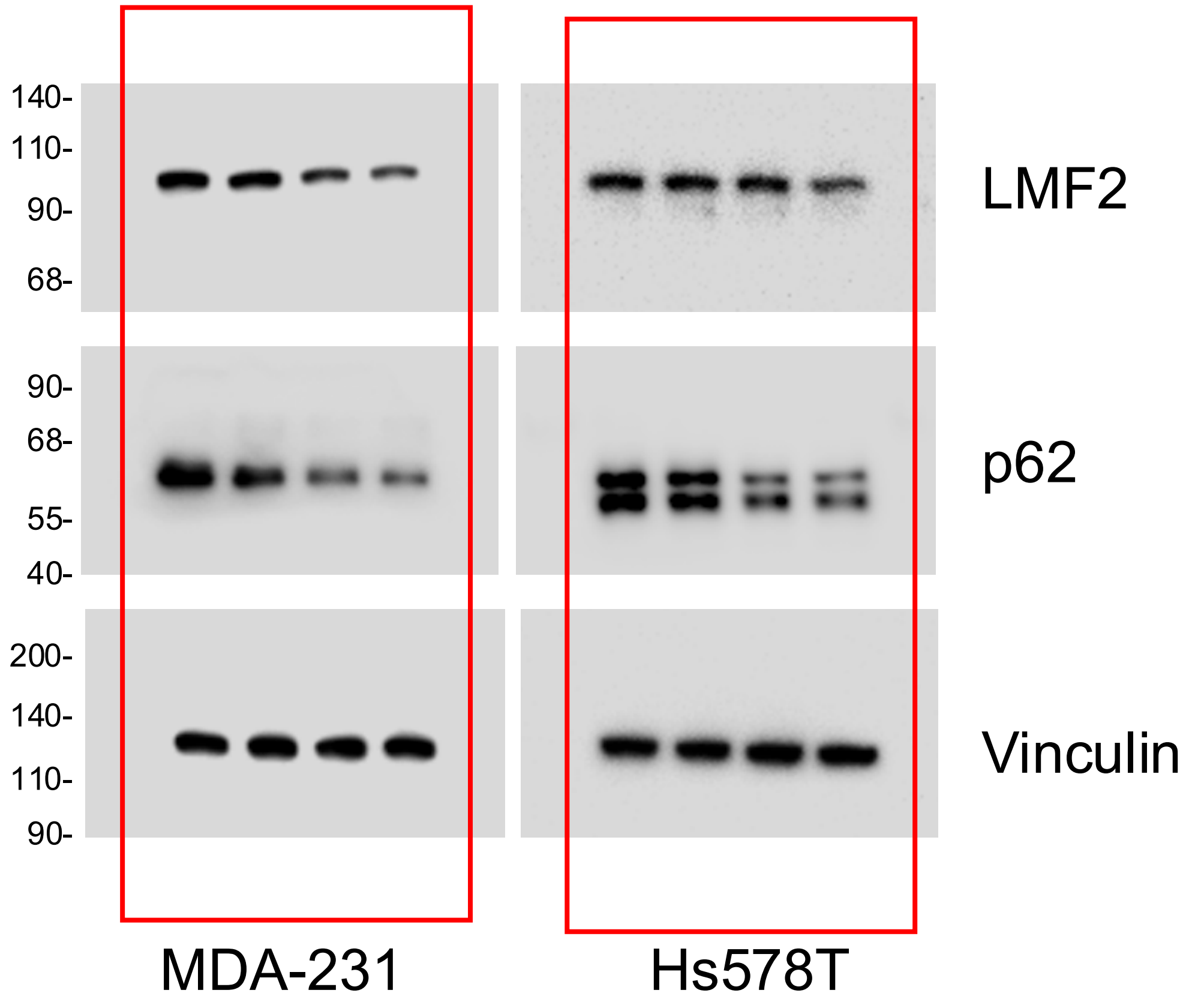

Figure S8A

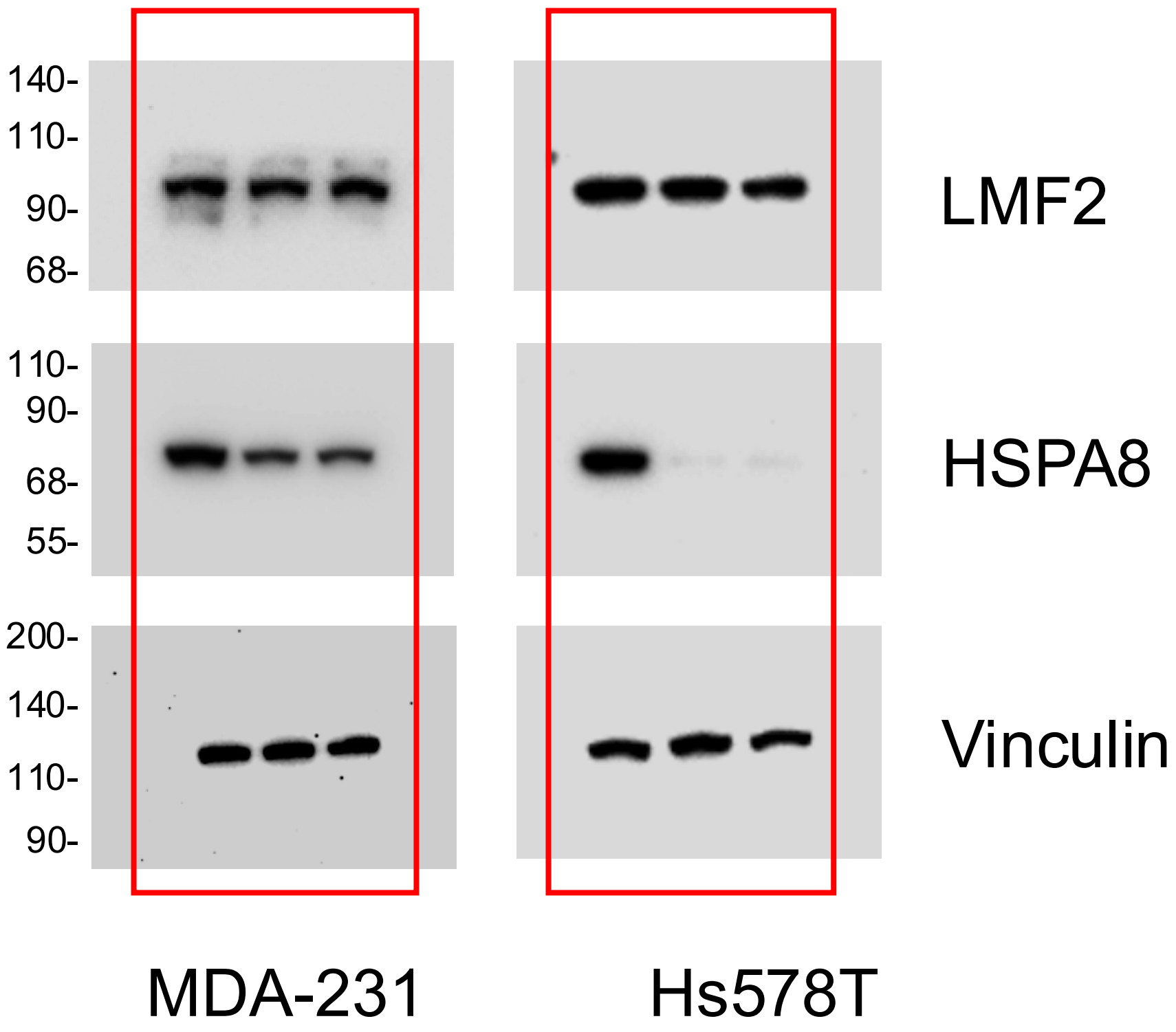

Figure S8B

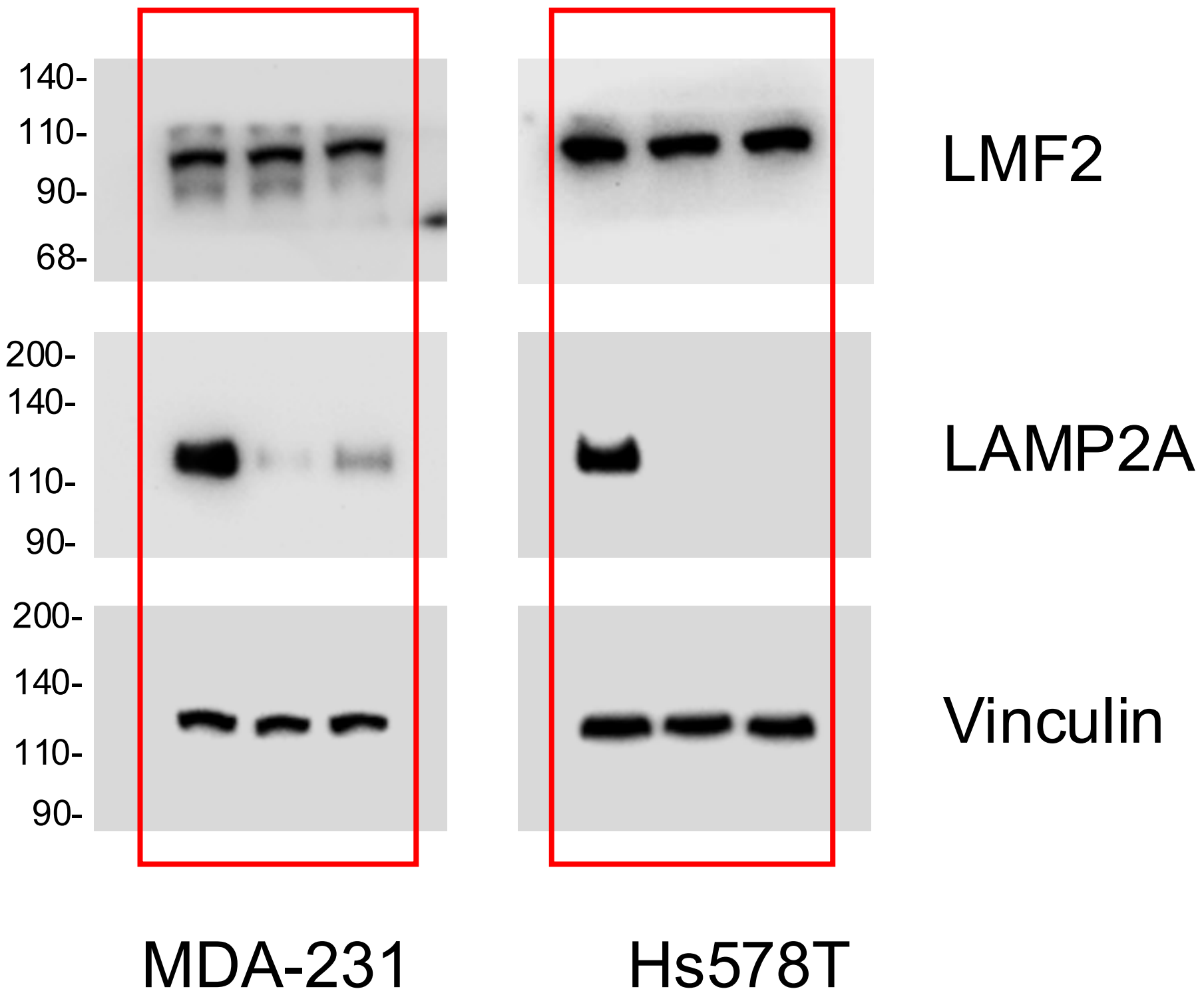

Figure S8C

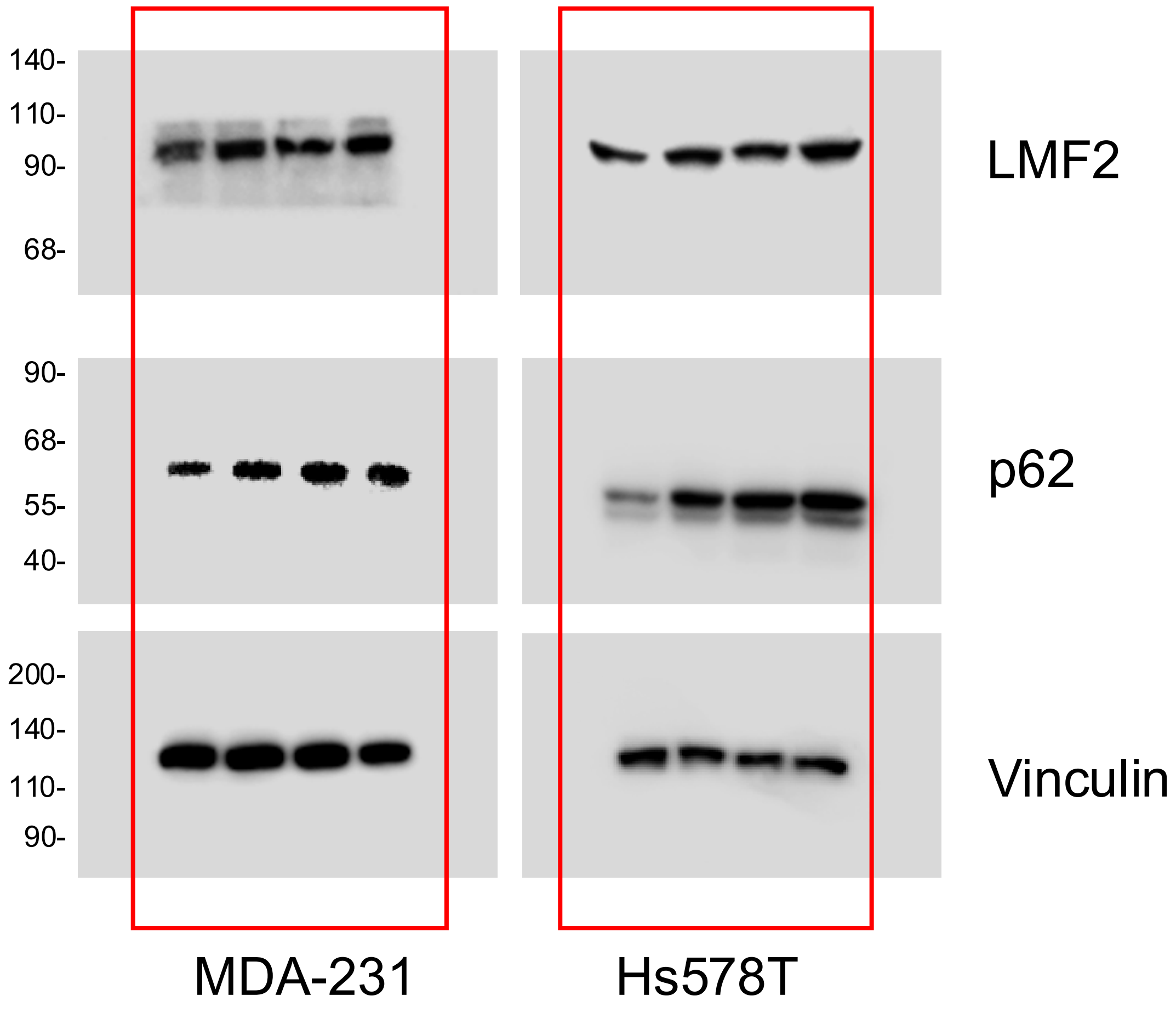

Figure S8D

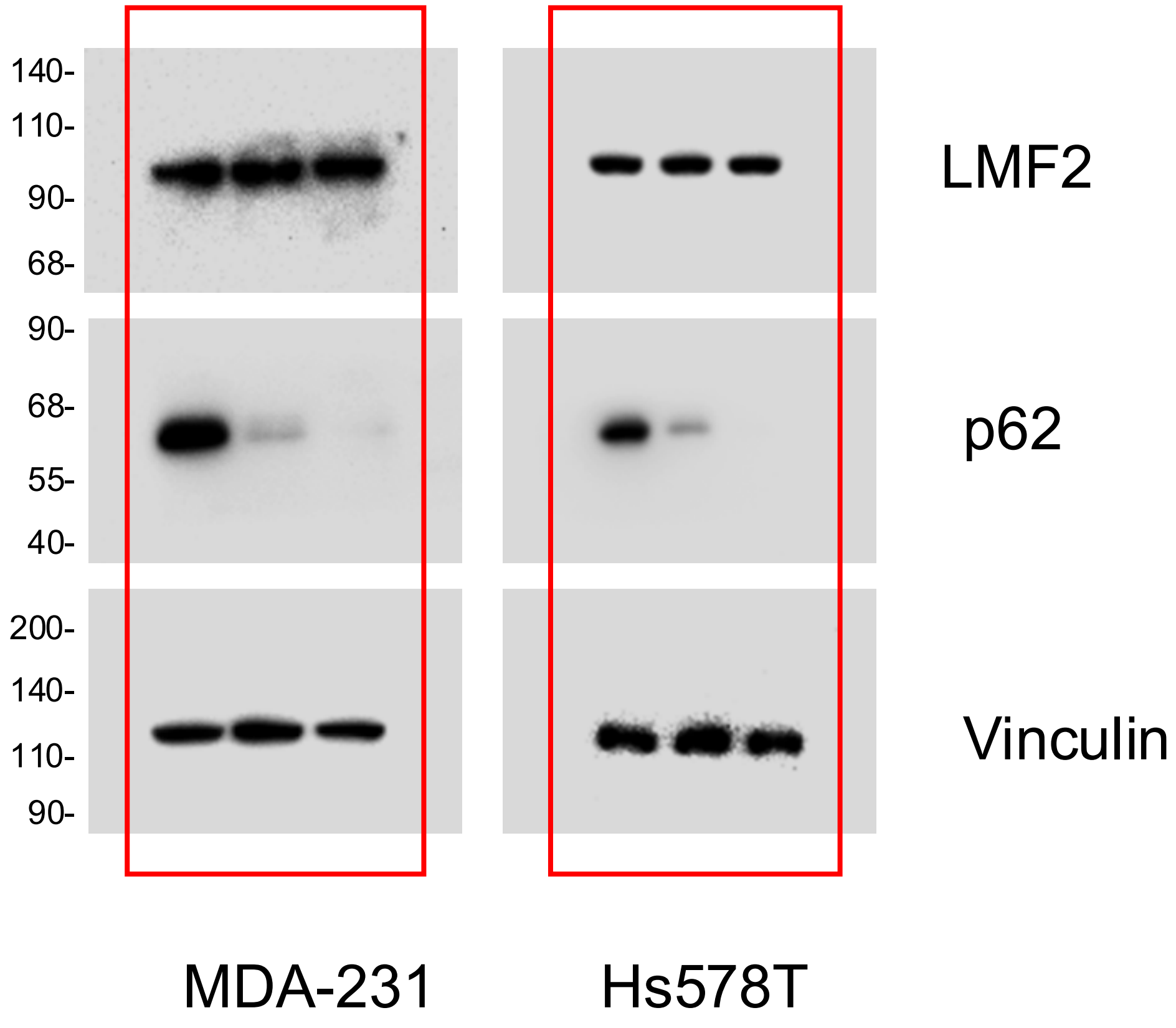

Figure S8E

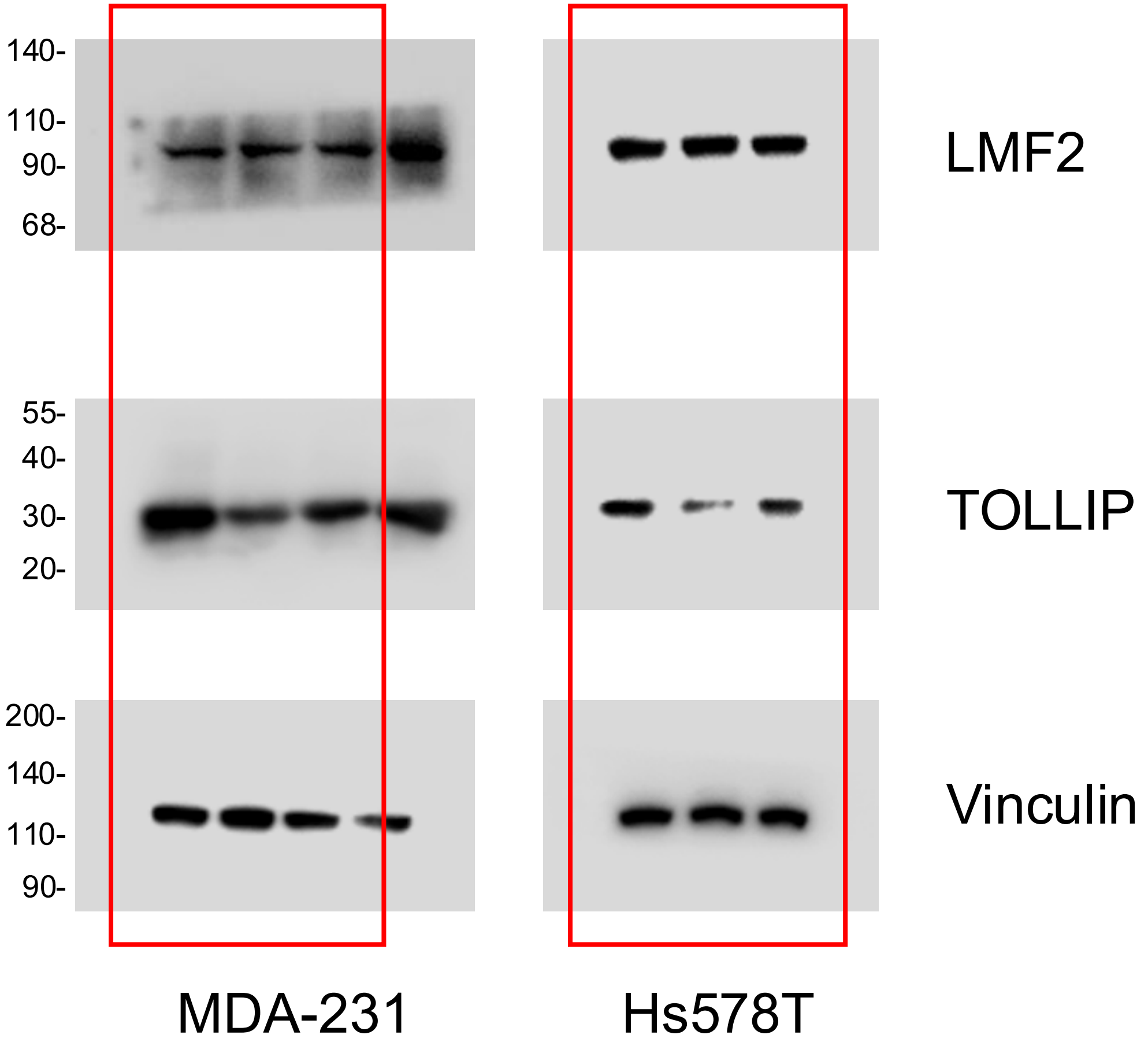

Figure S8G

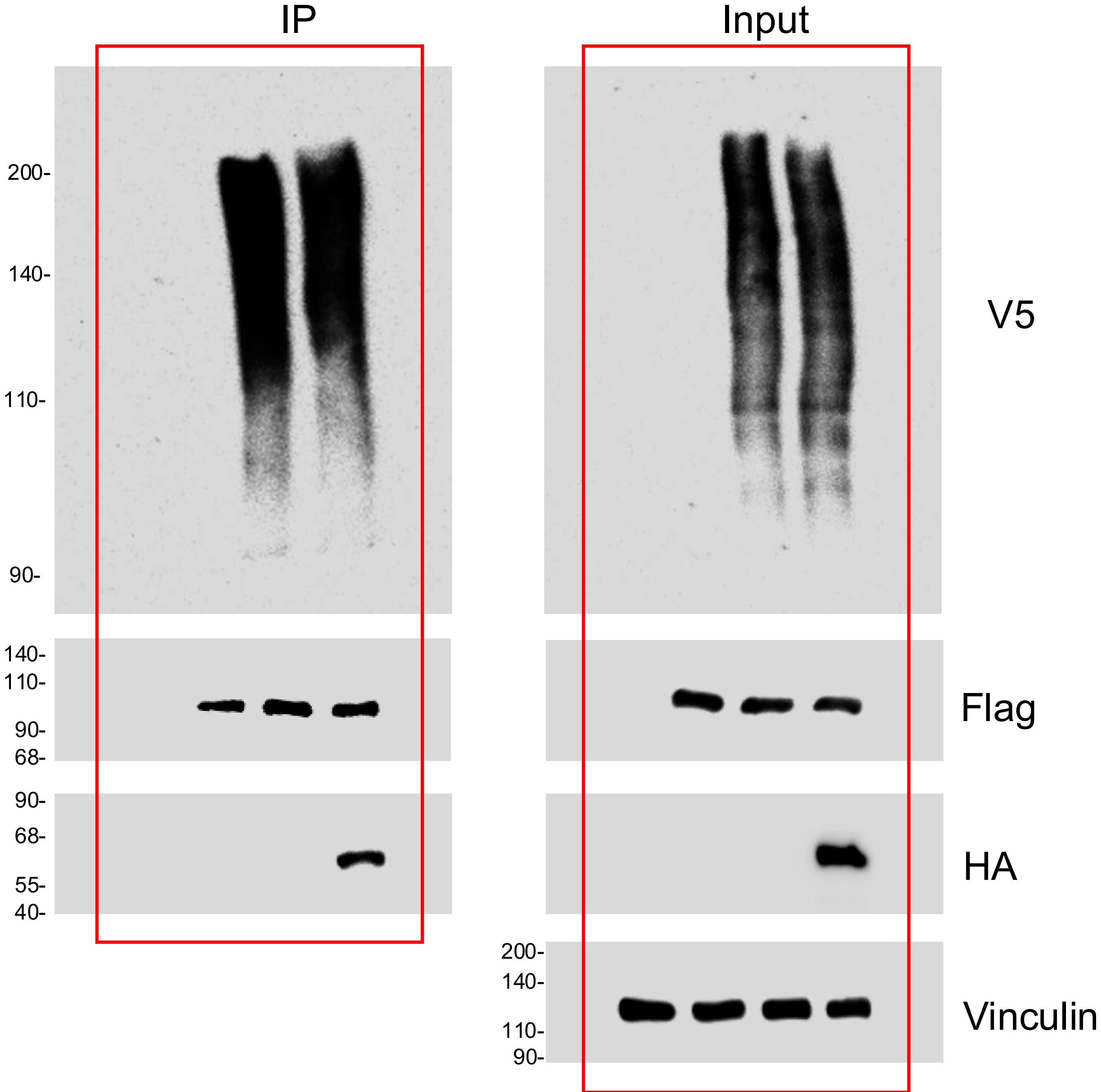

Figure S9A

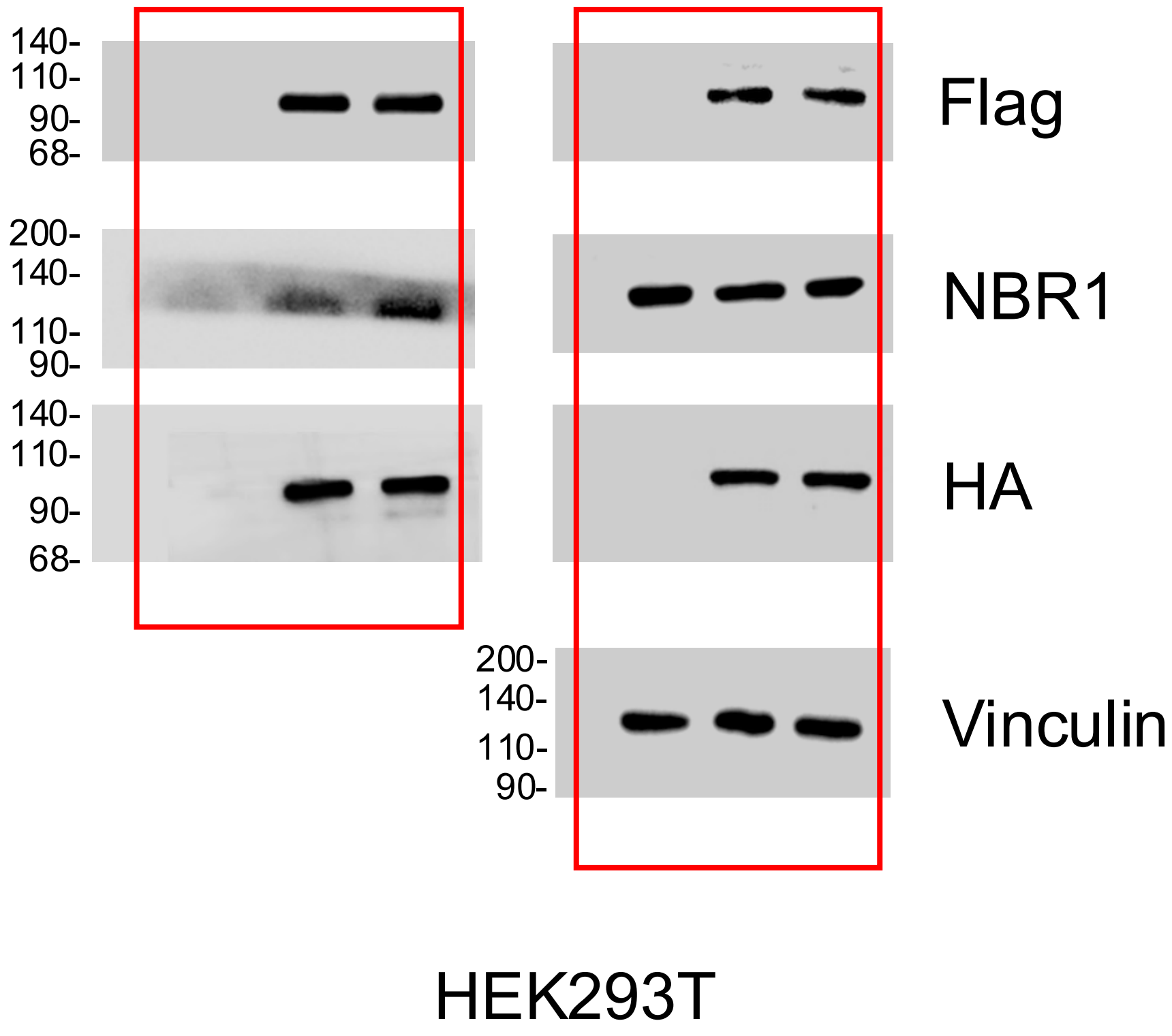

Figure S9C

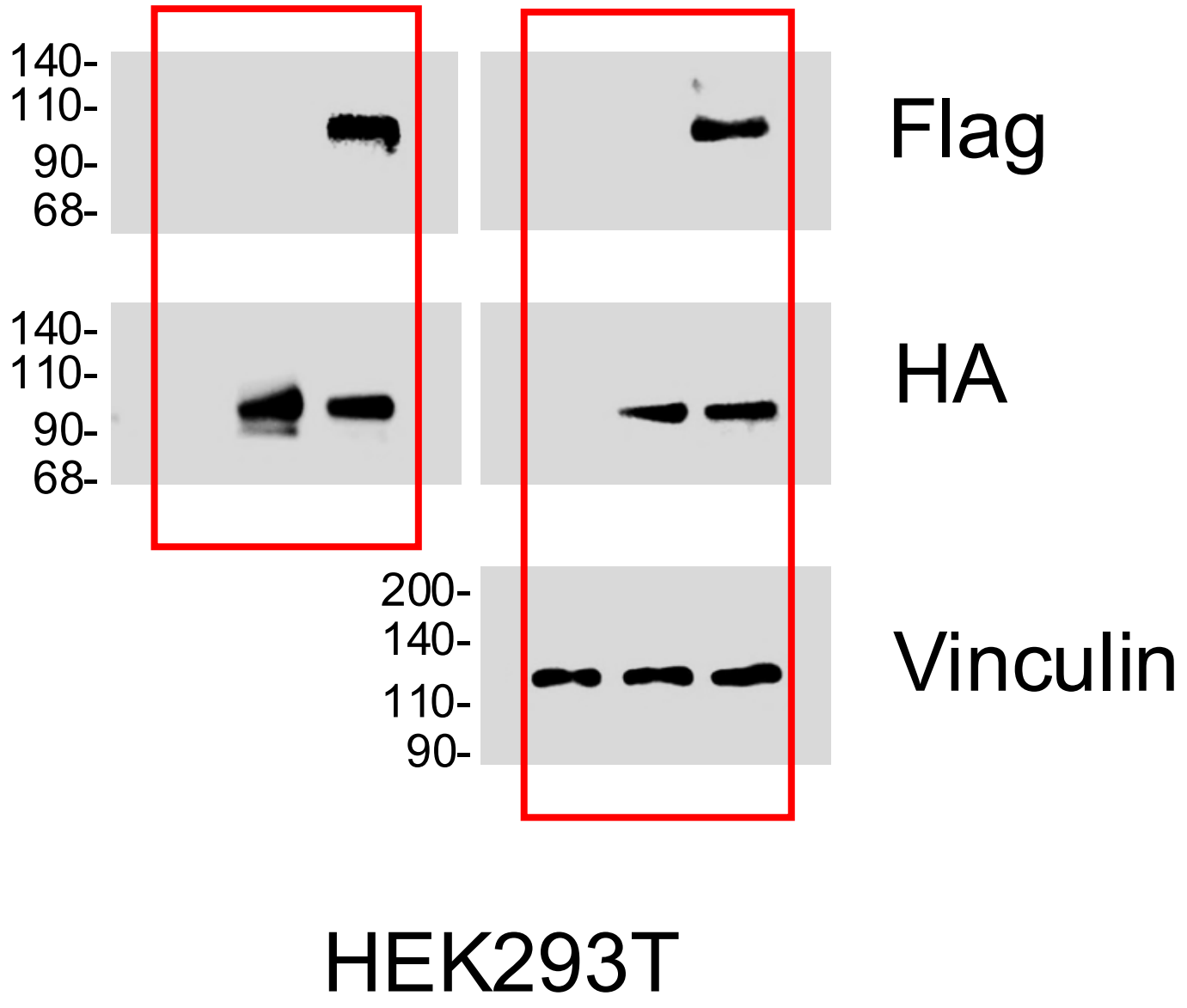

Figure S9D

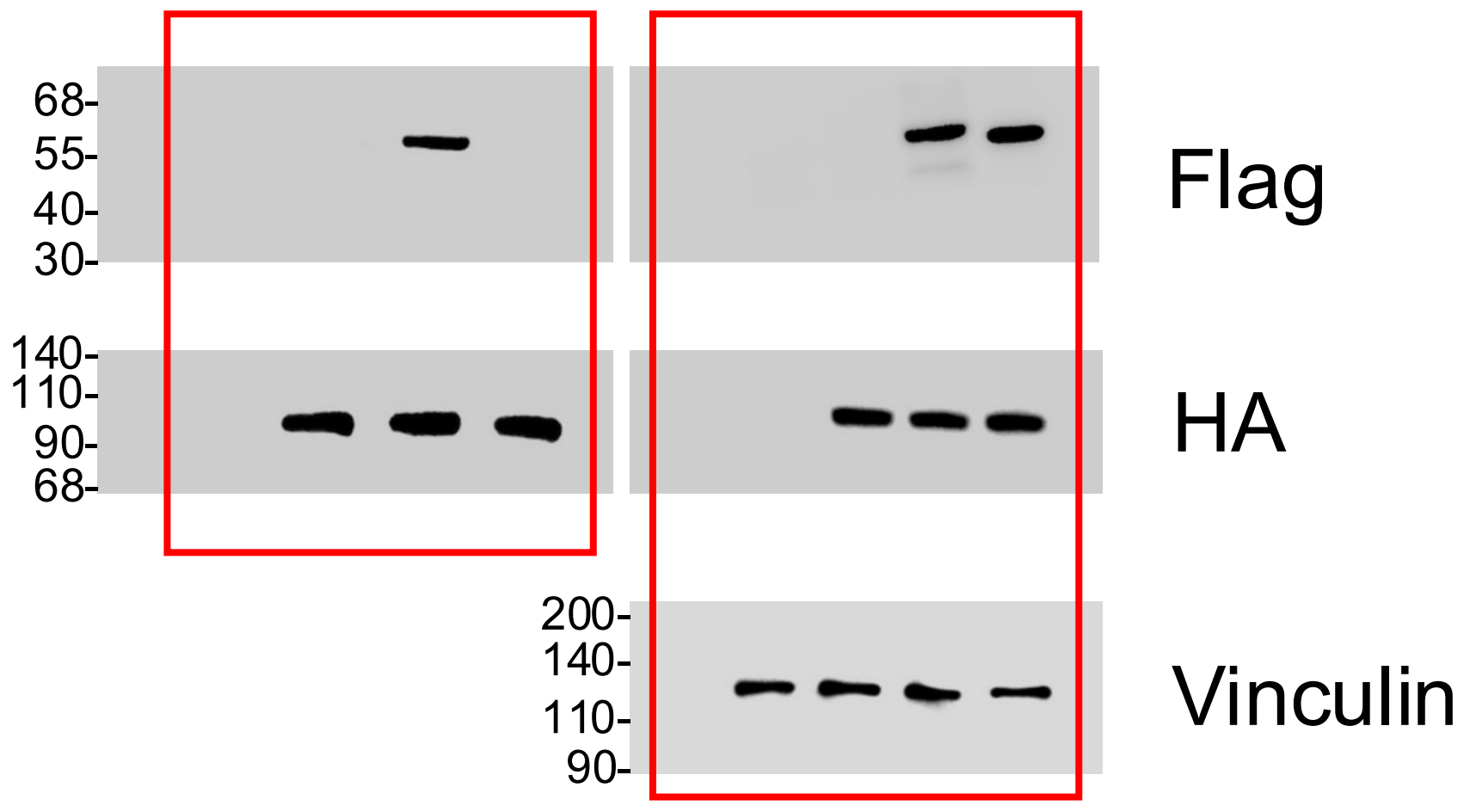

HEK293T

Figure S9E

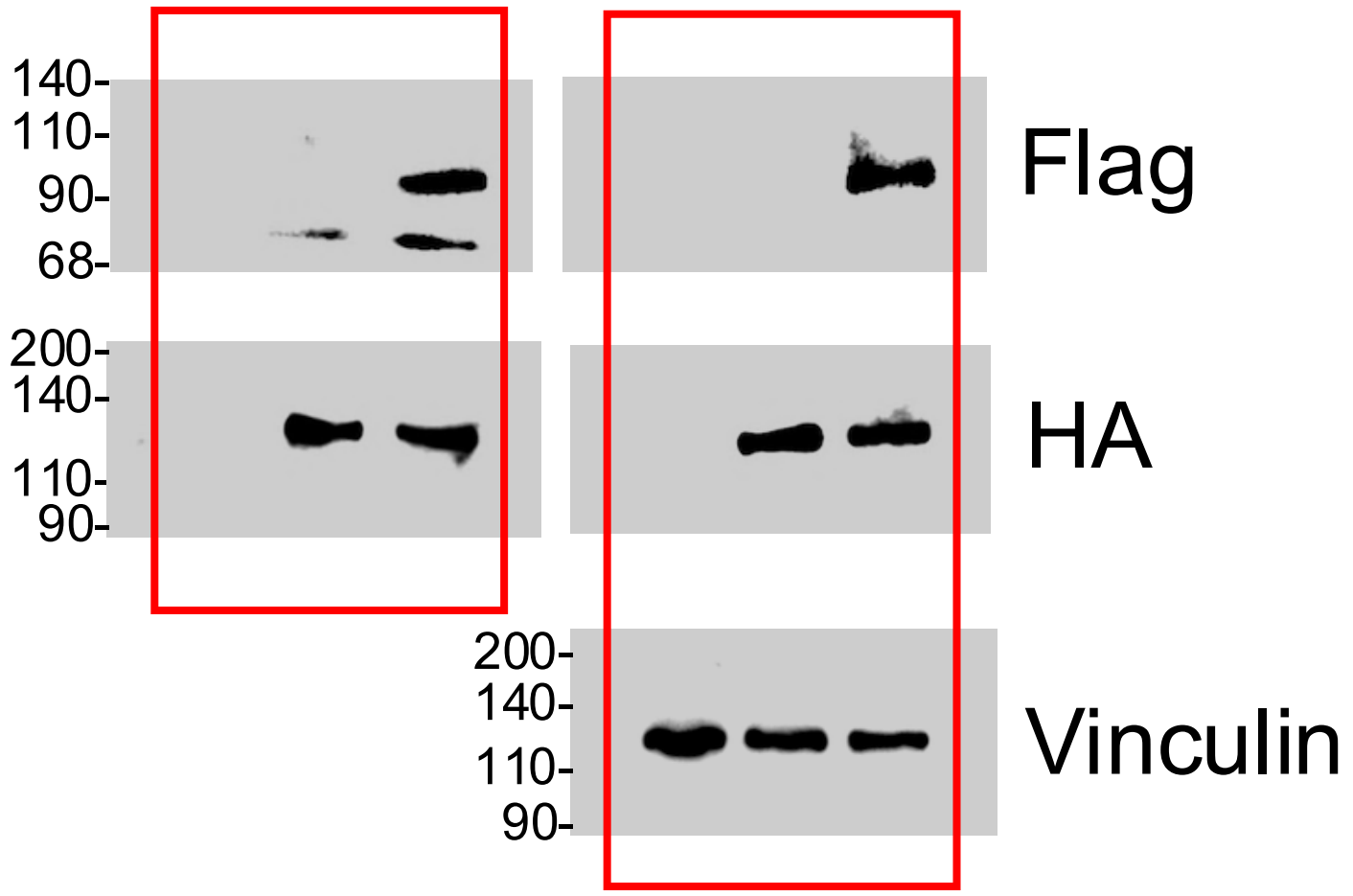

HEK293T

Figure S9F

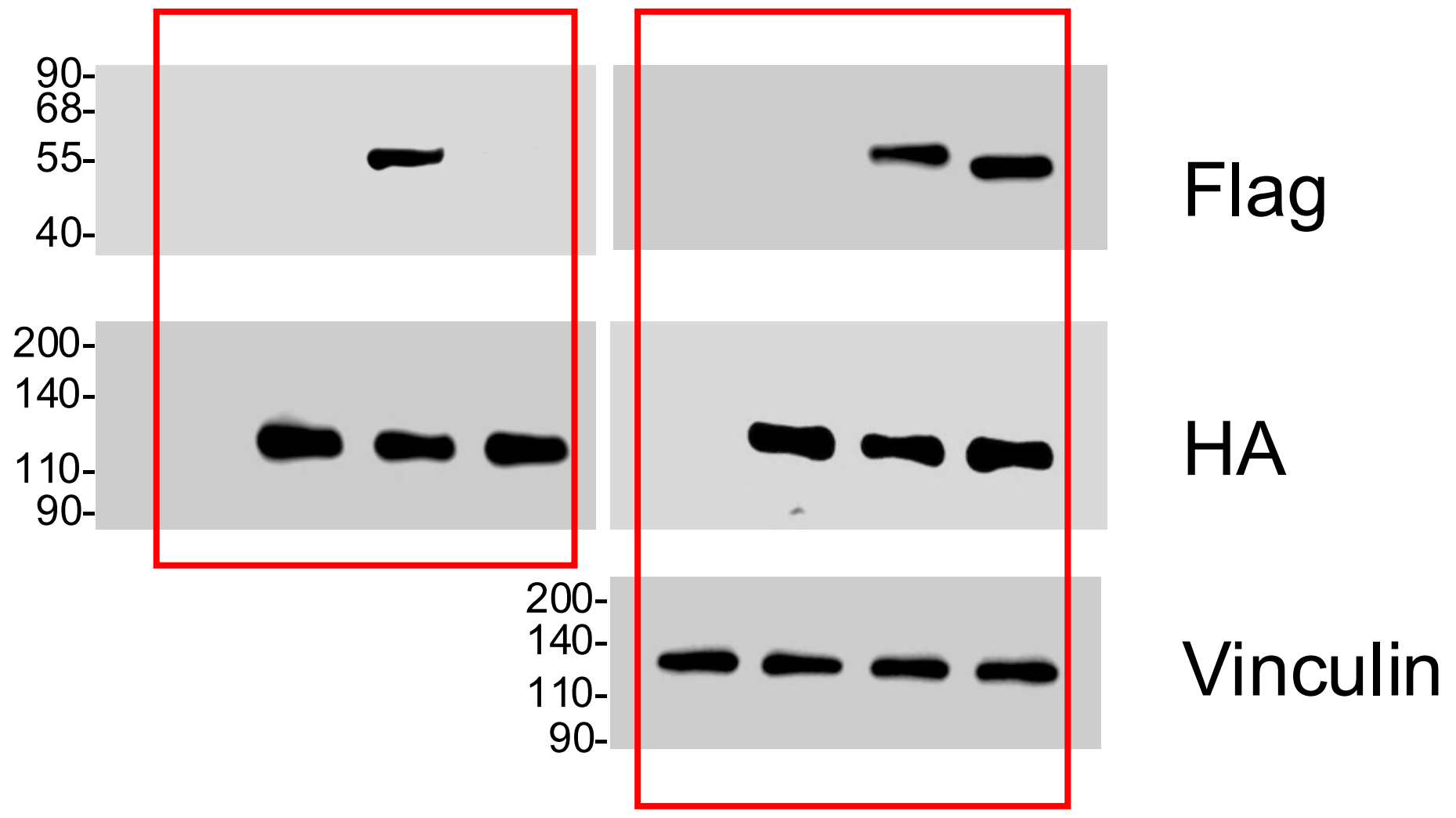

HEK293T

Figure S10A

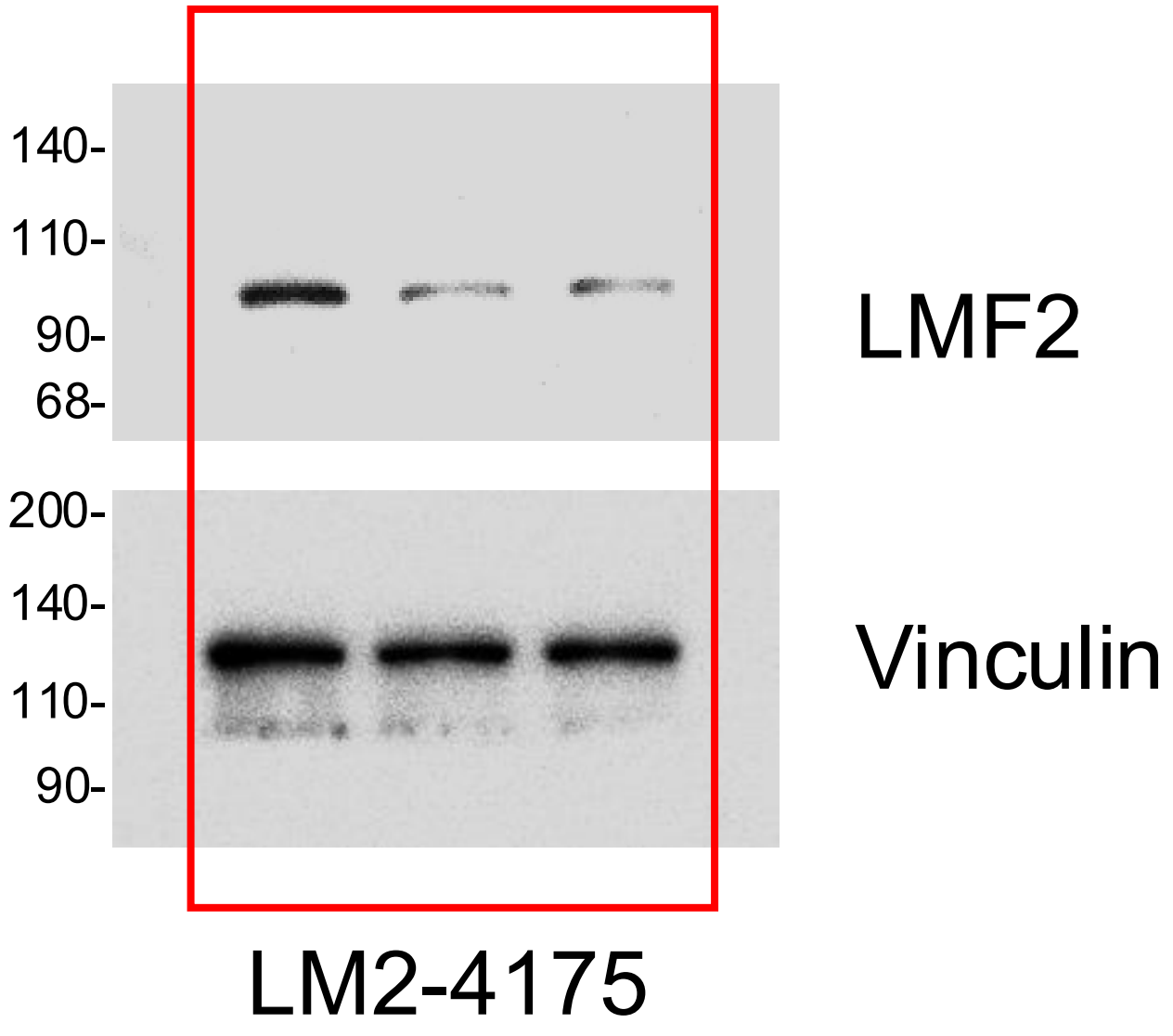

Figure S10I

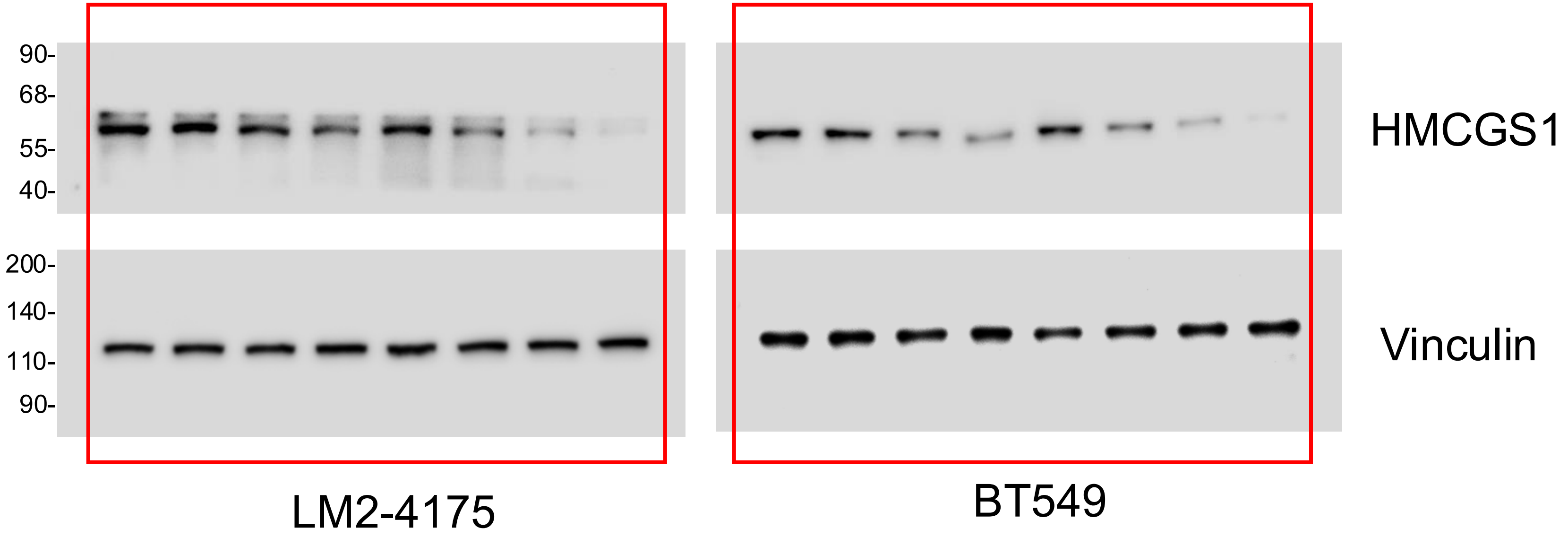

Figure S11A

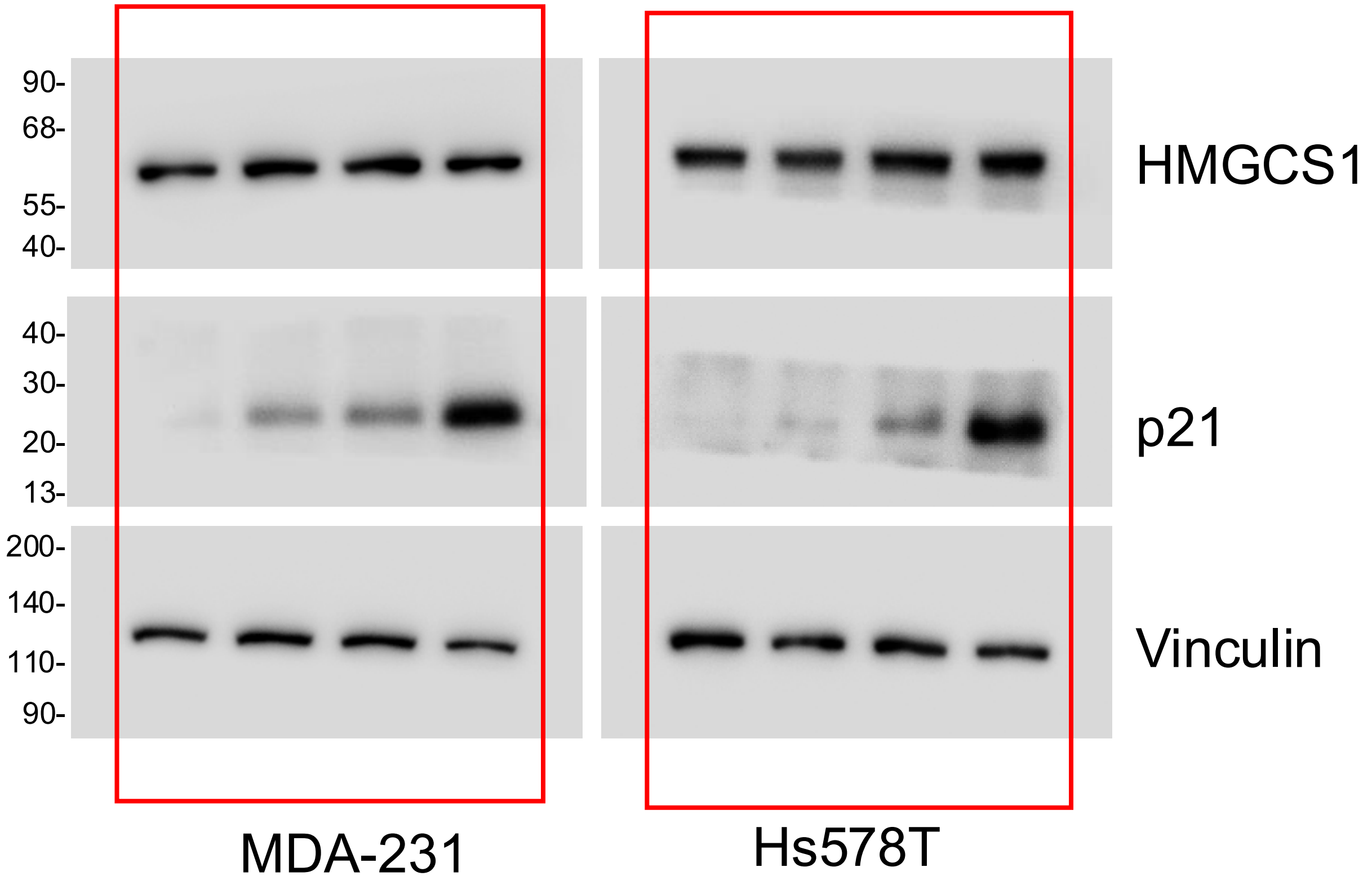

Figure S11B

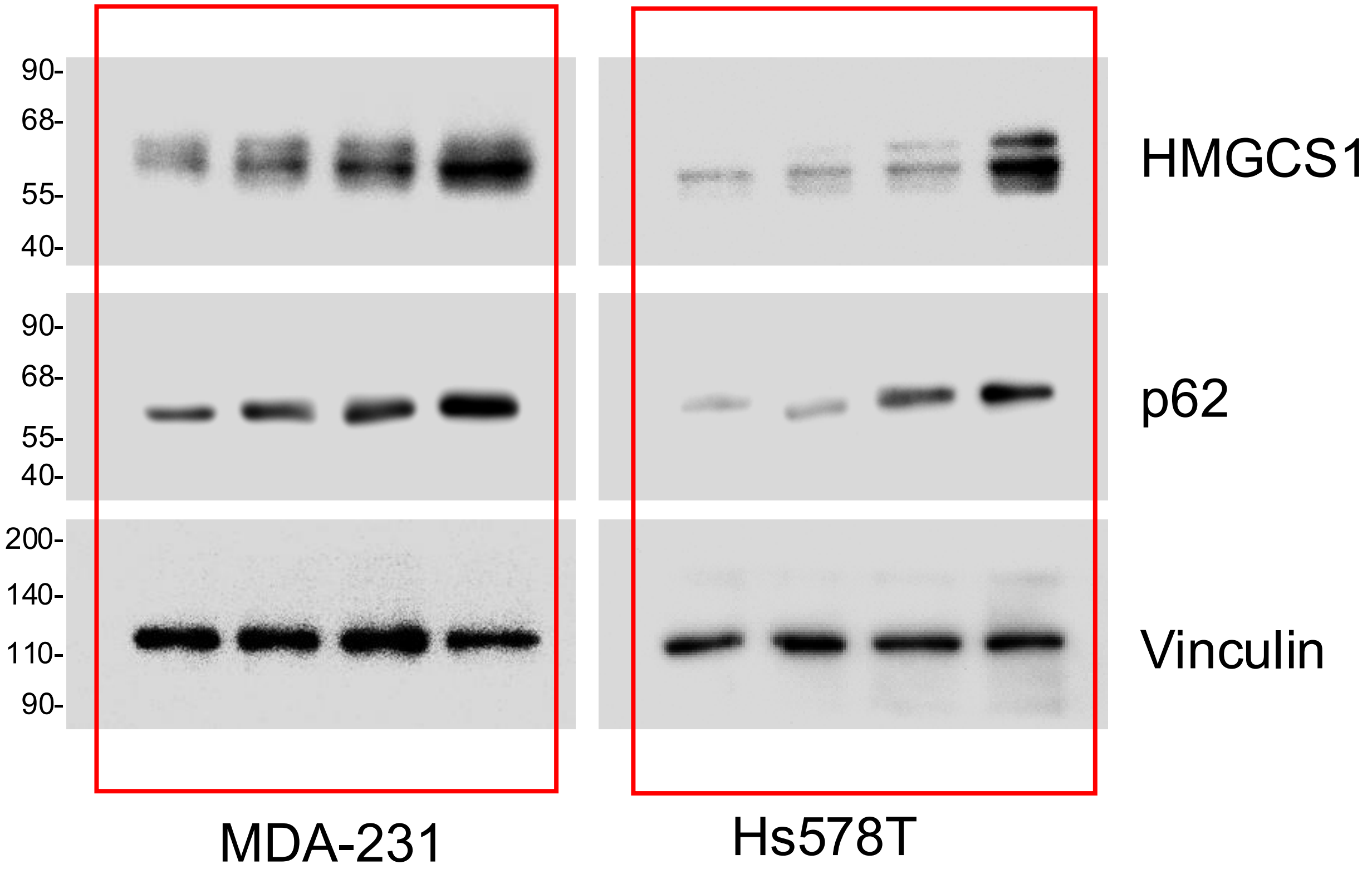

Figure S11C

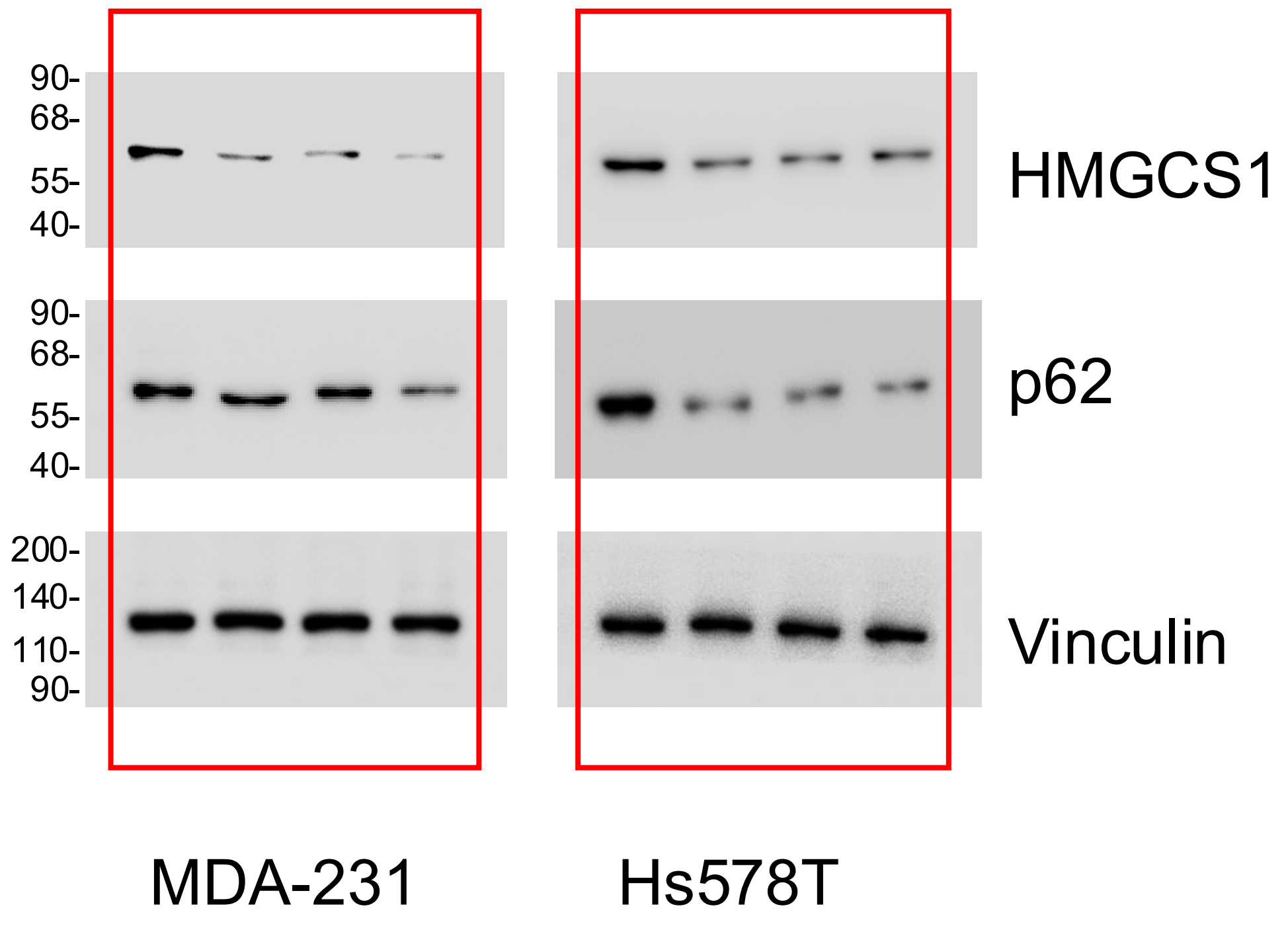

Figure S11D

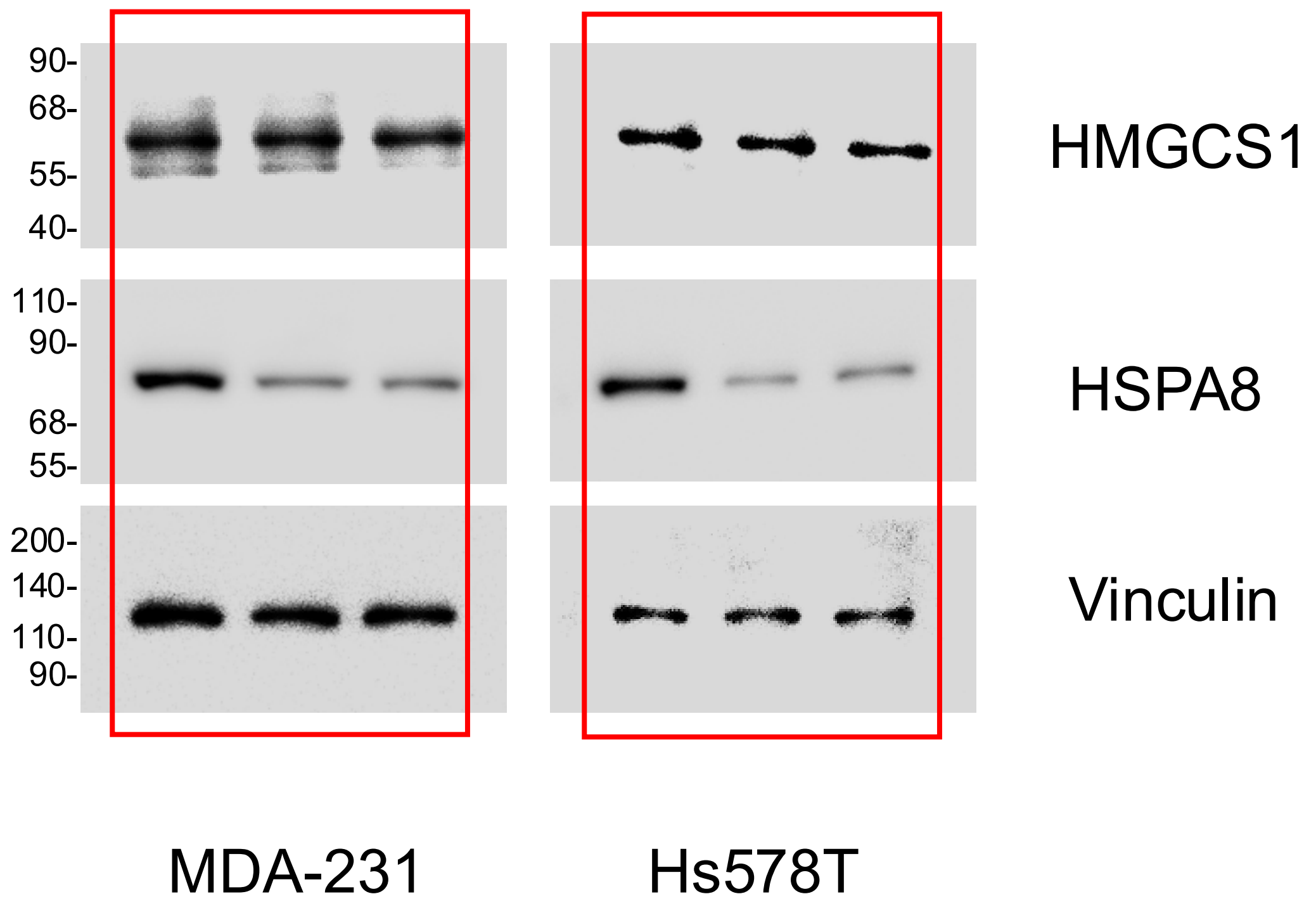

Figure S11E

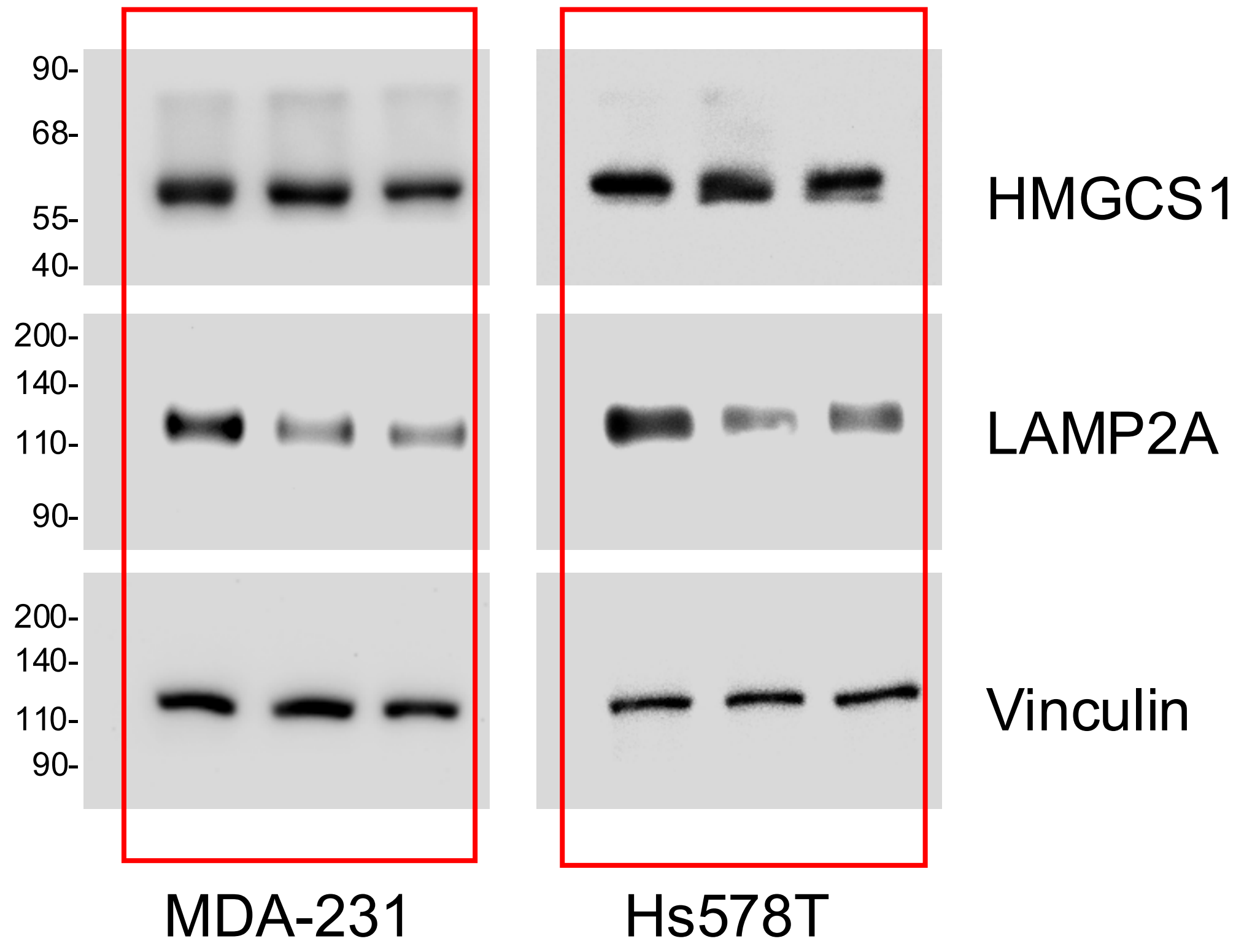

Figure S11F

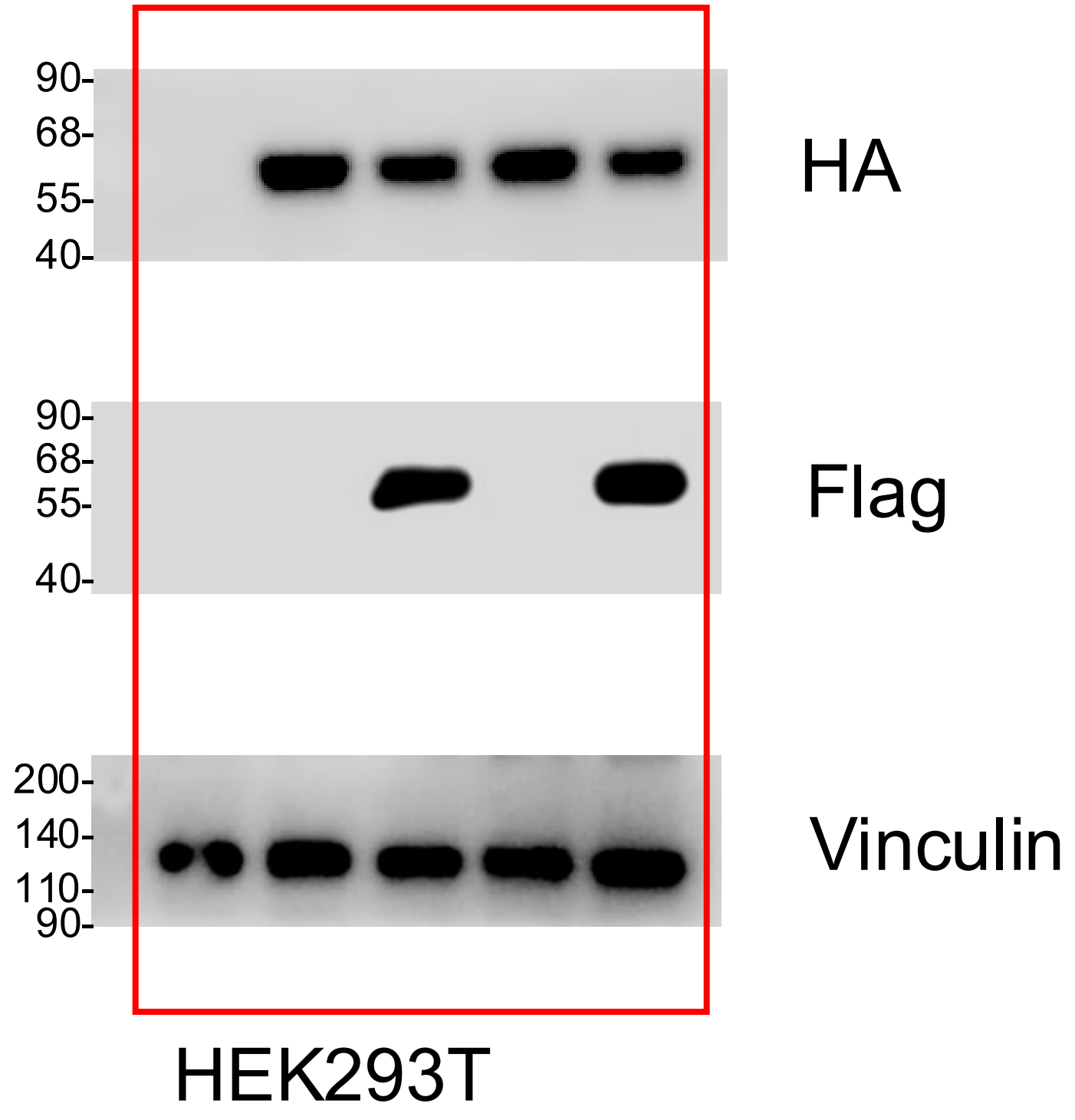

Figure S12B

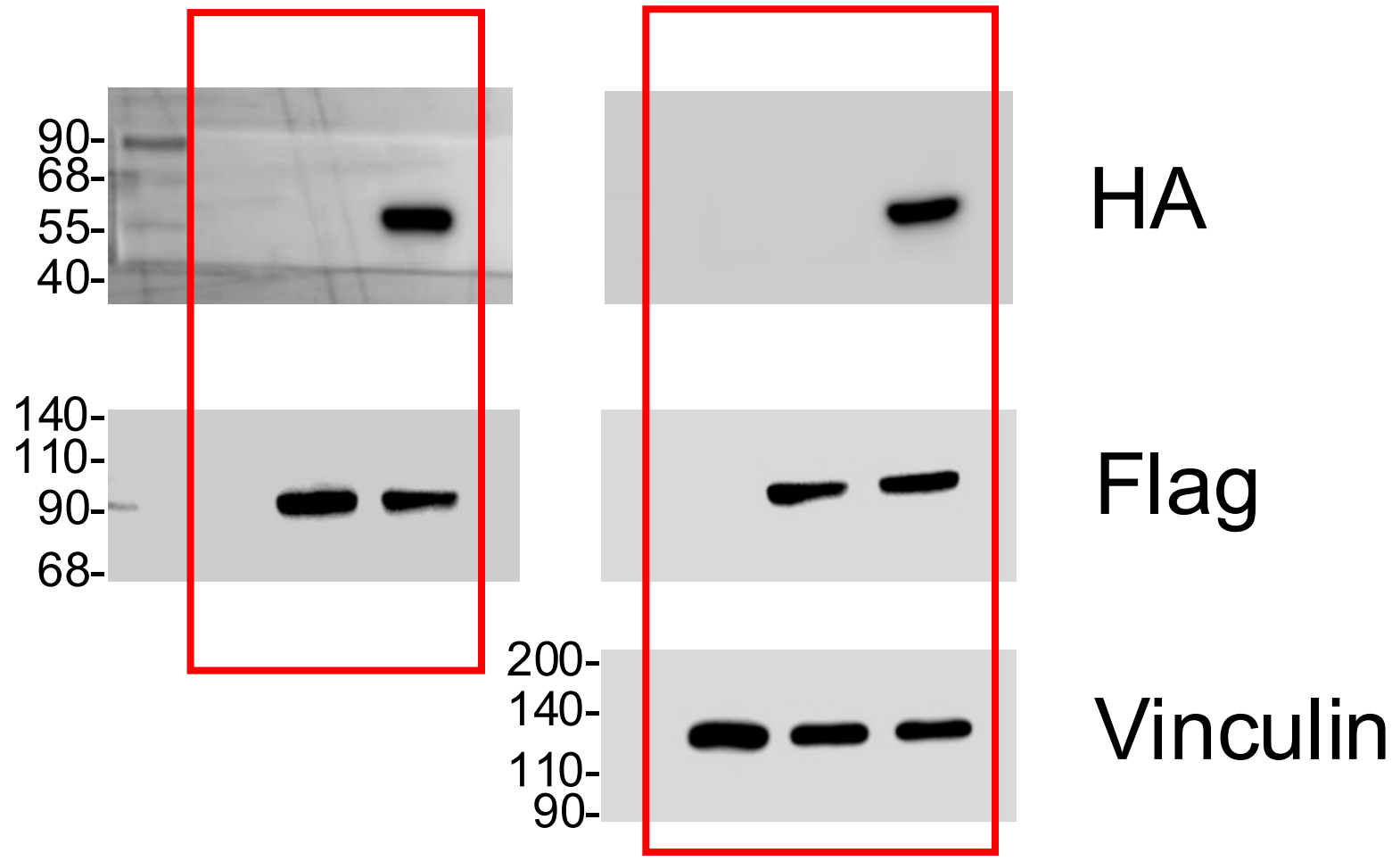

HEK293T

Figure S12C

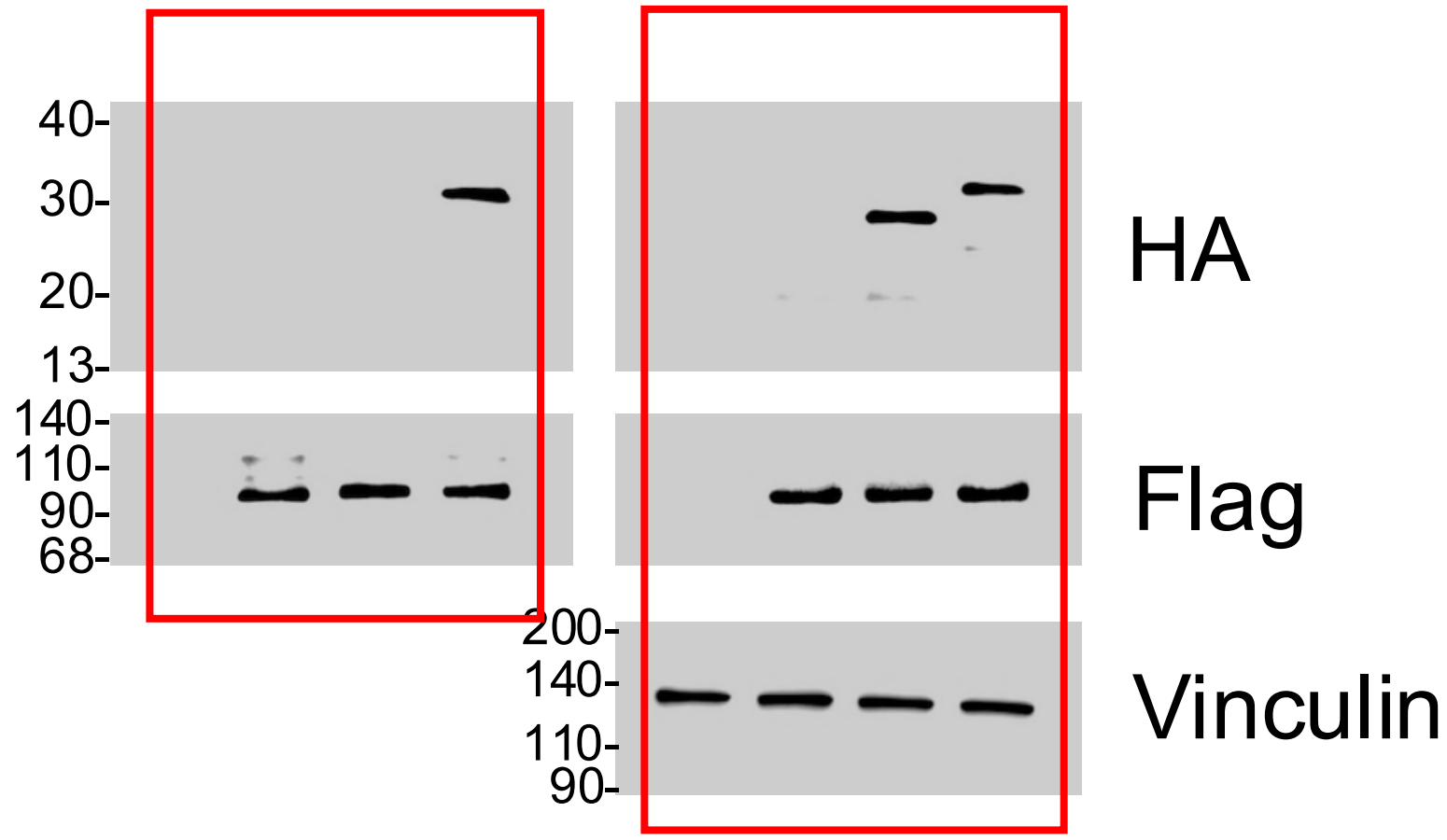

HEK293T

Figure S12D

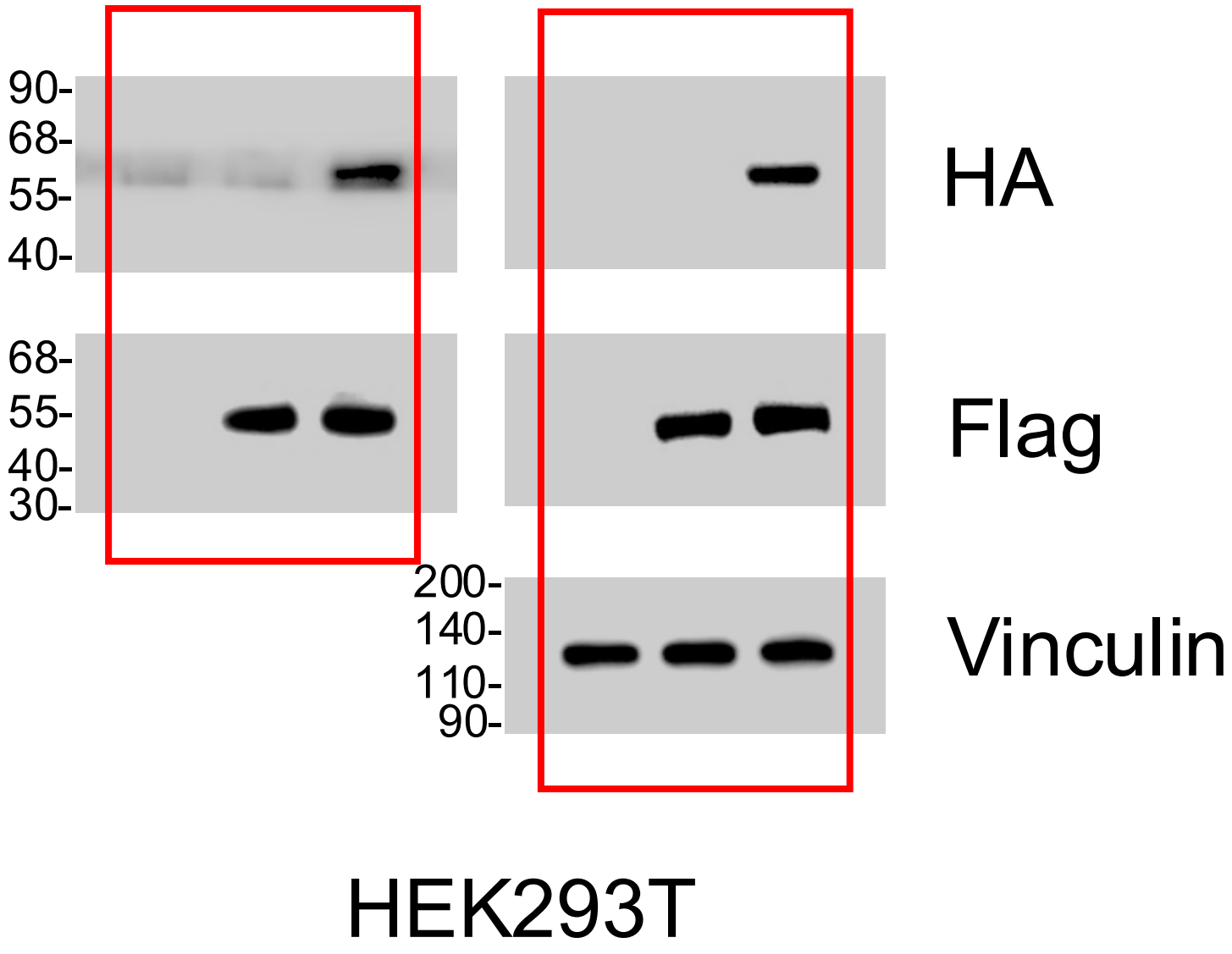

Figure S12E

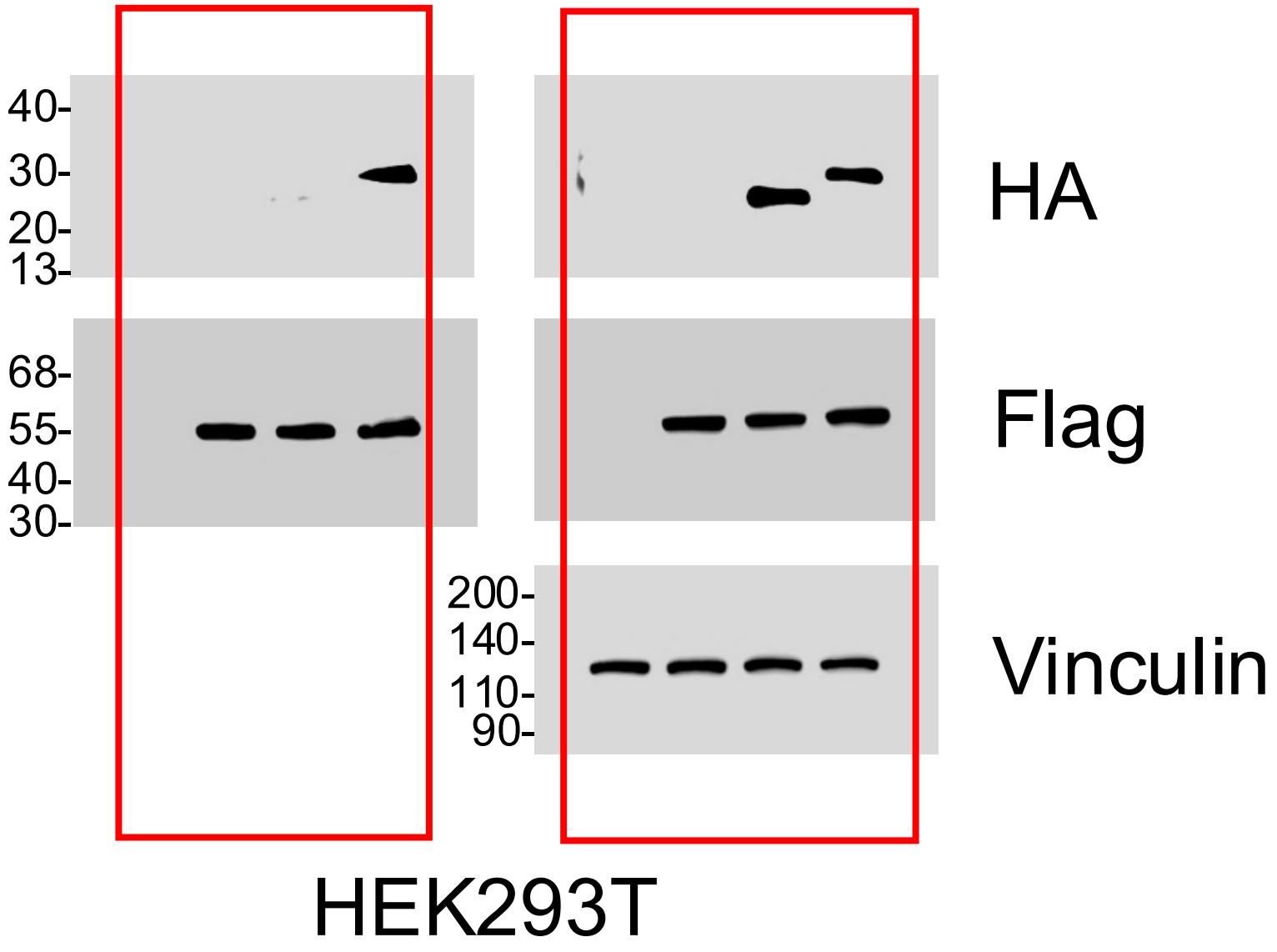

IHC
Figure 7H

TNBC：
4-2;4-3;4-4;4-5;4-6;4-7;4-8;4-9;4-10;
5-1;5-2;5-3;5-4;5-5;5-6;5-7;5-8;5-9;5-10;
6-1;6-2;6-3;6-4;6-5;6-6;

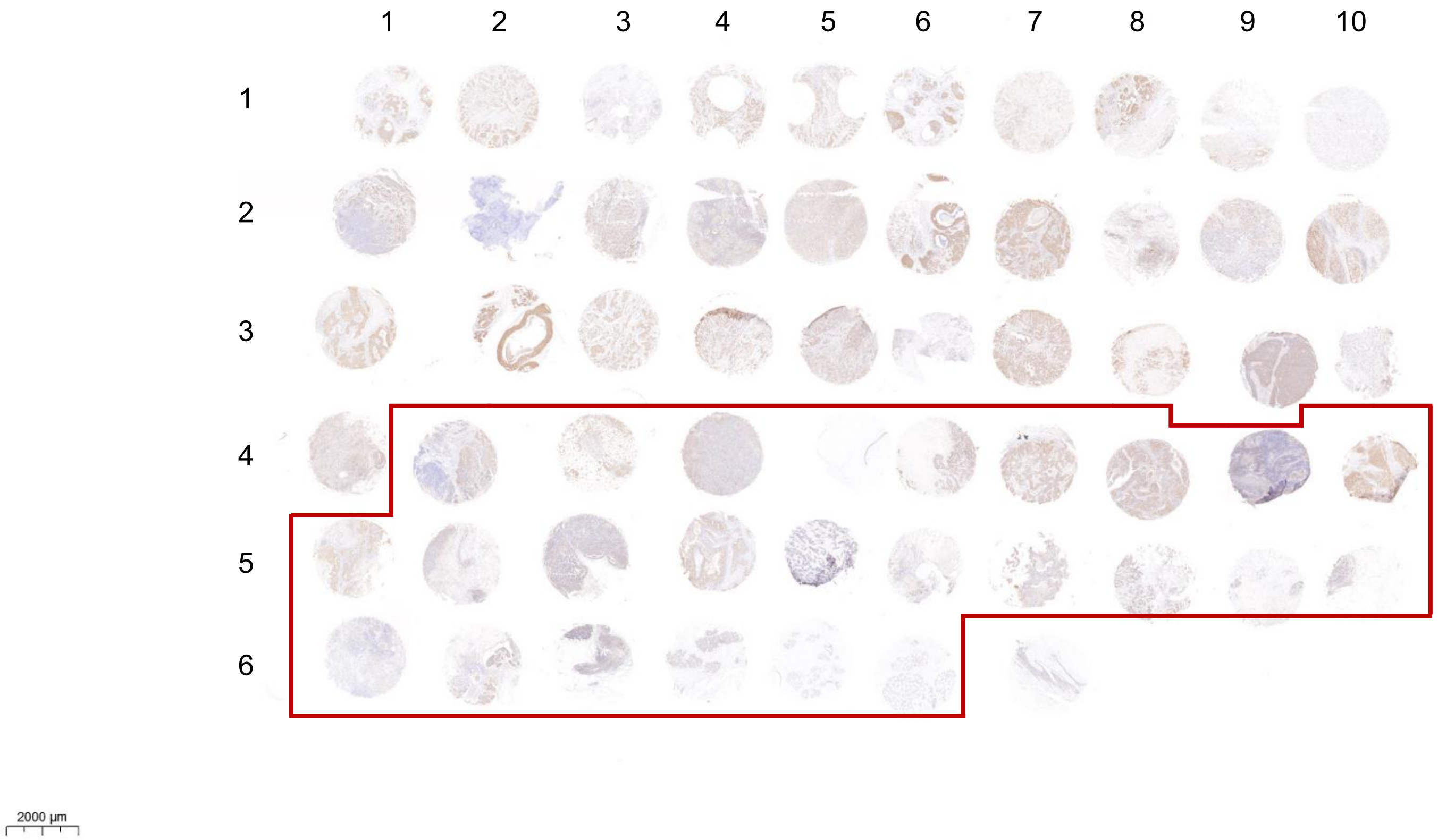

Figure7H
VCP use

IHC
Figure 7H

TNBC:
4-2;4-3;4-4;4-5;4-6;4-7;4-8;4-9;4-10;
5-1;5-2;5-3;5-4;5-5;5-6;5-7;5-8;5-9;5-10;
6-1;6-2;6-3;6-4;6-5;6-6;

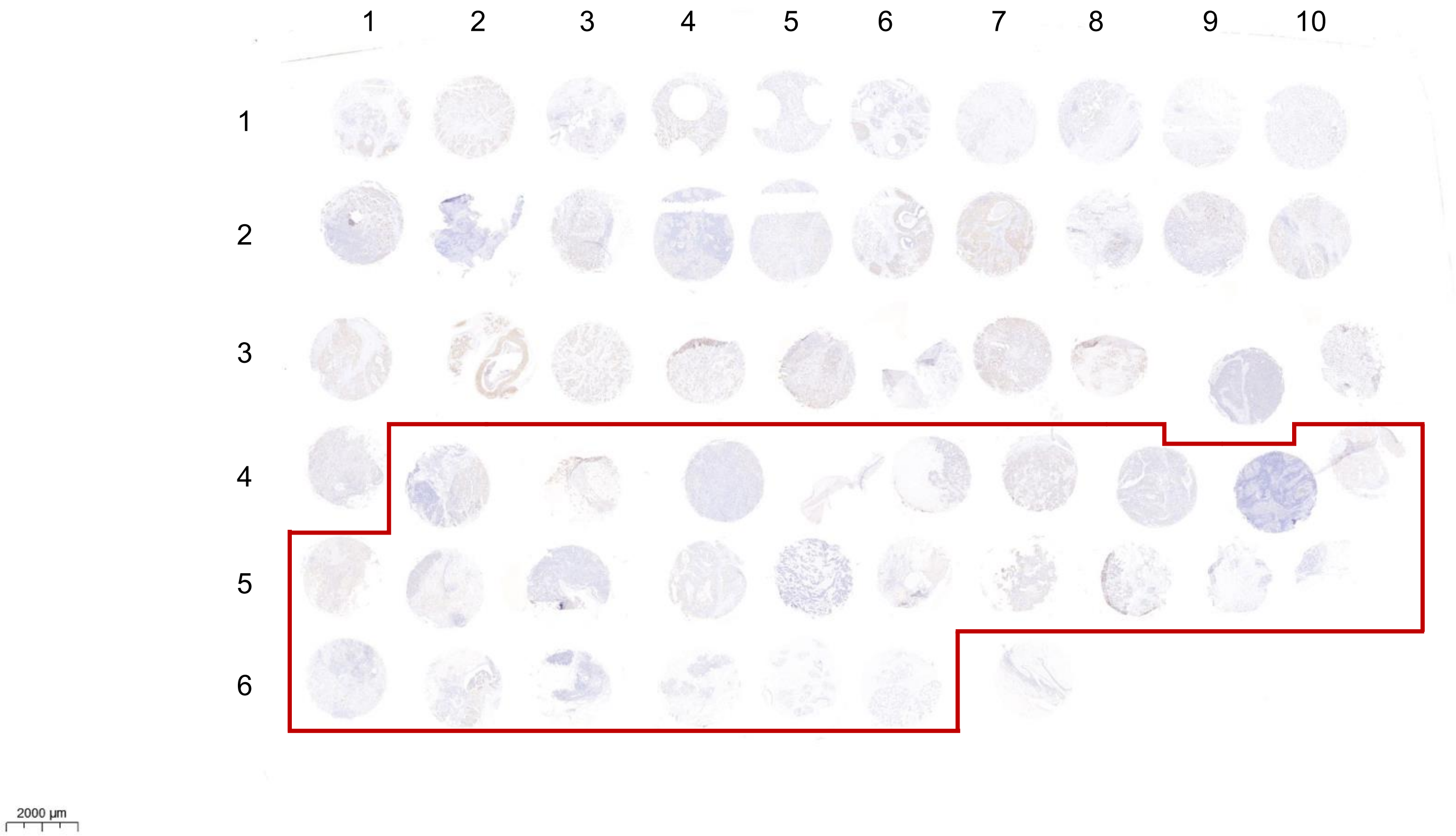

Figure7H
LMF2 use

IHC
Figure 7H

TNBC:
4-2;4-3;4-4;4-5;4-6;4-7;4-8;4-9;4-10;
5-1;5-2;5-3;5-4;5-5;5-6;5-7;5-8;5-9;5-10;
6-1;6-2;6-3;6-4;6-5;6-6;

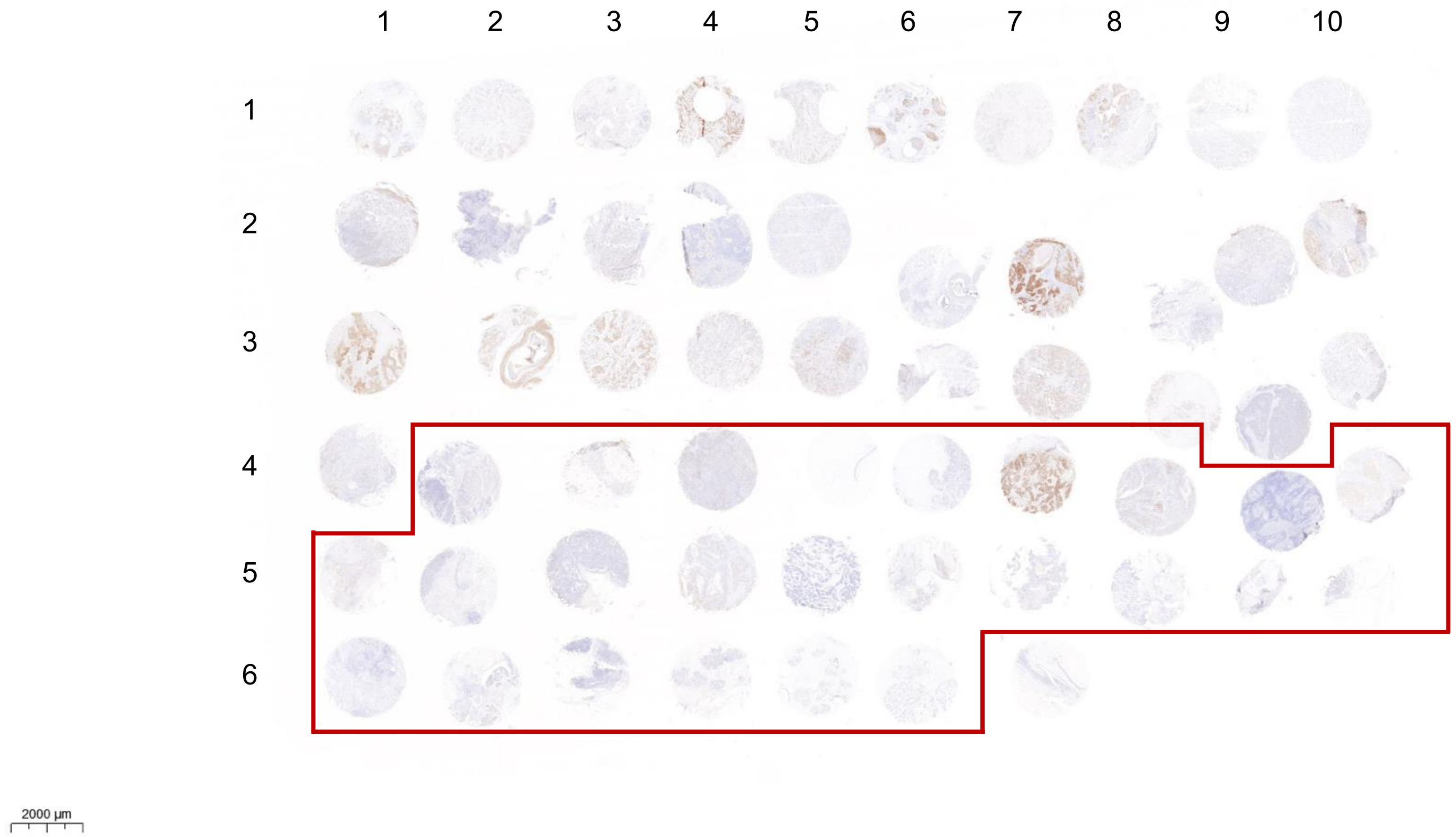

Figure7H
HMGCS1 use

IHC

Figure 4F

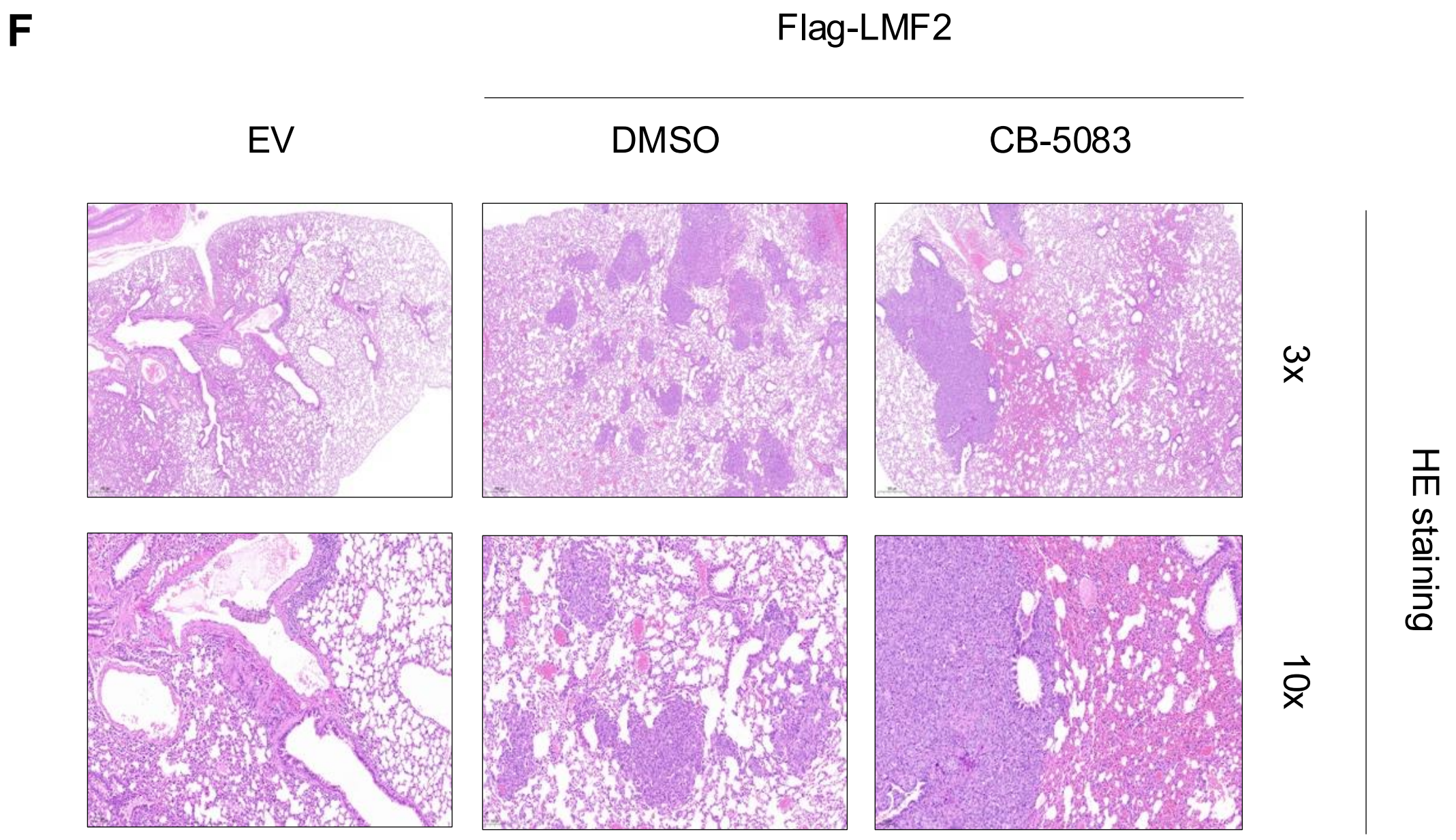

Figure 6G

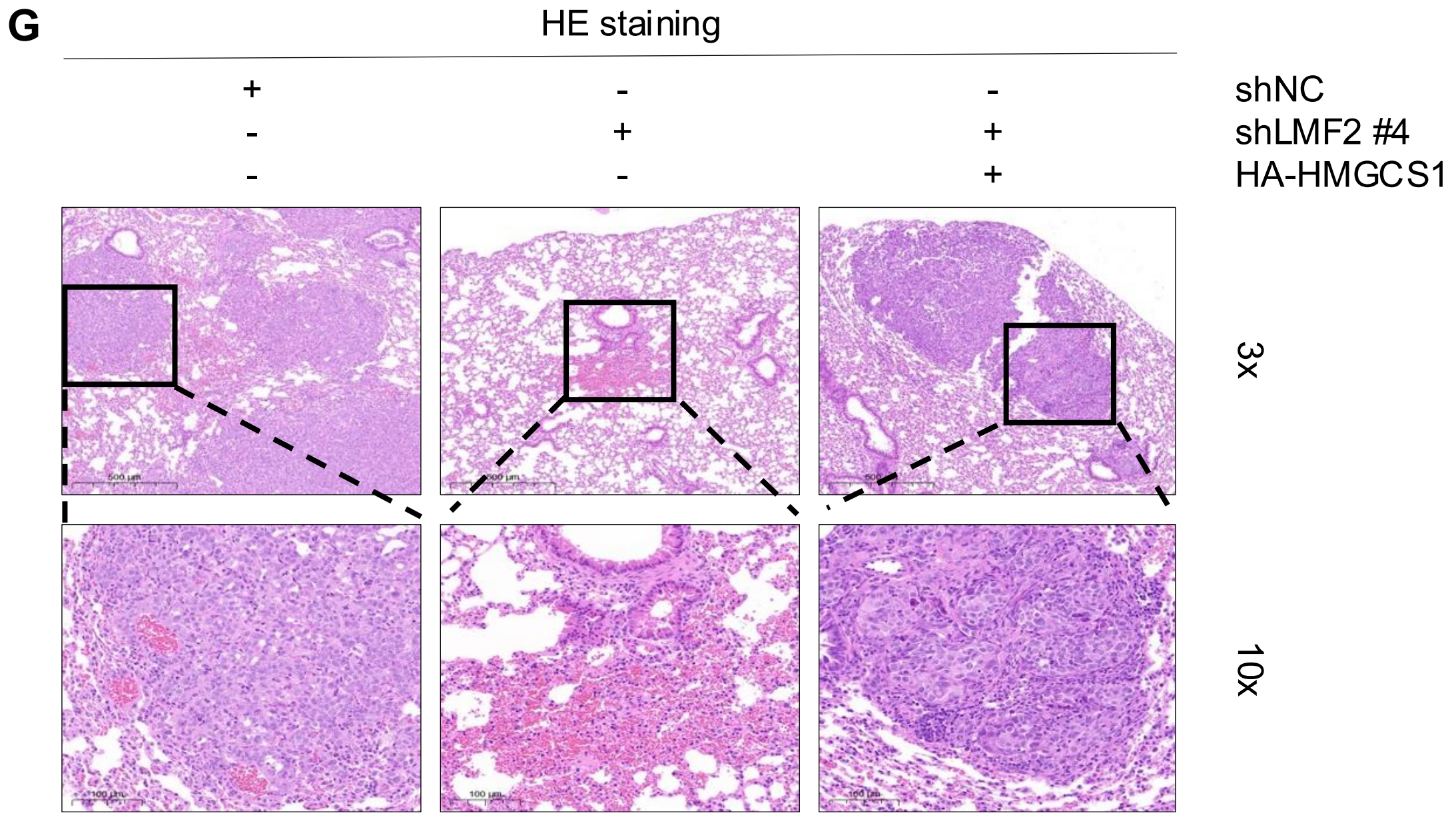

HE staining

Supplementary Figure S2H

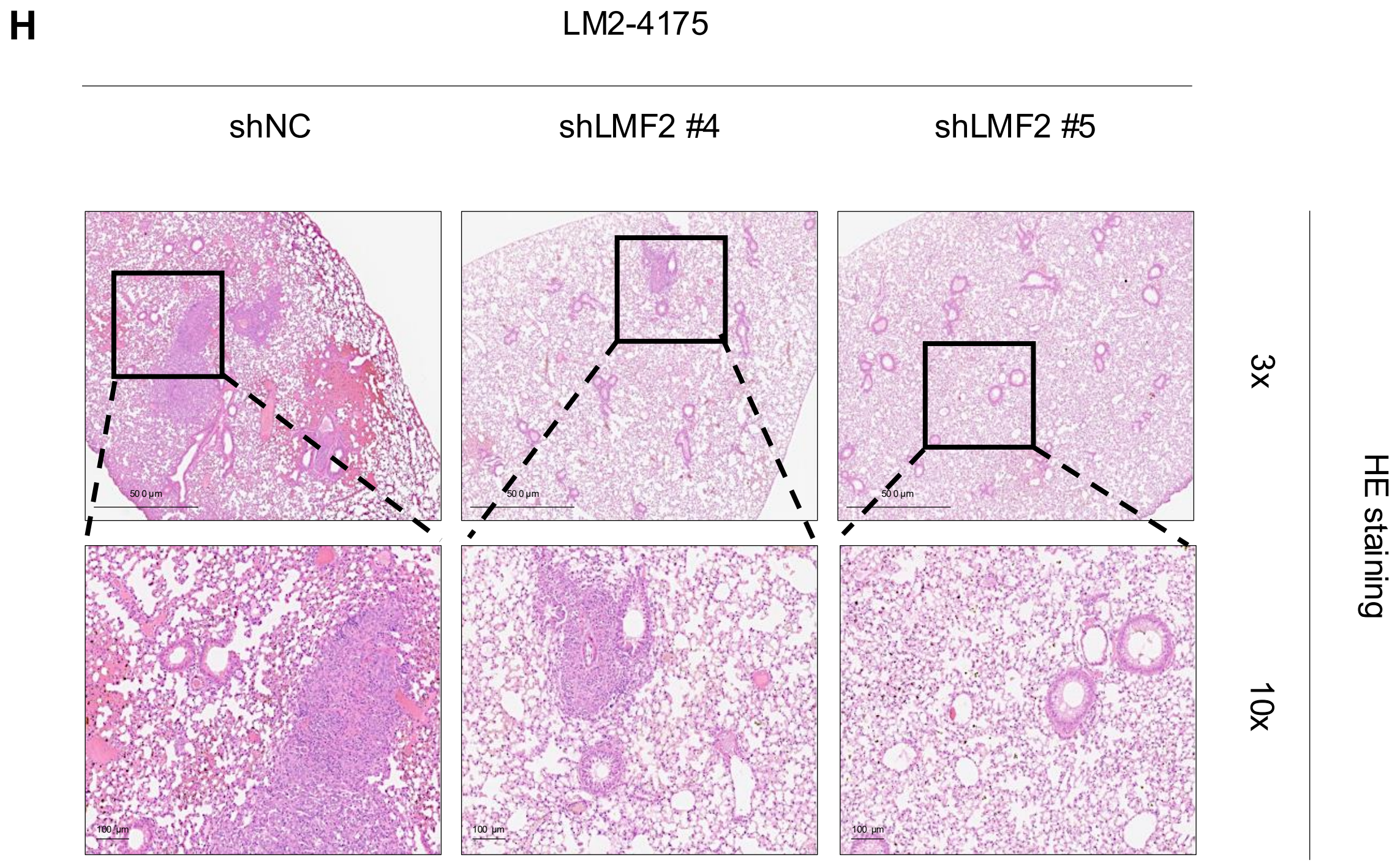

IF

Figure 1L

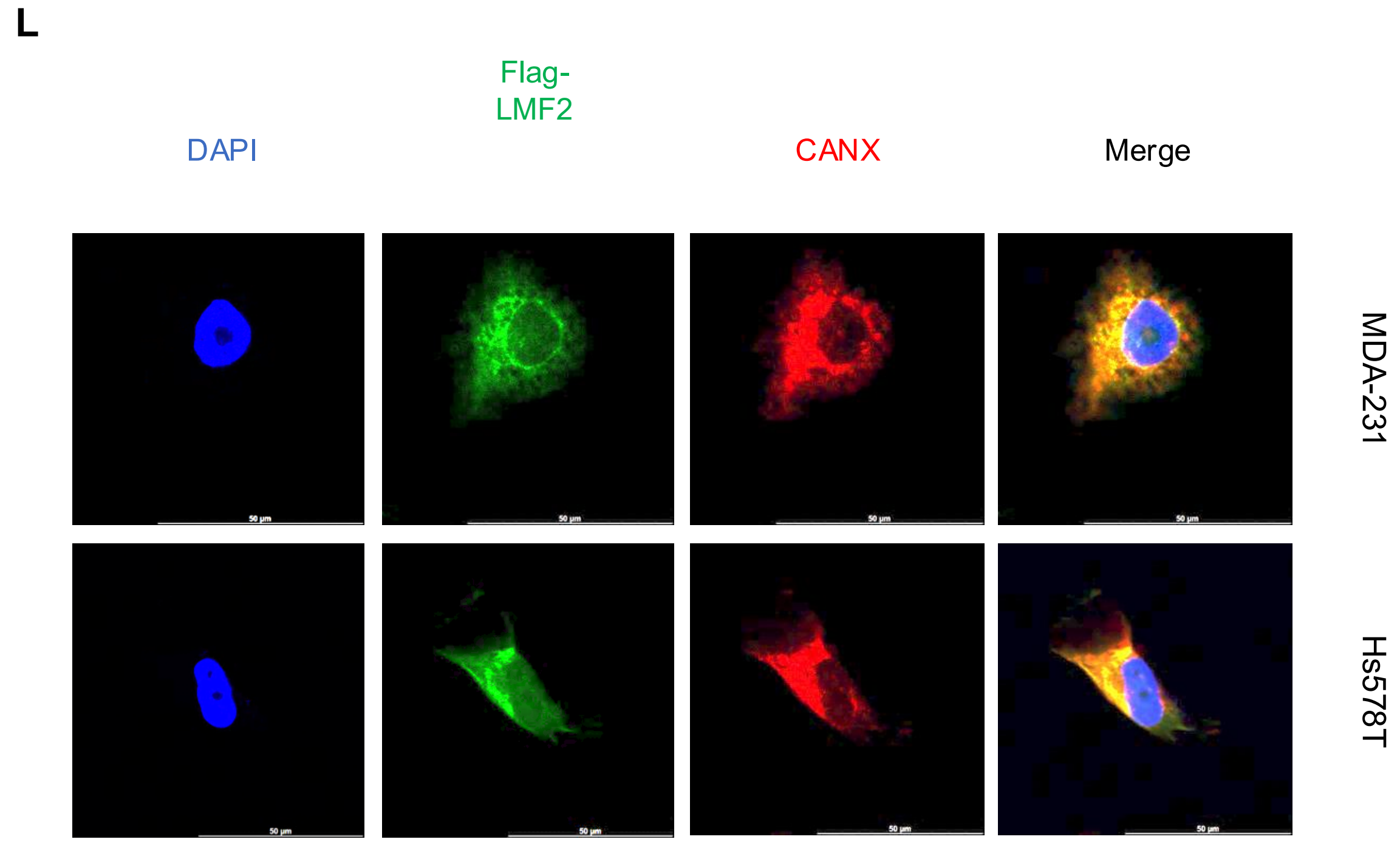

Figure 1M

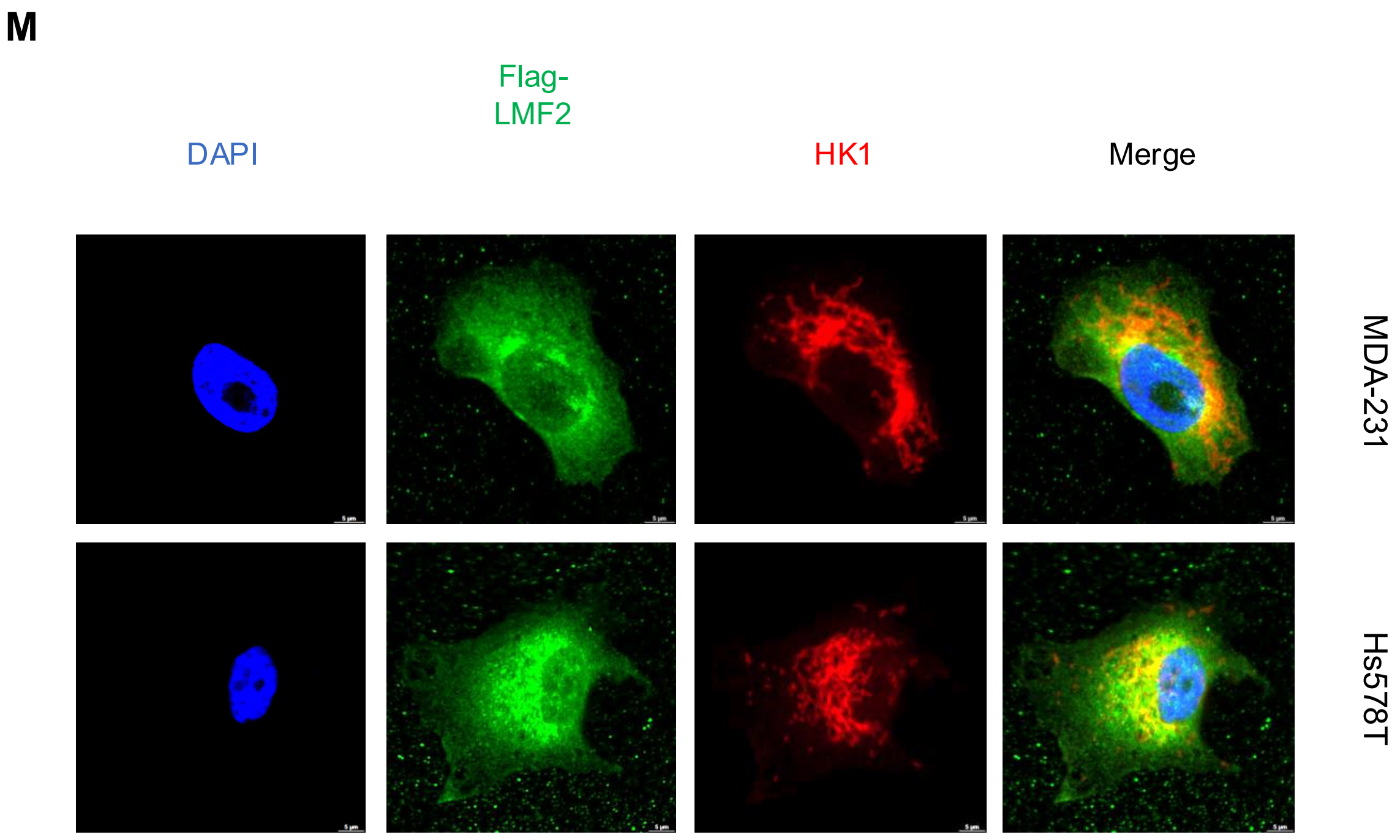

IF

Figure 1N

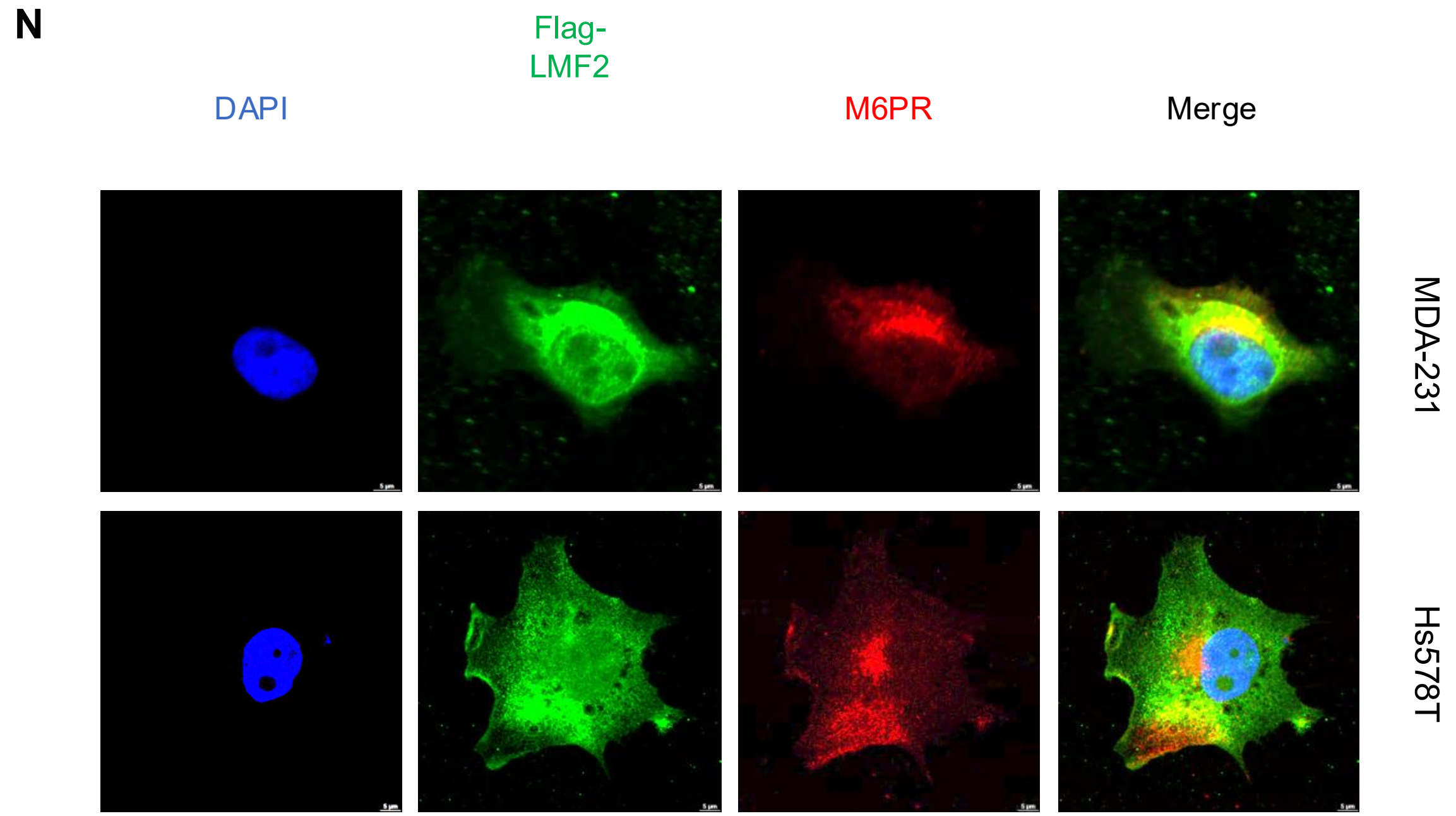

Figure 1O

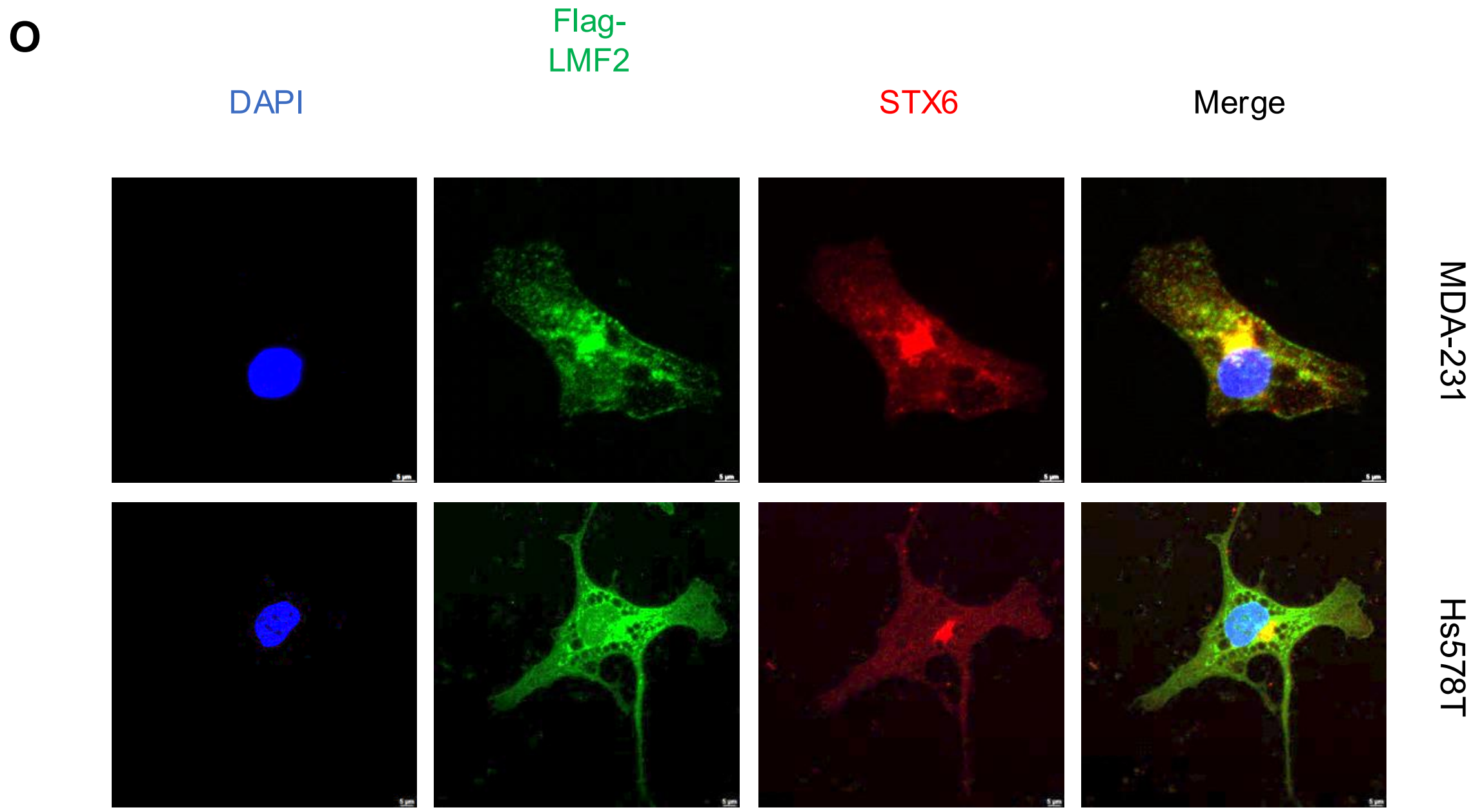

IF

Figure 1P

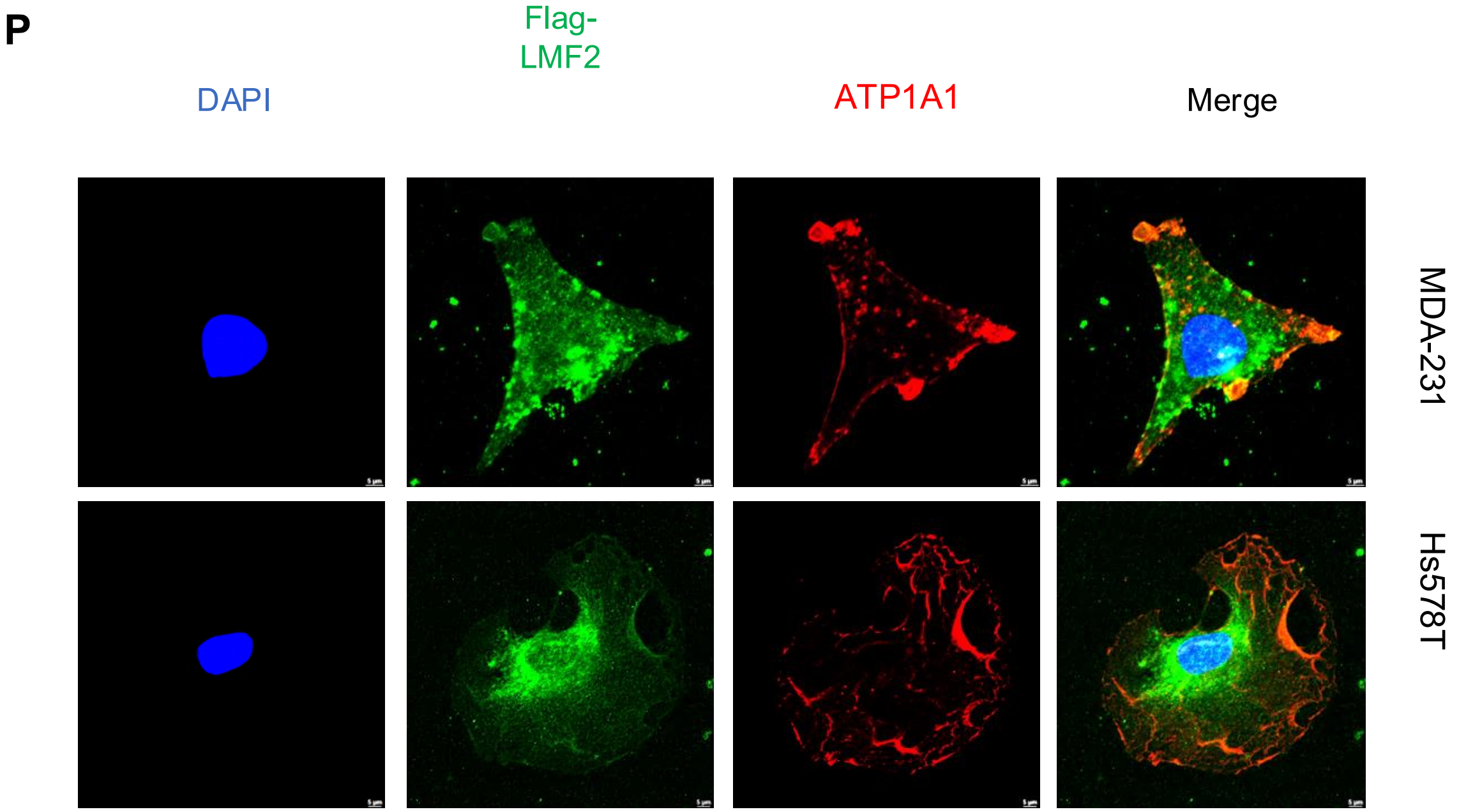

IF

Figure 3E

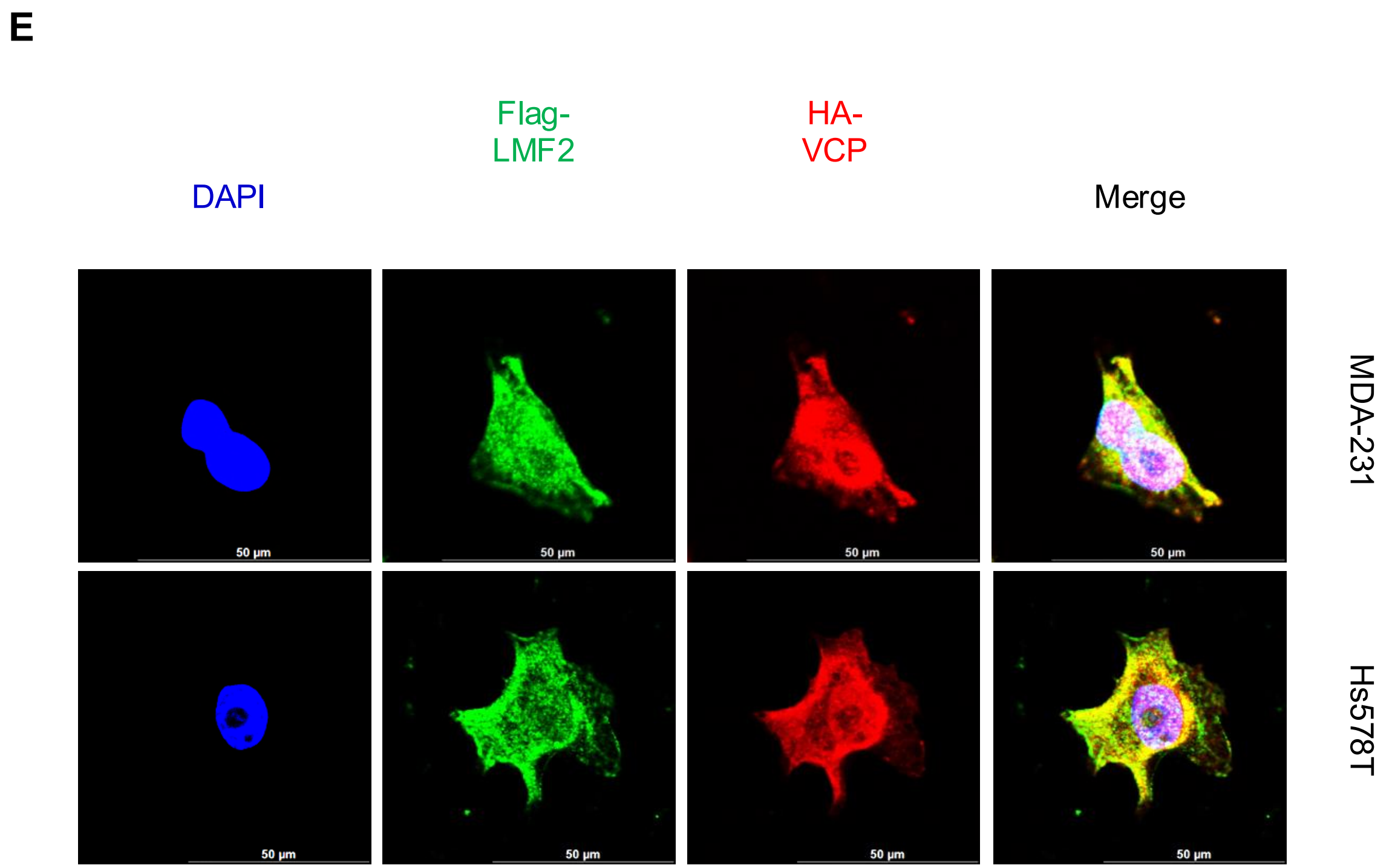

IF

Figure 5F

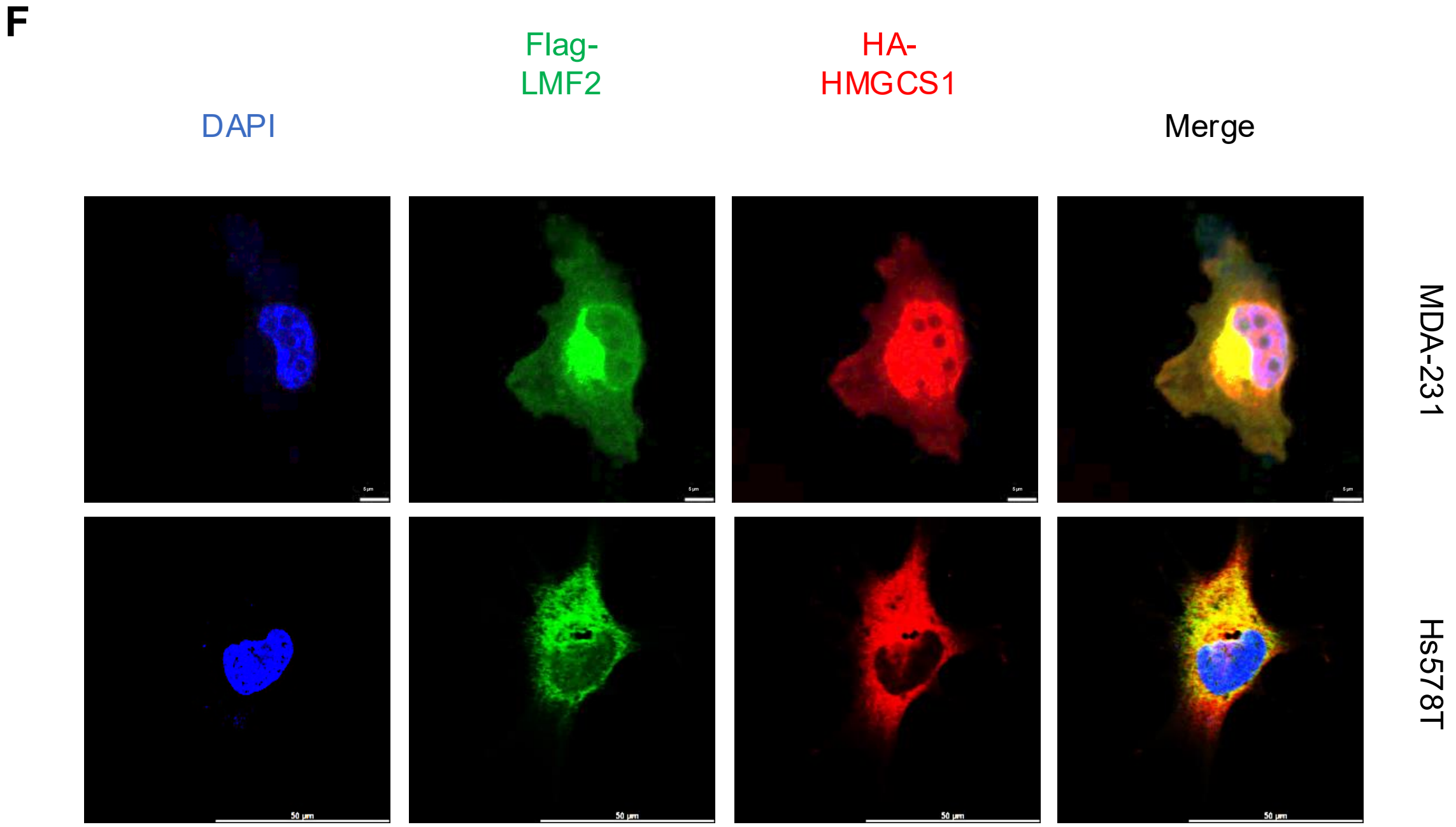

Transwell

Supplementary Figure S2E (100x)

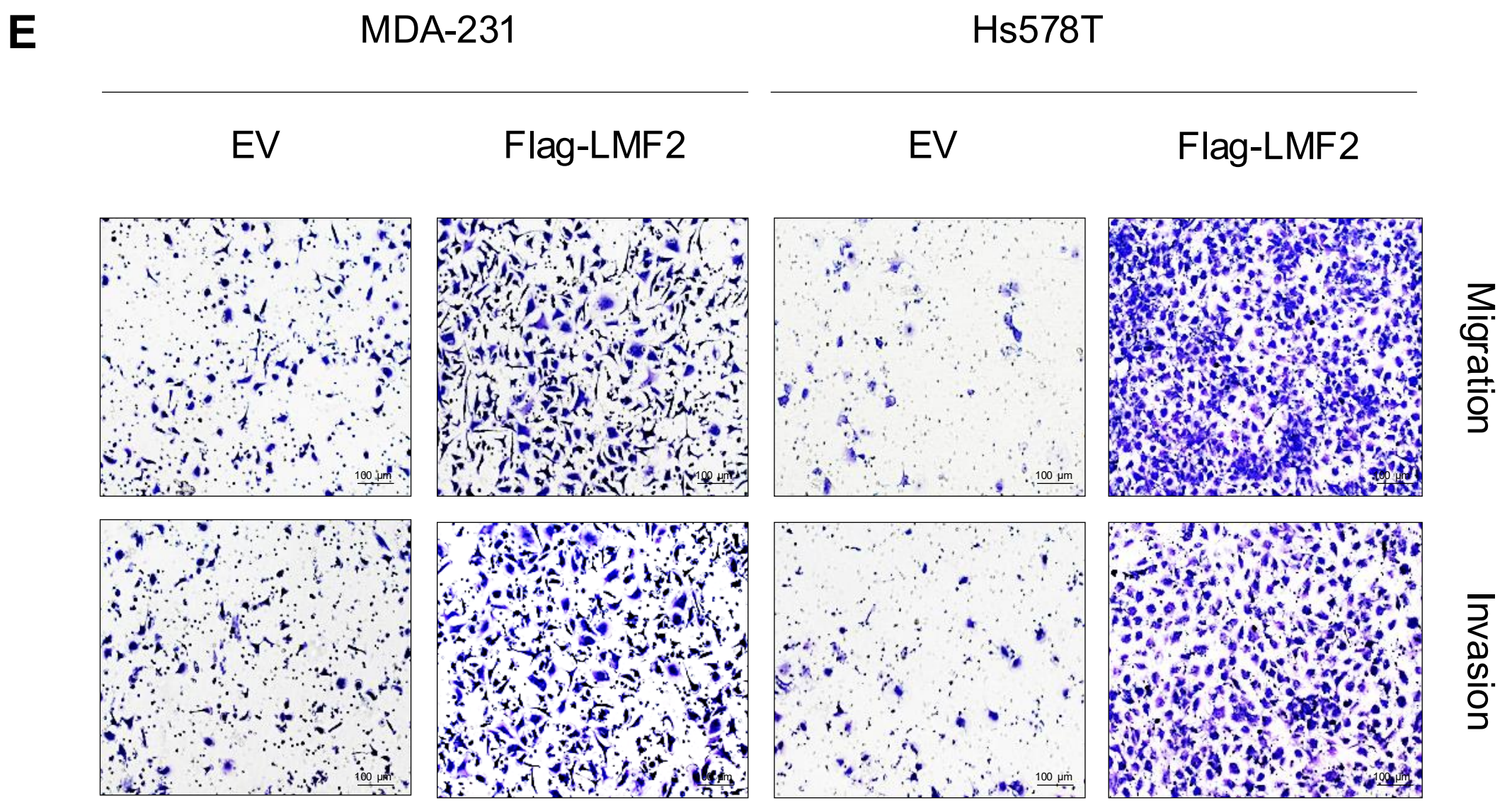

Supplementary Figure S2F (100x)

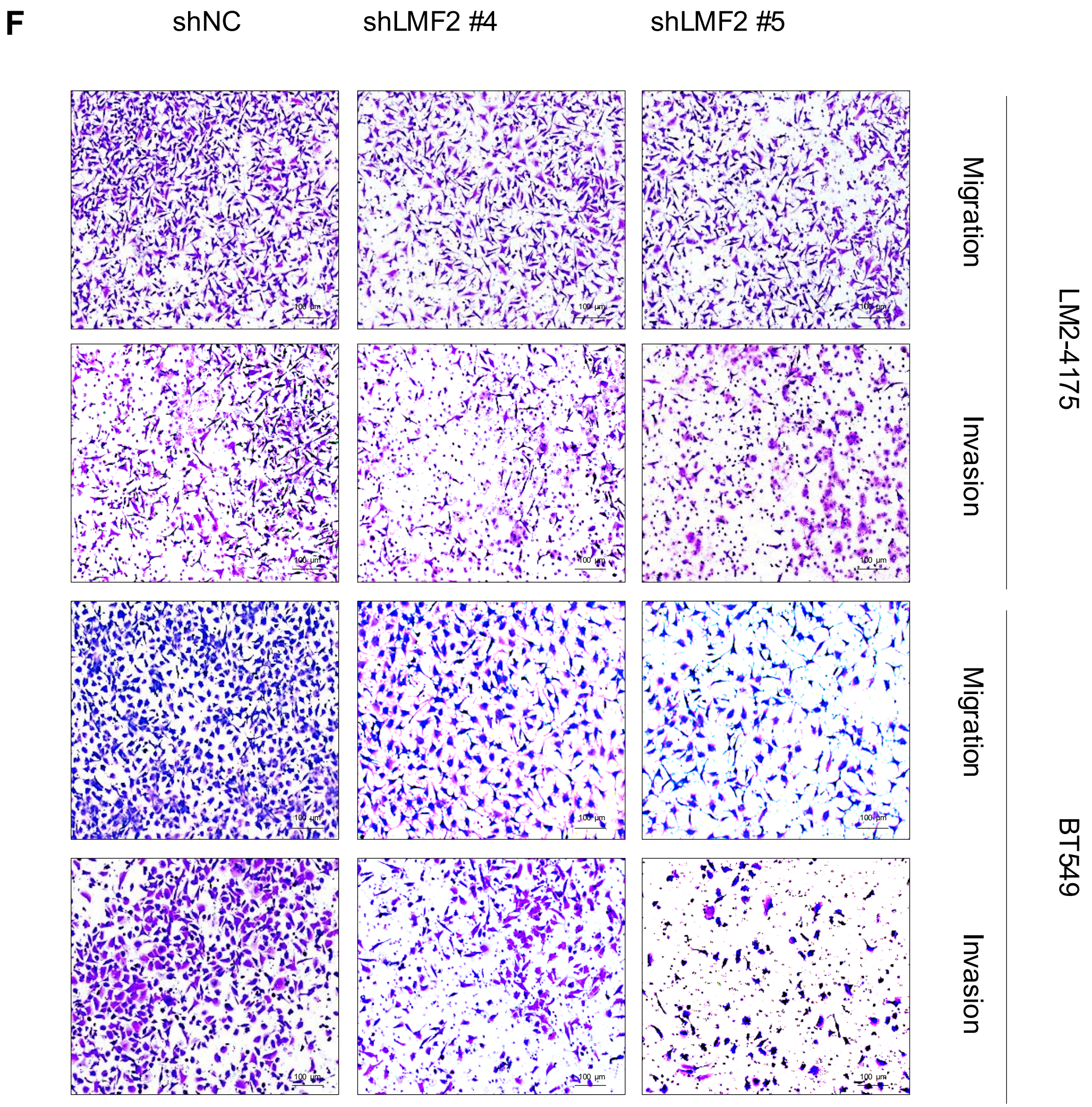

Transwell

Supplementary Figure S2D (00x)

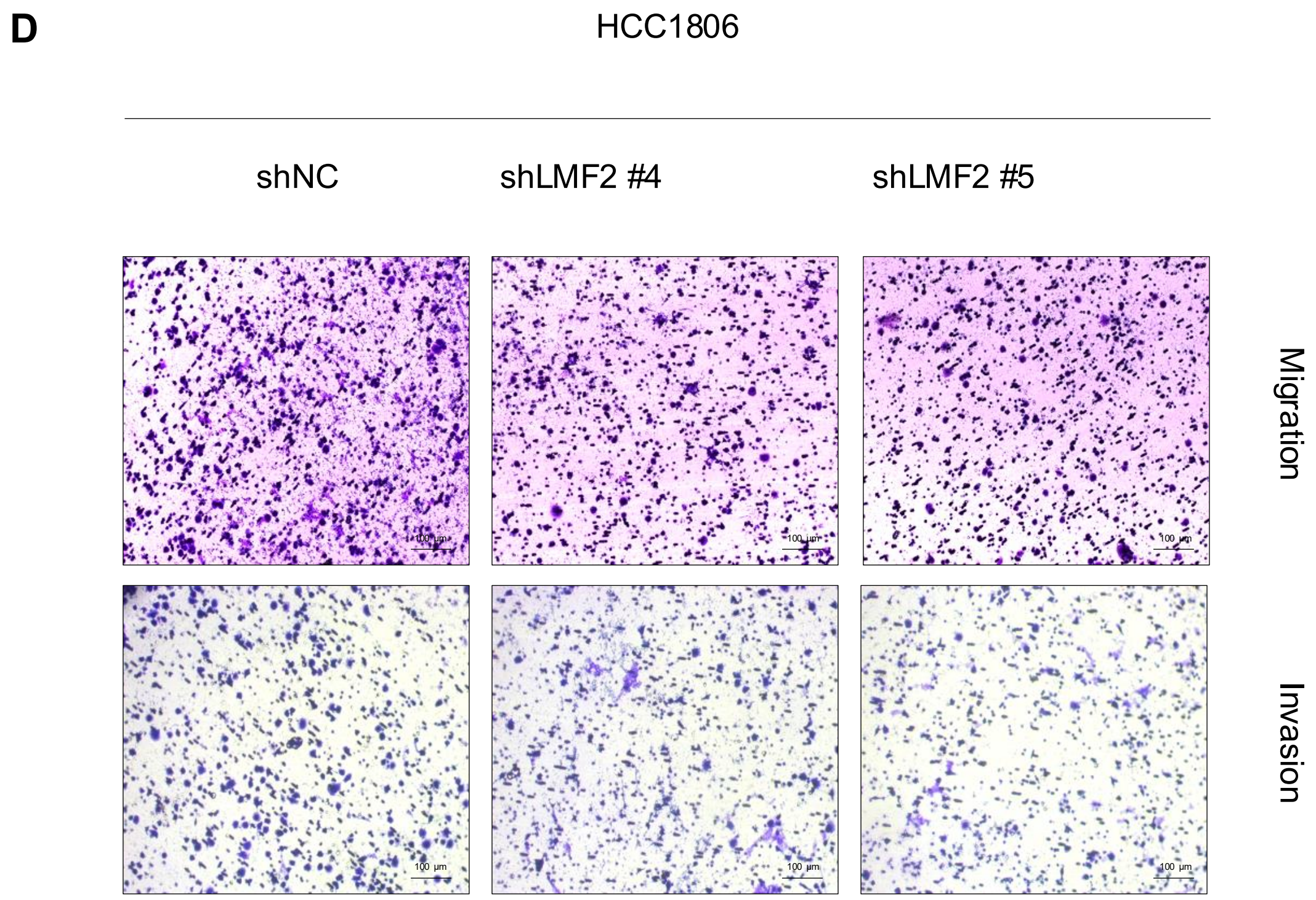

Transwell

Supplementary Figure S13D (100x)

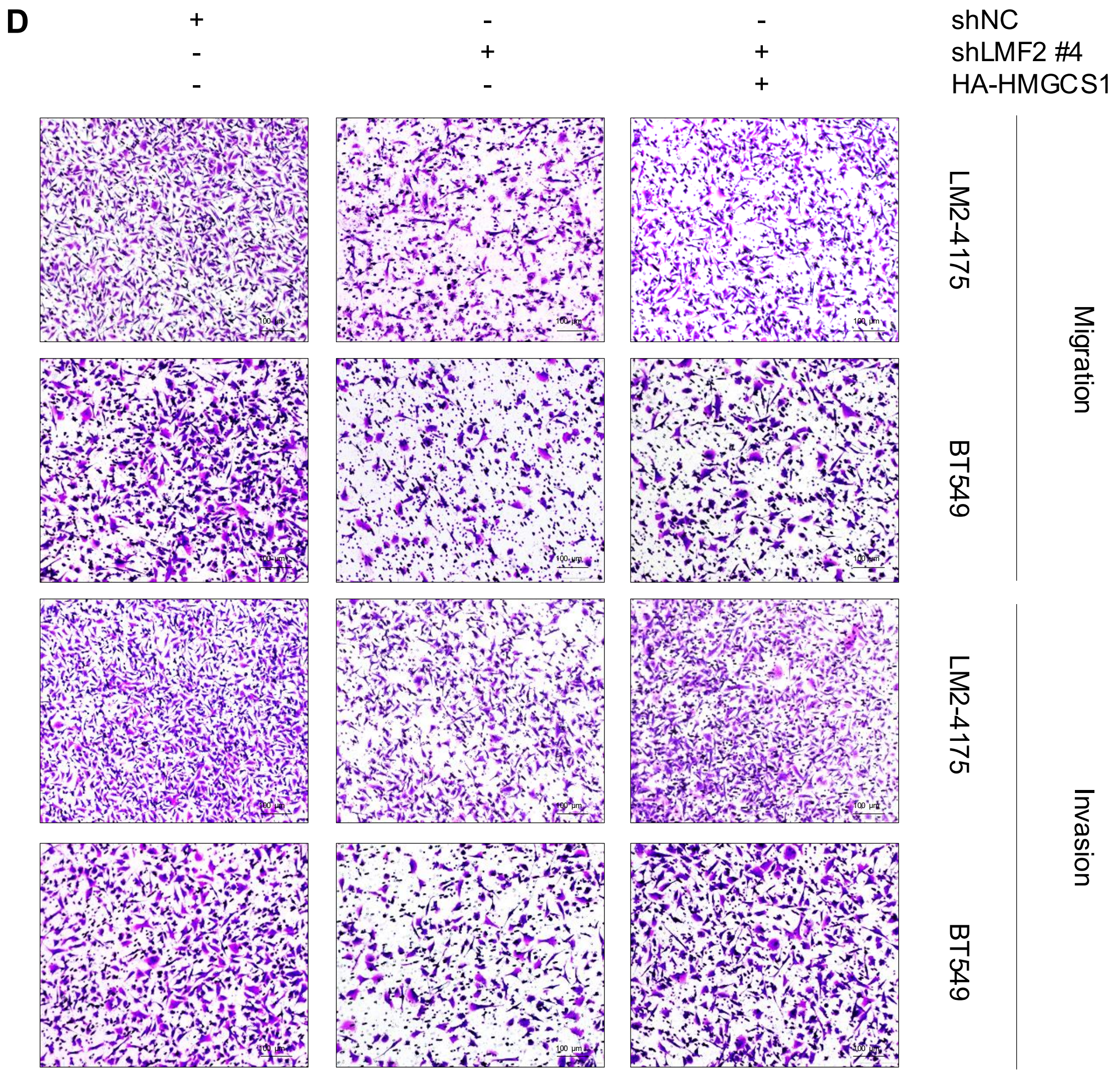

Supplementary Figure S14D (100x)

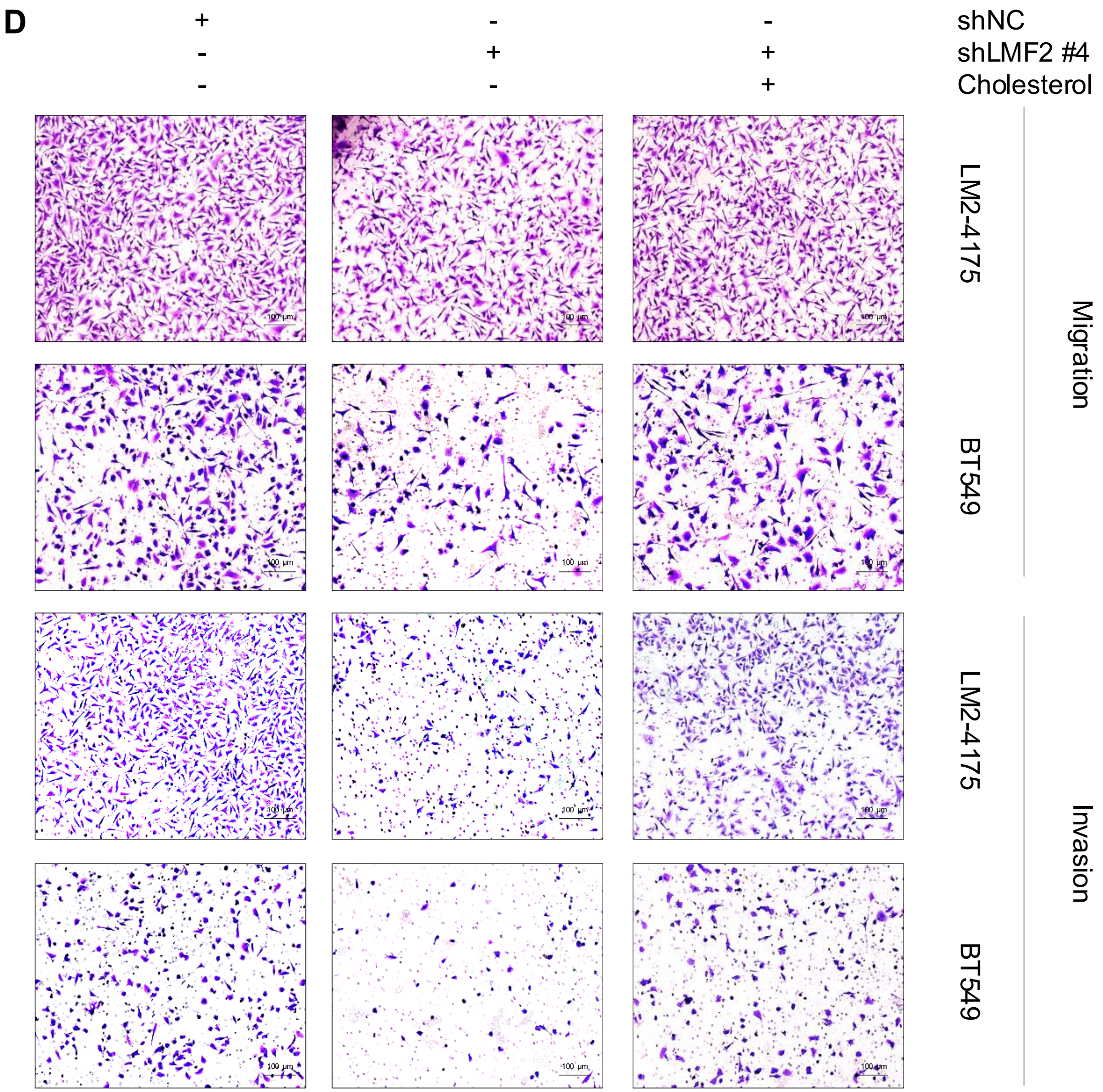

Supplement: Supplementary file 1 [file ADVS-9999-e78301-s001.pdf]
