# Supplementary material for: VCP‐Regulated LMF2 Stabilizes the Cholesterol Biosynthesis Enzyme HMGCS1 to Drive Cancer Progression and Confer Capecitabine Resistance in Triple‐Negative Breast Cancer
Source: Adv Sci (Weinh). 2026 Oct 20:e78301. Online ahead of print. doi: 10.1002/advs.78301 (PMC13683977; doi:10.1002/advs.78301)
Supplement: Supplementary file 2 [file ADVS-9999-e78301-s002.docx]

**Supplementary information for**

Zhao et al. VCP-regulated LMF2 stabilizes the cholesterol biosynthesis enzyme HMGCS1 to drive cancer progression and confer capecitabine resistance in triple-negative breast cancer

**This Supplementary information contains:**

Supplementary Figures: 15

Supplementary Tables: 8

**Supplementary Figures and Figure legends**

**Figure S1. Expression profiling of LMF2 across three major breast cancer subtypes using multiple public datasets.**

**(A)** Violin plots showing LMF2 mRNA expression levels across breast cancer subtypes (HR+, HER2+, and TNBC) in the CBCGA (n = 517 for HR+, n = 153 for HER2+, n = 127 for TNBC, cases with missing values were excluded), TCGA (n = 729 for HR+, n = 230 for HER2+, n = 183 for TNBC, cases with missing values were excluded), and METABRIC (n = 1140 for HR+, n = 220 for HER2+, n = 199 for TNBC, cases with missing values were excluded) transcriptomic cohorts.

**(B)** Violin plots showing LMF2 protein expression levels in the corresponding subtypes from the CBCGA (n = 152 for HR+, n = 63 for HER2+, n = 67 for TNBC, cases with missing values were excluded) and CPTAC (n = 65 for HR+, n = 10 for HER2+, n = 16 for TNBC, cases with missing values were excluded) proteomic datasets.

In violin plots, horizontal lines indicate the medians. Statistical significance was determined by two-tailed Wilcoxon rank-sum test (Mann–Whitney U test).

Notes: *, *p*<0.05; **, *p*<0.01; ***, *p*<0.001; ns, not significant.

**Figure S2. LMF2 drives the malignant progression of TNBC cells both *in vitro* and *in vivo*.**

(**A**) Immunoblotting analysis of LMF2 protein expression levels in seven representative human TNBC cell lines.

(**B**) MDA-231 and Hs578T cells stably expressing EV and Flag-LMF2 were subjected to colony formation assays. The representative images of survival colonies are shown.

(**C**) LM2-4175 and BT549 cells stably expressing shNC and shLMF2 (#4 and #5) were subjected to colony formation assays. The representative images of survival colonies are shown.

(**D**) LM2-4175 cells stably expressing shNC or shLMF2 (#4 and #5) were orthotopically injected into the mammary fat pad of 6-week-old BALB/c female nude mice (n=10). The representative images of removed xenograft tumors are shown.

(**E**) MDA-231 and Hs578T stably expressing LMF2 were subjected to Transwell migration and invasion assays. The representative images of migrated and invaded cells are shown (scale bars, 100 μm; 10× objective, total magnification 100×).

(**F**) LMF2-depleted LM2-4175 and BT549 cells were subjected to Transwell migration and invasion assays. The representative images of migrated and invaded cells are shown (scale bars, 100 μm; 10× objective, total magnification 100×).

(**G-H**) LM2-4175 cells stably expressing shNC or shLMF2 (#4 and #5) were injected into the tail vein of 7-week-old female BALB/c nude mice (n=6). Mice were sacrificed 6 weeks post-injection, and lungs were collected. The representative images of lung metastases (G) and HE-stained lung sections (H; scale bars, 500 μm for 3× images and 100 μm for 10× magnified images) from mice are shown.

**F****igure S3. LMF2 depletion significantly suppresses the proliferative, migratory, and invasive capacity of HCC1806 cells *in vitro*.**

(**A**) Validation of the expression status of LMF2 in LMF2-depleted HCC1806 cells by immunoblotting assays.

(**B-C**) HCC1806 cells stably expressing shNC and shLMF2 (#4 and #5) were subjected to colony formation assays. Representative images of survival colonies are shown in (B), and the corresponding quantitative results are shown in (C). Data are presented as mean ± SD (n = 3). Statistical significance was determined by ordinary one-way ANOVA followed by Šídák’s multiple comparisons test.

(**D-F**) LMF2-depleted HCC1806 cells were subjected to Transwell migration and invasion assays. Representative images of migrated and invaded cells are provided in (D; scale bars, 100 μm; 10× objective, total magnification 100×), and the corresponding quantitative results are shown in (E-F). Data are presented as mean ± SD (n = 3). Statistical significance was determined by ordinary one-way ANOVA followed by Šídák’s multiple comparisons test.

Notes: *, *p*<0.05; **, *p*<0.01; ***, *p*<0.001; ns, not significant.

**Figure S4. LMF2 confers capecitabine resistance in TNBC.**

(**A**) Correlation between LMF2 expression levels and chemotherapeutic drug sensitivity in the GDSC dataset.

(**B**) LM2-4175 and BT549 cells stably expressing shNC or shLMF2 (#4 and #5) were subjected to clonogenic survival assays with increasing concentrations of capecitabine (Cape) for 7–14 days. The representative images of survival colonies are shown.

(**C**) HCC1806 cells stably expressing shNC or shLMF2 (#4 and #5) were treated with increasing concentrations of capecitabine for 72 hours. Cell viability was measured by CCK-8 assays, and IC₅₀ values were calculated by nonlinear regression. Data are presented as mean ± SD (n = 3). Statistical significance of differences in IC_50_ curves compared with the shNC group was determined by extra sum-of-squares F-test.

(**D**) LM2-4175 cells stably expressing shNC or shLMF2 #4 were orthotopically implanted into the mammary fat pads of 6-week-old BALB/c female nude mice (n=6). Drug treatment began 24 days after implantation, with daily oral gavage of capecitabine (Cape; 250 mg/kg) or vehicle for 12 consecutive doses. The representative images of removed xenograft tumors are shown.

Notes: *, *p*<0.05; **, *p*<0.01; ***, *p*<0.001; ns, not significant.

**Figure S5. Depletion of LMF2 enhances capecitabine-induced DNA damage and apoptosis in TNBC cells *in vitro*.**

(**A**) Immunoblotting analysis of cleaved caspase-3 and γ-H2AX levels in LM2-4175 and BT549 cells stably expressing shNC or shLMF2 (#4) treated with 2 mM capecitabine for the indicated time points (0, 6, 12, and 24 hours).

(**B-C**) Flow cytometry analysis of apoptosis in LM2-4175 and BT549 cells stably expressing shNC or shLMF2 (#4) treated with DMSO or capecitabine (2 mM for LM2-4175; 3 mM for BT549) for 72 hours. Representative Annexin V-FITC/PE dot plots are shown in (B), and corresponding quantitative analysis of total apoptotic cells is presented in (C). Data are presented as mean ± SD (n = 3). Statistical significance was determined by ordinary one-way ANOVA followed by Šídák’s multiple comparisons test.

Notes: *, *p*<0.05; **, *p*<0.01; ***, *p*<0.001; ns, not significant.

**Figure S6. Identification and validation of LMF2-interacting proteins.**

(**A**) HEK293T cells stably expressing either empty vector (EV) or Flag-LMF2 were subjected to IP assays with anti-Flag antibody. The representative image of silver-stained gel is shown.

(**B**) Venn diagram showing proteins identified in HEK293T cells expressing EV or Flag-LMF2 with a unique spectral count ≥ 2.

(**C-D**) GO cellular component (C) and KEGG pathway (D) enrichment analyses of the 98 proteins uniquely identified in the Flag-LMF2 group (unique spectral count ≥ 2).

(**E-G**) Expression levels of CANX (E), RPN1(F), and VCP (G) in TNBC and normal breast tissues from the FUSCC proteomic cohort. Expression levels of CANX (E), RPN1 (F), and VCP (G) in TNBC (n = 82) and normal breast tissues (n = 66) from the FUSCC proteomic cohort (cases with missing values were excluded). Solid horizontal lines indicate median values. Statistical significance was determined by two-tailed Mann–Whitney U test.

(**H-J**) Correlation analysis of protein levels between LMF2 and CANX (H), RPN1 (I), or VCP (J) in TNBC based on the FUSCC-TNBC proteomic dataset (n = 62 pairs; cases with missing values were excluded). Linear regression lines (dashed) are shown. Statistical significance and correlation coefficients were determined by two-tailed Pearson correlation analysis.

**Figure S7. VCP stabilizes LMF2 by blocking its autophagic degradation.**

(**A**) MDA-231 cells stably expressing EV or Flag-LMF2 were treated with vehicle or 10 μM CB-5083 for 8 hours, followed by immunoblotting assays with the indicated antibodies.

(**B**) Immunoblotting analysis of VCP protein levels in LM2-4175 and BT549 cells stably expressing shLMF2 (#4 and #5).

(**C-D**) MDA-231 and Hs578T cells stably expressing EV or HA-VCP (B) and LM2-4175 and BT549 cells stably expressing shVCP (#3 and #4) (C) were subjected to RT-qPCR analysis of LMF2 mRNA levels. Data are presented as mean ± SD (n = 3). Statistical significance was determined by two-tailed unpaired Student’s t-test in (C), and ordinary one-way ANOVA followed by Šídák’s multiple comparisons test compared with the shNC group in (D) (ns, no significance).

(**E**) LM2-4175 and BT549 cells were treated with increasing concentrations of VCP inhibitor CB-5083 for 8 hours, followed by RT-qPCR analysis of LMF2 mRNA levels.

(**F**) LM2-4175 and BT549 cells stably expressing shNC or shVCP #4 were treated with 100 μg/mL cycloheximide (CHX) for the indicated times, followed by immunoblotting assays. The representative images of immunoblots are shown.

(**G**) MDA-231 and Hs578T cells were treated with 10 μM MG-132 for the indicated times and subjected to immunoblotting assays to examine LMF2 protein expression levels.

(**H**) MDA-231 and Hs578T cells were treated with 20 mM NH_4_Cl for the indicated times and subjected to immunoblotting assays to examine LMF2 protein expression levels.

(**I**) MDA-231 and Hs578T cells were treated with 1 μM rapamycin (Rapa) for the indicated times and subjected to immunoblotting assays to examine LMF2 protein expression levels.

**Figure S8.** **LMF2 undergoes autophagic degradation.**

(**A–B**) MDA-231 and Hs578T cells were transfected with siNC or siHSPA8 (#1 and #2) (A) or siLAMP2A (#1 and #2) (B). After 48 hours of transfection, total cellular lysates were subjected to immunoblotting assays with the indicated antibodies to assess LMF2 protein levels.

(**C**) MDA-231 and Hs578T cells were treated with 1 mM 3-MA for the indicated times and subjected to immunoblotting analysis to examine LMF2 protein expression levels.

(**D**) Immunoblotting analysis of LMF2 protein levels in MDA-231 and Hs578T cells stably expressing sgp62 (#1 and #2).

(**E**) Immunoblotting analysis of LMF2 protein levels in MDA-231 and Hs578T cells stably expressing sgTOLLIP (#1 and #2).

(**F**) Schematic representation of NBR1 protein domains.

(**G**) HEK293T cells were transfected with the indicated plasmids for 48 hours, and then incubated with 10 μM MG-132 for another 6 hours. Cellular lysates were collected and subjected to sequential IP and immunoblotting assays with the indicated antibodies.

**Figure S9. ATPase-dependent and domain-specific interactions of LMF2 with VCP and NBR1.**

(**A**) HEK293T cells were transfected with the indicated plasmids for 48 hours and subjected to IP and immunoblotting assays to examine the interaction of Flag-LMF2 with NBR1 and HA-VCP (wild-type or E578Q mutant).

(**B**) Schematic representation of LMF2 protein domains and truncated constructs.

(**C**) HEK293T cells were transfected with the indicated plasmids for 48 hours and subjected to IP and immunoblotting assays to examine the interaction between Flag-LMF2 and HA-VCP.

(**D**) HEK293T cells were transfected with the indicated plasmids for 48 hours and subjected to IP and immunoblotting assays to examine the interaction between HA-VCP and Flag-LMF2 truncated mutants (NT and CT).

(**E**) HEK293T cells were transfected with the indicated plasmids for 48 hours and subjected to IP and immunoblotting assays to examine the interaction between Flag-LMF2 and HA-NBR1.

(**F**) HEK293T cells were transfected with the indicated plasmids for 48 hours and subjected to IP and immunoblotting assays to examine the interaction between HA-NBR1 and Flag-LMF2 truncated mutants (NT and CT).

(**G**) Schematic representation of the interaction model showing that both VCP and NBR1 bind to the N-terminal domain (residues 1–350) of LMF2.

**Figure S10.** **LMF2 stabilizes HMGCS1 and enhances cholesterol production.**

(**A**) LM2-4175 cells stably expressing shNC or shLMF2 (#4 and #5) were subjected to immunoblotting assays to validate LMF2 expression levels.

(**B**) Venn diagram showing differentially expressed proteins upon LMF2 knockdown.

(**C**) The top 10 downregulated proteins following LMF2 knockdown in LM2-4175 cells, ranked by fold change.

(**D-F**) Correlation analysis of protein levels between LMF2 and HMGCS1 (D, n = 35 pairs), ME1 (E, n = 56 pairs), or ITGB6 (F, n = 17 pairs) in the FUSCC-TNBC proteomic dataset (cases with missing values were excluded). Linear regression lines (dashed) are shown. Statistical significance and correlation coefficients were determined by two-tailed Pearson correlation analysis.

(**G-H**) MDA-231 and Hs578T cells stably expressing Flag-LMF2 (G) and LM2-4175 and BT549 cells stably expressing shLMF2 (#4 and #5) were subjected to RT-qPCR analysis of HMGCS1 mRNA levels. Data are presented as mean ± SD (n = 3). Statistical significance was determined by two-way ANOVA followed by Šídák’s multiple comparisons test in (G), and two-way ANOVA followed by Dunnett’s multiple comparisons test compared with the shNC group in (H).

(**I**) LM2-4175 and BT549 cells expressing shNC or shLMF2 #4 were treated with 100 μg/mL CHX for the indicated times, followed by immunoblotting assays with the indicated antibodies.

(**J-K**) Quantification of relative cholesterol levels in the culture supernatant of LMF2-overexpressing MDA-231 and Hs578T cells (J) and LMF2-depleted LMF2-4175 and BT549 cells (K). Data are presented as mean ± SD (n = 3). Statistical significance was determined by two-tailed unpaired Student’s t-test in (J), and ordinary one-way ANOVA followed by Dunnett’s multiple comparisons test compared with the shNC group in (K).

(**L**) Quantification of relative cholesterol levels in the culture supernatant of LM2-4175 and BT549 cells stably expressing shNC or shLMF2 (#4) with or without HA-HMGCS1 overexpression. Data are presented as mean ± SD (n = 3). Statistical significance was determined by ordinary one-way ANOVA followed by Šídák’s multiple comparisons test.

(**M**) Quantification of LPL activity in control and LMF2-depleted LM2-4175 and BT549 cells. Data are presented as mean ± SD (n = 3). Statistical significance was determined by ordinary one-way ANOVA followed by Dunnett’s multiple comparisons test compared with the control (shNC) group.

Notes: *, *p*<0.05; **, *p*<0.01; ***, *p*<0.001; ns, not significant.

**Figure S11. HMGCS1 is primarily degraded via the autophagic-lysosomal pathway in TNBC cells.**

(**A**) MDA-231 and Hs578T cells were treated with 10 μM MG-132 for the indicated times and subjected to immunoblotting assays to examine HMGCS1 expression levels.

(**B**) MDA-231 and Hs578T cells were treated with 20 mM NH_4_Cl for the indicated times and subjected to immunoblotting assays to examine HMGCS1 expression levels.

(**C**) MDA-231 and Hs578T cells were treated with 1 μM Rapa for the indicated times and subjected to immunoblotting assays to examine HMGCS1 expression levels.

(**D–E**) MDA-231 and Hs578T cells were transfected with siNC or siHSPA8 (#1 and #2) (D) or siLAMP2A (#1 and #2) (E). After 48 hours of transfection, total cellular lysates were collected and analyzed by immunoblotting assays with the indicated antibodies to assess HMGCS1 protein levels.

(**F**) HEK293T cells were transfected with the indicated plasmids for 48 hours and subjected to immunoblotting assays to examine the protein expression levels of wild-type HA-HMGCS1 or its K273R mutant in the presence or absence of Flag-NDP52.

(**G**) Schematic representation of NDP52 protein domains.

**Figure S12. Domain mapping of HMGCS1 interactions with LMF2 and NDP52.**

(**A**) Schematic representation of HMGCS1 protein domains.

(**B**) HEK293T cells were transfected with the indicated plasmids for 48 hours and subjected to IP and immunoblotting assays to examine the interaction between Flag-LMF2 and HA-HMGCS1.

(**C**) HEK293T cells were transfected with the indicated plasmids for 48 hours and subjected to IP and immunoblotting assays to examine the interaction between Flag-LMF2 and HA-HMGCS1 truncated mutants (NT and CT).

(**D**) HEK293T cells were transfected with the indicated plasmids for 48 hours and subjected to IP and immunoblotting assays to examine the interaction between Flag-NDP52 and HA-HMGCS1.

(**E**) HEK293T cells were transfected with the indicated plasmids for 48 hours and subjected to IP and immunoblotting assays to examine the interaction between Flag-NDP52 and HA-HMGCS1 truncated mutants (NT and CT).

(**F**) Schematic representation of the interaction model showing that both LMF2 and NDP52 bind to the C-terminal domain (residues 251–520) of HMGCS1.

**Figure S13. Re-expression of HMGCS1 partially restores** **the impaired proliferative, migratory, and invasive potential of TNBC cells caused by LMF2 depletion.**

(**A-C**) LM2-4175 and BT549 cells stably expressing shNC or shLMF2 #4 alone or in combination with HA-HMGCS1 were subjected to CCK-8 (A) and colony formation assays (B-C). The representative images of survival colonies are shown in B and the corresponding quantitative results are shown in C. Data are presented as mean ± SD (n = 3). Statistical significance for cell viability in (A) was determined by two-way ANOVA followed by Šídák’s multiple comparisons test (significant differences at Day 7 are shown), and ordinary one-way ANOVA followed by Šídák’s multiple comparisons test in (C).

(**D-F**) LM2-4175 and BT549 cells stably expressing shNC or shLMF2 #4 alone or in combination with HA-HMGCS1 were subjected to Transwell migration and invasion assays. The representative images of migrated and invaded cells are shown in D (scale bars, 100 μm; 10× objective, total magnification 100×), and the corresponding quantitative results are shown in E and F, respectively. Data are presented as mean ± SD (n = 3). Statistical significance was determined by ordinary one-way ANOVA followed by Šídák’s multiple comparisons test.

Note: *** indicates statistically significant at *p*<0.001 level.

**Figure S14. LMF2 depletion impairs cellular phenotypes that are reversible by cholesterol supplementation, and enhances simvastatin sensitivity.**

(**A-C**) LM2-4175 and BT549 cells stably expressing shNC or shLMF2 #4 were treated with or without 2.5 μg/mL soluble cholesterol for 24 hours, and then subjected to CCK-8 (A) and colony formation (B-C) assays. The representative images of survival colonies are shown in B and the corresponding quantitative results are shown in C. Data are presented as mean ± SD (n = 3 independent experiments). Statistical significance for cell viability in (A) was determined by two-way ANOVA followed by Šídák’s multiple comparisons test (significant differences at Day 7 are shown), and ordinary one-way ANOVA followed by Šídák’s multiple comparisons test in (C).

(**D-F**) LM2-4175 and BT549 cells stably expressing shNC or shLMF2 #4 were treated with or without 2.5 μg/mL cholesterol for 24 hours, and then subjected to Transwell migration and invasion assays. The representative images of migrated and invaded cells are shown in D (scale bars, 100 μm; 10× objective, total magnification 100×), and the corresponding quantitative results are shown in E and F, respectively. Data are presented as mean ± SD (n = 3). Statistical significance was determined by ordinary one-way ANOVA followed by Šídák’s multiple comparisons test.

(**G**) LM2-4175 and BT549 cells stably expressing shNC or shLMF2 (#4 and #5) were treated with increasing concentrations of simvastatin for 72 hours. Cell viability was measured by CCK-8 assays, and IC₅₀ values were calculated by nonlinear regression. Data are presented as mean ± SD (n = 3). Statistical significance of differences in IC_50_ curves compared with the shNC group was determined by extra sum-of-squares F-test.

Note: *** indicates statistically significant at *p*<0.001 level.

**Figure S15. Single-cell transcriptomic characterization of the LMF2–VCP–HMGCS1 axis in TNBC cells.**

(**A**) Major cell populations identified in single-cell RNA sequencing data from the TNBC cohort (GEO: GSE246613). Clusters are color-coded by cell type.

(**B**) Expression profiles and functional marker co-localization across cells, with target genes (LMF2, VCP, and HMGCS1) shown in the top row, proliferation and stemness markers (MKi67 and CD44) in the middle, and EMT markers (CDH1, VIM, and ZEB1) at the bottom.

**Supplementary Tables**

**Tables S1. Chemical regents used in this study**

| Chemical reagents | Vendors | Catalog |
| --- | --- | --- |
| Phosphatase inhibitor cocktail | Bimake | B15001 |
| Protease inhibitor cocktail | Bimake | B14002 |
| Cycloheximide (CHX) | CST | 2112S |
| CB-5083 | MedChemExpress | [HY-12861](https://www.medchemexpress.cn/CB-5083.html) |
| Capecitabine | MedChemExpress | HYB0016 |
| 3-methyladenine (3-MA) | Selleck | S2767 |
| Bafilomycin A1 (Baf-A1) | Selleck | S1413 |
| Cholesterol | Selleck | S4154 |
| MG-132 | Selleck | S2619 |
| Rapamycin (Rapa) | Selleck | S1039 |
| Ammonium chloride (NH_4_Cl) | Sigma | A9434 |

**Table S2. Detailed information for all datasets analyzed in this study.**

| Dataset types | Sources | Identifiers |
| --- | --- | --- |
| FUSCC-TNBC transcriptomic dataset | NODE (https://www.biosino.org/node) | OEP000155 |
| FUSCC-TNBC proteomic dataset | iProX (http://www.iprox.org/) | IPX0003222000 |
| CBCGA transcriptomic dataset | GSA-Human (https://ngdc.cncb.ac.cn/gsa-human) | PRJCA017539 |
| CBCGA proteomic dataset | iProX (https://www.iprox.org/) | IPX0006535000 |
| TCGA breast cancer dataset | TCGA (https://portal.gdc.cancer.gov) | TCGA-BRCA |
| METABRIC breast cancer dataset | cBioPortal (https://www.cbioportal.org) | METABRIC |
| CPTAC breast cancer dataset | CPTAC | CPTAC-BRCA |
| ER-localized protein dataset | Gene Ontology | GO:0005783 |
| GDSC drug sensitivity dataset | GDSC (https://www.cancerrxgene.org) | GDSC |

**Table S3.** **List of primers used for molecular cloning in this study**

| Plasmids | Primers | Sequences |
| --- | --- | --- |
| Flag-LMF2 | Forward | AGGTCGACTCTAGAGGATCCCGCCACCATGGCGGGCTCCCGGCTCCC |
|  | Reverse | TCCTTGTAGTCCATACCGGTCTTCTTTCGCCGGGTGGTCCTCG |
| Flag-LMF2 NT | Forward | CTTGGGCTGCAGGTCGACTCTAGAGGATCCCGCCACCATGGCGGGCTCCCGGCT |
|  | Reverse | TTGTCATCGTCATCCTTGTAGTCCATACCGGTGGTTCTGGAGTGGATGGTGCGCTGCTGCCAG |
| Flag-LMF2 CT | Forward | CTTGGGCTGCAGGTCGACTCTAGAGGATCCCGCCACCATGACTTTCACCTTCCACCAGTTTTCTC |
|  | Reverse | TTGTCATCGTCATCCTTGTAGTCCATACCGGTCTTCTTTCGCCGGGTGGTCCTCGAACTACT |
| HA-HMGCS1 | Forward | GGATCTATTTCCGGTGAATTCGCCACCATGCCTGGATCACTTCCTTTG |
|  | Reverse | GGGATCCGCGGCCGCTCTAGATTAAGCGTAGTCTGGGACGTCGTATGGGTAATGTTCCCCATTACTAATGACAGC |
| HA-HMGCS1 NT | Forward | ACCGACTCTACTAGAGGATCTATTTCCGGTGAATTCGCCACCATGCCTGGATCACTTCCTTTGAATGC |
|  | Reverse | AGGGAGAGGGGCGGGATCCGCGGCCGCTCTAGATTAAGCGTAGTCTGGGACGTCGTATGGGTAATCATTTCCCTCTTTCTGCCACTG |
| HA-HMGCS1 CT | Forward | ACCGACTCTACTAGAGGATCTATTTCCGGTGAATTCGCCACCATGAAAGATTTTACCTTGAATGATTTTGGCTT |
|  | Reverse | GGAGGGAGAGGGGCGGGATCCGCGGCCGCTCTAGATTAAGCGTAGTCTGGG |
| HA-NBR1 | Forward | TCTACTAGAGGATCTATTTCCGGTGAATTCCTCGAGGCCACCATGGAACCACAGGTTACTC |
|  | Reverse | GGGAGAGGGGCGGGATCCGCGGCCGCTCTAGATTATGCATAATCCGGAACATCATACGGATAATAGCGTTGGCTGTACCAGTCGTTG |
| HA-NBR1 △PB1 | Forward | TCTACTAGAGGATCTATTTCCGGTGAATTCCTCGAGGCCACCATGGAACC |
|  | Reverse | CCCCAGACTGGTTCACAAGCTACCTGGAGACGTTCAGAGAACAAGTGGTTAACGAAACGGTT |
| HA-NBR1 △UBA | Forward | TTGATACCAGCCGCCCCCTGGGGGATTCTATGTACAGCTCTGCGCTCTCACAGCCAGGCCT |
|  | Reverse | GGGAGAGGGGCGGGATCCGCGGCCGCTCTAGATTATGCATAATCCGGAACATCATACGGATAATAGC |
| Flag-NDP52 | Forward | ACCTCCATAGAAGATTCTAGAGCCACCATGGAGGAGACCATCAAAGATCCC |
|  | Reverse | ATCCGATTTAAATTCGAATTCTTACTTGTCATCGTCGTCCTTGTAATCGAGAGAGTGGCAGAACACGTG |
| Flag-NDP52 △CC | Forward | AATGAGGAAGACATCCTGGATTTTAATTCTTTGCCGTAT |
|  | Reverse | AGAATTAAAATCCAGGATGTCTTCCTCATTTTCTGGACG |
| Flag-NDP52 △SKICH | Forward | ACCTCCATAGAAGATTCTAGAGCCACCGAAAATGAGGAAGACATCCTGGTTGTT |
|  | Reverse | ATCCGATTTAAATTCGAATTCTTACTTGTCATCGTCGTCCTTGTAATCGAGAGAGTGGCAGAACACGTG |
| Flag-NDP52 △LIM-L | Forward | ACCTCCATAGAAGATTCTAGAGCCACCATGGAGGAGACCATCAAAGATCCC |
|  | Reverse | ATCCGATTTAAATTCGAATTCTTACTTGTCATCGTCGTCCTTGTAATCGATGGAGAGCGGGCTGGG |
| HA-VCP E578Q | Forward | GGACCTCCTGGAACAGGAAAGACCCTGATTGCTCGAGCTGTAGCAAATGAGACTGGA |
|  | Reverse | CGGAGCGATCGCAGATCCTTCGCGGCCGCGGATCCATTTAAATTCGAATTCTTAAGC |
| HA-HMGCS1 K273R | Forward | GACTCTACTAGAGGATCTATTTCCGGTGAATTCGCCACCATGCCTGGATCACTTCC |
|  | Reverse | GATGCAAGGGAACCATATACTGAAGATGTGTACATATTTCCATTTTGATTTGATAC |

**Table S4. Targeting sequences for shRNAs**

| Plasmids | Primers | Sequences |
| --- | --- | --- |
| shLMF #4 | Forward | CCGGGGACGGAGATCGAGTTCATGTCTCGAGACATGAACTCGATCTCCGTCCTTTTTG |
|  | Reverse | AATTCAAAAAGGACGGAGATCGAGTTCATGTCTCGAGACATGAACTCGATCTCCGTCC |
| shLMF #5 | Forward | CCGGGCTCAGGCAGTTTGGACTACACTCGAGTGTAGTCCAAACTGCCTGAGCTTTTTG |
|  | Reverse | AATTCAAAAAGCTCAGGCAGTTTGGACTACACTCGAGTGTAGTCCAAACTGCCTGAGC |

**Table S5. Targeting sequences for sgRNAs**

| **Genes** | **Sequences** |
| --- | --- |
| sgATG5 #1 | GGCCATCAATCGGAAACTCA |
| sgATG5 #2 | CAGTGTGGGTGGGAATCCAG |
| sgNBR1 #1 | CACCGACTGAGCATTGGCATAACGGGTTT |
| sgNBR1 #3 | CACCGGTAGGCATTGAGACTTCTGAGTTT |
| sgp62 #1 | CACCGGATATCGATGTGGAGCACGGGTTT |
| sgp62 #2 | CACCGCGACTTGTGTAGCGTCTGCGGTTT |
| sgNDP52 #1 | CACCGAGAGTGGATCAGCTTCAGGTGTTT |
| sgNDP52 #3 | CACCGGTATTACCAGTTCTGCTATGGTTT |
| sgTOLLIP #1 | CACCGGGGCCGACTGAACATCACGGGTTT |
| sgTOLLIP #2 | CACCGTGGATGACCTTATTCCAGCGGTTT |

**Table S6. Targeting sequences for siRNAs**

| Genes | Primers | Sequences |
| --- | --- | --- |
| siHSPA8 #1 | Forward | GCUGUUGUCCAGUCUGAUATT |
|  | Reverse | UAUCAGACUGGACAACAGCTT |
| siHSPA8 #2 | Forward | CCAAGCAGACACAGACCUUTT |
|  | Reverse | AAGGUCUGUGUCUGCUUGGTT |
| siLAMP2A #1 | Forward | CGCUAUGAAACUACAAAUATT |
|  | Reverse | UAUUUGUAGUUUCAUAGCGTT |
| siLAMP2A #2 | Forward | GCUCUACUUAGACUCAAUATT |
|  | Reverse | UAUUGAGUCUAAGUAGAGCTT |

**Table S7. List of antibodies used in this study**

| Antibodies | Vendors | Catalog | Hosts | Working concentration |
| --- | --- | --- | --- | --- |
| CANX | Abcam | AB92573 | Rabbit | 1:20000 (WB)  1:500 (IF) |
| HSPA8 | Abcam | AB51052 | Rabbit | 1:3000 (WB) |
| LAMP2A | Abcam | AB125068 | Rabbit | 1:2000 (WB) |
| OPTN | Abcam | AB213556 | Rabbit | 1:1000 (WB) |
| p62 | Abcam | AB109012 | Rabbit | 1:10000 (WB) |
| VCP | Abcam | AB109240 | Rabbit | 1:5000 (WB) |
| ATG5 | Abclonal | A19677 | Rabbit | 1:1000 (WB) |
| ATP1A1 | Abclonal | A11683 | Rabbit | 1:100 (IF) |
| NBR1 | Abclonal | A3949 | Rabbit | 1:500 (WB) |
| RPN1 | Abclonal | A24537 | Rabbit | 1:1000 (WB) |
| TAX1BP1 | Abclonal | A19587 | Rabbit | 1:1000 (WB) |
| TOLLIP | Abclonal | A23780 | Rabbit | 1:1000 (WB) |
| HA | CST | 3724S | Rabbit | 1:3000 (WB)  1:200 (IF) |
| p21 | CST | 2947S | Rabbit | 1:1000 (WB) |
| LMF2 | Invitrogen | PA5-97930 | Rabbit | 1:100 (IF) |
| HK1 | Proteintech | 19662-1-AP | Rabbit | 1:200 (IF) |
| HMGCS1 | Proteintech | 17643-1-AP | Rabbit | 1:1500 (WB) |
| LMF2 | Proteintech | 16249-1-AP | Rabbit | 1:1500 (WB) |
| M6PR | Proteintech | 20253-1-AP | Rabbit | 1:200 (IF) |
| ME1 | Proteintech | 16619-1-AP | Rabbit | 1:2000 (WB) |
| NDP52 | Proteintech | 12229-1-AP | Rabbit | 1:1000 (WB) |
| STX6 | Proteintech | 60059-1-lg | Rabbit | 1:200 (IF) |
| Flag | Sigma | F1804 | Mouse | 1:3000 (WB)  1:200 (IF) |
| Vinculin | Sigma | V9131 | Mouse | 1:5000 (WB) |

Note: WB, Western blotting; IF, immunofluorescent staining.

**Table S8. Primers for qPCR analysis**

| Genes | Primers | Sequences |
| --- | --- | --- |
| LMF2 | Forward | CTCACCTACCACTACGAGACC |
|  | Reverse | GCACAGCGATCTCAATTAGGAAG |
| HMGCS1 | Forward | GATGTGGGAATTGTTGCCCTT |
|  | Reverse | ATTGTCTCTGTTCCAACTTCCAG |
| VCP | Forward | GCAGTTGTTCCGAGGTGACA |
|  | Reverse | GCACATGGATACGTTTGCCG |
| GAPDH | Forward | GGAGCGAGATCCCTCCAAAAT |
|  | Reverse | GGCTGTTGTCATACTTCTCATGG |
